# Triphenylmethyl Group as a Highly Diastereoselective *exo*,*endo*-Auxiliary in Double Diels–Alder Reactions with 2*H*-Pyran-2-ones

**DOI:** 10.3390/molecules31081301

**Published:** 2026-04-16

**Authors:** Marko Krivec, Žiga Štirn, Marijan Kočevar, Krištof Kranjc

**Affiliations:** Faculty of Chemistry and Chemical Technology, University of Ljubljana, Večna pot 113, SI-1000 Ljubljana, Slovenia; marko.krivec@fkkt.uni-lj.si (M.K.); ziga.stirn@gmail.com (Ž.Š.); marijan.kocevar@fkkt.uni-lj.si (M.K.)

**Keywords:** cycloaddition, steric hindrance, microwave chemistry, bicyclo[2.2.2]octenes, diastereoselectivity

## Abstract

The influence of steric hindrance caused by the dienophiles on the stereoselectivity of cycloadditions of 2*H*-pyran-2-ones with maleimides was investigated in this study. It was found that sufficiently bulky *N*-substituents on the maleimides (such as *N*-triphenylmethyl) can cause the cycloaddition to proceed differently than expected, thus yielding asymmetric *exo*,*endo*-bicyclo[2.2.2]octenes instead of the commonly obtained symmetric *exo*,*exo* products. Furthermore, the incorporation of an *N*-triphenylmethyl group, which induces highly diastereoselective formation of asymmetric *exo*,*endo* adducts and can later be easily removed under acidic conditions, can be described as an example of an efficient *exo*,*endo*-diastereoselective auxiliary.

## 1. Introduction

The high importance of pericyclic reactions in general and, in particular, the Diels–Alder reaction, as versatile tools for constructing novel C–C bonds as fragments of larger scaffolds [1,2,3,4,5], stems in great part from detailed knowledge of their theoretical background [6,7,8,9,10,11,12]. This knowledge, rooted in the pioneering work of Woodward and Hoffmann [6,9], is directly reflected in the ability to reliably predict the reactivity of dienes and dienophiles in such transformations, as well as their regio- and stereoselectivities. This is obviously of utmost importance for the successful application of Diels–Alder reactions, but there are still some cases where certain surprises might lurk, and the observed stereoselectivity differs from what is anticipated. Recently, it was demonstrated that the kinetic preference for the *endo* attack in cycloadditions (the so-called *endo rule*) is often not (only) a consequence of orbital interactions, but is mainly caused by the unfavorable steric arrangement encountered in the transition state arising from an *exo* attack [8,11]. One of the rare and unusual cases where insurmountable steric hindrance causes a switch in the stereochemical course of a cycloaddition of 2*H*-pyran-2-ones from the otherwise preferred *endo* attack to an *exo* attack is described in the present article.

**Scheme 1:**
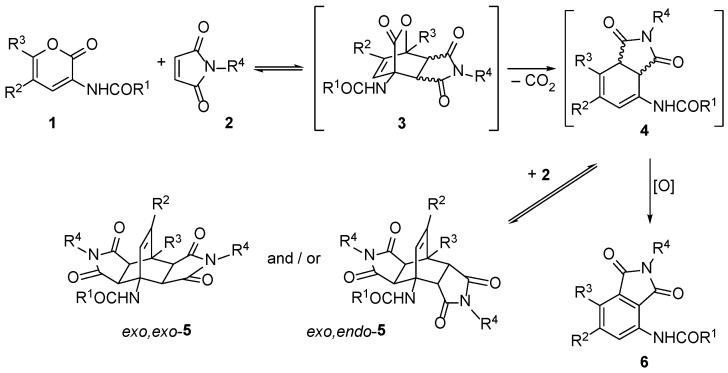
General reaction pathway of cycloadditions between 2*H*-pyran-2-ones **1** and maleimides **2**, yielding bicyclo[2.2.2]octenes **5** or isoindoles **6**.

Primary cycloadducts **3** containing a CO_2_-bridge, formed by the Diels–Alder reactions of substituted 3-acylamino-2*H*-pyran-2-ones **1** and suitable dienophiles such as *N*-substituted maleimides **2**, often undergo a retro-hetero-Diels–Alder cycloaddition, resulting in the elimination of a CO_2_ molecule (Scheme 1) [13]. The resulting cyclohexadiene intermediates **4** can be further transformed either into isoindole products **6** (through an additional oxidation step, often accelerated by suitable heterogeneous dehydrogenation catalysts) [14,15,16] or into bicyclo[2.2.2]octenes **5** (via a second cycloaddition step with **2**) [17,18,19]. These compounds **5**, also known as double cycloadducts or “butterfly”-like diazatetradecenes [19], are of great interest as they provide important insights into the stereoselectivity of these transformations and can be used for the subsequent synthesis of various compounds of pharmacological interest [13,20]. So far, in most cases, symmetric products *exo*,*exo*-**5** have been obtained [17,18,19]; only in special cases involving sterically highly demanding 2*H*-pyran-2-ones **1**, such as those fused with a cyclooctane ring (**1**, R^2^–R^3^ = -[CH_2_]_6_-) and with dienophiles **2** of sufficient size (R^4^ at least a Me group), have asymmetric cycloadducts *exo*,*endo*-**5** been isolated [21]. The only other approach leading to asymmetric bicyclo[2.2.2]octene adducts from 2*H*-pyran-2-ones is the application of photochemical conditions [22]. An alternative, though more circuitous, route to some *exo*,*endo* derivatives relies on chemical desymmetrization methods starting from various symmetric bicyclo[2.2.2]octene derivatives [23].

In addition to using conventional reaction conditions for the formation of **5** and **6** (e.g., heating under reflux), many of these adducts have also been successfully prepared under microwave irradiation [18,24,25] or at high pressures (13–15 kbar) [26,27]. Notably, the latter approach was the only suitable method for the synthesis of thermally sensitive intermediates of types **3** and **4** [27]. Moreover, a recent shift toward the use of conditions more consistent with the paradigms of modern green chemistry [28,29,30] is evident.

Herein we report the first example of a Diels–Alder reaction in which the significant steric hindrance of dienophiles **2** prevents the second cycloaddition step between **4** and **2** from proceeding with the same stereocourse as the first step, which, according to the literature data, preferentially occurs via the kinetically favored *endo* attack; where *endo* refers to the tendency for dienophile substituents to be oriented in the favored transition state so that they lie directly above the residual unsaturation of the diene, as described in [31]. Instead, the stereochemistry of the second step follows the opposite pathway to yield asymmetric *exo*,*endo*-**5** adducts. From a synthetic point of view, this strategy, which employs the *N*-triphenylmethyl-substituted **2** as an effective diastereoselective auxiliary that can be easily removed after the cycloaddition, provides a facile entry into the class of asymmetric *exo*,*endo*-**5**. This new pathway is highly complementary to our previously described strategy, in which asymmetric adducts are formed due to the steric effects of a large ring fused to the pyran-2-one system [21], and represents a substantial addition to the often-described synthetic applications of 2*H*-pyran-2-one derivatives [32,33,34,35].

## 2. Results and Discussion

Based on our previous experience in this field [13,15,16,18,21,27], we anticipated that an approach to the synthesis of asymmetric cycloadducts *exo*,*endo*-**5** that is complementary to the one described previously [21] could be realized by starting from sterically undemanding substituted 2*H*-pyran-2-ones **1** [36]. However, we recognized that this objective could only be achieved if the dienophile partners **2** are sufficiently sterically bulky. Therefore, we initially examined the reactions between the 5-methoxyphenyl derivative **1A**, an example of an electron-rich 2*H*-pyran-2-one previously shown to form only symmetric bicyclo[2.2.2]octene adducts (*exo*,*exo*-**5**) upon cycloaddition of *N*-ethyl- or *N*-phenylmaleimide [18]. For the sterically demanding dienophiles **2** in this study, we selected the following substituted maleimides: *N*-[4-(*tert*-butyl)phenyl]- (**2b**), *N*-[2-(*tert*-butyl)phenyl]- (**2c**), *N*-(*tert*-butyl)- (**2d**), and *N*-triphenylmethylmaleimide (**2e**). For all these dienophiles **2**, we found that they provided sufficient steric hindrance in the second cycloaddition step (i.e., **4** ⟶ **5**) to yield asymmetric bicyclo[2.2.2]octenes (*exo*,*endo*-**5**), either as exclusive products or as mixtures with the symmetric *exo*,*exo*-**5** derivatives. To evaluate the effects of steric hindrance in **2**, preliminary reactions with **2a**–**e** were conducted in closed vessels using toluene as the solvent and under microwave irradiation (1 h at 180 °C), which was sufficient for the complete conversion of **1a** (Table 1).

As expected, the results of these cycloadditions show that as the steric demand of dienophiles **2** increases and the distance between the reacting double bond of dienophiles **2** and the site of highest steric congestion decreases, the amount of the asymmetric *exo*,*endo* adduct **5A** increases. The most sterically congested dienophile, **2e**, which possesses a triphenylmethyl group, displays the highest stereoselectivity, yielding nearly exclusively asymmetric *exo*,*endo*-**5Ae** when cycloadded on **1A** (Table 1, Entry 5). In contrast, the other dienophiles **2b**–**d**, which are less sterically congested, provide only mixtures of *exo*,*endo* and *exo*,*exo* cycloadducts **5Ab**–**d** upon reaction with **1A** (Table 1, Entries 2–4). We were able to separate these mixtures by column chromatography on silica gel and characterize each stereoisomer of product **5A** individually. Specifically, **2b**, which has the *tert*-butyl group at the position most distant from the maleimide nitrogen atom, gives the smallest amount of the asymmetric adduct (*exo*,*endo*-**5Ab** : *exo*,*exo*-**5Ab** = 1 : 3.6); **2d**, with the *tert*-butyl group directly attached to the maleimide nitrogen, provides the largest amount of the asymmetric adduct (*exo*,*endo*-**5Ad** : *exo*,*exo*-**5Ad** = 1 : 1.1); and **2c**, with the *tert*-butyl group at an intermediate position, gives an amount of the asymmetric adduct between the other two values (*exo*,*endo*-**5Ac** : *exo*,*exo*-**5Ac** = 1 : 1.6). On the other hand, the absence of the *N*-substituent on the dienophile (i.e., **2a**) led primarily to the formation of the symmetric *exo*,*exo*-**5Aa** product (Table 1, Entry 1). It should also be noted that an insignificant amount (less than 5%) of the corresponding isoindole product **6A** was formed in the cases discussed above.

To develop reaction conditions favorable for yielding asymmetric *exo*,*endo* adducts, we conducted a model reaction of **1A** and **2c** under microwave irradiation, while screening the effects of various additives, temperature changes, and solvents used on the outcome of this reaction (Table 2). Unfortunately, none of the additives previously known to influence the stereoselectivity of Diels–Alder reactions [37,38] increased the amount of *exo*,*endo*-**5Ac** (Table 2, Entry 1). The amount of *exo*,*endo* adduct did not increase with the addition of a Brønsted acid such as benzoic acid (Table 2, Entry 2), a Lewis acid such as ZnCl_2_ (Table 2, Entry 3), the ionic liquid [emim]BF_4_ (Table 2, Entry 4), or various organocatalysts (Table 2, Entries 5–7). Although the addition of bases such as Et_3_N, quinine, or cinchonine (Table 2, Entries 8–10) slightly increased the amount of the desired *exo*,*endo*-**5Ac**, it also led to a significant increase in the formation of the undesired isoindole **6Ac** (as demonstrated by ^1^H NMR spectroscopy).

Consequently, we continued studying the model reaction without additives to evaluate the effect of reaction temperature. Increasing the temperature (while reducing the reaction time) resulted in a higher yield of the asymmetric adduct **5Ac**, while the formation of isoindole **6Ac** remained favorably low (Table 2, Entries 11–14). We then performed a series of experiments to determine the influence of the solvent on the model reaction (Table 2, Entries 15–24). In xylene (a mixture of isomers), the reaction proceeded less efficiently than in toluene, yielding a similar ratio of **5Ac** stereoisomers, but a larger amount of isoindole **6Ac** (Table 2, Entries 14 and 15). In contrast, anisole and *n*-heptane, the least-polar among the tested solvents, produced a higher amount of undesired *exo*,*exo*-**5Ac** with a moderately low quantity of **6Ac** (Table 2, Entries 16 and 17). Switching to a more polar and protic reaction medium disrupted the previously observed ratio between *exo*,*endo*- and *exo*,*exo*-**5Ac**. Neither 3-pentanone (Table 2, Entry 18) nor various alcohols (Table 2, Entries 19–21) improved selectivity toward the asymmetric product. Water was even less effective than previous examples (Table 2, Entry 22). Acetonitrile, on the other hand, yielded the largest amount of aromatic **6Ac** (Table 2, Entry 23), while the amount of the desired asymmetric product was the lowest among all solvents tested. When 1,4-dioxane was used (Table 2, Entry 24), conversion was very low, and the amount of asymmetric product was mediocre. Comparing these results, it is evident that non-polar solvents combined with high temperature are essential for achieving preferential formation of asymmetric *exo*,*endo*-**5Ac** adducts.

To further investigate the factors responsible for the stereoselectivity of these transformations, we studied the cycloaddition of various **2** with other 3-benzoylamino-2*H*-pyran-2-ones **1B**–**K** (Table 3, Entries 1–5, 7, 9, 11–17) under microwave irradiation (in toluene at 160 or 180 °C for 1 or 2 h in most cases). With maleimides **2a**–**d**, which are less sterically hindered than **2e**, symmetric *exo*,*exo*-**5Ba**–**d** were obtained as the major products (Table 3, Entries 1–4), with ratios of asymmetric to symmetric products ranging from 1 : 5.6 to 1 : 1.1. However, regardless of the electronic or steric properties (i.e., substituents at position 5) of the dienes **1**, the outcome of the cycloaddition with **2e** was analogous in all cases: highly dominant formation of the asymmetric *exo*,*endo*-**5B**–**Je** double cycloadducts.

**Scheme 2:**
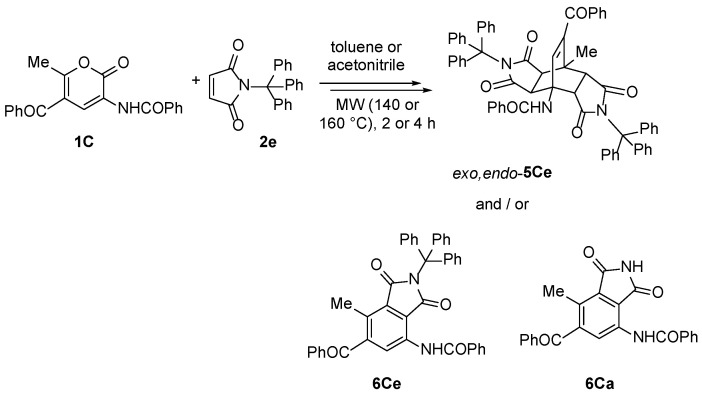
Synthesis of the bicyclo[2.2.2]octene derivative *exo*,*endo*-**5Ce** and isoindoles **6C**.

It is important to note that in one case (the reaction of **1C** with **2e**, Scheme 2), we had to modify the general reaction conditions to obtain pure **5Ce**, as the conditions used in other cases were not suitable. Specifically, when the cycloaddition of **1C** and **2e** was carried out at 160 °C in toluene under microwave irradiation for 2 h, a substantial amount of the undesired isoindole product **6Ce** was observed (*exo*,*endo*-**5Ce** : **6Ce** = 1 : 0.25). Changing the solvent from toluene to the more polar acetonitrile was detrimental for our purpose, as after 2 h of microwave irradiation at 160 °C, no double cycloadduct **5Ce** was formed; however, the isoindole **6Ce** and its *N*-deprotected derivative **6Ca** were detected in the crude reaction mixture (**6Ce** : **6Ca** = 1 : 2). Therefore, we further varied the reaction time and temperature (using toluene as the solvent) and found that increasing the time to 4 h and decreasing the temperature to 140 °C was sufficient to yield predominantly the desired asymmetric double cycloadduct *exo*,*endo*-**5Ce** (accompanied by approximately 10% of the isoindole adduct **6Ce**) (Table 3, Entry 7). Another case where the general reaction conditions required slight modification was the cycloaddition of **2e** with **1J** (Table 3, Entry 16). Here, due to the very low reactivity of the starting 2*H*-pyran-2-one **1J**, the reaction time was increased to 5 h (at 160 °C to minimize the formation of the undesired isoindole **6Je**), but the yield of the asymmetric *exo*,*endo*-**5Je** remained below average (56%).

**Scheme 3:**
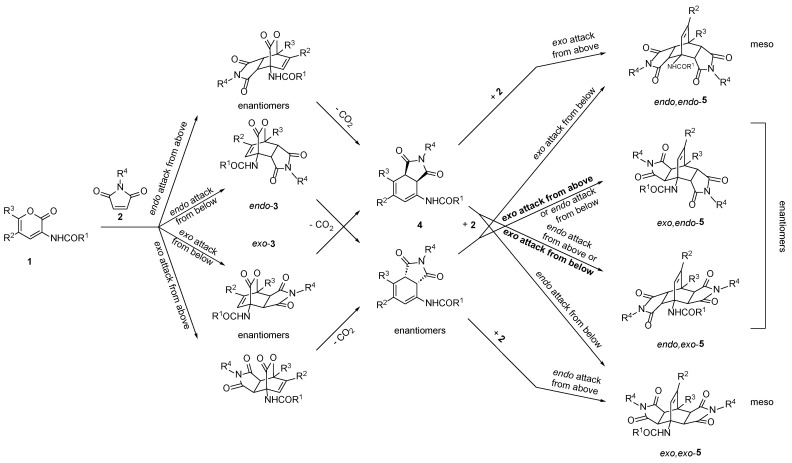
Schematic representation of the theoretically possible stereochemical pathways leading to four stereoisomeric bicyclo[2.2.2]octenes **5**, via *endo*-**3** or *exo*-**3** (each of which exists as a pair of enantiomers) and an enantiomeric pair of **4**. The attacks in the final cycloaddition step (i.e., **4** ⟶ **5**) that are presumed to be sterically favorable are highlighted in bold.

The results of these cycloadditions, along with data from the literature [21,27], show that the first cycloaddition step (i.e., **1** ⟶ **3**) should occur via an *endo* attack as expected, since it is kinetically favored over an *exo* attack. However, the elimination of CO_2_ that follows erases the stereochemical information from the first step; the two pairs of enantiomers of *endo*-**3** and *exo*-**3** initially formed [21,27] are reduced to a single pair of enantiomeric cyclohexadiene systems **4** (Scheme 3). The second cycloaddition step (i.e., **4** ⟶ **5**) can yield asymmetric *exo*,*endo*-**5** if it proceeds via a stereocourse opposite to the first step; therefore, if the first step took place via an *endo* attack, the second step must follow an *exo* attack from the opposite side of the diene system, where the previously incorporated dienophile ring is located, yielding the observed asymmetric *exo*,*endo*-**5**. Significant steric hindrance and congestion in the second transition state minimize the formation of symmetric *exo*,*exo*-**5** and instead favor the attack that yields asymmetric *exo*,*endo*-**5**, which are, understandably, formed as racemates [8,11].

The rather harsh reaction conditions required for the cycloadditions between **1** and **2**, as well as for the elimination of CO_2_, in certain cases also enable the aromatization of the cyclohexadiene intermediates **4** (at least in the reaction between **1C** and **2e**), resulting in the formation of isoindoles **6** as side products. Notably, pure isoindoles **6** can be prepared via direct cycloaddition between the corresponding 2*H*-pyran-2-ones **1** and maleimides **2**, facilitated by the application of a heterogeneous dehydrogenation catalyst such as active carbon Darco KB (which prevents the formation of bicyclo[2.2.2]octenes **5**), as demonstrated previously [16]. Although this approach with maleimide (**2a**) is feasible, the use of *N*-triphenylmethylmaleimide (**2e**) has proven inappropriate because the heterogeneous catalyst causes cleavage of the N–C bond in **2e**, consequently favoring the formation of a deprotected derivative **6Ca** (R^4^ = H).

To determine whether the symmetric adducts *exo*,*exo*-**5** can eventually convert into the corresponding asymmetric adducts *exo*,*endo*-**5** during the reactions, we investigated the transformation of pure *exo*,*exo*-**5Ad** under conditions analogous to those applied for the cycloadditions (i.e., microwave irradiation at 160 °C for 2 h in toluene). ^1^H NMR analysis of the crude reaction mixture showed that, at best, only a trace of *exo*,*endo*-**5Ad** was formed, with the vast majority of the starting material remaining unchanged and only a negligible amount of additional degradation material observed. This proves that the asymmetric *exo*,*endo*-**5** are formed directly and not via symmetric adducts followed by (intramolecular) isomerization.

On the other hand, we have observed that prolonging reaction times, especially at higher temperatures, consistently increases the amount of isoindole products **6** formed via thermal dehydrogenation of **4** (predominantly taking place via the hydrogen transfer to the maleimides **2**, which are reduced to the corresponding succinimides [16] or possibly also partially via the acceptorless mechanism [39,40,41]), consequently decreasing the yields of **5**.

For our further studies, we were eager to obtain asymmetric bicyclo[2.2.2]octenes *exo*,*endo*-**5** with less bulky R^4^ groups. Direct cycloaddition of the appropriate maleimides **2** has already proven inadequate for accessing these compounds. For example, cycloaddition between **1A** and maleimide (**2a**) yields exclusively symmetric *exo*,*exo*-**5Aa** (Table 1, Entry 1). To obtain the desired asymmetric counterpart, we devised an alternative route: a thermal, acid-promoted elimination of the *N*-triphenylmethyl substituent from asymmetric bicyclo[2.2.2]octenes *exo*,*endo*-**5** (Table 4). We established that 1 h of microwave irradiation at 100 °C in a mixture of trifluoroacetic acid (TFA) and *n*-BuOH (1 : 3) was appropriate, as it resulted in complete conversion to *N*-unsubstituted *exo*,*endo*-**5Aa** in high yield (Table 4, Entry 1). It is important to note that no isomerization to the symmetric *exo*,*exo*-**5Aa** was observed in the ^1^H NMR spectrum of the crude reaction mixture. Therefore, cycloaddition of *N*-triphenylmethyl group-containing dienophiles (i.e., **2e**) leads to diastereospecific formation of asymmetric bicyclo[2.2.2]octene frameworks (*exo*,*endo*-**5**) as racemates, and the triphenylmethyl group can be easily removed after cycloaddition; thus, it acts as a diastereospecific *exo*,*endo*-auxiliary. Besides the formation of *exo*,*endo*-**5Aa**, the same strategy was applied to *exo*,*endo*-**5B**–**Ea**, which were prepared under nearly identical conditions as above from *exo*,*endo*-**5B**–**Ee** in excellent yields (Table 4, Entries 2–5). In these cases, as well, direct cycloaddition between **1B**–**E** and **2a** is not applicable, as symmetric *exo*,*exo*-**5B**–**Ea** are formed overwhelmingly.

We also wanted to investigate the possible effects of the size of the 3-acylamino moieties (i.e., groups bound to position 3 of the starting compounds **1**). To this end, we replaced the benzoyl group in **1A** (R^1^ = Ph) with the much smaller acetyl group (**1**, R^1^ = Me) and reacted the resulting **1K** (prepared by removing the 3-benzoyl group from **1A** under acidic conditions, followed by the introduction of a 3-acetyl group, according to a modified procedure as described previously [42]) with *N*-triphenylmethylmaleimide (**2e**), obtaining a result analogous to those observed for **1A**–**J**, namely, predominantly asymmetric *exo*,*endo*-**5Ke** was formed (Table 3, Entry 17). Clearly, the size of the 3-acylamino group (i.e., R^1^) does not play a significant role in determining the stereoselectivity of these transformations; the R^4^ group of the maleimides **2** has a much greater impact. This is not surprising, as the NHCOR^1^ group is much more flexible (due to the –NH–CO– linker) than R^4^, making it easy to imagine that its conformational freedom does not interfere with the attack of the dienophile during the cycloaddition, and therefore does not influence the ratio of asymmetric *exo*,*endo*-**5** to symmetric *exo*,*exo*-**5** formation.

**Figure 1:**
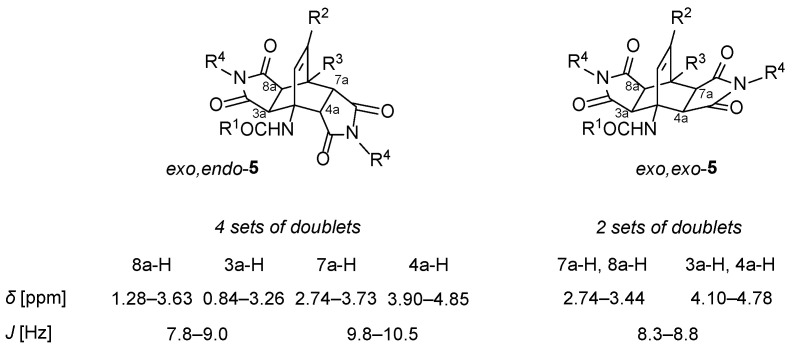
Comparison of the structural features of asymmetric and symmetric adducts **5**, highlighting the key differences reflected in the ^1^H NMR data.

To distinguish between the asymmetric and symmetric structures of **5**, NMR data were crucial (Figure 1). For *exo*,*endo*-**5**, four sets of doublets (each integrated as 1H) corresponding to the four aliphatic protons of the bicyclo[2.2.2]octene skeleton are observed. Two sets of doublets appear at *δ* 1.28–3.63 ppm (mean 2.55, range 2.35) and *δ* 0.84–3.26 ppm (mean 2.24, range 2.42), corresponding to the two protons (3a-H and 8a-H) being *anti* to the double bond. The other two doublets appear at *δ* 2.74–3.73 ppm (mean 3.10, range 0.99) and *δ* 3.90–4.85 ppm (mean 4.29, range 0.95), corresponding to the two protons (4a-H and 7a-H) being *syn* to the double bond. The coupling constant ranges are 7.8–9.0 Hz and 9.8–10.5 Hz for the pairs being *anti* and *syn* to the double bond, respectively. On the other hand, for symmetric *exo*,*exo*-**5**, only two doublets (each integrated as 2H) are observed at *δ* 2.74–3.44 ppm (mean 3.08, range 0.70) and *δ* 4.10–4.78 ppm (mean 4.40, range 0.68) in the ^1^H NMR spectra (coupling constants 8.3–8.8 Hz). All these data, corroborated by the ^13^C, ^1^H–^13^C *gs*-HSQC, and ^1^H–^13^C *gs*-HMBC 2D NMR spectra, clearly show the difference between the two structural types; furthermore, they are in agreement with previously published results for cyclooctane-fused asymmetric systems *exo*,*endo*-**5** (R^2^ – R^3^ = -[CH_2_]_6_-) [21]. Additionally, some of the *exo*,*endo* adducts were structurally characterized by single-crystal X-ray diffraction spectroscopy [43].

## 3. Materials and Methods

### 3.1. General

Melting points were determined on a micro hot stage apparatus and are uncorrected. NMR spectra were recorded with a Bruker (Zürich, Switzerland) Avance III 500 spectrometer at 29 °C, using TMS as the internal standard at 500 MHz for ^1^H NMR and 126 MHz for ^13^C NMR. Chemical shifts are provided as ppm values on the δ scale, and the coupling constants (*J*) are given in Hertz. ^13^C NMR spectra are referenced against the central line of the solvent signal (CDCl_3_ at 77.0 ppm and DMSO-*d*_6_ at 39.5 ppm). ^1^H NMR peak assignments are additionally based on analyses of ^1^H–^13^C *gs*-HSQC and ^1^H–^13^C *gs*-HMBC 2D NMR spectra. IR spectra of compounds as powders were obtained with a Bruker (Zürich, Switzerland) Alpha Platinum ATR FT-IR spectrophotometer. Mass spectra were recorded using an Agilent (Santa Clara, CA, USA) 6624 Accurate Mass TOF LC/MS spectrometer via ESI ionization. Elemental analyses were performed using a Perkin Elmer 2400 Series II CHNS/O analyzer (PerkinElmer, Inc., Waltham, MA, USA). TLC was carried out on silica gel TLC cards with a fluorescent indicator, and visualization was accomplished with UV light (254 nm). Reagents and solvents were used as received from commercial suppliers with a purity of 98% or higher. The xylene used was a commercially available mixture of all three isomers.

Microwave reactions were conducted in air using a focused microwave unit (Discover by CEM Corporation, Matthews, NC, USA). The instrument features a continuous, focused microwave power delivery system with operator-selectable power output from 0 to 300 W. Reactions were carried out in darkness in glass vessels (10 mL capacity) sealed with a septum. The pressure was controlled by a load cell connected to the vessel via the septum. The temperature of the vessel contents was monitored using a calibrated infrared temperature controller mounted beneath the reaction vessel, measuring the temperature of the vessel’s outer surface. The mixtures were stirred with a Teflon-coated magnetic stirring bar in the vessel. Temperature, pressure, and power profiles were recorded using commercially available software provided by the microwave unit manufacturer.

### 3.2. Synthesis and Characterization of Starting 2H-Pyran-2-Ones **1**

The starting compounds **1A**–**I** were prepared from appropriate compounds containing activated CH_2_ groups (such as 1,3-diketones, β-ketoesters, substituted acetones, etc.), a C_1_-synthon (DMFDMA, trimethyl or triethyl orthoformate, etc.) and hippuric acid, as described in [36]; compounds **2b**,**c**,**e** were prepared according to the procedures described previously [44,45]. The new 2*H*-pyran-2-one **1J** was synthesized by refluxing 1,2-diphenylethanone (5.89 g, 30 mmol) and DMFDMA (7.15 g, 60 mmol) for 4 h; thereafter, the volatile components were removed under reduced pressure. The crude residue was then reacted with hippuric acid (5.38 g, 30 mmol) in acetic anhydride (35 mL) at 90 °C for 4 h. After the removal of the volatile components, the residue was treated with ethanol (20 mL) and, after prolonged cooling (5 days), the precipitate was filtered off, washed with ethanol, and dried to afford the corresponding product **1J**.

3-Benzamido-5,6-diphenyl-2*H*-pyran-2-one (**1J**): yellow solid (8.69 g, 79%); mp 195–197 °C (EtOH); IR (ATR) ν_max_ 3392, 1698, 1677, 1634, 1513, 1482, 1379, 1249, 1152, 1071 cm^−1^; ^1^H NMR (CDCl_3_, 500 MHz) δ 8.78 (s, 1H, NH), 8.64 (s, 1H, CH), 7.90–7.94 (m, 2H, ArH), 7.57–7.62 (m, 1H, ArH), 7.50–7.55 (m, 2H, ArH), 7.27–7.38 (m, 8H, ArH), 7.22–7.26 (m, 2H, ArH); ^13^C NMR (CDCl_3_, 126 MHz) δ 166.0, 159.6, 150.9, 136.4, 133.5, 132.5, 131.8, 129.5, 129.4, 129.0, 128.94, 128.88, 128.3, 128.2, 128.0, 127.2, 123.7, 119.1; HRMS (ESI-TOF) *m*/*z* 368.1279 (calcd for C_24_H_18_NO_3_ (M + H)^+^ 368.1281); Anal. C, 78.35; H, 4.53; N, 3.85 (calcd for C_24_H_17_NO_3_ C, 78.46; H, 4.66; N, 3.81).


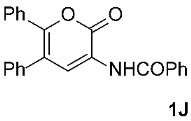


For the synthesis of **1K**, the mixture of 3-benzoylamino-2*H*-pyran-2-one derivative **1A** (3.35 g, 10 mmol), ethanol (250 mL), and concentrated aq. HCl (150 mL) was refluxed for 21 h. After cooling, the filtrate was neutralized with NaHCO_3_, and the volatile components were removed under reduced pressure. Water (200 mL) was added, and the mixture was extracted with CH_2_Cl_2_ (3 × 200 mL). The combined extracts were dried over Na_2_SO_4_ and concentrated in vacuo to give a solid residue. This was then dissolved in CH_2_Cl_2_ (40 mL) and left to react with acetyl chloride (735 μL, 10.3 mmol) and pyridine (840 μL, 10.4 mmol) for 4 h at room temperature. The volatile components were removed under reduced pressure, and water (20 mL) was added to the oily residue. The precipitated material was filtered off, washed with water and diethyl ether, and dried to yield the product **1K**.

3-Acetamido-5-(4-methoxyphenyl)-6-methyl-2*H*-pyran-2-one (**1K**): ochre solid (2.07 g, 76%); mp 179–181 °C (EtOH/CH_2_Cl_2_); IR (ATR) ν_max_ 3312, 1705, 1683, 1509, 1383, 1290, 1244, 1174, 1128, 1031 cm^−1^; ^1^H NMR (CDCl_3_, 500 MHz) δ 8.29 (s, 1H, CH), 7.94 (s, 1H, NH), 7.20 and 6.94 (AA’XX’, *J* = 8.5 Hz, 2H each, 4-OCH_3_-C_6_*H*_4_), 3.84 (s, 3H, OCH_3_), 2.25 (s, 3H, CH_3_), 2.20 (s, 3H, COCH_3_); ^13^C NMR (CDCl_3_, 126 MHz) δ 169.2, 159.8, 159.2, 151.4, 130.1, 128.2, 127.7, 122.7, 118.2, 114.0, 55.3, 24.6, 17.8; HRMS (ESI-TOF) *m*/*z* 274.1066 (calcd for C_15_H_16_NO_4_ (M + H)^+^ 274.1074); Anal. C, 66.04, H, 5.23; N, 5.18 (calcd for C_15_H_15_NO_4_ C, 65.92; H, 5.53; N, 5.13).


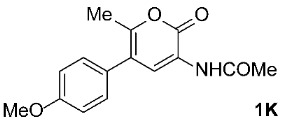


### 3.3. Synthesis of Bicyclo[2.2.2]Octenes exo,endo/exo,exo-**5** via Microwave Cycloaddition of 2H-Pyran-2-Ones **1** with Maleimides **2**

A mixture of 2*H*-pyran-2-one **1** (0.5 mmol), the corresponding *N*-substituted maleimide **2** (1.1 mmol), and toluene (2.2 mL) was irradiated in the focused microwave equipment for the time and at the temperature specified in Table 3. The power was set to 300 W with a ramp time of 5 min. After irradiation, the reaction mixture was cooled to room temperature, and the volatile components were removed under reduced pressure, yielding crude products **5**.

The products *exo*,*endo*-**5Ae** and *exo*,*endo*-**5Be** were purified by crystallization from EtOAc, yielding pure *exo*,*endo* adducts. In all cases of the synthesis of **5Ab**–**d** and **5Bb**–**d**, mixtures of both stereoisomers were separated by column chromatography (SiO_2_, petroleum ether : EtOAc = 10 : 1), yielding both types of adducts: *exo*,*endo*-**5Ab**–**d**,**Bb**–**d** and *exo*,*exo*-**5Ab**–**d**,**Bb**–**d**. Products *exo*,*endo*-**5C**–**Ke** were purified by column chromatography (SiO_2_, petroleum ether : EtOAc = 10 : 1). For the analyses, all products were recrystallized from the appropriate solvents. For ^1^H, ^13^C NMR and representative example of ^1^H–^13^C *gs*-HSQC and ^1^H–^13^C *gs*-HMBC 2D NMR NMR spectra of all new products **1** and **5**, see Supplementary Materials.

*rel*-*N*-((3a*R*,4*R*,4a*S*,7a*R*,8*S*,8a*S*)-9-(4-methoxyphenyl)-8-methyl-1,3,5,7-tetraoxo-2,3,3a,4a,5,6,7,7a,8,8a-decahydro-4,8-ethenopyrrolo[3,4-*f*]isoindol-4(1*H*)-yl)benzamide (*exo*,*exo*-**5Aa**): white solid (199 mg, 82%); mp 348–349 °C (EtOH); IR (ATR) ν_max_ 3364, 3153, 3063, 1697, 1648, 1543, 1510, 1346, 1307, 1207, 1181 cm^−1^; ^1^H NMR (DMSO-*d*_6_, 500 MHz) δ 11.26 (s, 2H, 2 × NH), 8.61 (s, 1H, 4-NH), 7.87–7.90 (m, 2H, ArH), 7.53–7.57 (m, 1H, ArH), 7.48–7.52 (m, 2H, ArH), 6.92 and 6.87 (AA’XX’, *J* = 8.5 Hz, 2H each, 4-OCH_3_-C_6_*H*_4_), 6.30 (s, 1H, C=CH), 4.23 (d, *J* = 8.5 Hz, 2H, 3a-CH, 4a-CH), 3.75 (s, 3H, OCH_3_), 3.08 (d, *J* = 8.5 Hz, 2H, 7a-CH, 8a-CH), 1.66 (s, 3H, CH_3_); ^13^C NMR (DMSO-*d*_6_, 126 MHz) δ 178.0, 176.4, 167.6, 158.8, 145.2, 135.7, 131.0, 130.0, 128.9, 128.0, 127.7, 127.3, 113.6, 57.8, 55.1, 50.2, 44.6, 42.1, 18.6; HRMS (ESI-TOF) *m*/*z* 486.1659 (calcd for C_27_H_24_N_3_O_6_ (M + H)^+^ 486.1660); Anal. C, 66.17; H, 4.53; N, 8.35 (calcd for C_27_H_23_N_3_O_6_ · 1/4 H_2_O C, 66.18; H, 4.83; N, 8.58).


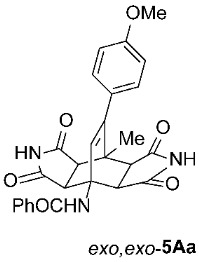


*rel*-*N*-((3a*R*,4*R*,4a*R*,7a*S*,8*S*,8a*S*)-2,6-Bis(4-(*tert*-butyl)phenyl)-9-(4-methoxyphenyl)-8-methyl-1,3,5,7-tetraoxo-2,3,3a,4a,5,6,7,7a,8,8a-decahydro-4,8-ethenopyrrolo[3,4-*f*]isoindol-4(1*H*)-yl)benzamide (*exo*,*endo*-**5Ab**): white solid (64 mg, 17%); *R*_f_ = 0.58 (PE/EtOAc 1/1); mp 247–249 °C (EtOH); IR (ATR) ν_max_ 2957, 2916, 2848, 1709, 1655, 1509, 1374, 1246, 1183 cm^−1^; ^1^H NMR (DMSO-*d*_6_, 500 MHz) δ 8.67 (s, 1H, NH), 7.87–7.91 (m, 2H, ArH), 7.49–7.60 (m, 7H, ArH), 7.30 and 7.11 (AA’XX’, *J* = 8.5 Hz, 2H each, ArH), 7.09 and 6.93 (AA’XX’, *J* = 9.0 Hz, 2H each, ArH), 6.39 (s, 1H, C=CH), 4.26 (d, *J* = 9.8 Hz, 1H, 4a-CH), 3.76 (s, 3H, OCH_3_), 3.63 (d, *J* = 8.3 Hz, 1H, 3a-CH), 3.37 (d, *J* = 9.8 Hz, 1H, 7a-CH), 3.24 (d, *J* = 8.3 Hz, 1H, 8a-CH), 1.69 (s, 3H, CH_3_), 1.32 (s, 9H, C(CH_3_)_3_), 1.30 (s, 9H, C(CH_3_)_3_); ^13^C NMR (DMSO-*d*_6_, 126 MHz) δ 175.6, 175.3, 175.2, 174.0, 166.6, 158.8, 151.2, 145.7, 134.8, 132.3, 131.6, 129.8, 129.54, 129.48, 129.2, 128.4, 127.3, 126.9, 126.2, 126.0, 125.6, 113.6, 57.3, 51.1, 50.9, 45.9, 45.5, 43.7, 41.5, 34.5, 31.1, 31.0, 19.3; HRMS (ESI-TOF) *m*/*z* 750.3525 (calcd for C_47_H_48_N_3_O_6_ (M + H)^+^ 750.3538); Anal. C, 73.30; H, 6.59; N, 5.53 (calcd for C_47_H_47_N_3_O_6_ · H_2_O C, 73.51; H, 6.43; N, 5.47).


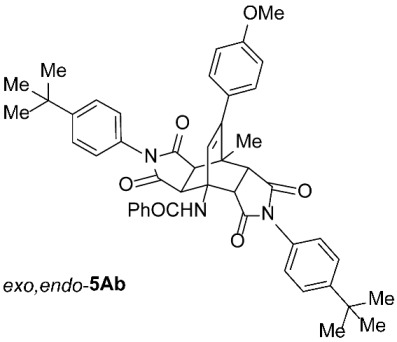


*rel*-*N*-((3a*R*,4*R*,4a*S*,7a*R*,8*S*,8a*S*)-2,6-Bis(4-(*tert*-butyl)phenyl)-9-(4-methoxyphenyl)-8-methyl-1,3,5,7-tetraoxo-2,3,3a,4a,5,6,7,7a,8,8a-decahydro-4,8-ethenopyrrolo[3,4-*f*]isoindol-4(1*H*)-yl)benzamide (*exo*,*exo*-**5Ab**): white solid (243 mg, 65%); *R*_f_ = 0.35 (PE/EtOAc 1/1); mp 233–235 °C (EtOH); IR (ATR) ν_max_ 3312, 2956, 1709, 1643, 1551, 1511, 1374, 1193, 1179, 1159 cm^−1^; ^1^H NMR (DMSO-*d*_6_, 500 MHz) δ 8.77 (s, 1H, NH), 7.82–7.86 (m, 2H, ArH), 7.45–7.56 (m, 7H, ArH), 7.03 (AA’BB’, *J* = 9.0 Hz, 4H, ArH), 6.93–6.98 (m, 4H, ArH), 6.45 (s, 1H, C=CH), 4.54 (d, *J* = 8.5 Hz, 2H, 3a-CH, 4a-CH), 3.76 (s, 3H, OCH_3_), 3.44 (d, *J* = 8.5 Hz, 2H, 7a-CH, 8a-CH), 1.82 (s, 3H, CH_3_), 1.28 (s, 18H, 2 × C(CH_3_)_3_); ^13^C NMR (DMSO-*d*_6_, 126 MHz) δ 175.7, 174.1, 168.2, 159.0, 151.0, 145.3, 135.8, 131.0, 129.7, 129.5, 128.8, 128.0, 127.6, 127.3, 126.4, 125.8, 113.9, 58.3, 55.2, 49.2, 43.9, 42.7, 34.5, 31.0, 18.9; HRMS (ESI-TOF) *m*/*z* 750.3533 (calcd for C_47_H_48_N_3_O_6_ (M + H)^+^ 750.3538); Anal. C, 74.82; H, 6.04; N, 5.67 (calcd for C_47_H_47_N_3_O_6_ · 1/5 H_2_O C, 74.92; H, 6.34; N, 5.58).


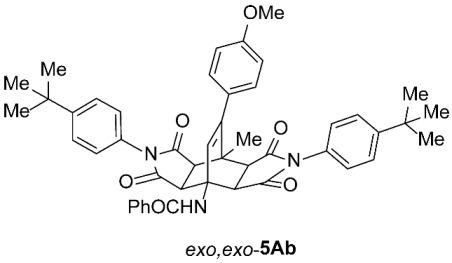


*rel*-*N*-((3a*R*,4*R*,4a*R*,7a*S*,8*S*,8a*S*)-2,6-Bis(2-(*tert*-butyl)phenyl)-9-(4-methoxyphenyl)-8-methyl-1,3,5,7-tetraoxo-2,3,3a,4a,5,6,7,7a,8,8a-decahydro-4,8-ethenopyrrolo[3,4-*f*]isoindol-4(1*H*)-yl)benzamide (*exo*,*endo*-**5Ac**): white solid (86 mg, 23%); *R*_f_ = 0.60 (PE/EtOAc 1/1); mp 341–342 °C (EtOH); IR (ATR) ν_max_ 2964, 1713, 1655, 1522, 1509, 1489, 1372, 1196, 1176 cm^−1^; ^1^H NMR (CDCl_3_, 500 MHz) δ 8.18 (s, 1H, NH), 7.89–7.92 (m, 2H, ArH), 7.61 (td, *J* = 8.5, 1.5 Hz, 2H, ArH), 7.46–7.50 (m, 1H, ArH), 7.37–7.45 (m, 4H, ArH), 7.33 (td, *J* = 7.5, 1.5 Hz, 1H, ArH), 7.19 (td, *J* = 7.5, 1.5 Hz, 1H, ArH), 7.14 and 6.86 (AA’XX’, *J* = 8.8 Hz, 2H each, 4-OCH_3_-C_6_*H*_4_), 6.70 (dd, *J* = 8.0, 1.3 Hz, 1H, ArH), 6.57 (dd, *J* = 8.0, 1.5 Hz, 1H, ArH), 6.54 (s, 1H, C=CH), 4.83 (d, *J* = 10.3 Hz, 1H, 4a-CH), 3.82 (s, 3H, OCH_3_), 3.39 (d, *J* = 8.5 Hz, 1H, 3a-CH), 3.26 (d, *J* = 8.5 Hz, 1H, 8a-CH), 3.14 (d, *J* = 10.3 Hz, 1H, 7a-CH), 1.95 (s, 3H, CH_3_), 1.35 (s, 9H, C(CH_3_)_3_), 1.29 (s, 9H, C(CH_3_)_3_); ^13^C NMR (CDCl_3_, 126 MHz) δ 176.5, 176.3, 175.9, 175.0, 167.7, 159.5, 148.4, 148.2, 147.8, 134.1, 132.5, 131.9, 130.4, 130.3, 130.1, 129.91, 129.89, 129.7, 129.5, 129.4, 129.1, 128.9, 128.6, 127.7, 127.4, 127.2, 113.7, 57.9, 55.3, 51.1, 45.7, 45.4, 43.1, 42.8, 35.9, 35.8, 31.8, 31.7, 18.6; HRMS (ESI-TOF) *m*/*z* 750.3536 (calcd for C_47_H_48_N_3_O_6_ (M + H)^+^ 750.3538); Anal. C, 75.10; H, 6.45; N, 5.62 (calcd for C_47_H_47_N_3_O_6_ C, 75.28; H, 6.32; N, 5.60).


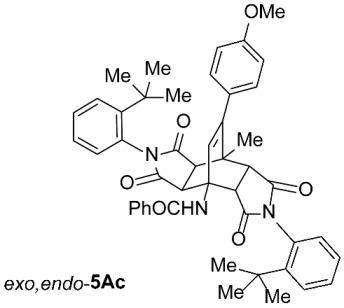


*rel*-*N*-((3a*R*,4*R*,4a*S*,7a*R*,8*S*,8a*S*)-2,6-Bis(2-(*tert*-butyl)phenyl)-9-(4-methoxyphenyl)-8-methyl-1,3,5,7-tetraoxo-2,3,3a,4a,5,6,7,7a,8,8a-decahydro-4,8-ethenopyrrolo[3,4-*f*]isoindol-4(1*H*)-yl)benzamide (*exo*,*exo*-**5Ac**): white solid (142 mg, 38%); *R*_f_ = 0.42 (PE/EtOAc 1/1); mp 338–340 °C (EtOAc); IR (ATR) ν_max_ 2960, 1711, 1510, 1488, 1440, 1369, 1285, 1248, 1201, 1176, 1155 cm^−1^; ^1^H NMR (CDCl_3_, 500 MHz) δ 7.85–7.89 (m, 2H, ArH), 7.54–7.58 (m, 2H, ArH), 7.44–7.48 (m, 1H, ArH), 7.33–7.42 (m, 4H, ArH), 7.18–7.23 (m, 2H, ArH), 7.13 and 6.86 (AA’XX’, *J* = 8.8 Hz, 2H each, 4-OCH_3_-C_6_*H*_4_), 6.61–6.67 (m, 3H, ArH, NH), 6.11 (s, 1H, C=CH), 4.77 (d, *J* = 8.5 Hz, 2H, 3a-CH, 4a-CH), 3.81 (s, 3H, OCH_3_), 3.18 (d, *J* = 8.5 Hz, 2H, 7a-CH, 8a-CH), 2.08 (s, 3H, CH_3_), 1.28 (s, 18H, 2 × C(CH_3_)_3_); ^13^C NMR (CDCl_3_, 126 MHz) δ 175.7, 174.7, 169.7, 159.8, 148.4, 147.9, 135.1, 131.6, 130.4, 129.9, 129.7, 129.2, 129.1, 128.6, 127.3, 127.1, 126.9, 114.0, 58.2, 55.3, 50.1, 44.0, 43.8, 35.8, 31.7, 19.4 (1 signal hidden); HRMS (ESI-TOF) *m*/*z* 750.3531 (calcd for C_47_H_48_N_3_O_6_ (M + H)^+^ 750.3538); Anal. C, 74.00; H, 6.48; N, 5.48 (calcd for C_47_H_47_N_3_O_6_ · 2/3 H_2_O C, 74.09; H, 6.39; N, 5.52).


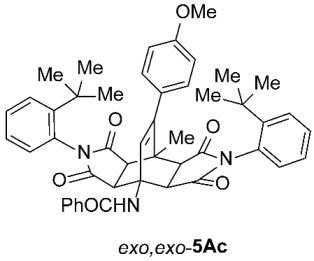


*rel*-*N*-((3a*R*,4*R*,4a*R*,7a*S*,8*S*,8a*S*)-2,6-Di-*tert*-butyl-9-(4-methoxyphenyl)-8-methyl-1,3,5,7-tetraoxo-2,3,3a,4a,5,6,7,7a,8,8a-decahydro-4,8-ethenopyrrolo[3,4-*f*]isoindol-4(1*H*)-yl)benzamide (*exo*,*endo*-**5Ad**): white solid (116 mg, 39%); *R*_f_ = 0.61 (PE/EtOAc 1/1); mp 267–268 °C (EtOH/Me_2_CO); IR (ATR) ν_max_ 3390, 2975, 1765, 1690, 1667, 1528, 1509, 1339, 1263, 1174 cm^−1^; ^1^H NMR (DMSO-*d*_6_, 500 MHz) δ 8.41 (s, 1H, NH), 7.86–7.90 (m, 2H, ArH), 7.54–7.64 (m, 3H, ArH), 7.03 and 6.91 (AA’XX’, *J* = 9.0 Hz, 2H each, 4-OCH_3_-C_6_*H*_4_), 6.13 (s, 1H, C=CH), 3.95 (d, *J* = 10.0 Hz, 1H, 4a-CH), 3.75 (s, 3H, OCH_3_), 3.08 (d, *J* = 8.3 Hz, 1H, 3a-CH), 2.92 (d, *J* = 10.0 Hz, 1H, 7a-CH), 2.74 (d, *J* = 8.3 Hz, 1H, 8a-CH), 1.58 (s, 3H, CH_3_), 1.53 (s, 9H, C(CH_3_)_3_), 1.46 (s, 9H, C(CH_3_)_3_); ^13^C NMR (DMSO-*d*_6_, 126 MHz) δ 177.4, 177.1, 176.8, 175.7, 166.1, 158.7, 145.8, 134.7, 131.9, 131.7, 129.8, 129.4, 128.6, 127.1, 113.4, 57.8, 57.6, 57.2, 55.1, 50.3, 45.0, 44.6, 42.8, 41.4, 28.0, 27.8, 19.4; HRMS (ESI-TOF) *m*/*z* 598.2908 (calcd for C_35_H_40_N_3_O_6_ (M + H)^+^ 598.2912); Anal. C, 69.71; H, 6.50; N, 6.99 (calcd for C_35_H_39_N_3_O_6_ · 1/4 H_2_O C, 69.81; H, 6.61; N, 6.98).


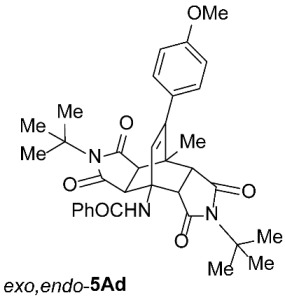


*rel*-*N*-((3a*R*,4*R*,4a*S*,7a*R*,8*S*,8a*S*)-2,6-Di-*tert*-butyl-9-(4-methoxyphenyl)-8-methyl-1,3,5,7-tetraoxo-2,3,3a,4a,5,6,7,7a,8,8a-decahydro-4,8-ethenopyrrolo[3,4-*f*]isoindol-4(1*H*)-yl)benzamide (*exo*,*exo*-**5Ad**): white solid (128 mg, 43%); *R*_f_ = 0.42 (PE/EtOAc 1/1); mp 307–308 °C (EtOH/H_2_O); IR (ATR) ν_max_ 3310, 2985, 2937, 1762, 1699, 1636, 1549, 1512, 1334, 1252, 1178, 1152 cm^−1^; ^1^H NMR (DMSO-*d*_6_, 500 MHz) δ 8.61 (s, 1H, NH), 7.86–7.91 (m, 2H, ArH), 7.50–7.59 (m, 3H, ArH), 6.91–6.97 (AA’BB’, *J* = 9.0 Hz, 4H, 4-OCH_3_-C_6_*H*_4_), 6.28 (s, 1H, C=CH), 4.14 (d, *J* = 8.5 Hz, 2H, 3a-CH, 4a-CH), 3.75 (s, 3H, OCH_3_), 2.97 (d, *J* = 8.5 Hz, 2H, 7a-CH, 8a-CH), 1.71 (s, 3H, CH_3_), 1.40 (s, 18H, 2 × C(CH_3_)_3_); ^13^C NMR (DMSO-*d*_6_, 126 MHz) δ 177.1, 175.6, 167.7, 158.8, 144.4, 135.9, 131.0, 129.8, 128.8, 128.1, 127.7, 126.9, 113.6, 58.3, 57.1, 55.1, 48.7, 42.8, 42.4, 28.1, 19.0; HRMS (ESI-TOF) *m*/*z* 598.2908 (calcd for C_35_H_40_N_3_O_6_ (M + H)^+^ 598.2912); Anal. C, 70.55; H, 6.43; N, 7.06 (calcd for C_35_H_39_N_3_O_6_ C, 70.33; H, 6.58; N, 7.03).


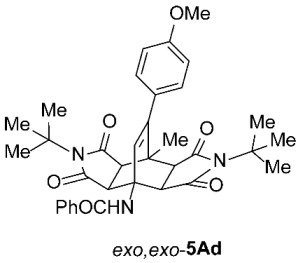


*rel*-*N*-((3a*R*,4*R*,4a*R*,7a*S*,8*S*,8a*S*)-9-(4-methoxyphenyl)-8-methyl-1,3,5,7-tetraoxo-2,6-ditrityl-2,3,3a,4a,5,6,7,7a,8,8a-decahydro-4,8-ethenopyrrolo[3,4-*f*]isoindol-4(1*H*)-yl)benzamide (*exo*,*endo*-**5Ae**): white solid (422 mg, 87%); mp 276–277 °C (EtOH); IR (ATR) ν_max_ 3058, 1713, 1704, 1648, 1510, 1450, 1322, 1249, 1154, 1030 cm^−1^; ^1^H NMR (CDCl_3_, 500 MHz) δ 7.72–7.75 (m, 2H, ArH), 7.61 (s, 1H, NH), 7.36–7.49 (m, 9H, ArH), 7.14–7.24 (m, 21H, ArH), 7.06–7.11 (m, 3H, ArH), 6.87 and 6.77 (AA’XX’, *J* = 8.5 Hz, 2H each, 4-OCH_3_-C_6_*H*_4_), 6.18 (s, 1H, C=CH), 4.45 (d, *J* = 10.3 Hz, 1H, 4a-CH), 3.80 (s, 3H, OCH_3_), 2.88 (d, *J* = 10.3 Hz, 1H, 7a-CH), 1.82 (d, *J* = 9.0 Hz, 1H, 8a-CH), 1.67 (s, 3H, CH_3_), 1.46 (d, *J* = 9.0 Hz, 1H, 3a-CH); ^13^C NMR (CDCl_3_, 126 MHz) δ 175.7, 174.94, 174.92, 174.4, 167.4, 159.3, 148.2, 142.0, 141.5, 134.1, 131.80, 131.79, 130.4, 129.0, 128.6, 128.2, 128.0, 127.63, 127.56, 127.3, 126.74, 126.73, 113.2, 75.2, 74.3, 57.8, 55.3, 49.7, 45.5, 45.0, 42.5, 41.9, 18.9; HRMS (ESI-TOF) *m*/*z* 970.3878 (calcd for C_65_H_52_N_3_O_6_ (M + H)^+^ 970.3856); Anal. C, 79.92; H, 5.25; N, 4.32 (calcd for C_65_H_51_N_3_O_6_ · 1/4 H_2_O C, 80.10; H, 5.33; N, 4.31).


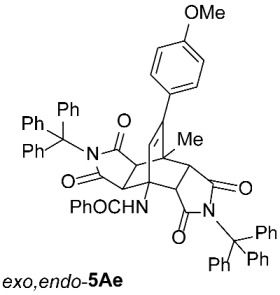


*rel*-*N*-((3a*R*,4*R*,4a*S*,7a*R*,8*S*,8a*S*)-9-(3,4-dimethoxyphenyl)-8-methyl-1,3,5,7-tetraoxo-2,3,3a,4a,5,6,7,7a,8,8a-decahydro-4,8-ethenopyrrolo[3,4-*f*]isoindol-4(1*H*)-yl)benzamide (*exo*,*exo*-**5Ba**): white solid (219 mg, 82%); mp 349–350 °C (MeOH); IR (ATR) ν_max_ 3221, 1703, 1671, 1514, 1346, 1302, 1253, 1202, 1153, 1136 cm^−1^; ^1^H NMR (DMSO-*d*_6_, 500 MHz) δ 11.28 (s, 2H, 2 × NH), 8.62 (s, 1H, 4-NH), 7.87–7.91 (m, 2H, ArH), 7.54–7.58 (m, 1H, ArH), 7.48–7.53 (m, 2H, ArH), 6.93 (d, *J* = 8.2 Hz, 1H, 3,4-(OCH_3_)_2_-C_6_*H*_3_), 6.51 (d, *J* = 2.0 Hz, 1H, 3,4-(OCH_3_)_2_-C_6_*H*_3_), 6.48 (dd, *J* = 8.2, 2.0 Hz, 1H, 3,4-(OCH_3_)_2_-C_6_*H*_3_), 6.32 (s, 1H, C=CH), 4.23 (d, *J* = 8.3 Hz, 2H, 3a-CH, 4a-CH), 3.75 (s, 3H, OCH_3_), 3.70 (s, 3H, OCH_3_), 3.08 (d, *J* = 8.3 Hz, 2H, 7a-CH, 8a-CH), 1.67 (s, 3H, CH_3_); ^13^C NMR (DMSO-*d*_6_, 126 MHz) δ 178.1, 176.4, 167.7, 148.4, 147.9, 145.3, 135.7, 131.0, 130.2, 128.1, 127.7, 127.2, 120.2, 111.6, 111.5, 57.8, 55.5, 55.4, 50.2, 44.6, 42.2, 18.6; HRMS (ESI-TOF) *m*/*z* 516.1768 (calcd for C_28_H_26_N_3_O_7_ (M + H)^+^ 516.1765); Anal. C, 64.49; H, 4.80; N, 7.96 (calcd for C_28_H_25_N_3_O_7_ · 1/3 H_2_O C, 64.49; H, 4.96; N, 8.06).


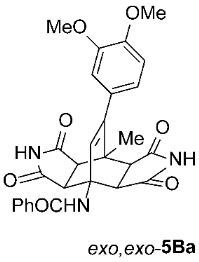


*rel*-*N*-((3a*R*,4*R*,4a*R*,7a*S*,8*S*,8a*S*)-2,6-Bis(4-(*tert*-butyl)phenyl)-9-(3,4-dimethoxyphenyl)-8-methyl-1,3,5,7-tetraoxo-2,3,3a,4a,5,6,7,7a,8,8a-decahydro-4,8-ethenopyrrolo[3,4-*f*]isoindol-4(1*H*)-yl)benzamide (*exo*,*endo*-**5Bb**): white solid (43 mg, 11%); *R*_f_ = 0.42 (PE/EtOAc 1/1); mp 194–196 °C (EtOAc); IR (ATR) ν_max_ 2960, 1710, 1664, 1514, 1488, 1381, 1310, 1255, 1189, 1175, 1138, 1024 cm^−1^; ^1^H NMR (CDCl_3_, 500 MHz) δ 8.10 (s, 1H, NH), 7.90–7.93 (m, 2H, ArH), 7.41–7.52 (m, 7H, ArH), 7.22 and 7.11 (AA’XX’, *J* = 9.0 Hz, 2H each, 4-C(CH_3_)_3_-C_6_*H*_4_), 6.82 (d, *J* = 8.2 Hz, 1H, 3,4-(OCH_3_)_2_-C_6_*H*_3_), 6.73 (dd, *J* = 8.2, 2.0 Hz, 1H, 3,4-(OCH_3_)_2_-C_6_*H*_3_), 6.60 (d, *J* = 2.0 Hz, 1H, 3,4-(OCH_3_)_2_-C_6_*H*_3_), 6.47 (s, 1H, C=CH), 4.84 (d, *J* = 10.0 Hz, 1H, 4a-CH), 3.89 (s, 3H, OCH_3_), 3.74 (s, 3H, OCH_3_), 3.35 (d, *J* = 8.3 Hz, 1H, 3a-CH), 3.21 (d, *J* = 8.3 Hz, 1H, 8a-CH), 3.17 (d, *J* = 10.0 Hz, 1H, 7a-CH), 1.93 (s, 3H, CH_3_), 1.33 (s, 9H, C(CH_3_)_3_), 1.31 (s, 9H, C(CH_3_)_3_); ^13^C NMR (CDCl_3_, 126 MHz) δ 175.5, 174.9, 174.7, 174.1, 167.7, 152.6, 152.2, 148.9, 148.4, 147.8, 134.1, 132.2, 131.9, 129.3, 128.6, 128.5, 128.2, 127.1, 126.4, 126.3, 125.7, 125.6, 121.1, 111.4, 110.8, 57.9, 55.9, 55.8, 51.0, 45.3, 45.1, 43.2, 42.9, 34.79, 34.78, 31.21, 31.17, 18.9; HRMS (ESI-TOF) *m*/*z* 780.3647 (calcd for C_48_H_50_N_3_O_7_ (M + H)^+^ 780.3643); Anal. C, 73.32; H, 6.24; N, 5.33 (calcd for C_48_H_49_N_3_O_7_ · 1/3 H_2_O C, 73.36; H, 6.37; N, 5.35).


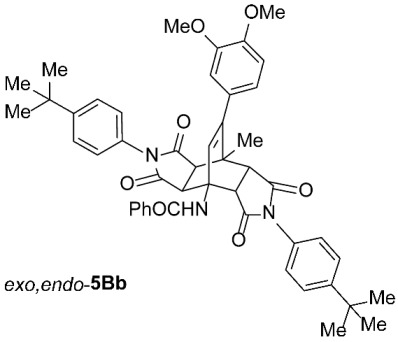


*rel*-*N*-((3a*R*,4*R*,4a*S*,7a*R*,8*S*,8a*S*)-2,6-Bis(4-(*tert*-butyl)phenyl)-9-(3,4-dimethoxyphenyl)-8-methyl-1,3,5,7-tetraoxo-2,3,3a,4a,5,6,7,7a,8,8a-decahydro-4,8-ethenopyrrolo[3,4-*f*]isoindol-4(1*H*)-yl)benzamide (*exo*,*exo*-**5Bb**): white solid (273 mg, 70%); *R*_f_ = 0.23 (PE/EtOAc 1/1); mp 323–325 °C (EtOAc); IR (ATR) ν_max_ 3313, 2958, 1709, 1636, 1558, 1514, 1377, 1247, 1203, 1187, 1160, 1142 cm^−1^; ^1^H NMR (CDCl_3_, 500 MHz) δ 7.84–7.88 (m, 2H, ArH), 7.36–7.49 (m, 7H, ArH), 7.06–7.11 (m, 4H, ArH), 6.80 (d, *J* = 8.3 Hz, 1H, 3,4-(OCH_3_)_2_-C_6_*H*_3_), 6.74 (s, 1H, NH), 6.63 (dd, *J* = 8.3, 2.0 Hz, 1H, 3,4-(OCH_3_)_2_-C_6_*H*_3_), 6.54 (d, *J* = 2.0 Hz, 1H, 3,4-(OCH_3_)_2_-C_6_*H*_3_), 6.00 (s, 1H, C=CH), 4.72 (d, *J* = 8.5 Hz, 2H, 3a-CH, 4a-CH), 3.87 (s, 3H, OCH_3_), 3.63 (s, 3H, OCH_3_), 3.13 (d, *J* = 8.5 Hz, 2H, 7a-CH, 8a-CH), 2.00 (s, 3H, CH_3_), 1.29 (s, 18H, 2 × C(CH_3_)_3_); ^13^C NMR (CDCl_3_, 126 MHz) δ 174.6, 173.9, 169.5, 152.0, 149.2, 148.6, 147.7, 135.1, 131.6, 129.0, 128.61, 128.58, 127.1, 126.8, 126.2, 125.7, 120.4, 111.0, 58.3, 55.9, 55.7, 49.8, 44.0, 43.7, 34.7, 31.2, 19.3 (1 signal hidden); HRMS (ESI-TOF) *m*/*z* 780.3636 (calcd for C_48_H_50_N_3_O_7_ (M + H)^+^ 780.3643); Anal. C, 73.36; H, 6.33; N, 5.36 (calcd for C_48_H_49_N_3_O_7_ · 1/3 H_2_O C, 73.36; H, 6.37; N, 5.35).


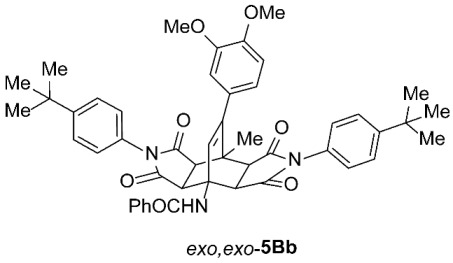


*rel*-*N*-((3a*R*,4*R*,4a*R*,7a*S*,8*S*,8a*S*)-2,6-Bis(2-(*tert*-butyl)phenyl)-9-(3,4-dimethoxyphenyl)-8-methyl-1,3,5,7-tetraoxo-2,3,3a,4a,5,6,7,7a,8,8a-decahydro-4,8-ethenopyrrolo[3,4-*f*]isoindol-4(1*H*)-yl)benzamide (*exo*,*endo*-**5Bc**): white solid (74 mg, 19%); *R*_f_ = 0.58 (PE/EtOAc 1/1); mp 353–355 °C (EtOAc); IR (ATR) ν_max_ 3419, 2960, 1714, 1658, 1513, 1490, 1441, 1372, 1251, 1196, 1137, 1028 cm^−1^; ^1^H NMR (CDCl_3_, 500 MHz) δ 8.19 (s, 1H, NH), 7.89–7.92 (m, 2H, ArH), 7.61 (td, *J* = 8.3, 1.0 Hz, 2H, ArH), 7.37–7.50 (m, 5H, ArH), 7.34 (td, *J* = 7.5, 1.3 Hz, 1H, ArH), 7.18 (td, *J* = 7.6, 1.2 Hz, 1H, ArH), 6.83 (d, *J* = 8.0 Hz, 1H, ArH), 6.78 (dd, *J* = 8.3, 1.8 Hz, 1H, ArH), 6.68–6.72 (m, 2H, ArH), 6.61 (dd, *J* = 7.8, 1.3 Hz, 1H, ArH), 6.55 (s, 1H, C=CH), 4.85 (d, *J* = 10.3 Hz, 1H, 4a-CH), 3.90 (s, 3H, OCH_3_), 3.78 (s, 3H, OCH_3_), 3.40 (d, *J* = 8.5 Hz, 1H, 3a-CH), 3.26 (d, *J* = 8.5 Hz, 1H, 8a-CH), 3.15 (d, *J* = 10.3 Hz, 1H, 7a-CH), 1.95 (s, 3H, CH_3_), 1.35 (s, 9H, C(CH_3_)_3_), 1.29 (s, 9H, C(CH_3_)_3_); ^13^C NMR (CDCl_3_, 126 MHz) δ 176.4, 176.3, 175.9, 175.0, 167.8, 148.9, 148.43, 148.39, 148.2, 147.9, 134.1, 132.7, 131.9, 130.4, 130.3, 130.1, 129.9, 129.7, 129.5, 129.4, 129.2, 128.6, 127.5, 127.4, 127.2, 121.3, 111.4, 110.8, 57.9, 55.91, 55.85, 51.1, 45.5, 45.4, 43.1, 42.8, 35.9, 35.8, 31.74, 31.68, 18.6 (1 signal hidden); HRMS (ESI-TOF) *m*/*z* 780.3650 (calcd for C_48_H_50_N_3_O_7_ (M + H)^+^ 780.3643); Anal. C, 73.50; H, 6.28; N, 5.33 (calcd for C_48_H_49_N_3_O_7_ · 1/4 H_2_O C, 73.50; H, 6.36; N, 5.36).


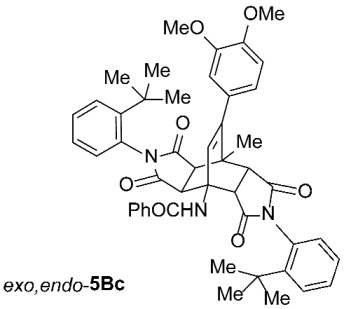


*rel*-*N*-((3a*R*,4*R*,4a*S*,7a*R*,8*S*,8a*S*)-2,6-Bis(2-(*tert*-butyl)phenyl)-9-(3,4-dimethoxyphenyl)-8-methyl-1,3,5,7-tetraoxo-2,3,3a,4a,5,6,7,7a,8,8a-decahydro-4,8-ethenopyrrolo[3,4-*f*]isoindol-4(1*H*)-yl)benzamide (*exo*,*exo*-**5Bc**): white solid (191 mg, 49%); *R*_f_ = 0.44 (PE/EtOAc 1/1); mp 336–338 °C (EtOAc); IR (ATR) ν_max_ 2959, 1713, 1646, 1514, 1490, 1369, 1247, 1195, 1140, 1023 cm^−1^; ^1^H NMR (CDCl_3_, 500 MHz) δ 7.85–7.89 (m, 2H, ArH), 7.57 (dd, *J* = 8.0, 1.0 Hz, 2H, ArH), 7.44–7.48 (m, 1H, ArH), 7.34–7.41 (m, 4H, ArH), 7.18 (td, *J* = 7.5, 0.8 Hz, 2H, ArH), 6.82 (d, *J* = 8.5 Hz, 1H, ArH), 6.72 (dd, *J* = 8.5, 2.0 Hz, 1H, ArH), 6.65–6.70 (m, 4H, ArH, NH), 6.12 (s, 1H, C=CH), 4.78 (d, *J* = 8.8 Hz, 2H, 3a-CH, 4a-CH), 3.88 (s, 3H, OCH_3_), 3.74 (s, 3H, OCH_3_), 3.19 (d, *J* = 8.8 Hz, 2H, 7a-CH, 8a-CH), 2.08 (s, 3H, CH_3_), 1.28 (s, 18H, 2 × C(CH_3_)_3_); ^13^C NMR (CDCl_3_, 126 MHz) δ 175.7, 174.6, 169.7, 149.2, 148.63, 148.60, 148.0, 135.1, 131.6, 130.4, 129.9, 129.7, 129.3, 128.9, 128.6, 127.1, 127.0, 120.3, 111.0, 110.8, 58.3, 55.9, 55.8, 50.1, 44.0, 43.7, 35.8, 31.7, 19.4 (1 signal hidden); HRMS (ESI-TOF) *m*/*z* 780.3649 (calcd for C_48_H_50_N_3_O_7_ (M + H)^+^ 780.3643); Anal. C, 73.11; H, 6.39; N, 5.31 (calcd for C_48_H_49_N_3_O_7_ · 1/2 H_2_O C, 73.08; H, 6.39; N, 5.33).


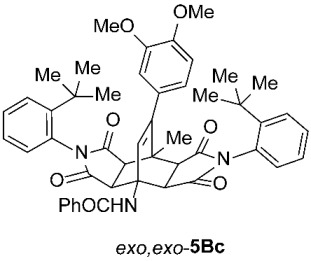


*rel*-*N*-((3a*R*,4*R*,4a*R*,7a*S*,8*S*,8a*S*)-2,6-Di-*tert*-butyl-9-(3,4-dimethoxyphenyl)-8-methyl-1,3,5,7-tetraoxo-2,3,3a,4a,5,6,7,7a,8,8a-decahydro-4,8-ethenopyrrolo[3,4-*f*]isoindol-4(1*H*)-yl)benzamide (*exo*,*endo*-**5Bd**): white solid (119 mg, 38%); *R*_f_ = 0.44 (PE/EtOAc 1/1); mp 278–280 °C (EtOH); IR (ATR) ν_max_ 3401, 2963, 2936, 1701, 1688, 1660, 1510, 1335, 1260, 1168, 1135 cm^−1^; ^1^H NMR (CDCl_3_, 500 MHz) δ 8.16 (s, 1H, NH), 7.93–7.96 (m, 2H, ArH), 7.47–7.56 (m, 3H, ArH), 6.79 (d, *J* = 8.3 Hz, 1H, 3,4-(OCH_3_)_2_-C_6_*H*_3_), 6.68 (dd, *J* = 8.3, 2.0 Hz, 1H, 3,4-(OCH_3_)_2_-C_6_*H*_3_), 6.64 (d, *J* = 2.0 Hz, 1H, 3,4-(OCH_3_)_2_-C_6_*H*_3_), 6.28 (s, 1H, C=CH), 4.42 (d, *J* = 10.3 Hz, 1H, 4a-CH), 3.88 (s, 3H, OCH_3_), 3.87 (s, 3H, OCH_3_), 2.88 (d, *J* = 8.8 Hz, 1H, 3a-CH), 2.74 (d, *J* = 10.3 Hz, 1H, 7a-CH), 2.72 (d, *J* = 8.8 Hz, 1H, 8a-CH), 1.80 (s, 3H, CH_3_), 1.59 (s, 9H, C(CH_3_)_3_), 1.55 (s, 9H, C(CH_3_)_3_); ^13^C NMR (CDCl_3_, 126 MHz) δ 177.5, 177.2, 176.6, 175.9, 167.3, 148.8, 148.3, 147.4, 134.4, 131.9, 131.8, 129.6, 128.7, 127.2, 121.3, 111.6, 110.6, 59.5, 58.9, 57.7, 55.9, 55.8, 50.6, 44.64, 44.60, 42.5, 42.4, 28.40, 28.36, 19.0; HRMS (ESI-TOF) *m*/*z* 626.2893 (calcd for C_36_H_40_N_3_O_7_ (M − H)^−^ 626.2872); Anal. C, 69.13; H, 6.58; N, 6.63 (calcd for C_36_H_41_N_3_O_7_ C, 68.88; H, 6.58; N, 6.69).


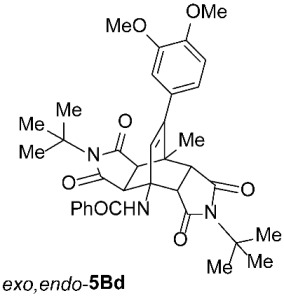


*rel*-*N*-((3a*R*,4*R*,4a*S*,7a*R*,8*S*,8a*S*)-2,6-Di-*tert*-butyl-9-(3,4-dimethoxyphenyl)-8-methyl-1,3,5,7-tetraoxo-2,3,3a,4a,5,6,7,7a,8,8a-decahydro-4,8-ethenopyrrolo[3,4-*f*]isoindol-4(1*H*)-yl)benzamide (*exo*,*exo*-**5Bd**): white solid (138 mg, 44%); *R*_f_ = 0.33 (PE/EtOAc 1/1); mp 293–295 °C (EtOH); IR (ATR) ν_max_ 3377, 2936, 1698, 1646, 1537, 1516, 1461, 1332, 1265, 1250, 1180, 1142 cm^−1^; ^1^H NMR (CDCl_3_, 500 MHz) δ 7.90–7.94 (m, 2H, ArH), 7.45–7.55 (m, 3H, ArH), 6.79 (d, *J* = 8.3 Hz, 1H, 3,4-(OCH_3_)_2_-C_6_*H*_3_), 6.65–6.67 (m, 1H, 3,4-(OCH_3_)_2_-C_6_*H*_3_), 6.62 (d, *J* = 8.3 Hz, 1H, 3,4-(OCH_3_)_2_-C_6_*H*_3_), 6.53 (s, 1H, NH), 5.82 (s, 1H, C=CH), 4.33 (d, *J* = 8.8 Hz, 2H, 3a-CH, 4a-CH), 3.88 (s, 3H, OCH_3_), 3.86 (s, 3H, OCH_3_), 2.74 (d, *J* = 8.8 Hz, 2H, 7a-CH, 8a-CH), 1.92 (s, 3H, CH_3_), 1.50 (s, 18H, 2 × C(CH_3_)_3_); ^13^C NMR (CDCl_3_, 126 MHz) δ 176.5, 175.6, 169.0, 149.1, 148.4, 146.9, 135.3, 131.6, 129.5, 128.7, 127.2, 126.5, 120.7, 111.2, 110.7, 58.6, 58.2, 55.9, 55.8, 49.6, 43.6, 43.2, 28.5, 19.4; HRMS (ESI-TOF) *m*/*z* 626.2893 (calcd for C_36_H_40_N_3_O_7_ (M − H)^−^ 626.2872); Anal. C, 68.67; H, 6.57; N, 6.74 (calcd for C_36_H_41_N_3_O_7_ C, 68.88; H, 6.58; N, 6.69).


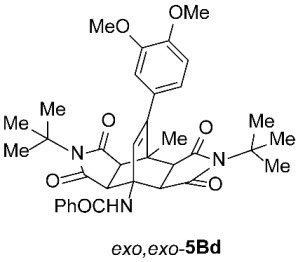


*rel*-*N*-((3a*R*,4*R*,4a*R*,7a*S*,8*S*,8a*S*)-9-(4-methoxyphenyl)-8-methyl-1,3,5,7-tetraoxo-2,6-ditrityl-2,3,3a,4a,5,6,7,7a,8,8a-decahydro-4,8-ethenopyrrolo[3,4-*f*]isoindol-4(1*H*)-yl)benzamide (*exo*,*endo*-**5Be**): white solid (425 mg, 85%); mp 292–293 °C (EtOAc); IR (ATR) ν_max_ 3390, 3062, 2930, 1704, 1656, 1528, 1514, 1334, 1316, 1201, 1162, 1027 cm^−1^; ^1^H NMR (DMSO-*d*_6_, 500 MHz) δ 8.17 (s, 1H, NH), 7.71–7.75 (m, 2H, ArH), 7.53–7.57 (m, 1H, ArH), 7.47–7.52 (m, 8H, ArH), 7.23–7.29 (m, 12H, ArH), 7.17–7.22 (m, 9H, ArH), 7.10–7.15 (m, 3H, ArH), 6.68–6.72 (m, 2H, 3,4-(OCH_3_)_2_-C_6_*H*_3_), 5.97 (s, 1H, C=CH), 5.70 (dd, *J* = 8.3, 1.8 Hz, 1H, 3,4-(OCH_3_)_2_-C_6_*H*_3_), 4.20 (d, *J* = 10.5 Hz, 1H, 4a-CH), 3.75 (s, 3H, OCH_3_), 3.66 (s, 3H, OCH_3_), 3.27 (d, *J* = 10.5 Hz, 1H, 7a-CH), 2.13 (d, *J* = 9.0 Hz, 1H, 3a-CH), 1.54 (d, *J* = 9.0 Hz, 1H, 8a-CH), 1.44 (s, 3H, CH_3_); ^13^C NMR (DMSO-*d*_6_, 126 MHz) δ 175.9, 174.9, 173.8, 173.7, 165.9, 148.3, 147.7, 146.7, 142.5, 141.6, 134.3, 132.0, 131.8, 129.5, 128.6, 128.03, 127.97, 127.6, 127.4, 127.0, 126.5, 126.3, 120.6, 112.1, 111.2, 74.2, 73.2, 57.1, 55.5, 55.4, 49.4, 44.82, 44.77, 42.5, 41.2, 19.3; HRMS (ESI-TOF) *m*/*z* 998.3826 (calcd for C_66_H_52_N_3_O_7_ (M − H)^−^ 998.3811); Anal. C, 77.86; H, 5.34; N, 4.12 (calcd for C_66_H_53_N_3_O_7_ C, 77.86; H, 5.45; N, 4.13).


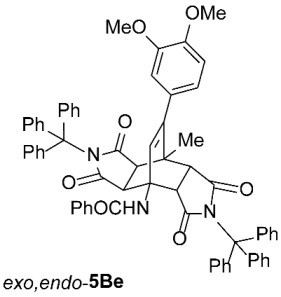


*rel*-*N*-((3a*R*,4*R*,4a*S*,7a*R*,8*S*,8a*S*)-9-benzoyl-8-methyl-1,3,5,7-tetraoxo-2,3,3a,4a,5,6,7,7a,8,8a-decahydro-4,8-ethenopyrrolo[3,4-*f*]isoindol-4(1*H*)-yl)benzamide (*exo*,*exo*-**5Ca**): white solid (181 mg, 75%); mp decomposes at 350 °C (EtOH); IR (ATR) ν_max_ 3374, 3181, 3063, 1770, 1709, 1686, 1661, 1533, 1347, 1300, 1277, 1210, 1187 cm^−1^; ^1^H NMR (DMSO-*d*_6_, 500 MHz) δ 11.35 (s, 2H, 2 × NH), 8.72 (s, 1H, 4-NH), 7.84–7.88 (m, 2H, ArH), 7.68–7.72 (m, 1H, ArH), 7.63–7.66 (m, 2H, ArH), 7.51–7.57 (m, 3H, ArH), 7.46–7.50 (m, 2H, ArH), 6.94 (s, 1H, C=CH), 4.33 (d, *J* = 8.3 Hz, 2H, 3a-CH, 4a-CH), 3.12 (d, *J* = 8.3 Hz, 2H, 7a-CH, 8a-CH), 1.84 (s, 3H, CH_3_); ^13^C NMR (DMSO-*d*_6_, 126 MHz) δ 192.0, 177.0, 176.0, 167.4, 141.8, 138.5, 136.3, 135.3, 133.6, 131.2, 129.3, 128.8, 128.1, 127.6, 57.9, 50.3, 44.6, 41.1, 17.3; HRMS (ESI-TOF) *m*/*z* 484.1500 (calcd for C_27_H_22_N_3_O_6_ (M + H)^+^ 484.1503); Anal. C, 66.66; H, 4.24; N, 8.73 (calcd for C_27_H_21_N_3_O_6_ · 1/6 H_2_O C, 66.66; H, 4.42; N, 8.64).


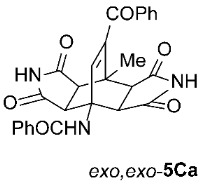


*rel*-*N*-((3a*R*,4*R*,4a*R*,7a*S*,8*S*,8a*S*)-9-benzoyl-8-methyl-1,3,5,7-tetraoxo-2,6-ditrityl-2,3,3a,4a,5,6,7,7a,8,8a-decahydro-4,8-ethenopyrrolo[3,4-*f*]isoindol-4(1*H*)-yl)benzamide (*exo*,*endo*-**5Ce**): white solid (382 mg, 79%); *R*_f_ = 0.57 (PE/EtOAc 1/1); mp 283–285 °C (EtOAc); IR (ATR) ν_max_ 3388, 3055, 3023, 1708, 1651, 1526, 1490, 1448, 1321, 1264, 1200, 1162 cm^−1^; ^1^H NMR (CDCl_3_, 500 MHz) δ 7.89–7.92 (m, 2H, ArH), 7.70–7.74 (m, 2H, ArH), 7.61–7.65 (m, 1H, ArH), 7.51–7.55 (m, 3H, ArH, NH), 7.45–7.49 (m, 1H, ArH), 7.35–7.41 (m, 8H, ArH), 7.27–7.30 (m, 6H, ArH), 7.21–7.26 (m, 6H, ArH), 7.11–7.20 (m, 9H, ArH), 7.02–7.06 (m, 3H, ArH), 6.73 (s, 1H, C=CH), 4.63 (d, *J* = 10.3 Hz, 1H, 4a-CH), 3.06 (d, *J* = 10.3 Hz, 1H, 7a-CH), 1.82 (d, *J* = 9.0 Hz, 1H, 3a-CH), 1.68 (s, 3H, CH_3_), 1.23 (d, *J* = 9.0 Hz, 1H, 8a-CH); ^13^C NMR (CDCl_3_, 126 MHz) δ 192.7, 174.6, 174.5, 174.4, 172.7, 167.7, 144.1, 141.8, 141.5, 139.9, 136.6, 133.8, 133.7, 132.0, 129.9, 128.9, 128.6, 128.4, 127.9, 127.7, 127.5, 127.3, 126.9, 126.7, 75.5, 75.0, 57.0, 49.6, 44.6, 44.1, 42.8, 41.8, 17.1; HRMS (ESI-TOF) *m*/*z* 966.3560 (calcd for C_65_H_48_N_3_O_6_ (M − H)^−^ 966.3549); Anal. C, 80.47; H, 4.88; N, 4.34 (calcd for C_65_H_49_N_3_O_6_ C, 80.64; H, 5.10; N, 4.34).


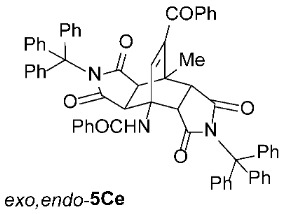


Ethyl *rel*-(3a*R*,4*R*,4a*S*,7a*R*,8*S*,8a*S*)-4-Benzamido-8-methyl-1,3,5,7-tetraoxo-1,2,3,3a,4,4a,5,6,7,7a,8,8a-dodecahydro-4,8-ethenopyrrolo[3,4-*f*]isoindole-9-carboxylate (*exo*,*exo*-**5Da**): white solid (194 mg, 86%); mp 339–341 °C (EtOH); IR (ATR) ν_max_ 3357, 3207, 3044, 1716, 1693, 1654, 1548, 1340, 1307, 1267, 1184, 1154, 1045 cm^−1^; ^1^H NMR (DMSO-*d*_6_, 500 MHz) δ 11.18 (s, 2H, 2 × NH), 8.80 (s, 1H, 4-NH), 7.89–7.93 (m, 2H, ArH), 7.55–7.59 (m, 1H, ArH), 7.49–7.53 (m, 2H, ArH), 7.42 (s, 1H, C=CH), 4.25 (d, *J* = 8.3 Hz, 2H, 3a-CH, 4a-CH), 4.10 (q, *J* = 7.3 Hz, 2H, CH_3_C*H*_2_), 3.03 (d, *J* = 8.3 Hz, 2H, 7a-CH, 8a-CH), 1.91 (s, 3H, CH_3_), 1.19 (t, *J* = *7*.3 Hz, 3H, C*H*_3_CH_2_); ^13^C NMR (DMSO-*d*_6_, 126 MHz) δ 176.9, 175.8, 167.5, 163.1, 139.8, 135.3, 134.8, 131.2, 128.1, 127.6, 60.6, 57.9, 50.3, 44.1, 40.8, 18.2, 14.0; HRMS (ESI-TOF) *m*/*z* 452.1456 (calcd for C_23_H_22_N_3_O_7_ (M + H)^+^ 452.1452); Anal. C, 60.83; H, 4.43; N, 9.20 (calcd for C_23_H_21_N_3_O_7_ C, 61.19; H, 4.69; N, 9.31).


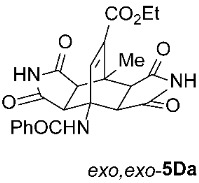


Ethyl *rel*-(3a*R*,4*R*,4a*R*,7a*S*,8*S*,8a*S*)-4-Benzamido-8-methyl-1,3,5,7-tetraoxo-2,6-ditrityl-1,2,3,3a,4,4a,5,6,7,7a,8,8a-dodecahydro-4,8-ethenopyrrolo[3,4-*f*]isoindole-9-carboxylate (*exo*,*endo*-**5De**): white solid (379 mg, 81%); *R*_f_ = 0.59 (PE/EtOAc 1/1); mp 286–288 °C (EtOH); IR (ATR) ν_max_ 3399, 3057, 3033, 1708, 1667, 1519, 1488, 1449, 1308, 1267, 1196, 1156, 1034 cm^−1^; ^1^H NMR (CDCl_3_, 500 MHz) δ 7.73–7.76 (m, 2H, ArH), 7.47–7.51 (m, 1H, ArH), 7.47 (s, 1H, NH), 7.35–7.42 (m, 8H, ArH), 7.28 (s, 1H, C=CH), 7.16–7.26 (m, 15H, ArH), 7.09–7.13 (m, 6H, ArH), 6.96–7.00 (m, 3H, ArH), 4.33 (d, *J* = 10.5 Hz, 1H, 4a-CH), 4.24 (q, *J* = 7.1 Hz, 2H, CH_3_C*H*_2_), 2.98 (d, *J* = 10.5 Hz, 1H, 7a-CH), 1.79 (s, 3H, CH_3_), 1.70 (d, *J* = 9.0 Hz, 1H, 3a-CH), 1.27 (t, *J* = 7.1 Hz, 3H, C*H*_3_CH_2_), 1.10 (d, *J* = 9.0 Hz, 1H, 8a-CH); ^13^C NMR (CDCl_3_, 126 MHz) δ 174.5, 174.4, 174.0, 172.9, 167.6, 163.3, 143.7, 141.8, 141.4, 138.2, 133.6, 132.1, 128.7, 128.1, 127.8, 127.6, 127.5, 127.3, 126.84, 126.75, 75.6, 74.6, 61.4, 57.3, 50.0, 44.7, 43.8, 42.5, 41.2, 18.4, 14.1; HRMS (ESI-TOF) *m*/*z* 966.3560 (calcd for C_61_H_48_N_3_O_7_ (M − H)^−^ 966.3549); Anal. C, 77.89; H, 5.06; N, 4.60 (calcd for C_61_H_49_N_3_O_7_ C, 78.27; H, 5.28; N, 4.49).


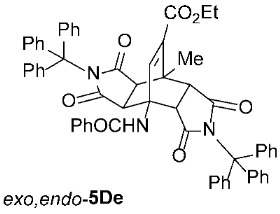


*rel*-*N*-((3a*S*,4*R*,9a*S*,9b*R*,10*R*,14*S*)-1,3,11,13-tetraoxo-2,3,3a,6,7,8,9,9b-octahydro-4,9a-[3,4]epipyrrolobenzo[*e*]isoindol-4(1*H*)-yl)benzamide (*exo*,*exo*-**5Ea**): white solid (168 mg, 80%); mp 335–337 °C (EtOH); IR (ATR) ν_max_ 3345, 3137, 3052, 2940, 1718, 1698, 1640, 1543, 1346, 1309, 1213, 1197 cm^−1^; ^1^H NMR (DMSO-*d*_6_, 500 MHz) δ 11.11 (s, 2H, 2 × NH), 8.49 (s, 1H, 4-NH), 7.85–7.89 (m, 2H, ArH), 7.52–7.56 (m, 1H, ArH), 7.46–7.51 (m, 2H, ArH), 6.12 (s, 1H, C=CH), 4.10 (d, *J* = 8.3 Hz, 2H, 3a-CH, 10-CH), 2.96 (d, *J* = 8.3 Hz, 2H, 9b-CH, 14-CH), 2.58 (t, *J* = 6.8 Hz, 2H, 9-CH_2_), 2.16 (t, *J* = 5.8 Hz, 2H, 6-CH_2_), 1.55–1.61 (m, 2H, 8-CH_2_), 1.31–1.38 (m, 2H, 7-CH_2_); ^13^C NMR (DMSO-*d*_6_, 126 MHz) δ 178.3, 176.4, 167.5, 140.8, 135.7, 130.9, 128.0, 127.7, 123.6, 57.2, 50.3, 44.8, 40.3, 29.2, 26.3, 22.9, 21.0; HRMS (ESI-TOF) *m*/*z* 420.1546 (calcd for C_23_H_22_N_3_O_5_ (M + H)^+^ 420.1554); Anal. C, 65.54; H, 5.22; N, 10.00 (calcd for C_23_H_21_N_3_O_5_ C, 65.86; H, 5.05; N, 10.02).


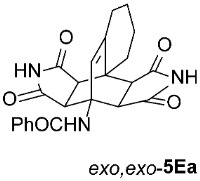


*rel*-*N*-((3a*R*,4*R*,9a*S*,9b*S*,10*R*,14*S*)-1,3,11,13-tetraoxo-2,12-ditrityl-2,3,3a,6,7,8,9,9b-octahydro-4,9a-[3,4]epipyrrolobenzo[*e*]isoindol-4(1*H*)-yl)benzamide (*exo*,*endo*-**5Ee**): white solid (316 mg, 70%); *R*_f_ = 0.53 (PE/EtOAc 1/1); mp 271–272 °C (EtOH/Me_2_CO); IR (ATR) ν_max_ 3438, 3271, 3054, 2937, 1706, 1641, 1486, 1449, 1316, 1305, 1199, 1153 cm^−1^; ^1^H NMR (CDCl_3_, 500 MHz) δ 7.71–7.75 (m, 2H, ArH), 7.53 (s, 1H, NH), 7.43–7.48 (m, 1H, ArH), 7.34–7.42 (m, 8H, ArH), 7.13–7.26 (m, 21H, ArH), 7.02–7.07 (m, 3H, ArH), 5.95 (s, 1H, C=CH), 4.29 (d, *J* = 10.5 Hz, 1H, 10-CH), 2.99 (d, *J* = 10.5 Hz, 1H, 9b-CH), 2.38–2.45 (m, 1H, 9-CH_2_), 2.27–2.35 (m, 1H, 9-CH_2_), 2.18–2.25 (m, 1H, 6-CH_2_), 2.02–2.11 (m, 1H, 6-CH_2_), 1.64–1.74 (m, 2H, 7-CH_2_, 8-CH_2_), 1.57 (d, *J* = 8.8 Hz, 1H, 14-CH), 1.54–1.62 (m, 1H, 8-CH_2_), 1.39–1.48 (m, 1H, 7-CH_2_), 1.38 (d, *J* = 8.8 Hz, 1H, 3a-CH); ^13^C NMR (CDCl_3_, 126 MHz) δ 176.0, 175.0, 174.8, 174.2, 167.4, 145.9, 142.0, 141.5, 134.1, 131.7, 128.5, 128.1, 127.9, 127.6, 127.5, 127.2, 127.0, 126.70, 126.68, 75.2, 74.2, 57.4, 45.1, 44.4, 43.6, 42.6, 42.2, 27.0, 22.9, 19.8, 17.8; HRMS (ESI-TOF) *m*/*z* 902.3596 (calcd for C_61_H_48_N_3_O_5_ (M − H)^−^ 902.3599); Anal. C, 80.14; H, 5.53; N, 4.71 (calcd for C_61_H_49_N_3_O_5_ · 1/2 H_2_O C, 80.24; H, 5.52; N, 4.60).


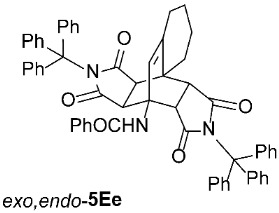


*rel*-*N*-((3a*R*,4*R*,4a*R*,7a*S*,8*S*,8a*S*)-9-acetyl-8-methyl-1,3,5,7-tetraoxo-2,6-ditrityl-2,3,3a,4a,5,6,7,7a,8,8a-decahydro-4,8-ethenopyrrolo[3,4-*f*]isoindol-4(1*H*)-yl)benzamide (*exo*,*endo*-**5Fe**): white solid (340 mg, 75%); *R*_f_ = 0.45 (PE/EtOAc 1/1); mp 216–218 °C (Me_2_CO); IR (ATR) ν_max_ 3364, 3062, 1701, 1673, 1527, 1488, 1324, 1313, 1200, 1163 cm^−1^; ^1^H NMR (DMSO-*d*_6_, 500 MHz) δ 8.52 (s, 1H, NH), 7.80–7.84 (m, 2H, ArH), 7.54–7.59 (m, 1H, ArH), 7.42–7.52 (m, 8H, ArH), 7.07–7.28 (m, 25H, ArH, C=CH), 4.07 (d, *J* = 10.5 Hz, 1H, 4a-CH), 2.96 (d, *J* = 10.5 Hz, 1H, 7a-CH), 2.39 (d, *J* = 9.0 Hz, 1H, 3a-CH), 2.03 (s, 3H, COCH_3_), 1.55 (s, 3H, CH_3_), 1.03 (d, *J* = 9.0 Hz, 1H, 8a-CH); ^13^C NMR (DMSO-*d*_6_, 126 MHz) δ 196.8, 175.0, 174.5, 173.1, 172.9, 166.2, 144.3, 143.2, 142.5, 141.7, 133.9, 131.9, 128.5, 127.93, 127.90, 127.5, 127.37, 127.36, 126.4, 126.3, 74.3, 73.3, 56.6, 49.3, 43.9, 43.5, 42.8, 40.5, 28.4, 17.7; HRMS (ESI-TOF) *m*/*z* 904.3387 (calcd for C_60_H_46_N_3_O_6_ (M − H)^−^ 904.3392); Anal. C, 79.46; H, 5.13; N, 4.66 (calcd for C_60_H_47_N_3_O_6_ C, 79.54; H, 5.23; N, 4.64).


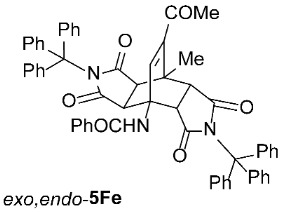


Methyl *rel*-(3a*R*,4*R*,4a*R*,7a*S*,8*S*,8a*S*)-4-Benzamido-8-(2-methoxy-2-oxoethyl)-1,3,5,7-tetraoxo-2,6-ditrityl-1,2,3,3a,4,4a,5,6,7,7a,8,8a-dodecahydro-4,8-ethenopyrrolo[3,4-*f*]isoindole-9-carboxylate (*exo*,*endo*-**5Ge**): white solid (353 mg, 72%); *R*_f_ = 0.53 (PE/EtOAc 1/1); mp 278–280 °C (EtOAc/Me_2_CO); IR (ATR) ν_max_ 3372, 3056, 1721, 1698, 1672, 1519, 1488, 1325, 1313, 1273, 1201, 1168 cm^−1^; ^1^H NMR (DMSO-*d*_6_, 500 MHz) δ 8.77 (s, 1H, NH), 7.82–7.86 (m, 2H, ArH), 7.54–7.58 (m, 1H, ArH), 7.42–7.51 (m, 8H, ArH), 7.05–7.27 (m, 25H, ArH, C=CH), 4.04 (d, *J* = 10.3 Hz, 1H, 4a-CH), 3.73 (d, *J* = 10.3 Hz, 1H, 7a-CH), 3.65 (d, *J* = 18.3 Hz, 1H, CH_2_), 3.58 (s, 3H, CO_2_CH_3_), 3.57 (s, 3H, CO_2_CH_3_), 2.95 (d, *J* = 18.3 Hz, 1H, CH_2_), 2.57 (d, *J* = 8.8 Hz, 1H, 3a-CH), 0.84 (d, *J* = 8.8 Hz, 1H, 8a-CH); ^13^C NMR (DMSO-*d*_6_, 126 MHz) δ 175.6, 174.6, 172.6, 172.2, 171.8, 166.3, 163.5, 146.4, 142.3, 141.5, 133.7, 132.8, 131.9, 128.4, 127.9, 127.8, 127.5, 127.4, 126.5, 126.4, 74.5, 73.6, 56.5, 52.0, 51.4, 44.9, 42.9, 42.61, 42.57, 40.8, 32.4 (1 signal hidden); HRMS (ESI-TOF) *m*/*z* 978.3395 (calcd for C_62_H_48_N_3_O_9_ (M − H)^−^ 978.3396); Anal. C, 75.71; H, 4.88; N, 4.28 (calcd for C_62_H_49_N_3_O_9_ C, 75.98; H, 5.04; N, 4.29).


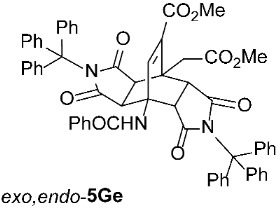


*rel*-*N*-((3a*R*,4*R*,10a*S*,10b*S*,11*R*,15*S*)-1,3,12,14-tetraoxo-2,13-ditrityl-2,3,3a,7,8,9,10,10b-octahydro-1*H*-4,10a-[3,4]epipyrrolocyclohepta[*e*]isoindol-4(6*H*)-yl)benzamide (*exo*,*endo*-**5He**): white solid (353 mg, 77%); *R*_f_ = 0.52 (PE/EtOAc 1/1); mp 271–272 °C (EtOH); IR (ATR) ν_max_ 3412, 3058, 2923, 1704, 1650, 1514, 1487, 1450, 1317, 1198, 1156 cm^−1^; ^1^H NMR (CDCl_3_, 500 MHz) δ 7.71–7.75 (m, 2H, ArH), 7.57 (s, 1H, NH), 7.45–7.49 (m, 1H, ArH), 7.35–7.42 (m, 8H, ArH), 7.14–7.24 (m, 21H, ArH), 7.04–7.08 (m, 3H, ArH), 5.90 (s, 1H, C=CH), 4.33 (d, *J* = 10.5 Hz, 1H, 11-CH), 3.25 (d, *J* = 10.5 Hz, 1H, 10b-CH), 2.27–2.37 (m, 3H, 6-CH_2_, 10-CH_2_), 2.00 (t, *J* = 12.8 Hz, 1H, 6-CH_2_), 1.81–1.93 (m, 3H, 7-CH_2_, 8-CH_2_, 9-CH_2_), 1.55–1.63 (m, 1H, 9-CH_2_), 1.51 (d, *J* = 8.5 Hz, 1H, 3a-CH), 1.43 (d, *J* = 8.5 Hz, 1H, 15-CH), 1.13–1.32 (m, 2H, 7-CH_2_, 8-CH_2_); ^13^C NMR (CDCl_3_, 126 MHz) δ 175.3, 175.1, 174.0, 167.3, 149.5, 142.1, 141.5, 134.1, 131.8, 128.8, 128.6, 128.1, 128.0, 127.6, 127.5, 127.3, 126.7, 75.2, 74.2, 57.4, 46.4, 45.5, 44.6, 44.1, 42.8, 35.3, 31.0, 29.5, 29.2, 24.3 (2 signals hidden); HRMS (ESI-TOF) *m*/*z* 918.3915 (calcd for C_62_H_52_N_3_O_5_ (M + H)^+^ 918.3907); Anal. C, 80.28; H, 5.63; N, 4.55 (calcd for C_62_H_51_N_3_O_5_ · 1/2 H_2_O C, 80.32; H, 5.65; N, 4.53).


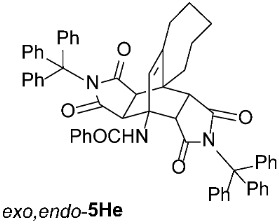


*rel*-*N*-((3a*R*,4*R*,8a*S*,8b*S*,9*R*,13*S*)-1,3,10,12-tetraoxo-2,11-ditrityl-2,3,3a,7,8,8b-hexahydro-1*H*-4,8a-[3,4]epipyrrolocyclopenta[*e*]isoindol-4(6*H*)-yl)benzamide (*exo*,*endo*-**5Ie**): white solid (245 mg, 55%); *R*_f_ = 0.46 (PE/EtOAc 1/1); mp 305–307 °C (EtOH); IR (ATR) ν_max_ 3056, 1706, 1650, 1521, 1488, 1450, 1319, 1306, 1198, 1154 cm^−1^; ^1^H NMR (DMSO-*d*_6_, 500 MHz) δ 8.22 (s, 1H, NH), 7.74–7.77 (m, 2H, ArH), 7.52–7.56 (m, 1H, ArH), 7.43–7.50 (m, 8H, ArH), 7.08–7.28 (m, 24H, ArH), 5.86 (s, 1H, C=CH), 3.99 (d, *J* = 10.5 Hz, 1H, 9-CH), 2.87 (d, *J* = 10.5 Hz, 1H, 8b-CH), 2.02–2.23 (m, 4H, 3a-CH, 6-CH_2_, 8-CH_2_), 1.65–1.73 (m, 1H, 6-CH_2_), 1.54–1.63 (m, 1H, 7-CH_2_), 1.27–1.37 (m, 1H, 7-CH_2_), 1.18 (d, *J* = 8.8 Hz, 1H, 13-CH); ^13^C NMR (DMSO-*d*_6_, 126 MHz) δ 176.7, 175.2, 174.3, 173.9, 165.9, 148.8, 142.5, 141.6, 134.4, 131.6, 128.5, 127.94, 127.87, 127.5, 127.3, 127.1, 126.4, 126.2, 124.6, 74.0, 73.2, 57.9, 48.3, 48.2, 44.4, 44.1, 40.8, 30.2, 28.9, 25.1; HRMS (ESI-TOF) *m*/*z* 888.3456 (calcd for C_60_H_46_N_3_O_5_ (M − H)^−^ 888.3443); Anal. C, 80.32; H, 5.25; N, 4.70 (calcd for C_60_H_47_N_3_O_5_ · 1/3 H_2_O C, 80.43; H, 5.36; N, 4.69).


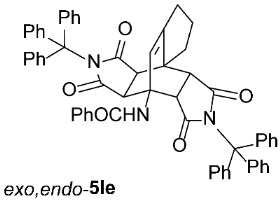


*rel*-*N*-((3a*R*,4*R*,4a*R*,7a*S*,8*S*,8a*S*)-1,3,5,7-tetraoxo-8,9-diphenyl-2,6-ditrityl-2,3,3a,4a,5,6,7,7a,8,8a-decahydro-4,8-ethenopyrrolo[3,4-*f*]isoindol-4(1*H*)-yl)benzamide (*exo*,*endo*-**5Je**): white solid (281 mg, 56%); *R*_f_ = 0.59 (PE/EtOAc 1/1); mp 248–250 °C (EtOH); IR (ATR) ν_max_ 3388, 3057, 1710, 1669, 1533, 1490, 1449, 1316, 1197, 1148 cm^−1^; ^1^H NMR (CDCl_3_, 500 MHz) δ 7.73–7.77 (m, 2H, ArH), 7.48–7.61 (m, 3H, ArH, NH), 7.38–7.42 (m, 2H, ArH), 7.31–7.34 (m, 6H, ArH), 6.96–7.19 (m, 29H, ArH), 6.85–6.95 (m, 1H, ArH), 6.79–6.83 (m, 2H, ArH), 6.60 (s, 1H, C=CH), 6.42–6.58 (m, 1H, ArH), 4.46 (d, *J* = 10.5 Hz, 1H, 4a-CH), 3.51 (d, *J* = 10.5 Hz, 1H, 7a-CH), 2.92 (d, *J* = 8.3 Hz, 1H, 8a-CH), 1.28 (d, *J* = 8.3 Hz, 1H, 3a-CH); ^13^C NMR (CDCl_3_, 126 MHz) δ 174.7, 174.0, 173.80, 173.78, 167.5, 147.8, 142.0, 141.5, 137.0, 136.4, 134.0, 133.3, 132.0, 131.0, 130.0, 129.5, 128.6, 128.0, 127.9, 127.6, 127.50, 127.46, 127.3, 126.7, 126.58, 126.57, 75.3, 74.7, 58.1, 53.0, 50.9, 45.3, 43.8, 41.9 (1 signal hidden); HRMS (ESI-TOF) *m*/*z* 1000.3746 (calcd for C_69_H_50_N_3_O_5_ (M − H)^−^ 1000.3756); Anal. C, 82.07; H, 5.16; N, 4.15 (calcd for C_69_H_51_N_3_O_5_ · 1/3 H_2_O C, 82.20; H, 5.17; N, 4.17).


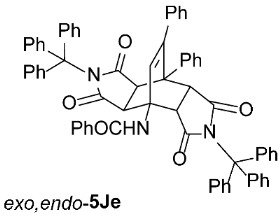


*rel*-*N*-((3a*R*,4*R*,4a*R*,7a*S*,8*S*,8a*S*)-9-(4-methoxyphenyl)-8-methyl-1,3,5,7-tetraoxo-2,6-ditrityl-2,3,3a,4a,5,6,7,7a,8,8a-decahydro-4,8-ethenopyrrolo[3,4-*f*]isoindol-4(1*H*)-yl)acetamide (*exo*,*endo*-**5Ke**): white solid (350 mg, 77%); *R*_f_ = 0.37 (PE/EtOAc 1/1); mp 216–218 °C (EtOH); IR (ATR) ν_max_ 3397, 3057, 1705, 1675, 1510, 1492, 1449, 1323, 1288, 1245, 1198, 1182, 1154, 1034 cm^−1^; ^1^H NMR (CDCl_3_, 500 MHz) δ 7.42–7.47 (m, 6H, ArH), 7.11–7.26 (m, 24H, ArH), 6.91 (s, 1H, NH), 6.72 and 6.66 (AA’XX’, *J* = 9.0 Hz, 2H each, 4-OCH_3_-C_6_*H*_4_), 5.97 (s, 1H, C=CH), 4.32 (d, *J* = 10.5 Hz, 1H, 4a-CH), 3.79 (s, 3H, OCH_3_), 2.81 (d, *J* = 10.5 Hz, 1H, 7a-CH), 1.97 (s, 3H, COCH_3_), 1.78 (d, *J* = 9.0 Hz, 1H, 8a-CH), 1.60 (s, 3H, CH_3_), 1.44 (d, *J* = 9.0 Hz, 1H, 3a-CH); ^13^C NMR (CDCl_3_, 126 MHz) δ 175.7, 174.71, 174.68, 174.6, 170.6, 159.1, 148.0, 142.1, 141.4, 132.1, 130.1, 129.0, 128.2, 128.0, 127.7, 127.6, 126.78, 126.77, 113.2, 75.2, 74.1, 57.4, 55.2, 49.8, 45.1, 44.3, 42.3, 41.7, 24.2, 19.0; HRMS (ESI-TOF) *m*/*z* 906.3549 (calcd for C_60_H_48_N_3_O_6_ (M − H)^−^ 906.3549); Anal. C, 78.13; H, 5.21; N, 4.57 (calcd for C_60_H_49_N_3_O_6_ · 2/3 H_2_O C, 78.33; H, 5.51; N, 4.57).


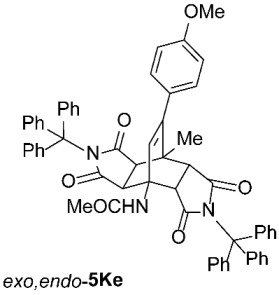


### 3.4. Synthesis of exo,endo-**5A**–**Ea** by Acid-Induced Elimination of the Triphenylmethyl Group from exo,endo-**5A**–**Ee**

A mixture of the starting bicyclo[2.2.2]octene *exo*,*endo*-**5A**–**Ee** (0.3 mmol), *n*-BuOH (3 mL), and TFA (1 mL) was irradiated in the focused microwave equipment for 1 h. The power was set to 100 W, the final temperature to 100 °C or 140 °C, and the ramp time to 5 min (Table 4). In the case of **5De**, *n*-BuOH was replaced with ethanol (3 mL), and the volume of TFA was 0.3 mL. Afterward, the reaction mixture was cooled, and the precipitated material was filtered off and washed with cold EtOH (1 mL), yielding the destrityl *exo*,*endo*-**5A**–**Ea** products.

*rel*-*N*-((3a*R*,4*R*,4a*R*,7a*S*,8*S*,8a*S*)-9-(4-methoxyphenyl)-8-methyl-1,3,5,7-tetraoxo-2,3,3a,4a,5,6,7,7a,8,8a-decahydro-4,8-ethenopyrrolo[3,4-*f*]isoindol-4(1*H*)-yl)benzamide (*exo*,*endo*-**5Aa**): white solid (124 mg, 85%); mp 293–295 °C (EtOH); IR (ATR) ν_max_ 3392, 3200, 1724, 1699, 1642, 1541, 1508, 1340, 1316, 1241, 1194, 1177 cm^−1^; ^1^H NMR (DMSO-*d*_6_, 500 MHz) δ 11.63 (s, 1H, NH), 11.42 (s, 1H, NH), 8.42 (s, 1H, 4-NH), 7.86–7.91 (m, 2H, ArH), 7.58–7.63 (m, 1H, ArH), 7.52–7.57 (m, 2H, ArH), 7.00 and 6.90 (AA’XX’, *J* = 9.0 Hz, 2H each, 4-OCH_3_-C_6_*H*_4_), 6.15 (s, 1H, C=CH), 4.09 (d, *J* = 10.0 Hz, 1H, 4a-CH), 3.75 (s, 3H, OCH_3_), 3.28 (d, *J* = 7.8 Hz, 1H, 3a-CH), 3.11 (d, *J* = 10.0 Hz, 1H, 7a-CH), 2.91 (d, *J* = 7.8 Hz, 1H, 8a-CH), 1.56 (s, 3H, CH_3_); ^13^C NMR (DMSO-*d*_6_, 126 MHz) δ 178.0, 177.9, 177.7, 176.4, 166.1, 158.7, 145.8, 134.7, 132.1, 131.6, 129.9, 129.4, 128.6, 127.1, 113.4, 56.7, 55.1, 51.8, 46.6, 46.2, 44.5, 41.1, 18.8; HRMS (ESI-TOF) *m*/*z* 486.1653 (calcd for C_27_H_24_N_3_O_6_ (M + H)^+^ 486.1660); Anal. C, 66.31; H, 4.88; N, 8.50 (calcd for C_27_H_23_N_3_O_6_ · 1/5 H_2_O C, 66.30; H, 4.82; N, 8.59).


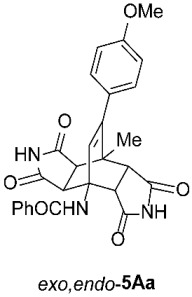


*rel*-*N*-((3a*R*,4*R*,4a*R*,7a*S*,8*S*,8a*S*)-9-(3,4-dimethoxyphenyl)-8-methyl-1,3,5,7-tetraoxo-2,3,3a,4a,5,6,7,7a,8,8a-decahydro-4,8-ethenopyrrolo[3,4-*f*]isoindol-4(1*H*)-yl)benzamide (*exo*,*endo*-**5Ba**): white solid (134 mg, 87%); mp 320–322 °C (EtOH/H_2_O); IR (ATR) ν_max_ 3372, 3180, 1719, 1705, 1640, 1536, 1515, 1489, 1343, 1254, 1200, 1137 cm^−1^; ^1^H NMR (DMSO-*d*_6_, 500 MHz) δ 11.64 (s, 1H, NH), 11.41 (s, 1H, NH), 8.42 (s, 1H, 4-NH), 7.87–7.90 (m, 2H, ArH), 7.58–7.63 (m, 1H, ArH), 7.53–7.57 (m, 2H, ArH), 6.91 (d, *J* = 8.3 Hz, 1H, 3,4-(OCH_3_)_2_-C_6_*H*_3_), 6.66 (d, *J* = 1.7 Hz, 1H, 3,4-(OCH_3_)_2_-C_6_*H*_3_), 6.57 (dd, *J* = 8.3, 1.7 Hz, 1H, 3,4-(OCH_3_)_2_-C_6_*H*_3_), 6.16 (s, 1H, C=CH), 4.09 (d, *J* = 9.8 Hz, 1H, 4a-CH), 3.75 (s, 3H, OCH_3_), 3.74 (s, 3H, OCH_3_), 3.28 (d, *J* = 8.0 Hz, 1H, 3a-CH), 3.17 (d, *J* = 9.8 Hz, 1H, 7a-CH), 2.90 (d, *J* = 8.0 Hz, 1H, 8a-CH), 1.57 (s, 3H, CH_3_); ^13^C NMR (DMSO-*d*_6_, 126 MHz) δ 177.9, 177.8, 177.7, 176.5, 166.1, 148.3, 147.9, 146.0, 134.7, 132.0, 131.6, 130.2, 128.6, 127.1, 120.6, 112.1, 111.3, 56.7, 55.5, 55.4, 51.7, 46.6, 46.1, 44.6, 41.2, 18.8; HRMS (ESI-TOF) *m*/*z* 516.1766 (calcd for C_28_H_26_N_3_O_7_ (M + H)^+^ 516.1765); Anal. C, 64.98; H, 4.58; N, 8.07 (calcd for C_28_H_25_N_3_O_7_ C, 65.24; H, 4.89; N, 8.15).


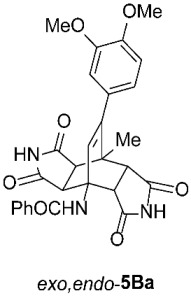


*rel*-*N*-((3a*R*,4*R*,4a*R*,7a*S*,8*S*,8a*S*)-9-benzoyl-8-methyl-1,3,5,7-tetraoxo-2,3,3a,4a,5,6,7,7a,8,8a-decahydro-4,8-ethenopyrrolo[3,4-*f*]isoindol-4(1*H*)-yl)benzamide (*exo*,*endo*-**5Ca**): white solid (135 mg, 93%); mp 342–344 °C (EtOH); IR (ATR) ν_max_ 3367, 3254, 3052, 1726, 1708, 1660, 1552, 1346, 1324, 1207, 1188 cm^−1^; ^1^H NMR (DMSO-*d*_6_, 500 MHz) δ 11.70 (s, 1H, NH), 11.50 (s, 1H, NH), 8.66 (s, 1H, 4-NH), 7.86–7.90 (m, 2H, ArH), 7.72–7.76 (m, 2H, ArH), 7.66–7.71 (m, 1H, ArH), 7.57–7.61 (m, 1H, ArH), 7.50–7.56 (m, 4H, ArH), 6.68 (s, 1H, C=CH), 4.04 (d, *J* = 9.8 Hz, 1H, 4a-CH), 3.45 (d, *J* = 8.3 Hz, 1H, 3a-CH), 3.12 (d, *J* = 9.8 Hz, 1H, 7a-CH), 2.95 (d, *J* = 8.3 Hz, 1H, 8a-CH), 1.74 (s, 3H, CH_3_); ^13^C NMR (DMSO-*d*_6_, 126 MHz) δ 192.6, 177.14, 177.09, 176.9, 175.8, 166.5, 142.4, 141.6, 136.4, 134.3, 133.7, 131.7, 129.4, 128.8, 128.4, 127.4, 56.7, 51.8, 46.3, 46.1, 44.4, 40.1, 17.4; HRMS (ESI-TOF) *m*/*z* 484.1515 (calcd for C_27_H_22_N_3_O_6_ (M + H)^+^ 484.1503); Anal. C, 66.39; H, 4.16; N, 8.57 (calcd for C_27_H_21_N_3_O_6_ · 1/3 H_2_O C, 66.25; H, 4.46; N, 8.58).


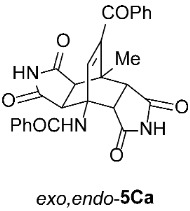


Ethyl *rel*-(3a*R*,4*R*,4a*R*,7a*S*,8*S*,8a*S*)-4-Benzamido-8-methyl-1,3,5,7-tetraoxo-1,2,3,3a,4,4a,5,6,7,7a,8,8a-dodecahydro-4,8-ethenopyrrolo[3,4-*f*]isoindole-9-carboxylate (*exo*,*endo*-**5Da**): white solid (119 mg, 88%); mp 334–336 °C (EtOH); IR (ATR) ν_max_ 3379, 3154, 3042, 2934, 1708, 1664, 1529, 1352, 1315, 1286, 1237, 1202, 1042 cm^−1^; ^1^H NMR (DMSO-*d*_6_, 500 MHz) δ 11.49 (s, 1H, NH), 11.45 (s, 1H, NH), 8.63 (s, 1H, 4-NH), 7.89–7.94 (m, 2H, ArH), 7.61–7.64 (m, 1H, ArH), 7.52–7.58 (m, 2H, ArH), 7.13 (s, 1H, C=CH), 4.06–4.15 (m, 2H, CH_3_C*H*_2_), 3.90 (d, *J* = 10.0 Hz, 1H, 4a-CH), 3.36 (d, *J* = 8.0 Hz, 1H, 3a-CH), 2.95 (d, *J* = 9.5 Hz, 1H, 7a-CH), 2.85 (d, *J* = 8.0 Hz, 1H, 8a-CH), 1.85 (s, 3H, CH_3_), 1.20 (t, *J* = 7.0 Hz, 3H, C*H*_3_CH_2_); ^13^C NMR (DMSO-*d*_6_, 126 MHz) δ 177.1, 176.9, 176.7, 175.8, 166.5, 163.5, 143.7, 135.8, 134.4, 131.7, 128.5, 127.4, 60.6, 56.6, 51.7, 46.4, 45.5, 44.0, 39.9, 17.7, 14.0; HRMS (ESI-TOF) *m*/*z* 452.1435 (calcd for C_23_H_22_N_3_O_7_ (M + H)^+^ 452.1452); Anal. C, 60.74; H, 4.48; N, 9.14 (calcd for C_23_H_21_N_3_O_7_ · 1/6 H_2_O C, 60.79; H, 4.73; N, 9.25).


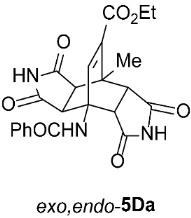


*rel*-*N*-((3a*R*,4*R*,9a*S*,9b*S*,10*R*,14*S*)-1,3,11,13-tetraoxo-2,3,3a,6,7,8,9,9b-octahydro-4,9a-[3,4]epipyrrolobenzo[*e*]isoindol-4(1*H*)-yl)benzamide (*exo*,*endo*-**5Ea**): white solid (114 mg, 91%); mp 335–337 °C (EtOH); IR (ATR) ν_max_ 3394, 3136, 3073, 2950, 1703, 1641, 1523, 1340, 1312, 1179 cm^−1^; ^1^H NMR (DMSO-*d*_6_, 500 MHz) δ 11.50 (s, 1H, NH), 11.34 (s, 1H, NH), 8.28 (s, 1H, 4-NH), 7.83–7.87 (m, 2H, ArH), 7.58–7.63 (m, 1H, ArH), 7.53–7.57 (m, 2H, ArH), 5.98 (s, 1H, C=CH), 3.97 (d, *J* = 9.8 Hz, 1H, 10-CH), 3.13 (d, *J* = 8.0 Hz, 1H, 3a-CH), 3.07 (d, *J* = 9.8 Hz, 1H, 9b-CH), 2.83 (d, *J* = 8.0 Hz, 1H, 14-CH), 2.41–2.48 (m, 1H, 9-CH_2_), 2.28–2.35 (m, 1H, 9-CH_2_), 2.17–2.25 (m, 1H, 6-CH_2_), 1.95–2.04 (m, 1H, 6-CH_2_), 1.57–1.76 (m, 3H, 7-CH_2_, 8-CH_2_), 1.37–1.48 (m, 1H, 7-CH_2_); ^13^C NMR (DMSO-*d*_6_, 126 MHz) δ 178.16, 178.15, 177.7, 176.7, 165.9, 143.3, 134.7, 131.6, 128.6, 127.0, 126.7, 56.5, 47.3, 46.1, 44.9, 44.5, 41.1, 26.3, 22.7, 19.6, 17.5; HRMS (ESI-TOF) *m*/*z* 420.1535 (calcd for C_23_H_22_N_3_O_5_ (M + H)^+^ 420.1554); Anal. C, 65.34; H, 4.95; N, 9.89 (calcd for C_23_H_21_N_3_O_5_ · 1/6 H_2_O C, 65.39; H, 5.09; N, 9.95).


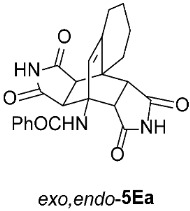


## 4. Conclusions

In this study, we described the first examples where the steric hindrance of the diene (i.e., *N*-substituted maleimides **2**) causes the double cycloaddition of 2*H*-pyran-2-one derivatives **1** to proceed via a different stereochemical pathway, yielding bicyclo[2.2.2]octenes with the asymmetric *exo*,*endo*-**5** structure instead of the symmetric *exo*,*exo*-**5** adducts obtained in all previous cases (with the only exception being when cyclooctane-fused pyran-2-one derivatives were applied). The work presented here clearly shows that steric interactions strong enough to reverse the stereoselectivity of these cycloadditions can arise solely from the bulkiness of the dienophiles **2**, which, together with other groups in the reactants, results in steric hindrance sufficient to yield nearly exclusively asymmetric *exo*,*endo* products. Based on the experimental results, a qualitative correlation between the steric demand of the substituents present in **2** and the observed ratio of the asymmetric *exo*,*endo*- to symmetric *exo*,*exo*-cycloadducts **5** was established, showing that greater steric hindrance leads to the formation of a larger amount of asymmetric *exo*,*endo*-**5** adducts, with stereospecific formation of *exo*,*endo*-**5** when the sterically highly congested *N*-triphenylmethylmaleimide (**2e**) is used. Furthermore, we have shown that asymmetric *exo*,*endo*-**5** with R^4^ = H, which cannot be accessed via direct cycloaddition of maleimide (**2a**) on **1A**–**E**, can be obtained by acid-induced elimination of the triphenylmethyl group from the asymmetric bicyclo[2.2.2]octenes *exo*,*endo*-**5A**–**Ee**, which are easily obtained by cycloaddition of *N*-triphenylmethylmaleimide (**2e**). In these cases, the *N*-triphenylmethyl group acts as a diastereoselective *exo*,*endo*-auxiliary.

## Figures and Tables

**Table 1:**
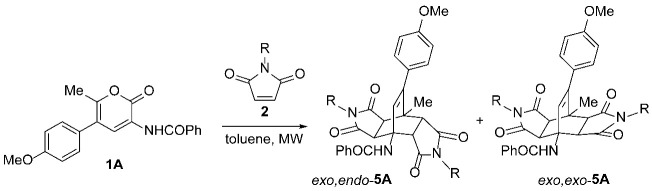
Steric effect of dienophile **2** on the stereochemical outcome of the double Diels–Alder reaction with **1A** ^a^.

Entry	Dienophile 2 (R)		Product 5A	*exo*,*endo*-5A : *exo*,*exo*-5A ^b,c^	Isolated Yields *exo*,*endo*-5A (*exo*,*exo*-5A) [%]
1	H	**2a**	**5Aa**	1 : 4.9 ^d^	— ^e^ (82)
2	4-*t*-Bu-C_6_H_4_-	**2b**	**5Ab**	1 : 3.6 ^d^	17 (65)
3	2-*t*-Bu-C_6_H_4_-	**2c**	**5Ac**	1 : 1.6 ^d^	23 (38)
4	*t*-Bu	**2d**	**5Ad**	1 : 1.1 ^d^	39 (43)
5	Ph_3_C-	**2e**	**5Ae**	1 : 0.04 ^d^	87 (—) ^e^

^a^ Reaction conditions: 2*H*-pyran-2-one **1A** (0.5 mmol) and maleimide **2** (1.1 mmol) in toluene (2.2 mL), MW (1 h, 180 °C, power set to 300 W). ^b^ Determined by ^1^H NMR spectroscopic analysis of the crude reaction mixture. ^c^ Conversion of starting **1A** was quantitative. ^d^ Accompanied by a minor amount (<5%) of isoindoles **6A**. ^e^ Not isolated.

**Table 2:**
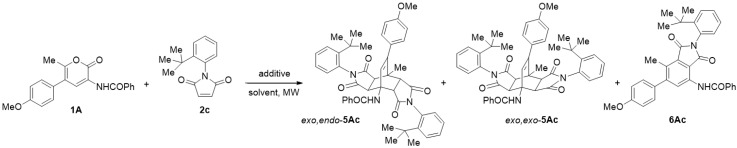
Optimization of reaction conditions ^a^.

Entry	Additive	Solvent	*T* [°C]	*t* [h]	Conversion of 1A [%] ^b^	Ratio of Products in Crude Reaction Mixture ^b^		
						*exo*,*endo*-5Ac	*exo*,*exo*-5Ac	6Ac
1	—	toluene	120	0.5	66	1	3.3	0.04
2	benzoic acid	toluene	120	0.5	62	1	3.7	0.06
3	ZnCl_2_	toluene	120	0.5	60	1	3.7	0.42
4	[emim]BF_4_	toluene	120	0.5	56	1	4.1	0.09
5	*N*,*N*′-diphenylthiourea	toluene	120	0.5	76	1	3.5	0.10
6	L-prolinol	toluene	120	0.5	65	1	3.8	0.58
7	L-proline	toluene	120	0.5	72	1	3.3	0.04
8	Et_3_N	toluene	120	0.5	72	1	2.6	5.8
9	quinine	toluene	120	0.5	70	1	2.9	5.7
10	cinchonine	toluene	120	0.5	66	1	2.9	8.3
11	—	toluene	120	2	89	1	2.6	0.08
12	—	toluene	140	2 ^c^	93	1	2.2	0.08
13	—	toluene	160	2 ^c^	99	1	2.0	0.09
14	—	toluene	180	1 ^d^	>99	1	1.6	0.06
15	—	xylene ^e^	180	1 ^d^	>99	1	1.7	0.44
16	—	anisole	180	1 ^d^	>99	1	2.4	0.25
17	—	*n*-heptane	180	1 ^d^	>99	1	2.4	0.04
18	—	3-pentanone	180	1 ^d^	>99	1	3.3	0.22
19	—	*n*-butanol	180	1 ^d^	99	1	3.3	0.10
20	—	isobutanol	180	1 ^d^	99	1	3.8	0.06
21	—	*n*-propanol	180	1 ^d^	97	1	4.1	0.16
22	—	H_2_O	180	1 ^d^	>99	1	6.1	0.03
23	—	MeCN	180	1 ^d^	98	1	6.4	1.7
24	—	1,4-dioxane	180	1 ^d^	79	1	3.7	1.1

^a^ Reaction conditions: 2*H*-pyran-2-one **1A** (0.5 mmol), maleimide **2c** (1.1 mmol), additive (0.1 mmol), solvent (2.2 mL), and MW (power set to 100 W). ^b^ Determined by ^1^H NMR spectroscopic analysis of the crude reaction mixture. ^c^ MW power set to 150 W. ^d^ MW power set to 300 W. ^e^ Mixture of isomers.

**Table 3:**
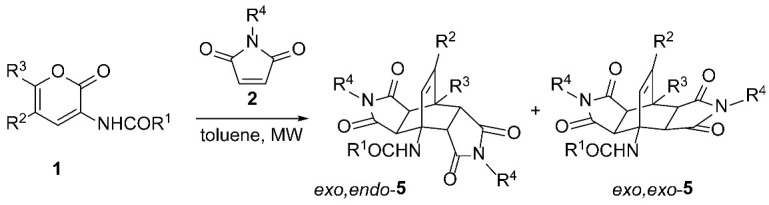
Products **5** prepared by the Diels–Alder reaction of substituted 2*H*-pyran-2-ones **1** and maleimides **2** ^a^.

Entry	2*H*-Pyran-2-ones 1			1	2	R^4^	*t* [h]	*T* [°C]	Product 5	*exo*,*endo*-5 : *exo*,*exo*-5 ^b,c^	Isolated Yields *exo*,*endo*-5 (*exo*,*exo*-5) [%]
	R^1^	R^2^	R^3^								
1	Ph	3,4-(MeO)_2_-C_6_H_3_-	Me	**1B**	**2a**	H	1	180	**5Ba**	1 : 4.9	— ^d^ (85)
2	Ph	3,4-(MeO)_2_-C_6_H_3_-	Me	**1B**	**2b**	4-*t*-Bu-C_6_H_4_-	1	180	**5Bb**	1 : 5.6	11 (70)
3	Ph	3,4-(MeO)_2_-C_6_H_3_-	Me	**1B**	**2c**	2-*t*-Bu-C_6_H_4_-	1	180	**5Bc**	1 : 2.5	19 (49)
4	Ph	3,4-(MeO)_2_-C_6_H_3_-	Me	**1B**	**2d**	*t*-Bu	1	180	**5Bd**	1 : 1.1	38 (44)
5	Ph	3,4-(MeO)_2_-C_6_H_3_-	Me	**1B**	**2e**	Ph_3_C-	1	180	**5Be**	1 : 0.05	85 (—) ^d^
6	Ph	COPh	Me	**1C**	**2a**	H	2	160	**5Ca**	1 : 4.0	— ^d^ (75)
7	Ph	COPh	Me	**1C**	**2e**	Ph_3_C-	4	140	**5Ce**	1 : 0	79 (—) ^d^
8	Ph	CO_2_Et	Me	**1D**	**2a**	H	2	180	**5Da**	1 : 8.4	— ^d^ (86)
9	Ph	CO_2_Et	Me	**1D**	**2e**	Ph_3_C-	1	180	**5De**	1 : 0	81 (—) ^d^
10	Ph	-[CH_2_]_4_-		**1E**	**2a**	H	2	160	**5Ea**	1 : 4.1	— ^d^ (80)
11	Ph	-[CH_2_]_4_-		**1E**	**2e**	Ph_3_C-	2	160	**5Ee**	1 : 0.1	70 (—) ^d^
12	Ph	COMe	Me	**1F**	**2e**	Ph_3_C-	1	180	**5Fe**	1 : 0	75 (—) ^d^
13	Ph	CH_2_CO_2_Me	CO_2_Me	**1G**	**2e**	Ph_3_C-	3	160	**5Ge**	1 : 0	72 (—) ^d^
14	Ph	-[CH_2_]_5_-		**1H**	**2e**	Ph_3_C-	1	180	**5He**	1 : 0	77 (—) ^d^
15	Ph	-[CH_2_]_3_-		**1I**	**2e**	Ph_3_C-	1	180	**5Ie**	1 : 0.4	55 (—) ^d^
16	Ph	Ph	Ph	**1J**	**2e**	Ph_3_C-	5	160	**5Je**	1 : 0.3	56 (—) ^d^
17	Me	4-MeO-C_6_H_4_-	Me	**1K**	**2e**	Ph_3_C-	2	160	**5Ke**	1 : 0.1	77 (—) ^d^

^a^ Reaction conditions: 2*H*-pyran-2-one **1** (0.5 mmol) and maleimide **2** (1.1 mmol), toluene (2.2 mL), and MW (power set to 300 W). ^b^ Determined by ^1^H NMR spectroscopic analysis of the crude reaction mixture. ^c^ Conversion of starting **1** was quantitative. ^d^ Not isolated.

**Table 4:**
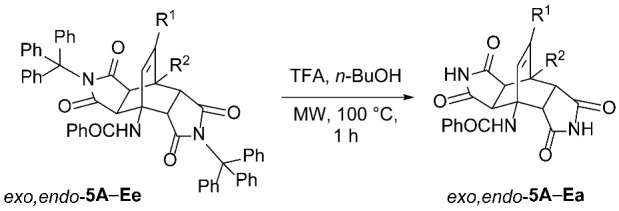
Preparation of *exo*,*endo*-**5A**–**Ea** via removal of *N*-triphenylmethyl groups from *exo*,*endo*-**5A**–**Ee** ^a^.

Entry	Bicyclo[2.2.2]Octene *exo*,*endo*-5A–Ee			*T* (°C)	Product *exo*,*endo*-5A–Ea ^b^	Isolated Yield [%]
	R^1^	R^2^				
1	4-MeO-C_6_H_4_-	Me	**5Ae**	100	**5Aa**	85
2	3,4-(MeO)_2_-C_6_H_3_-	Me	**5Be**	100	**5Ba**	87
3	COPh	Me	**5Ce**	100	**5Ca**	93
4	CO_2_Et	Me	**5De**	140 ^c^	**5Da**	88
5	-[CH_2_]_4_-		**5Ee**	140	**5Ea**	91

^a^ Reaction conditions: bicyclo[2.2.2]octene *exo*,*endo*-**5A**–**Ee** (0.33 mmol), *n*-BuOH (3 mL), TFA (1 mL), and MW (power set to 100 W, 1 h). ^b^ Conversion of starting *exo*,*endo*-**5A**–**Ee** was quantitative. ^c^ Reaction was conducted in EtOH; the amount of TFA added was reduced to 0.3 mL.

## Data Availability

The raw data supporting the conclusions of this article will be made available by the authors on request.

## Supplementary Materials

The following supporting information can be downloaded at: https://www.mdpi.com/article/10.3390/molecules31081301/s1, ^1^H and ^13^C NMR spectra of all new products **1** and **5**, a representative example of ^1^H–^13^C *gs*-HSQC and ^1^H–^13^C *gs*-HMBC 2D NMR spectra for the asymmetric/symmetric pair **5Aa**, and the ^1^H NMR spectrum of isolated **6Ac**.
