# Supplementary material for: Triphenylmethyl Group as a Highly Diastereoselective exo,endo-Auxiliary in Double Diels–Alder Reactions with 2H-Pyran-2-ones
Source: Molecules. 2026 Apr 16;31(8):1301. doi: 10.3390/molecules31081301 (PMC13118795; doi:10.3390/molecules31081301)
Supplement: Supplementary file 1 [file molecules-31-01301-s001.zip › molecules-4254369-supplementary.pdf]

Triphenylmethyl Group as a Highly Diastereoselective *exo,endo*-Auxiliary in Double Diels–Alder Reactions with 2*H*-Pyran-2-ones

Marko Krivec, Žiga Štirn, Marijan Kočevar † and Krištof Kranjc *

Faculty of Chemistry and Chemical Technology, University of Ljubljana, Večna pot 113, SI-1000 Ljubljana, Slovenia; marko.krivec@fkkt.uni-lj.si (M.K.); ziga.stirn@gmail.com (Ž.Š.); marijan.kocevar@fkkt.uni-lj.si (M.K.)

* Correspondence: kristof.kranjc@fkkt.uni-lj.si; Tel.: +386-(0)1-479-8563

† Retired.

Supplementary Materials

^{1}H and ^{13}C NMR spectra of products **1** and **5** S2

Representative example of ^{1}H–^{13}C *gs*-HSQC and ^{1}H–^{13}C *gs*-HMBC 2D NMR spectra for the asymmetric/symmetric pair **5Aa** S83

^{1}H NMR spectrum of isolated **6Ac** S87

Academic Editor(s): Andrea Penoni

Received: 27 March 2026

Revised: 14 April 2026

Accepted: 15 April 2026

Published: date

Copyright: © 2026 by the authors. Submitted for possible open access publication under the terms and conditions of the Creative Commons Attribution (CC BY) license.

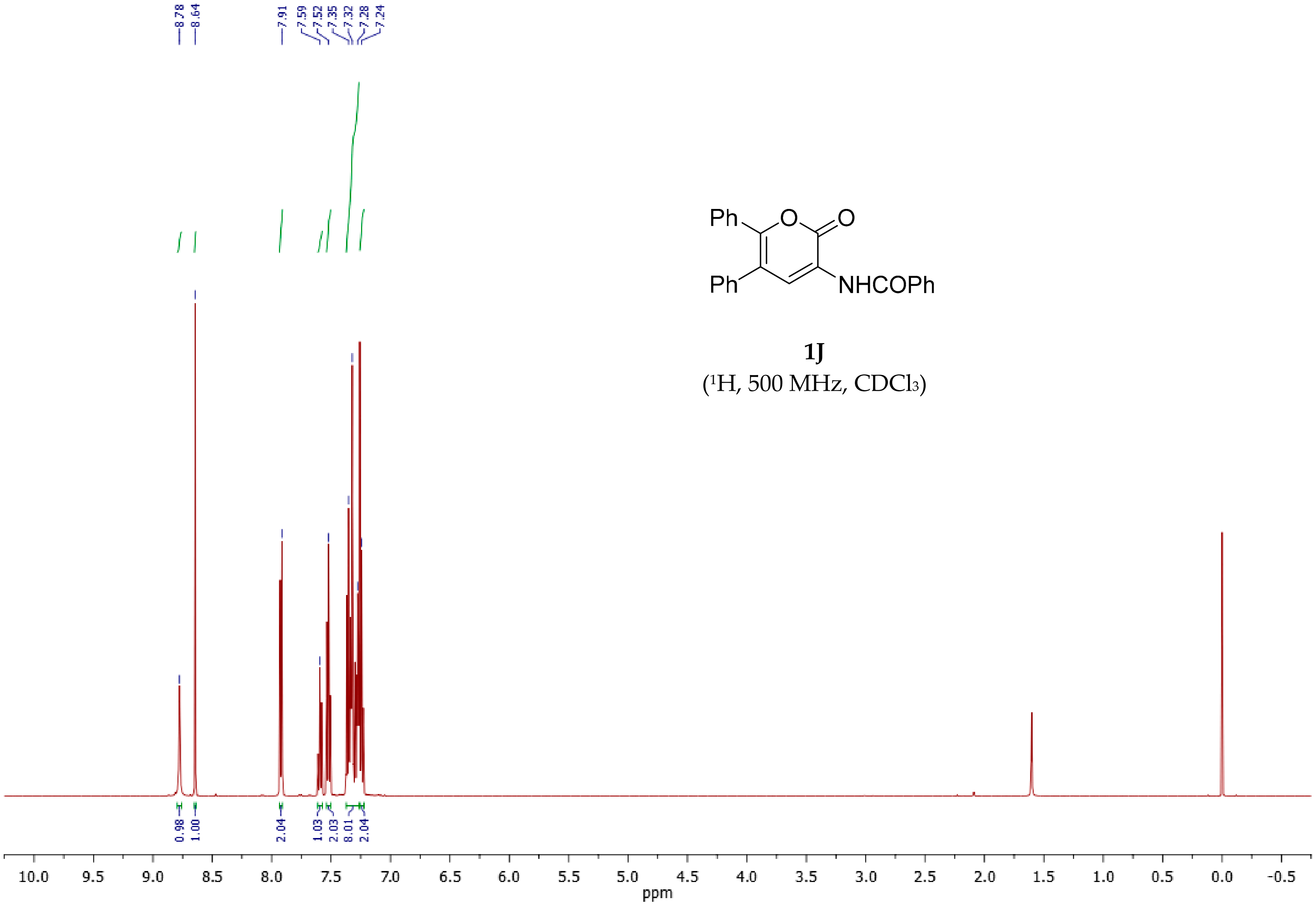

1J

(^{1}H, 500 MHz, $CDCl_3$)

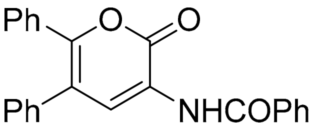

1J

(^{13}C, 126 MHz, $CDCl_3$)

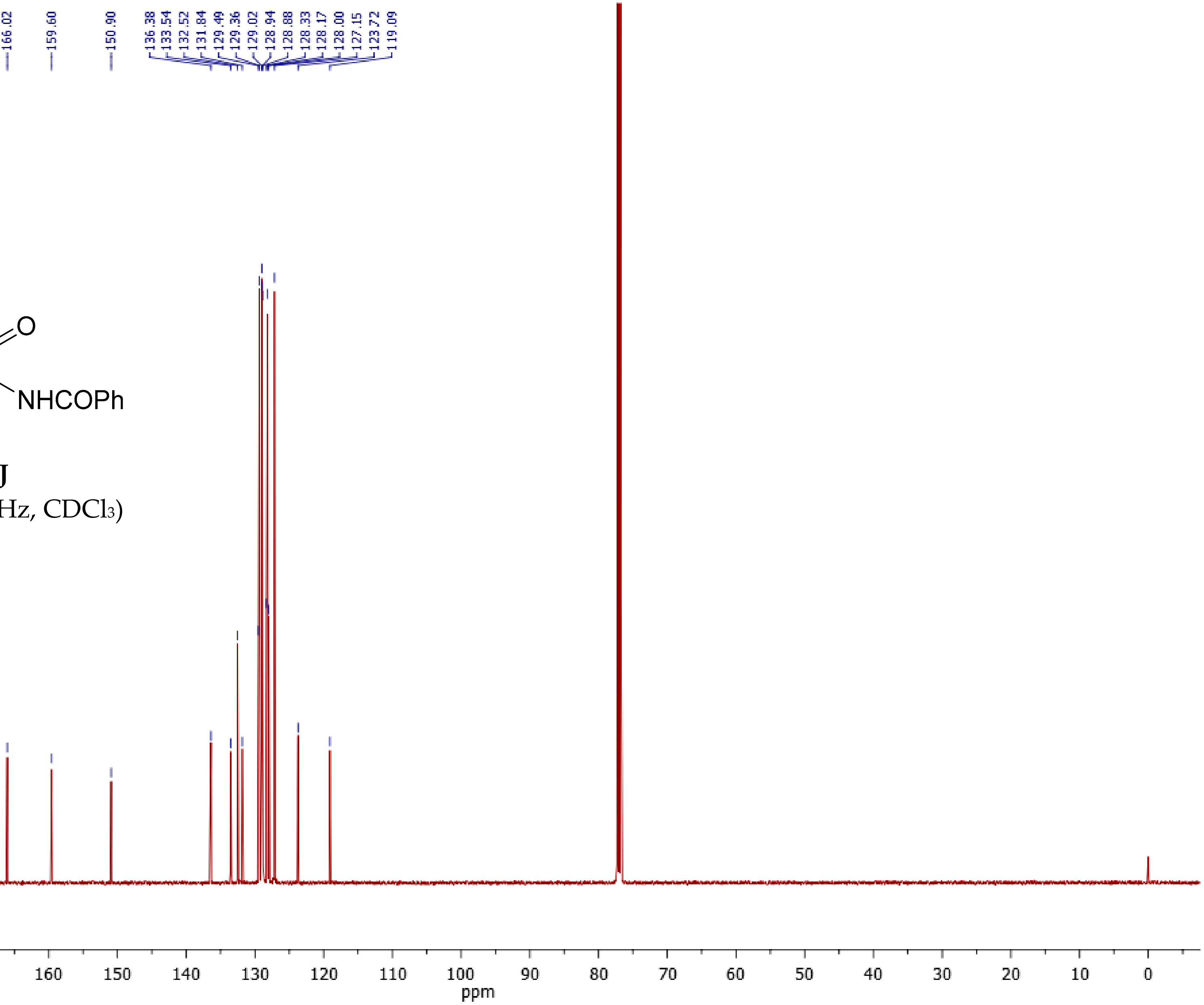

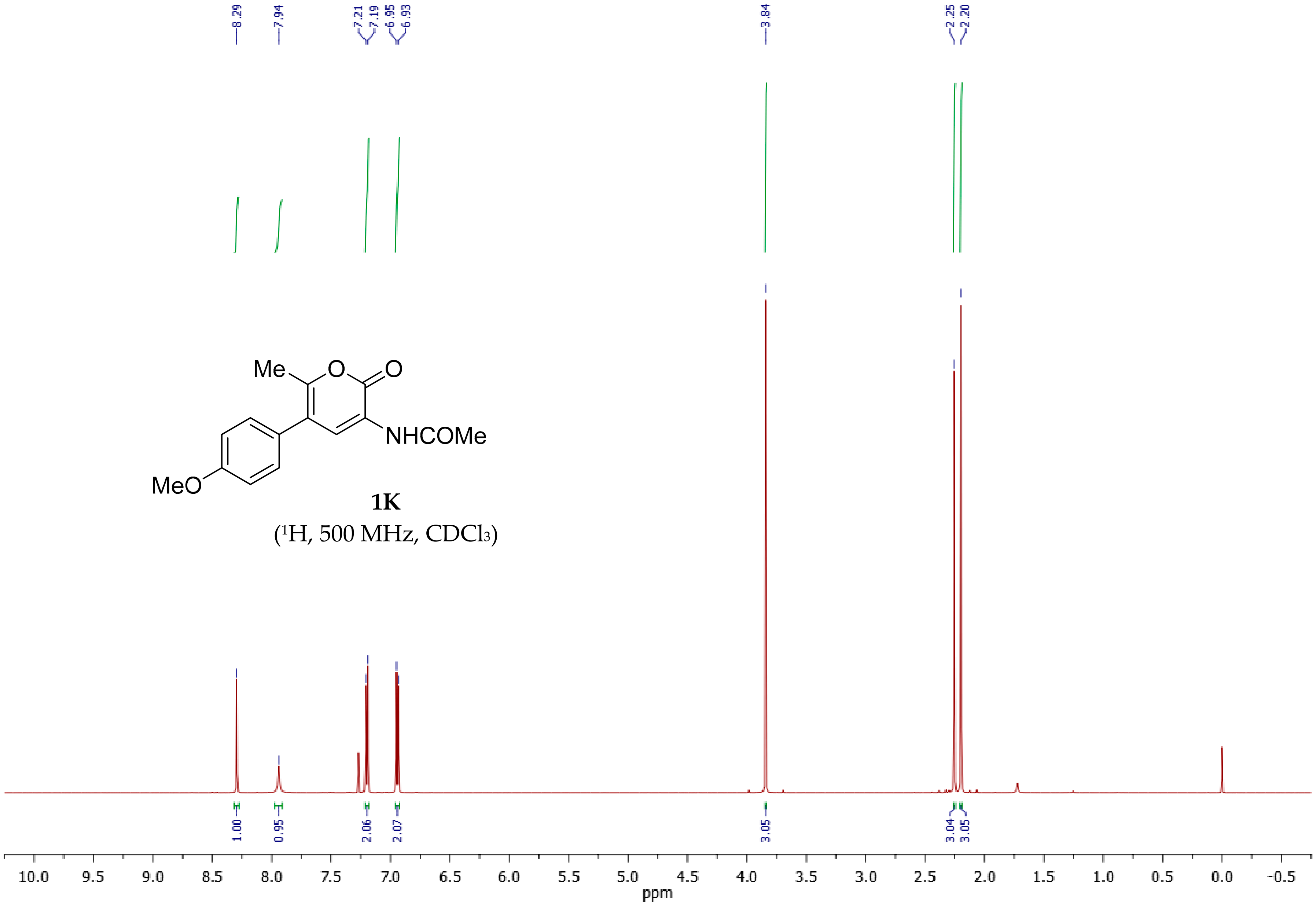

1K

(^{1}H, 500 MHz, $CDCl_3$)

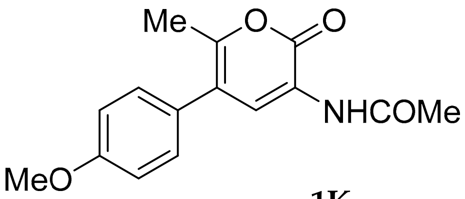

1K

(^{13}C, 126 MHz, $CDCl_3$)

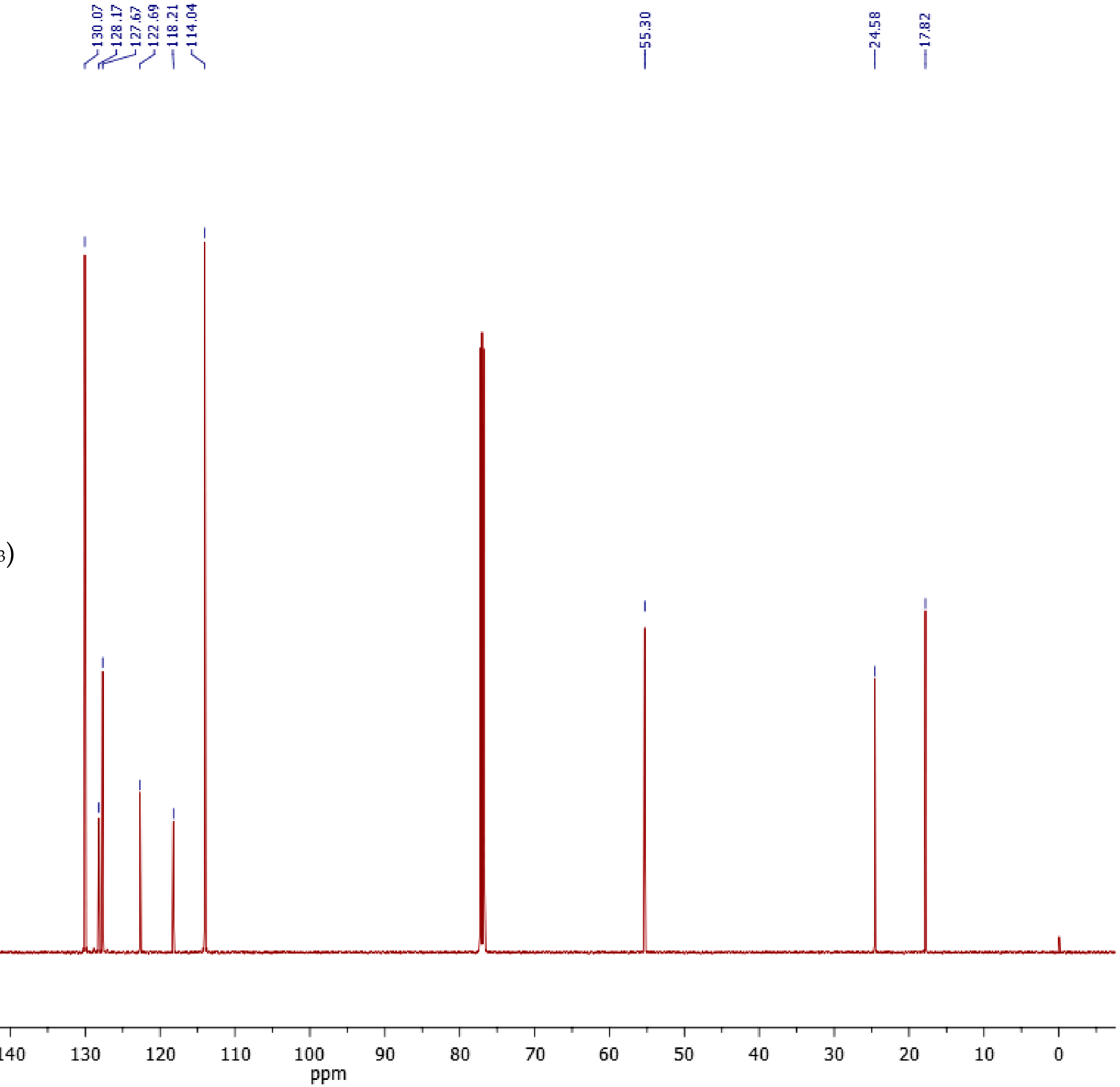

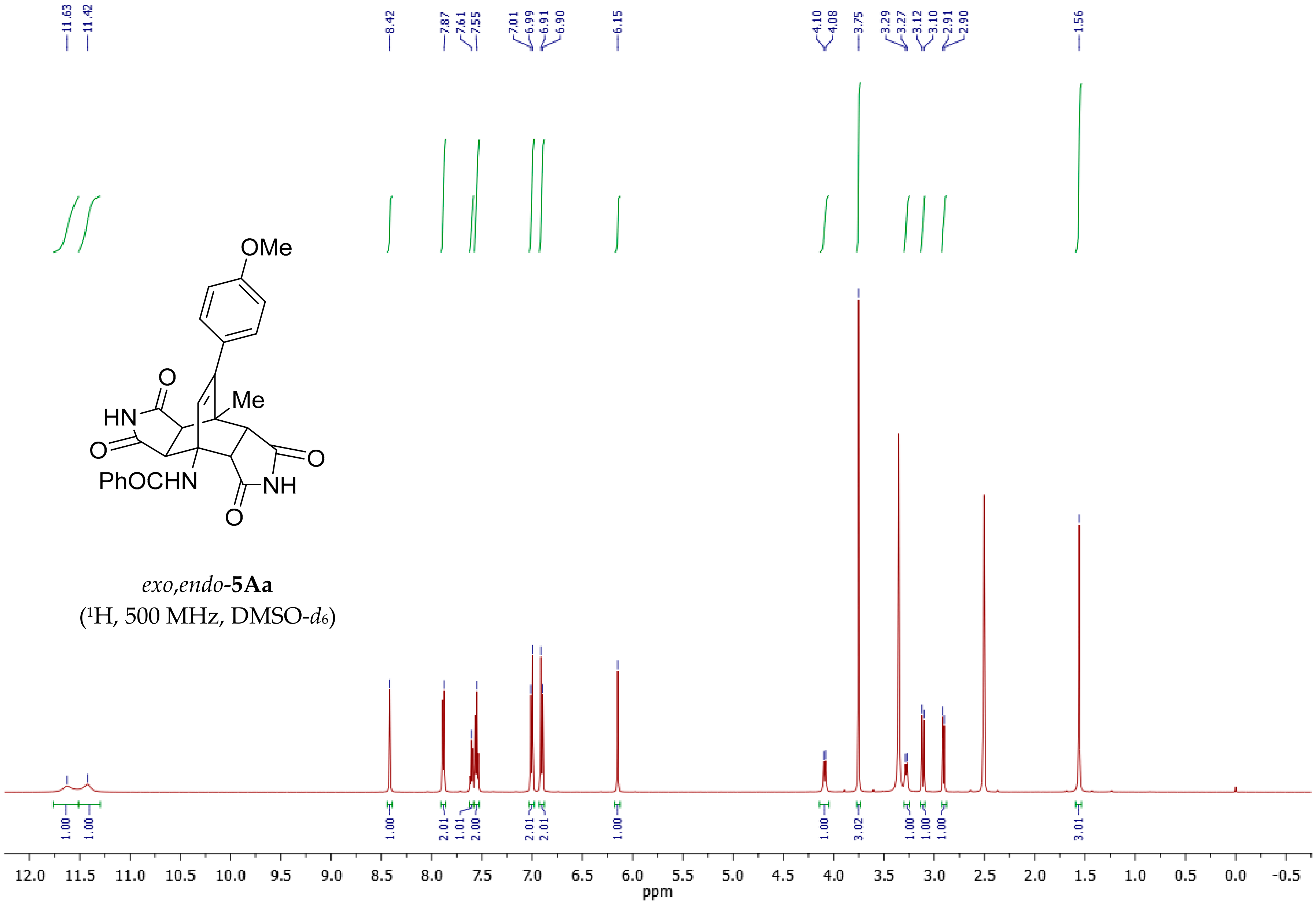

OMe
Me
HN
O
O
PhOCHN
NH
O
O
exo,endo-5Aa
(1H, 500 MHz, DMSO-d6)
11.63
11.42
8.42
7.87
7.61
7.55
7.01
6.99
6.91
6.90
6.15
4.10
4.08
3.75
3.29
3.27
3.12
3.10
2.91
2.90
1.56
1.00
1.00
1.00
2.01
1.01
2.00
2.01
2.01
1.00
1.00
3.02
1.00
1.00
1.00
3.01
12.0
11.5
11.0
10.5
10.0
9.5
9.0
8.5
8.0
7.5
7.0
6.5
6.0
5.5
5.0
4.5
4.0
3.5
3.0
2.5
2.0
1.5
1.0
0.5
0.0
-0.5
ppm

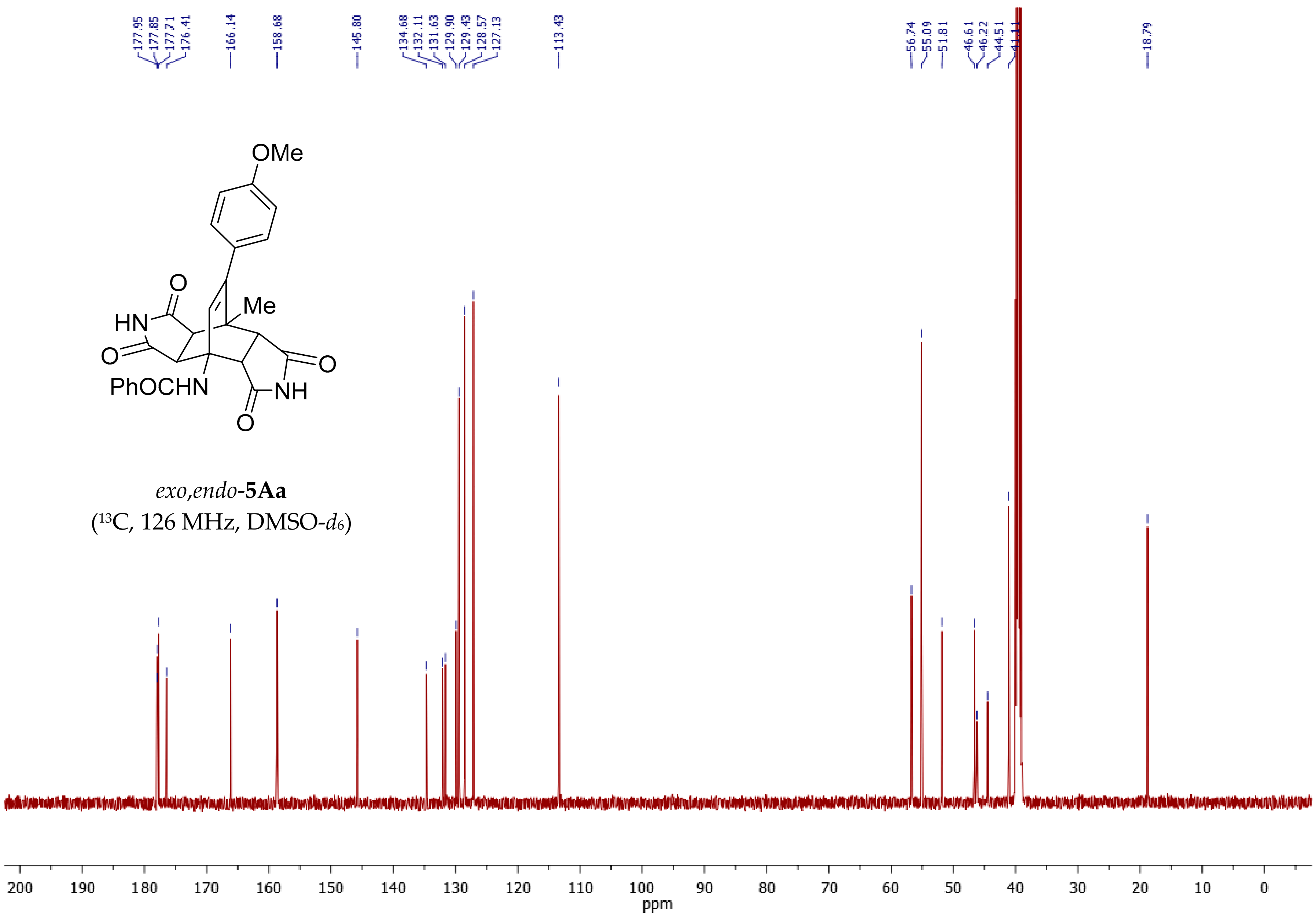

exo,endo-**5Aa**

(^{13}C, 126 MHz, DMSO-d_6)

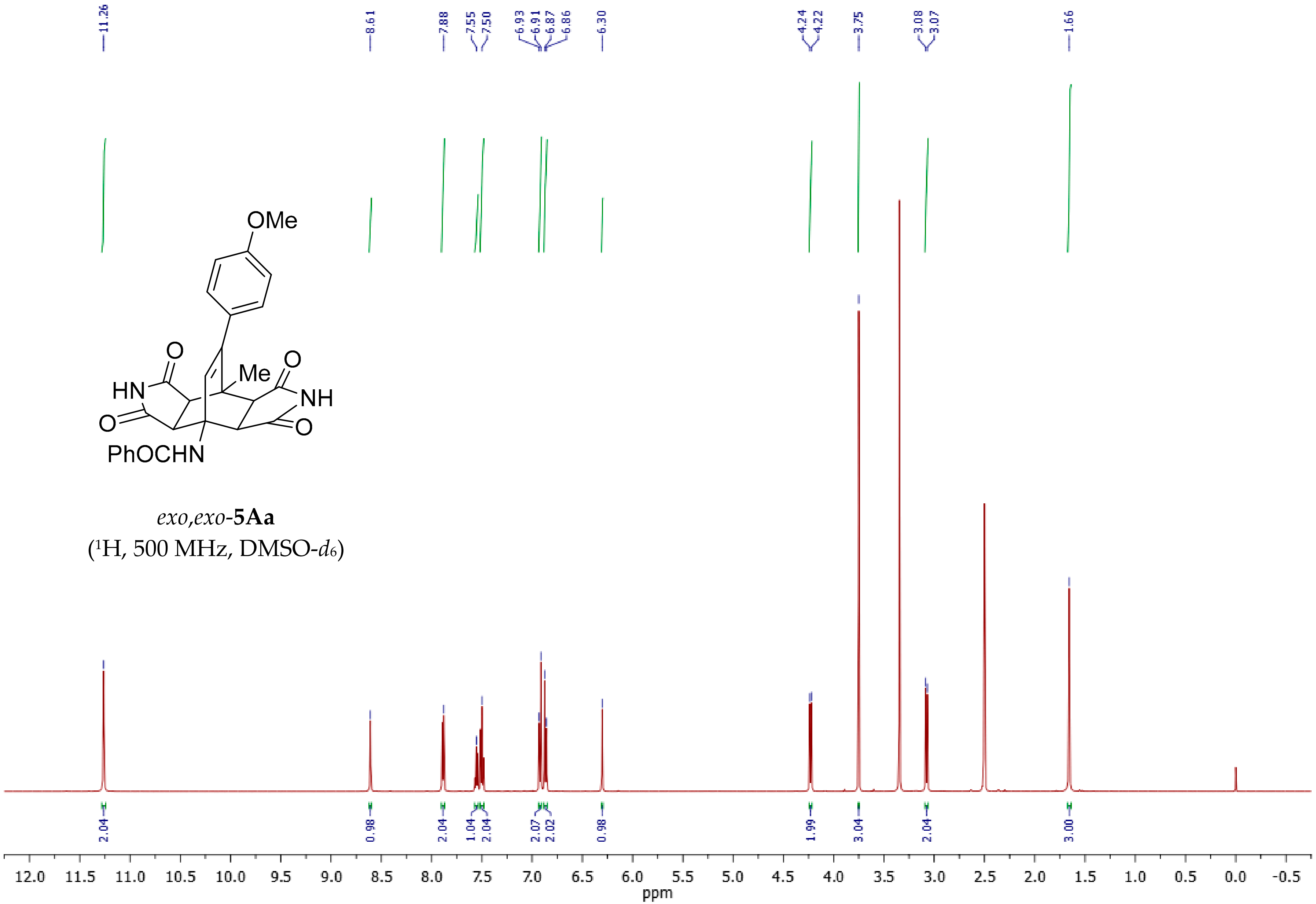

exo,exo-**5Aa**
(^{1}H, 500 MHz, DMSO-*d*6)

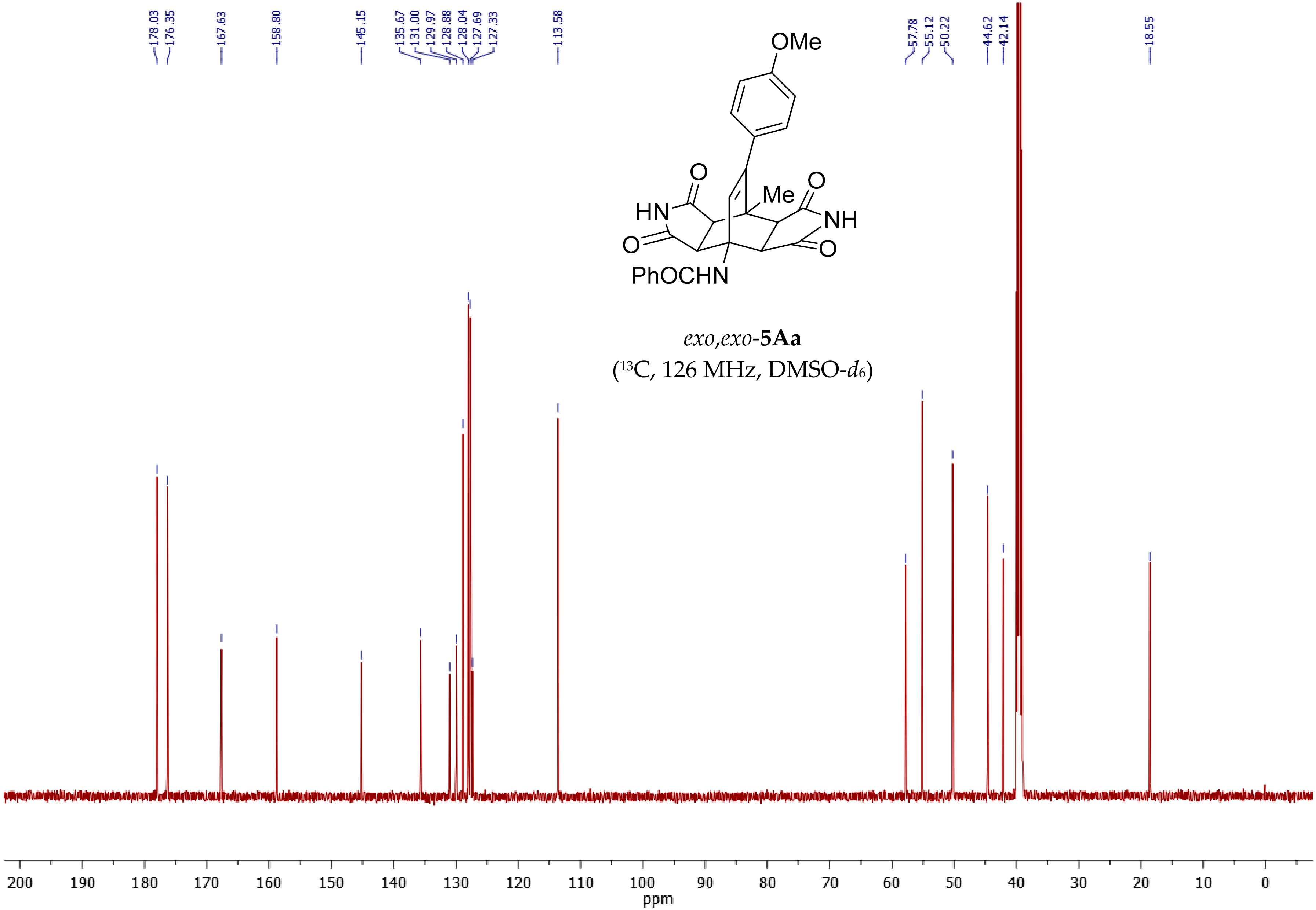

exo,exo-**5Aa**

(^{13}C, 126 MHz, DMSO-d_6)

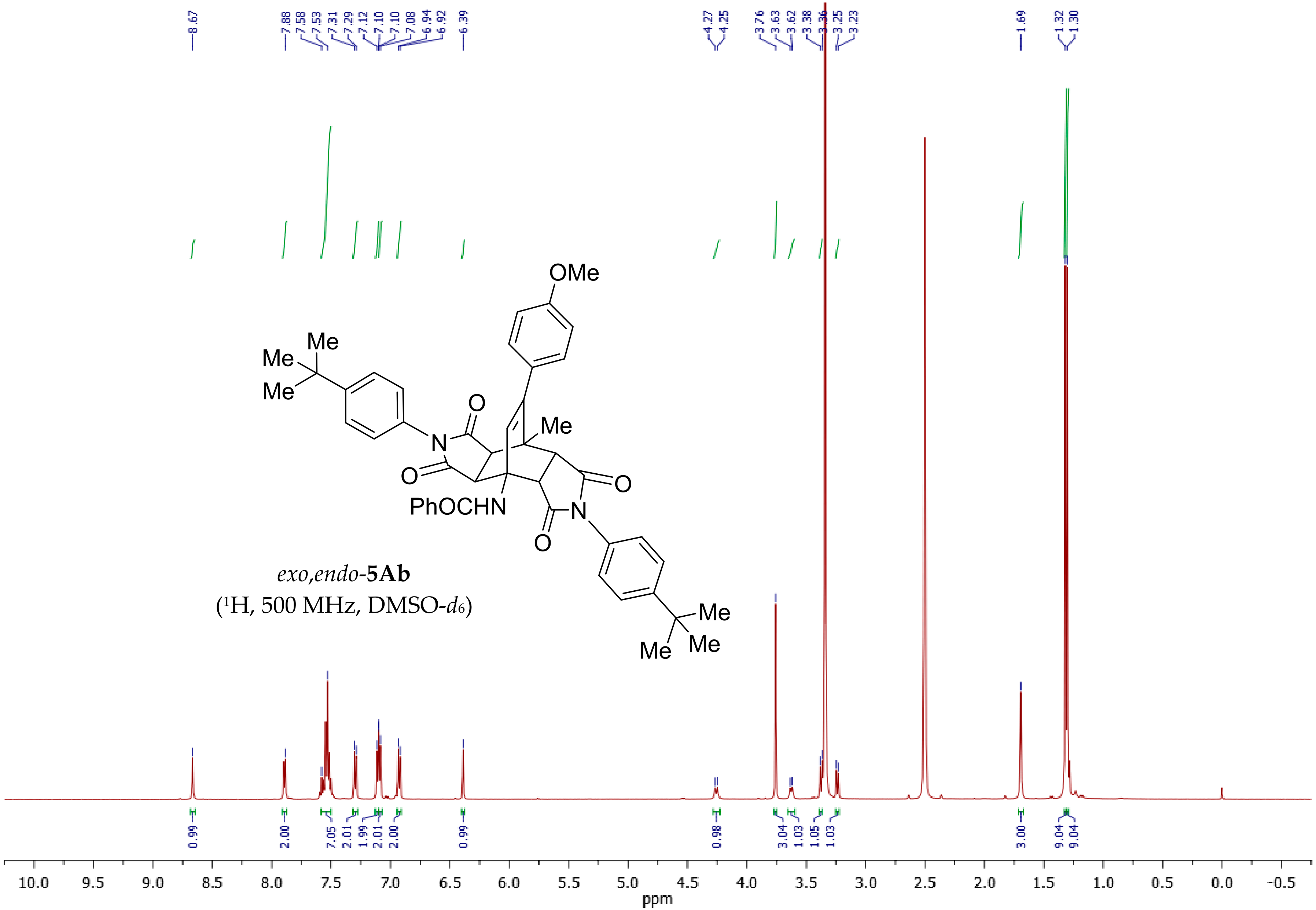

exo,endo-**5Ab**

(^{1}H, 500 MHz, DMSO-d_6)

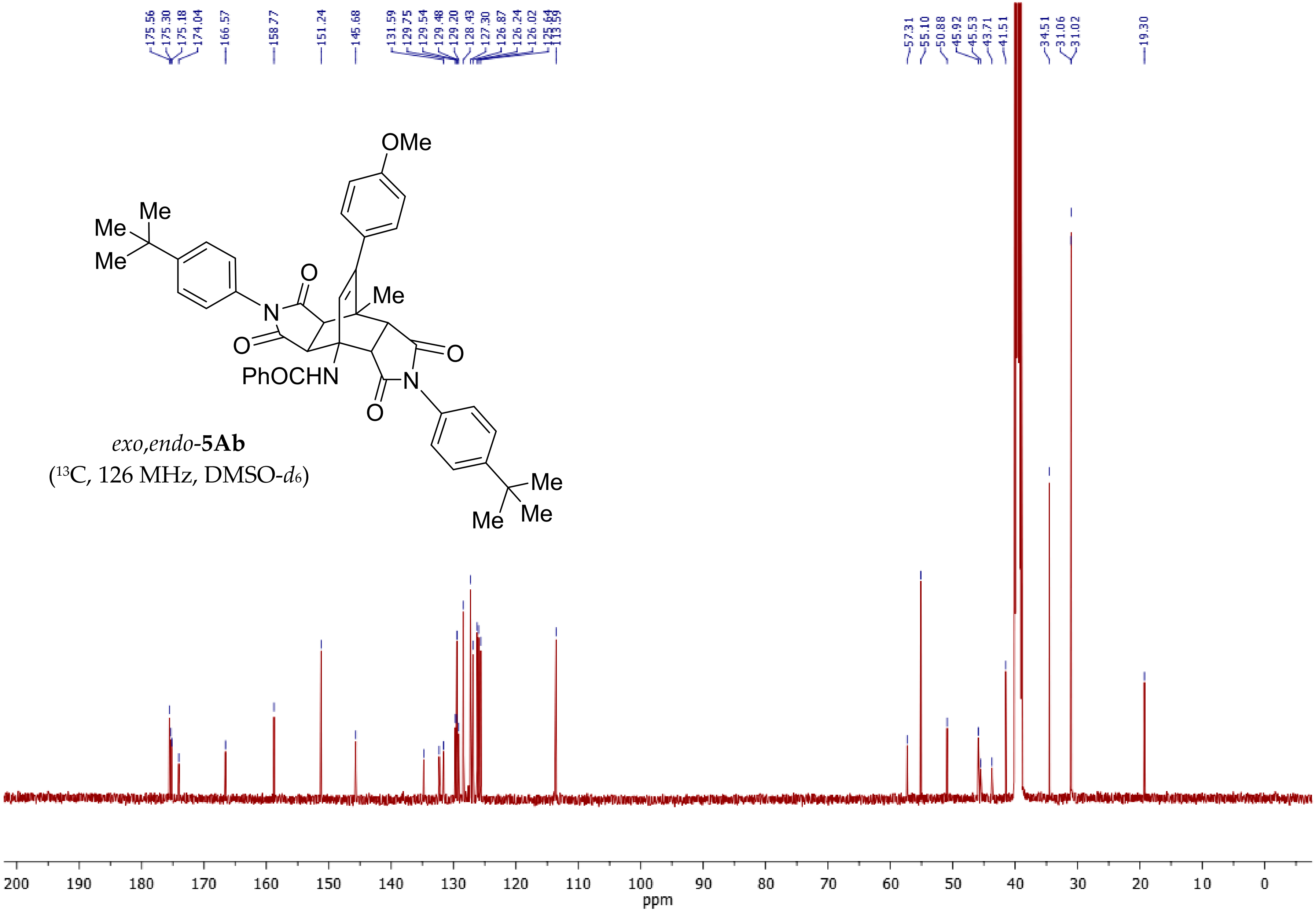

exo,endo-**5Ab**
(^{13}C, 126 MHz, DMSO-d_6)

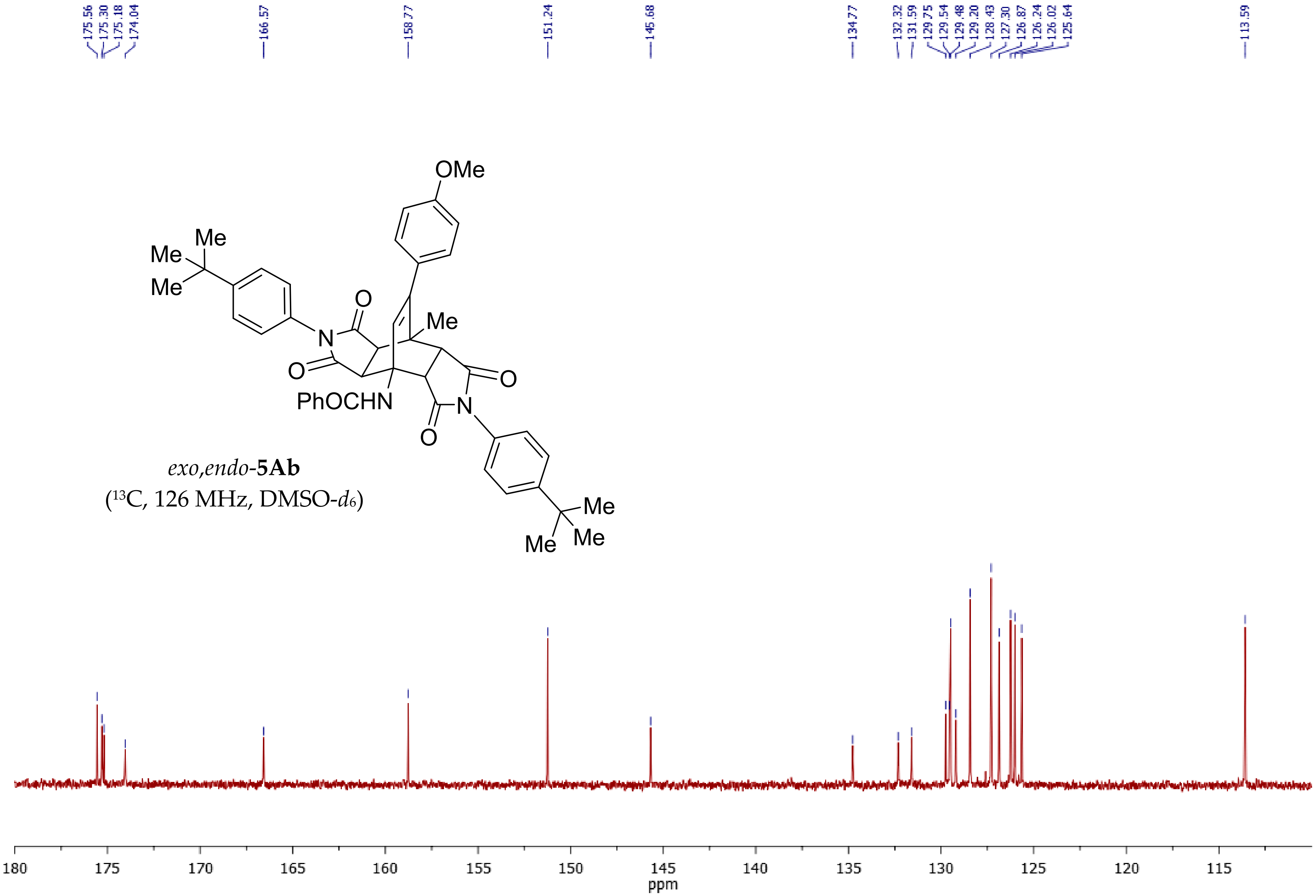

exo,*endo*-**5Ab**
(^{13}C, 126 MHz, DMSO-d_6)

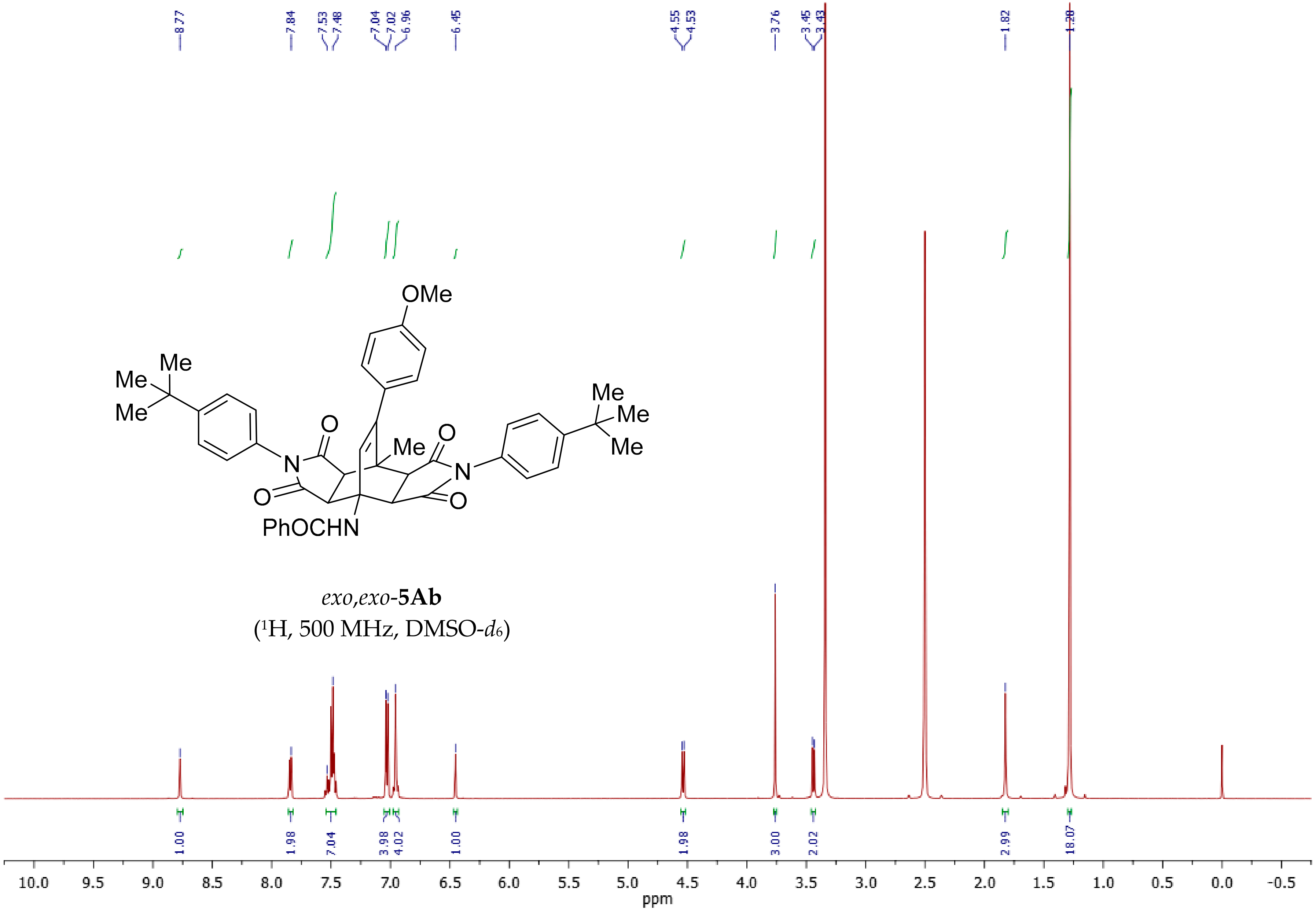

exo,*exo*-**5Ab**
(^{1}H, 500 MHz, DMSO-d_6)

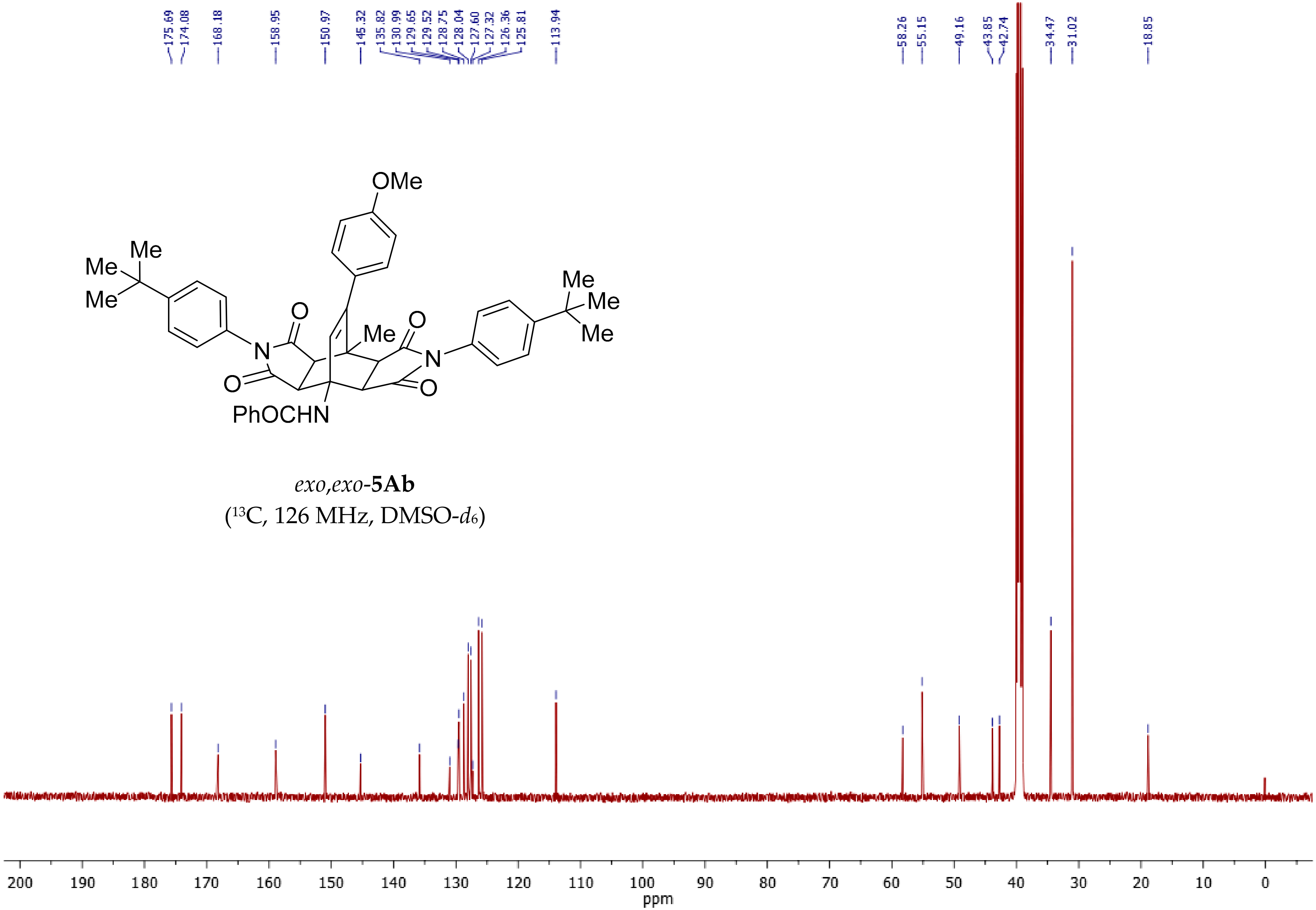

exo,*exo*-**5Ab**

(^{13}C, 126 MHz, DMSO-d_6)

exo,endo-**5Ac**
(^{1}H, 500 MHz, $CDCl_3$)

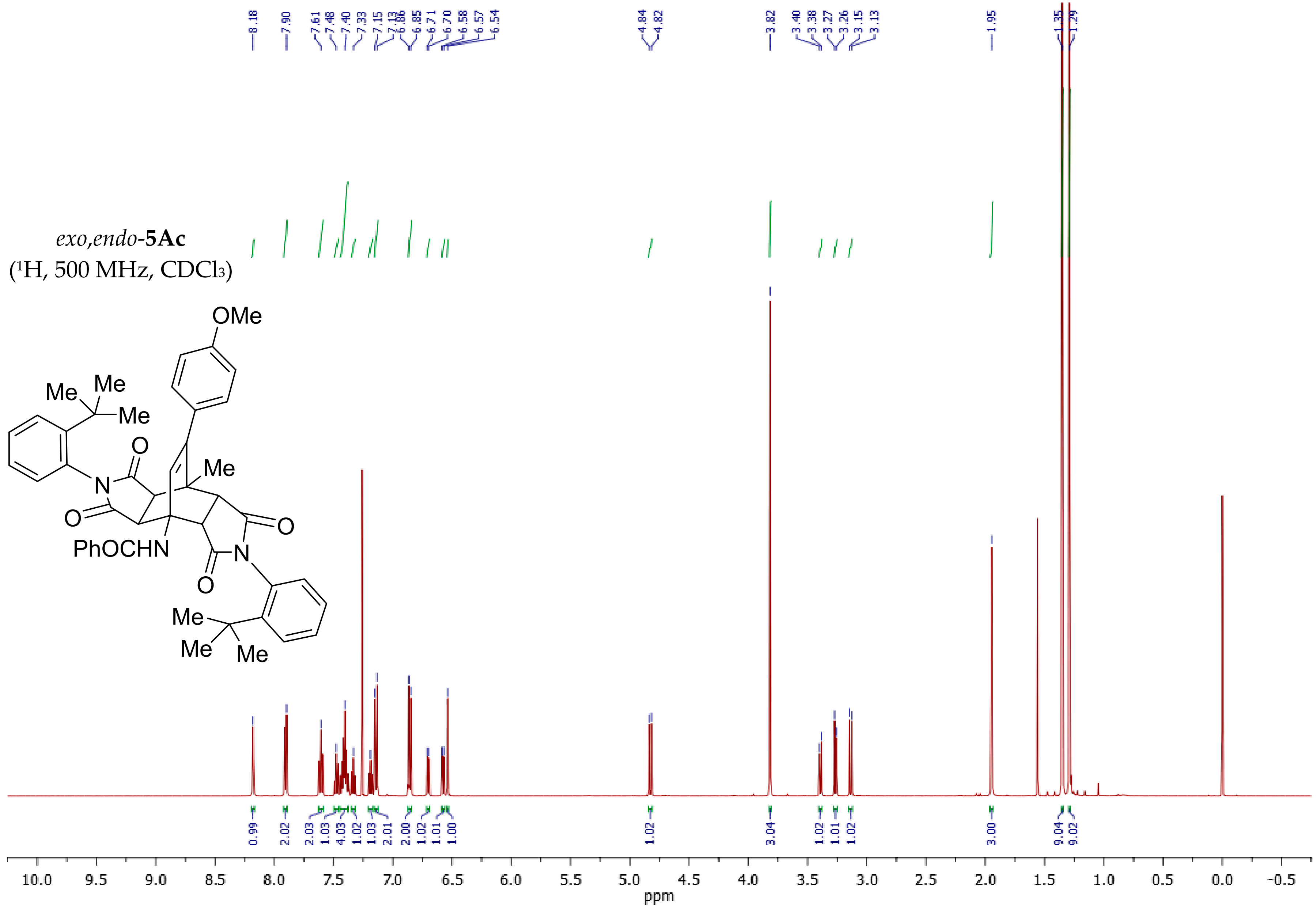

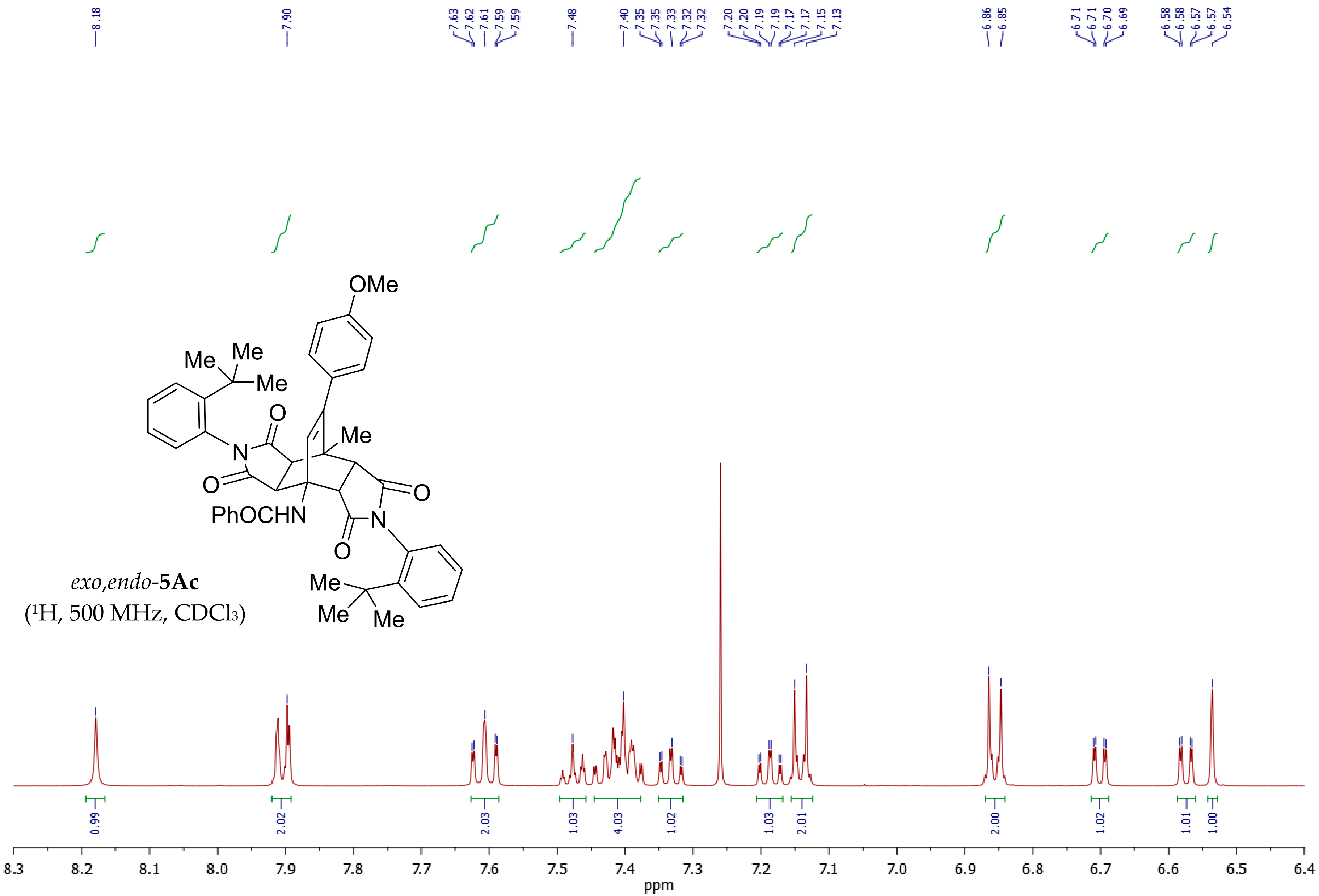

exo,endo-**5Ac**

(^{1}H, 500 MHz, $CDCl_3$)

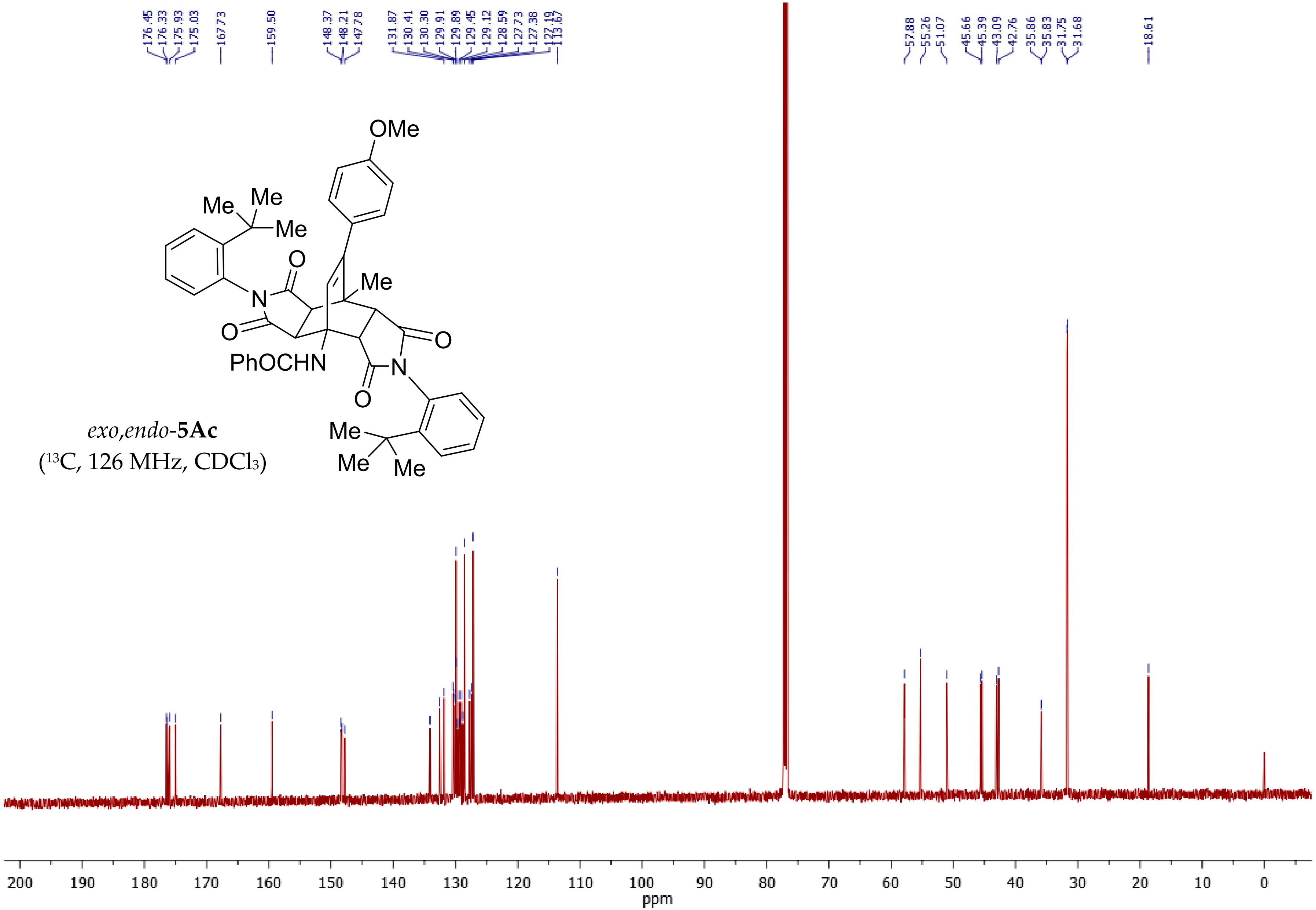

exo,endo-**5Ac**
(^{13}C, 126 MHz, $CDCl_3$)

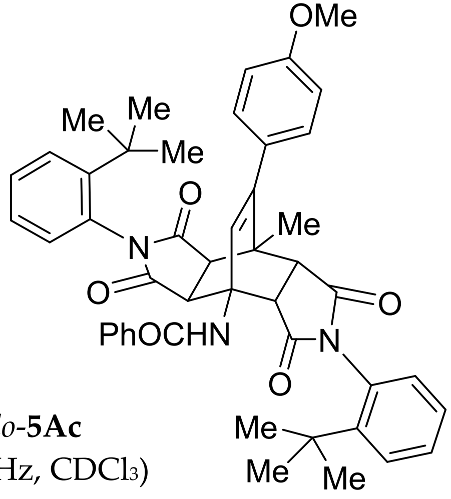

exo,endo-**5Ac**
(^{13}C, 126 MHz, $CDCl_3$)

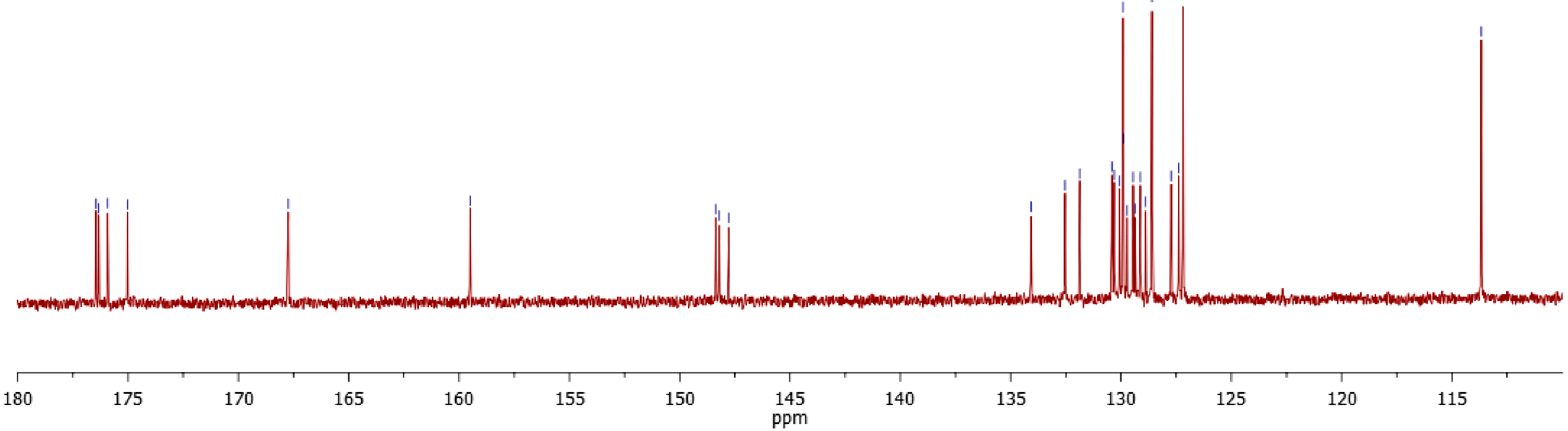

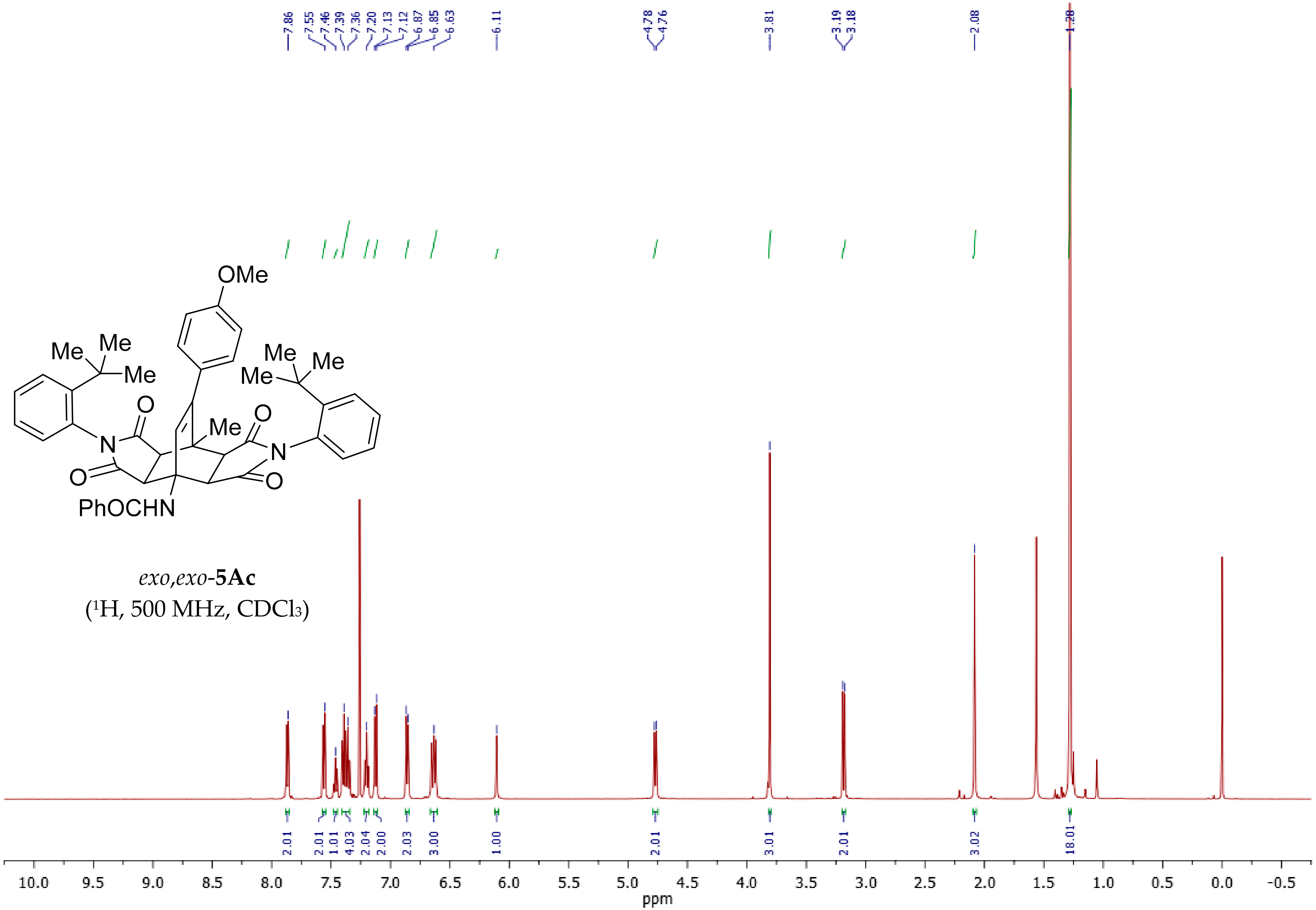

exo,exo-**5Ac**

(^{1}H, 500 MHz, $CDCl_3$)

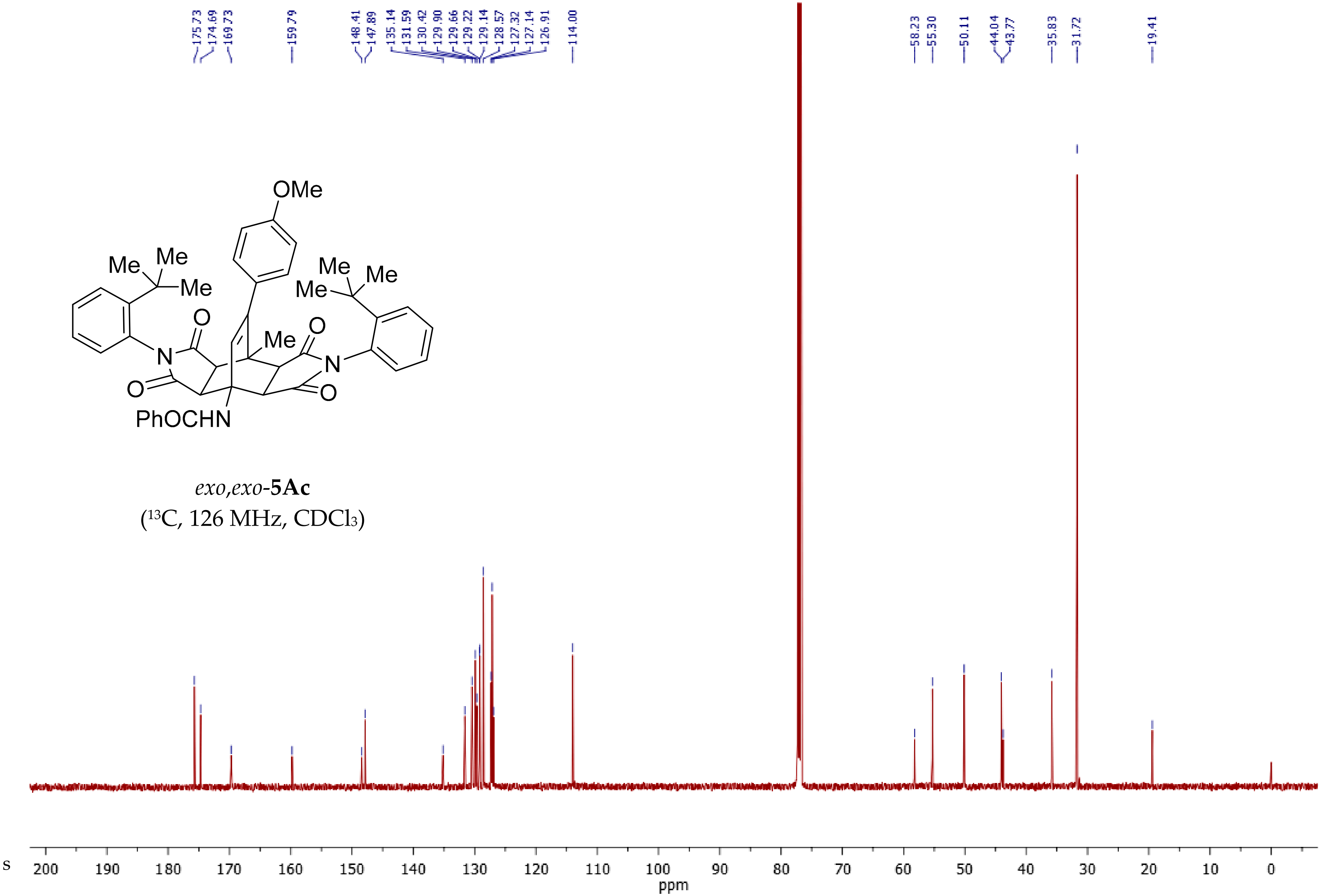

exo,exo-**5Ac**
(^{13}C, 126 MHz, $CDCl_3$)

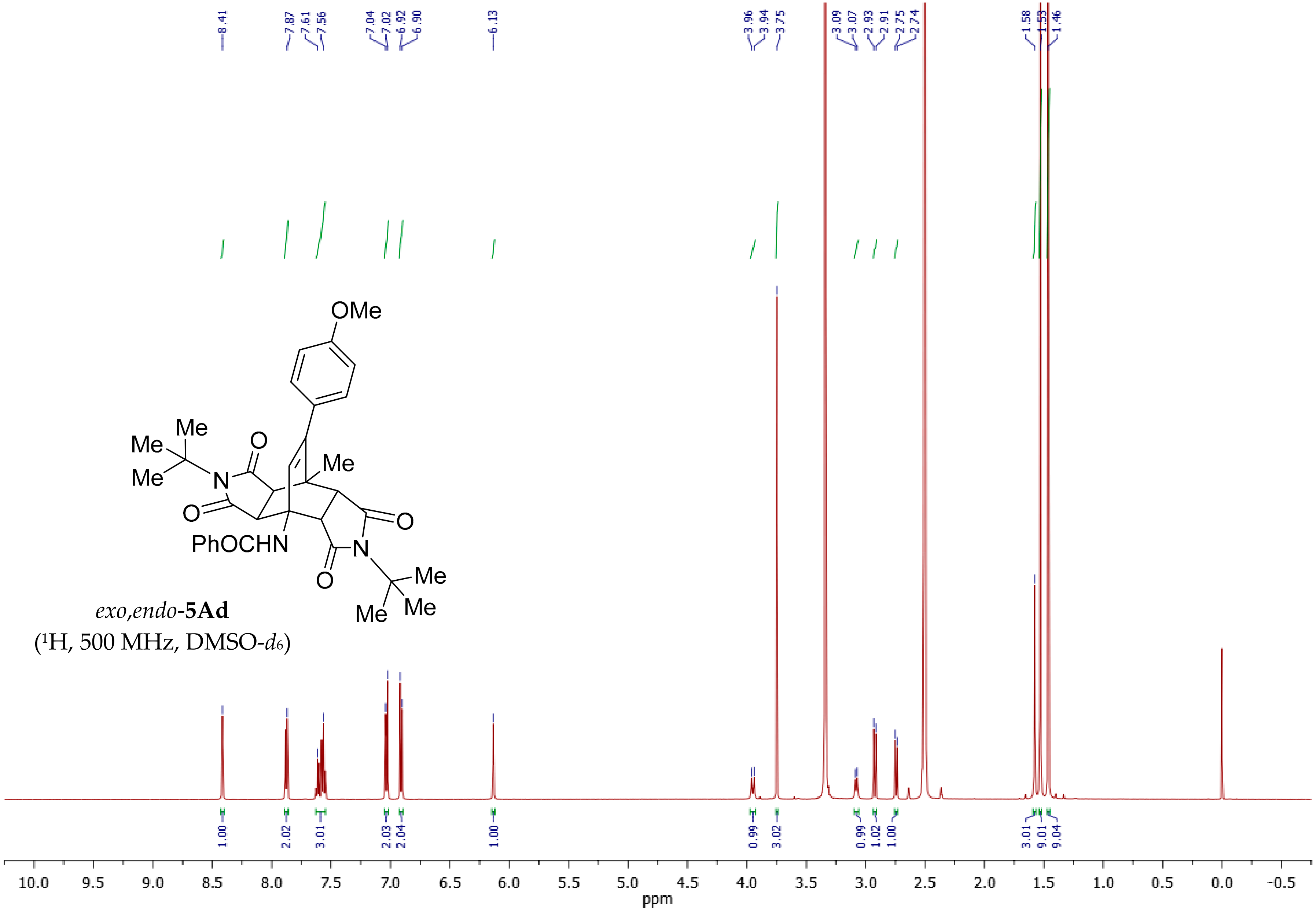

exo,endo-**5Ad**

(^{1}H, 500 MHz, DMSO-d_6)

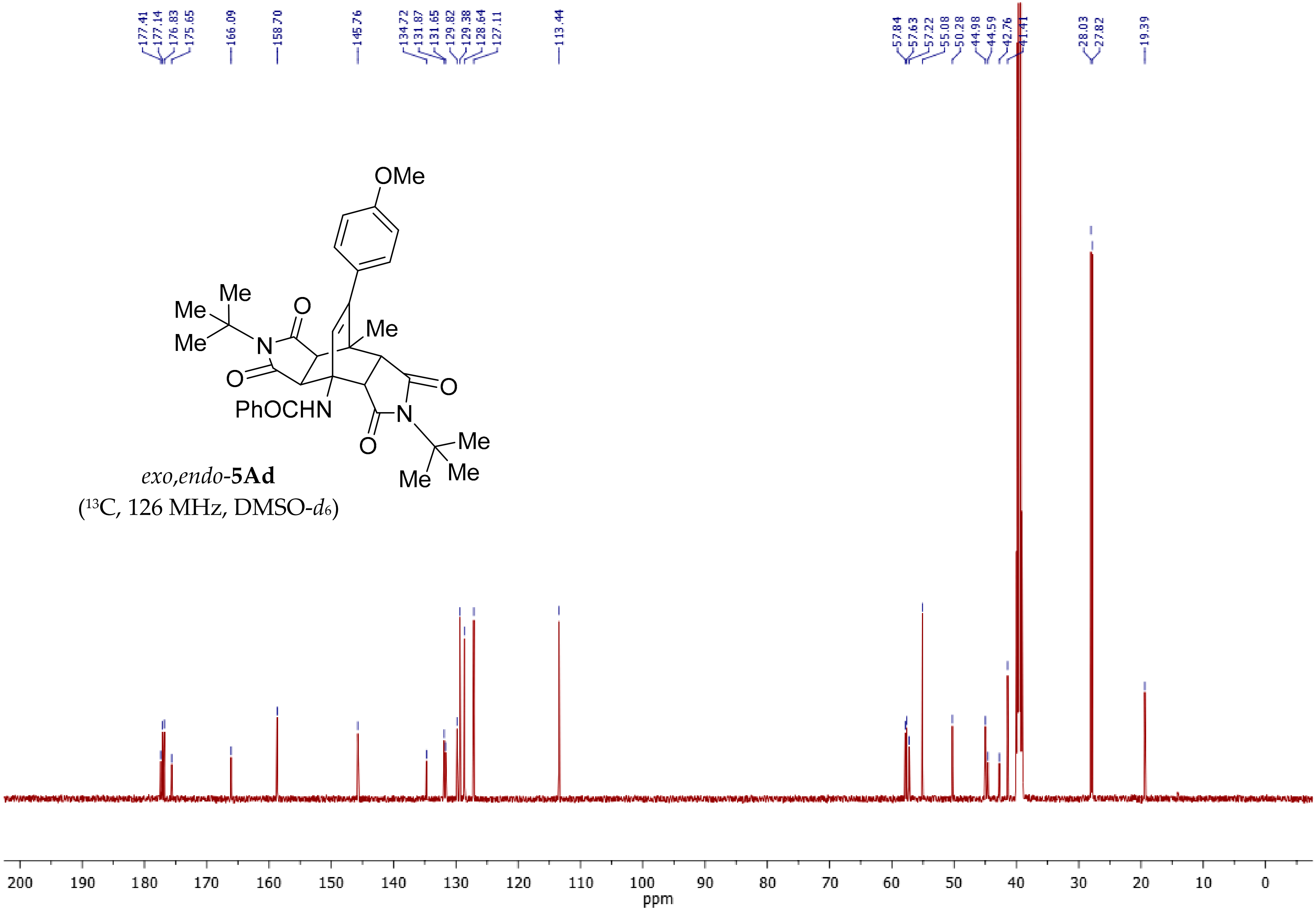

exo,endo-**5Ad**
(^{13}C, 126 MHz, DMSO-d_6)

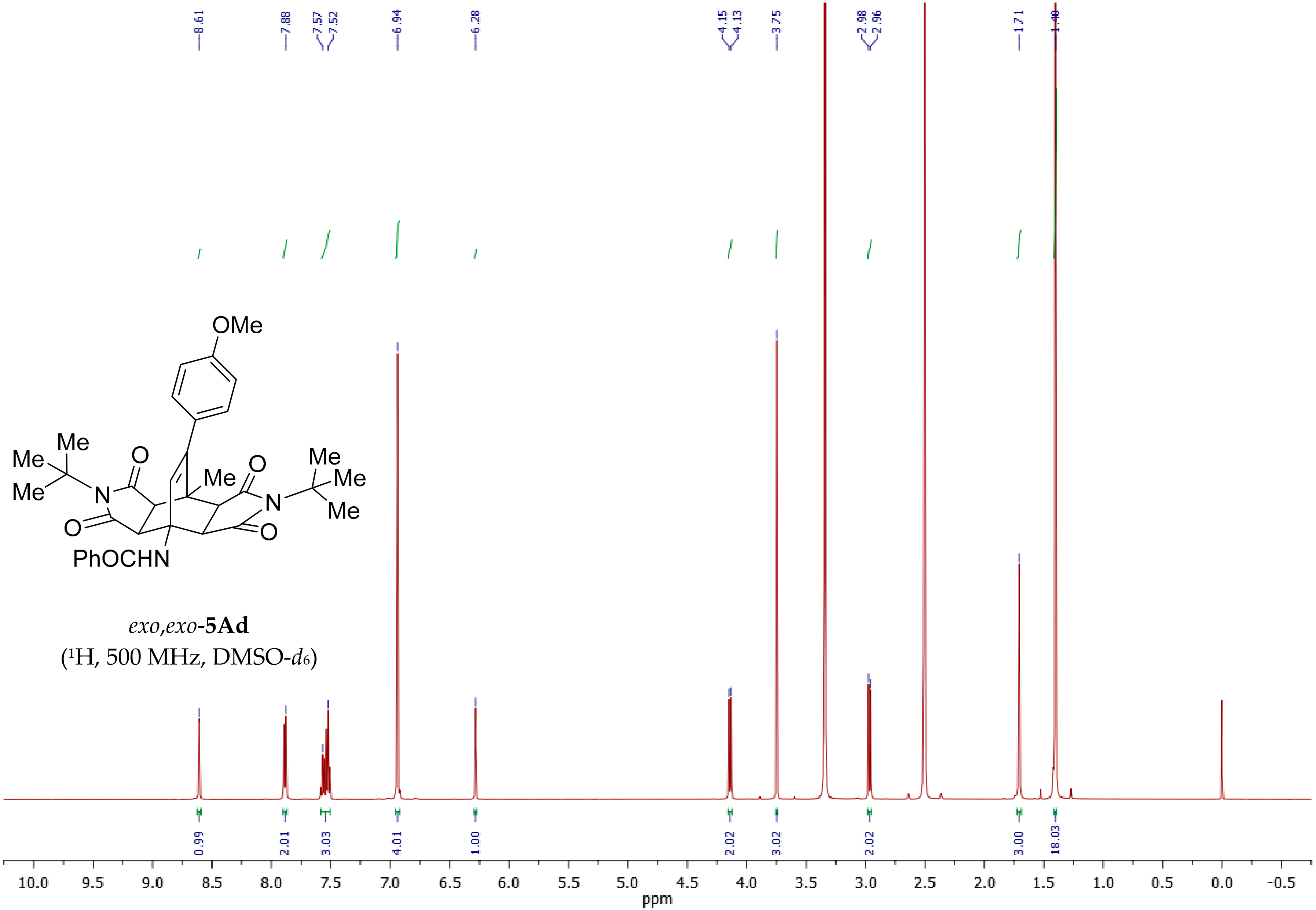

exo,*exo*-**5Ad**

(^{1}H, 500 MHz, DMSO-d_6)

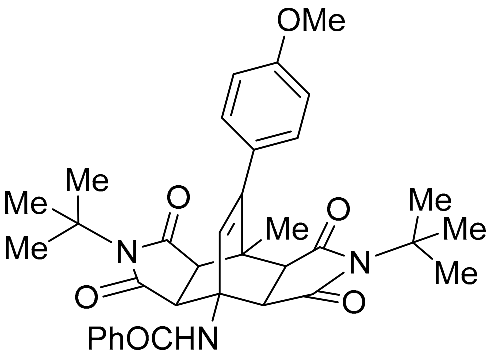

exo,exo-**5Ad**

(^{13}C, 126 MHz, DMSO-d_6)

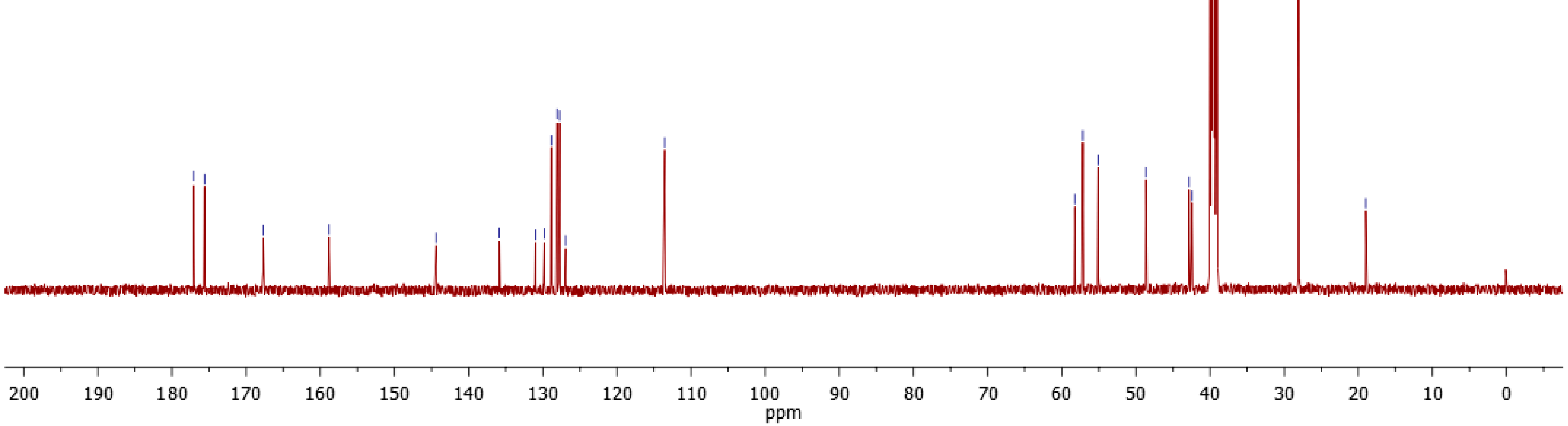

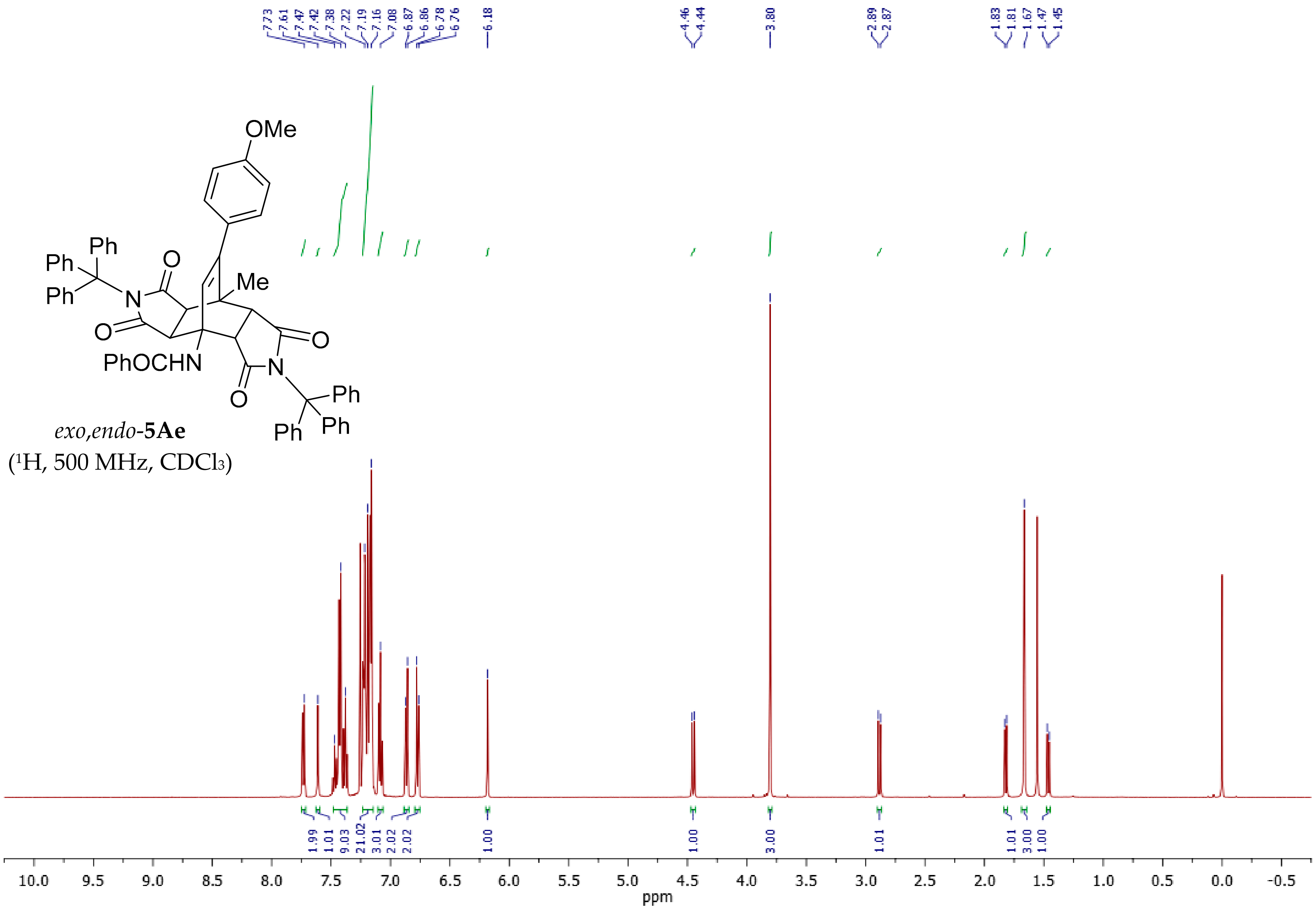

exo,endo-**5Ae**
(^{1}H, 500 MHz, $CDCl_3$)

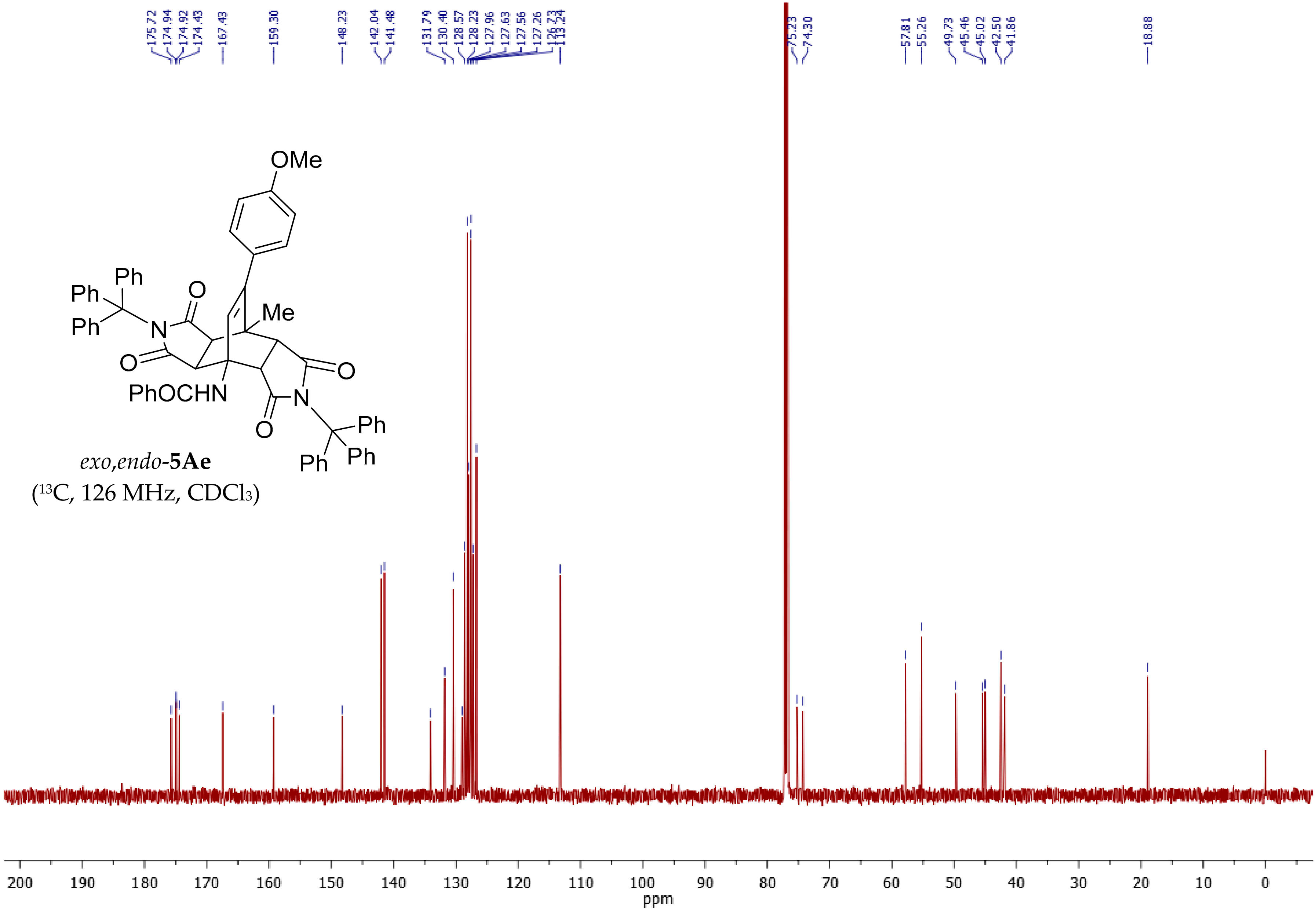

exo,endo-**5Ae**
(^{13}C, 126 MHz, $CDCl_3$)

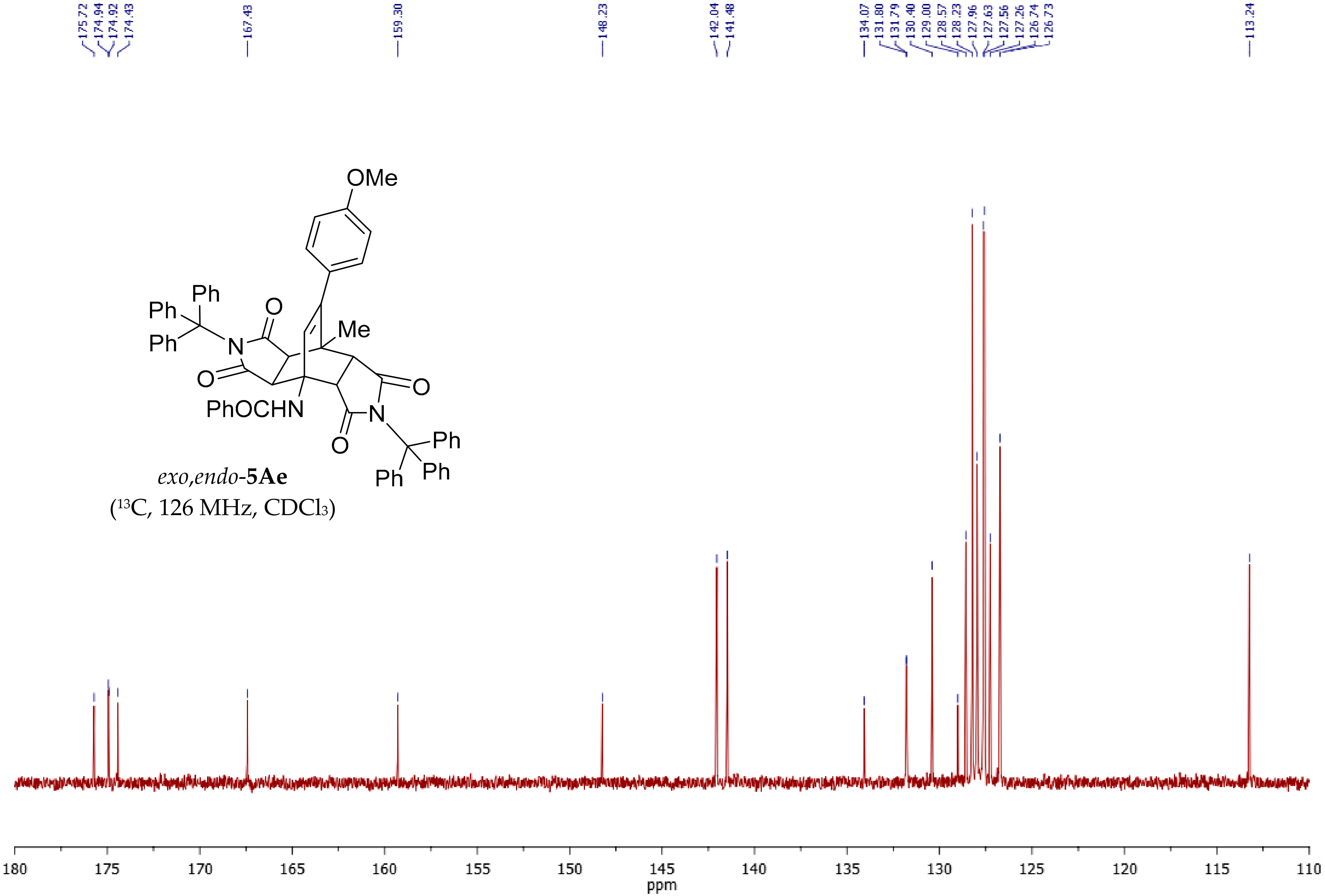

exo,endo-**5Ae**

(^{13}C, 126 MHz, $CDCl_3$)

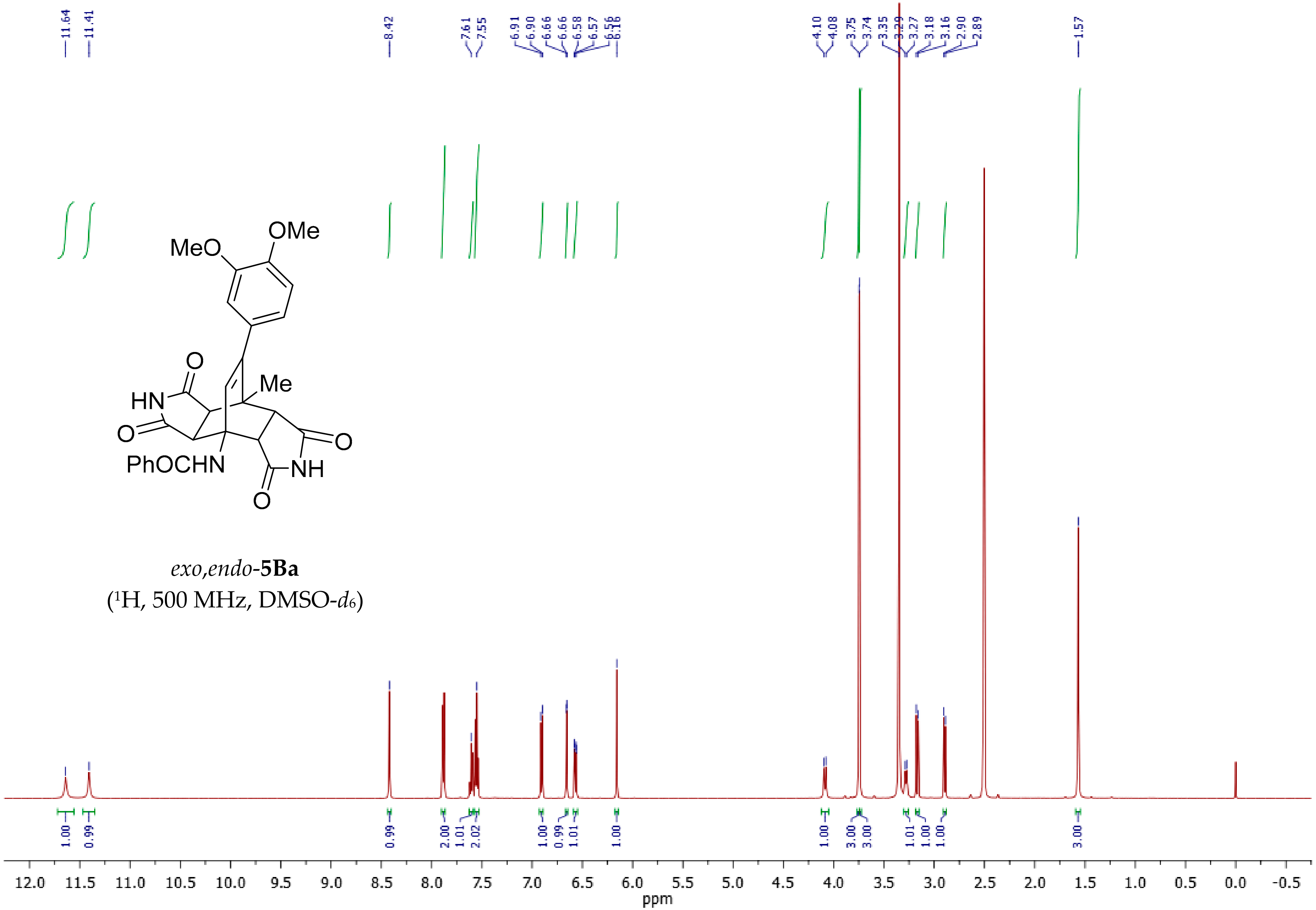

exo,endo-**5Ba**

(^{1}H, 500 MHz, DMSO-d_6)

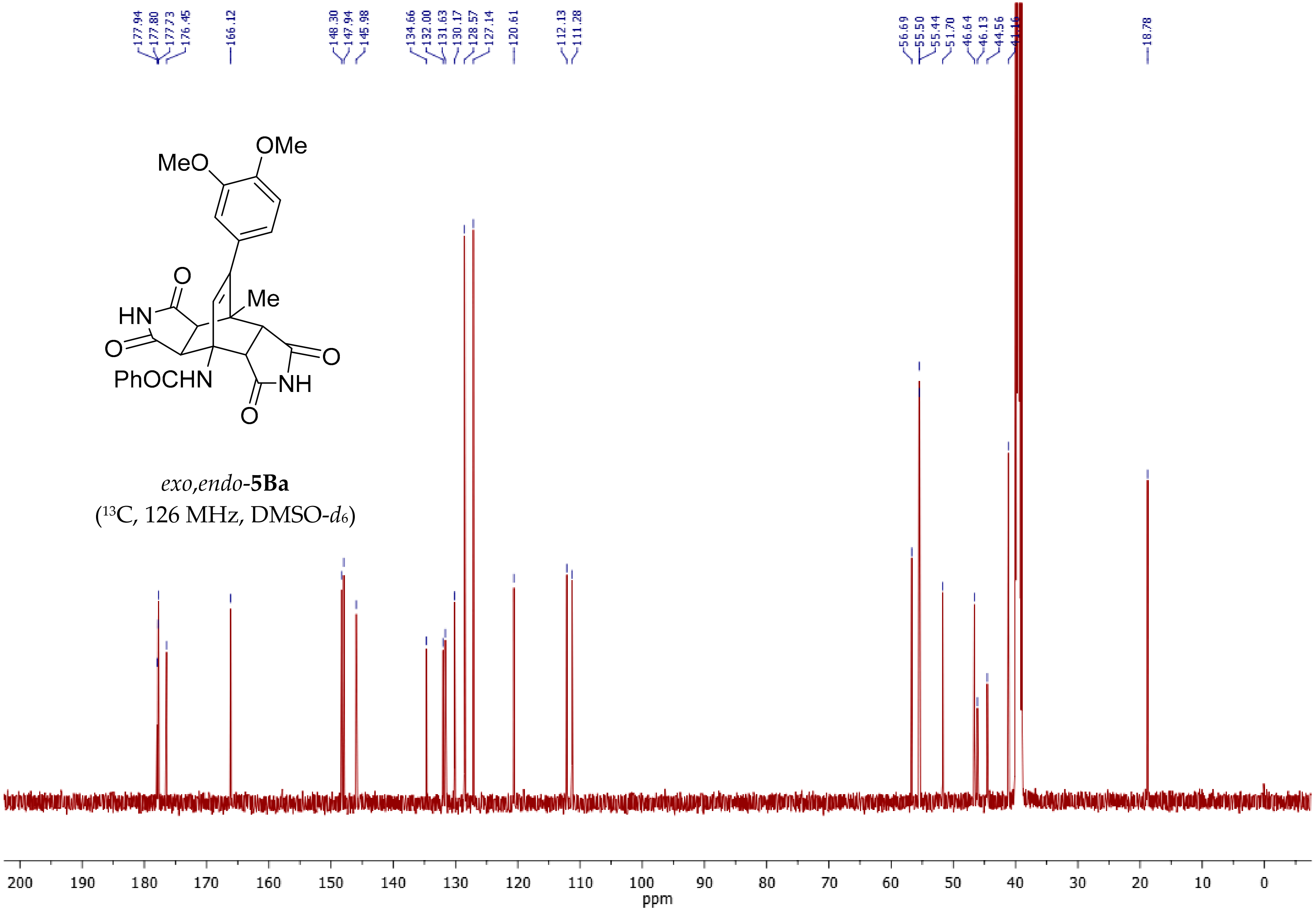

exo,endo-**5Ba**
(^{13}C, 126 MHz, DMSO-d_6)

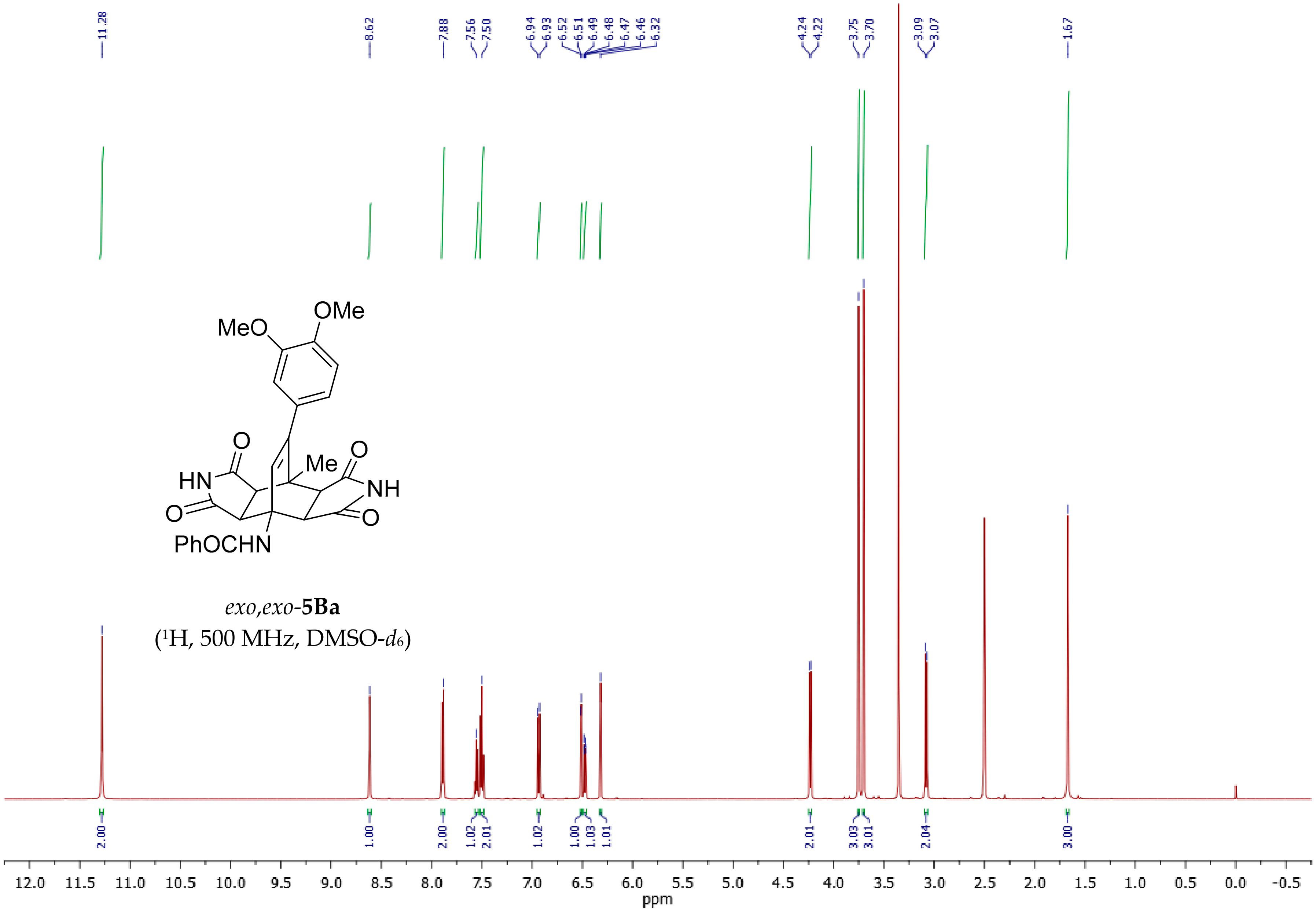

11.28
8.62
7.88
7.56
7.50
6.94
6.93
6.52
6.51
6.49
6.48
6.47
6.46
6.32
4.24
4.22
3.75
3.70
3.09
3.07
1.67
OMe
MeO
O
Me
O
HN
NH
O
O
PhOCHN
exo,*exo*-**5Ba**
(^{1}H, 500 MHz, DMSO-d_6)
2.00
1.00
2.00
1.02
2.01
1.02
1.00
1.03
1.01
2.01
3.03
3.01
2.04
3.00
12.0
11.5
11.0
10.5
10.0
9.5
9.0
8.5
8.0
7.5
7.0
6.5
6.0
5.5
5.0
4.5
4.0
3.5
3.0
2.5
2.0
1.5
1.0
0.5
0.0
-0.5
ppm

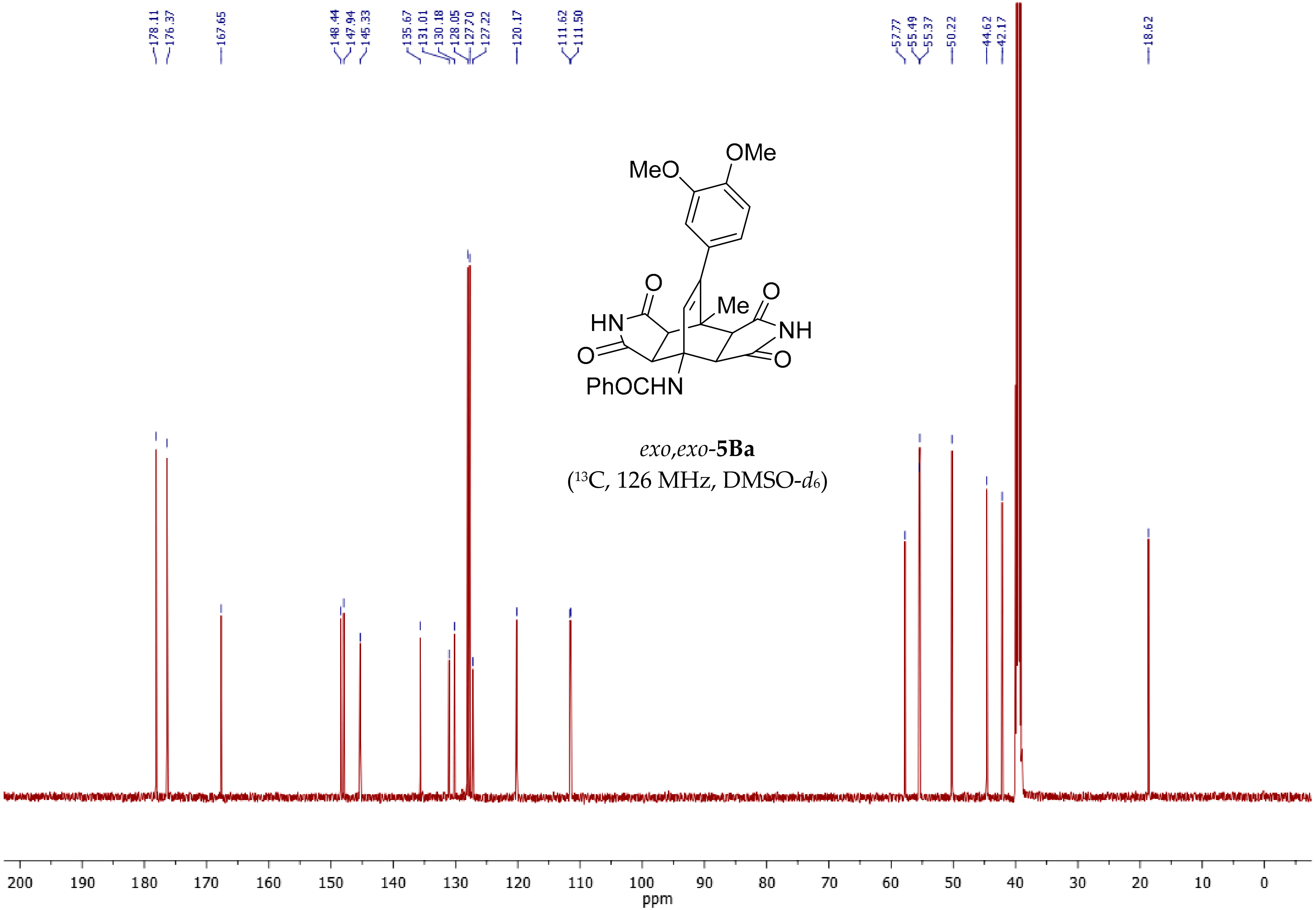

exo,exo-**5Ba**
(^{13}C, 126 MHz, DMSO-d_6)

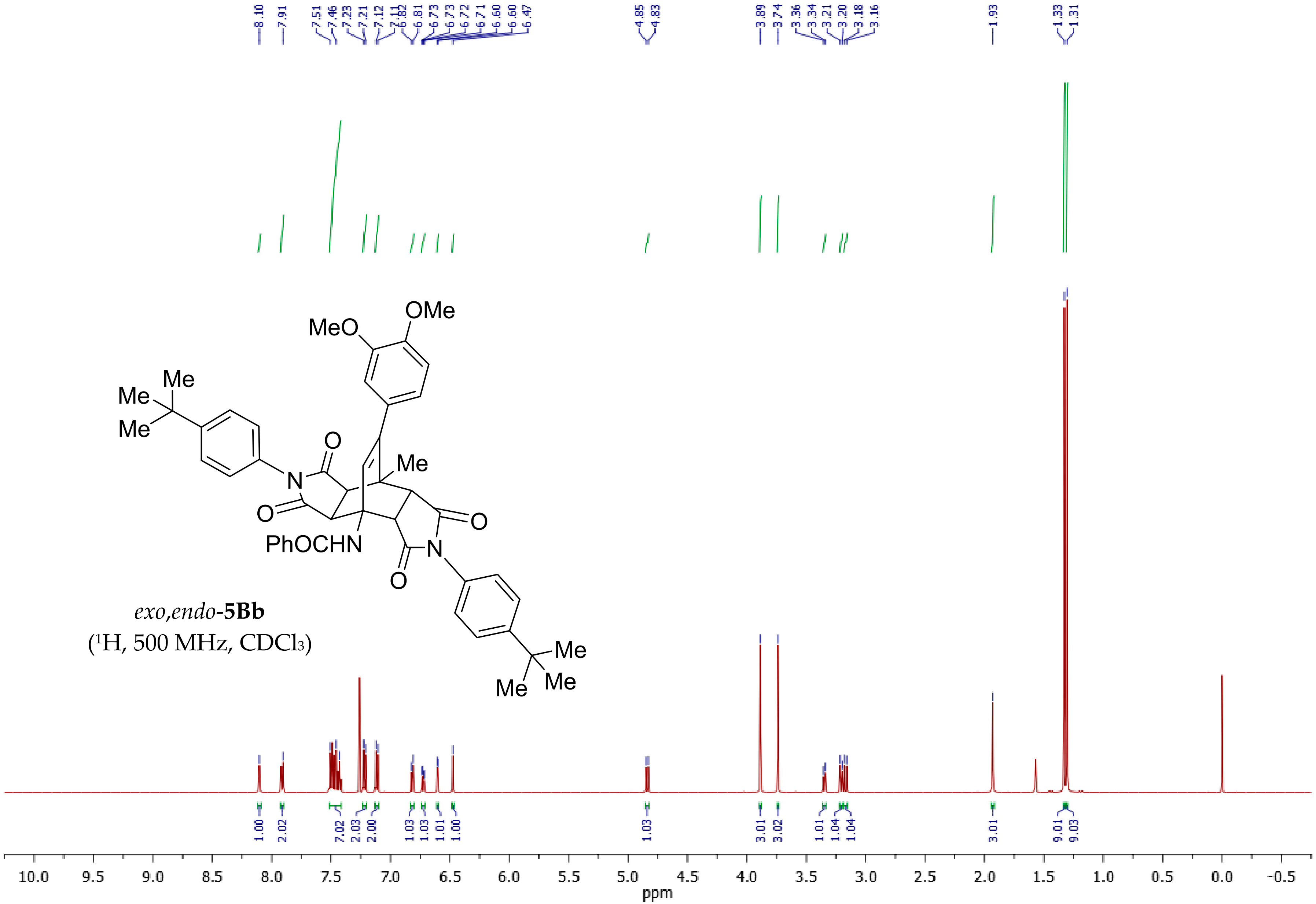

exo,endo-**5Bb**
(^{1}H, 500 MHz, $CDCl_3$)

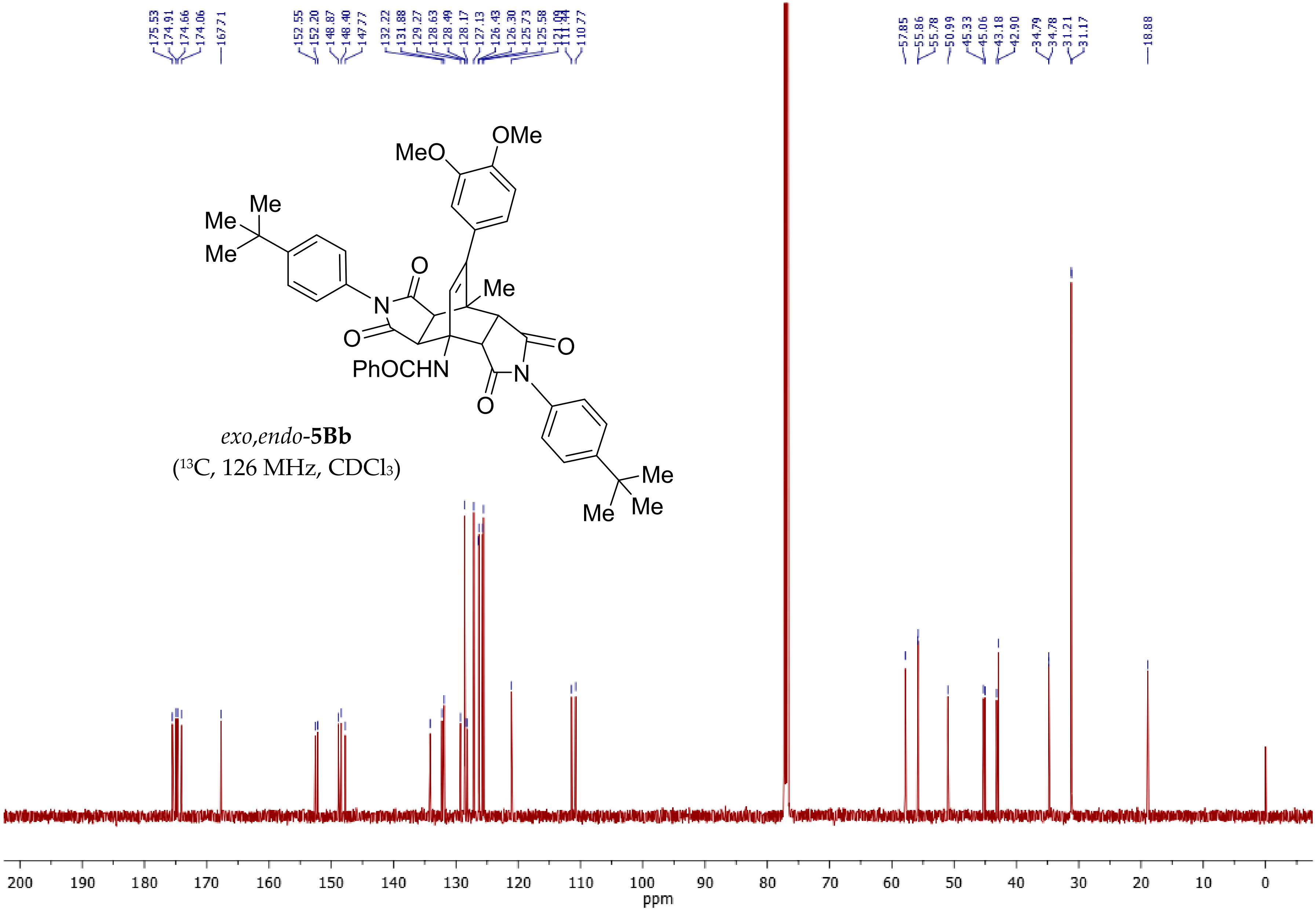

exo,endo-**5Bb**
(^{13}C, 126 MHz, $CDCl_3$)

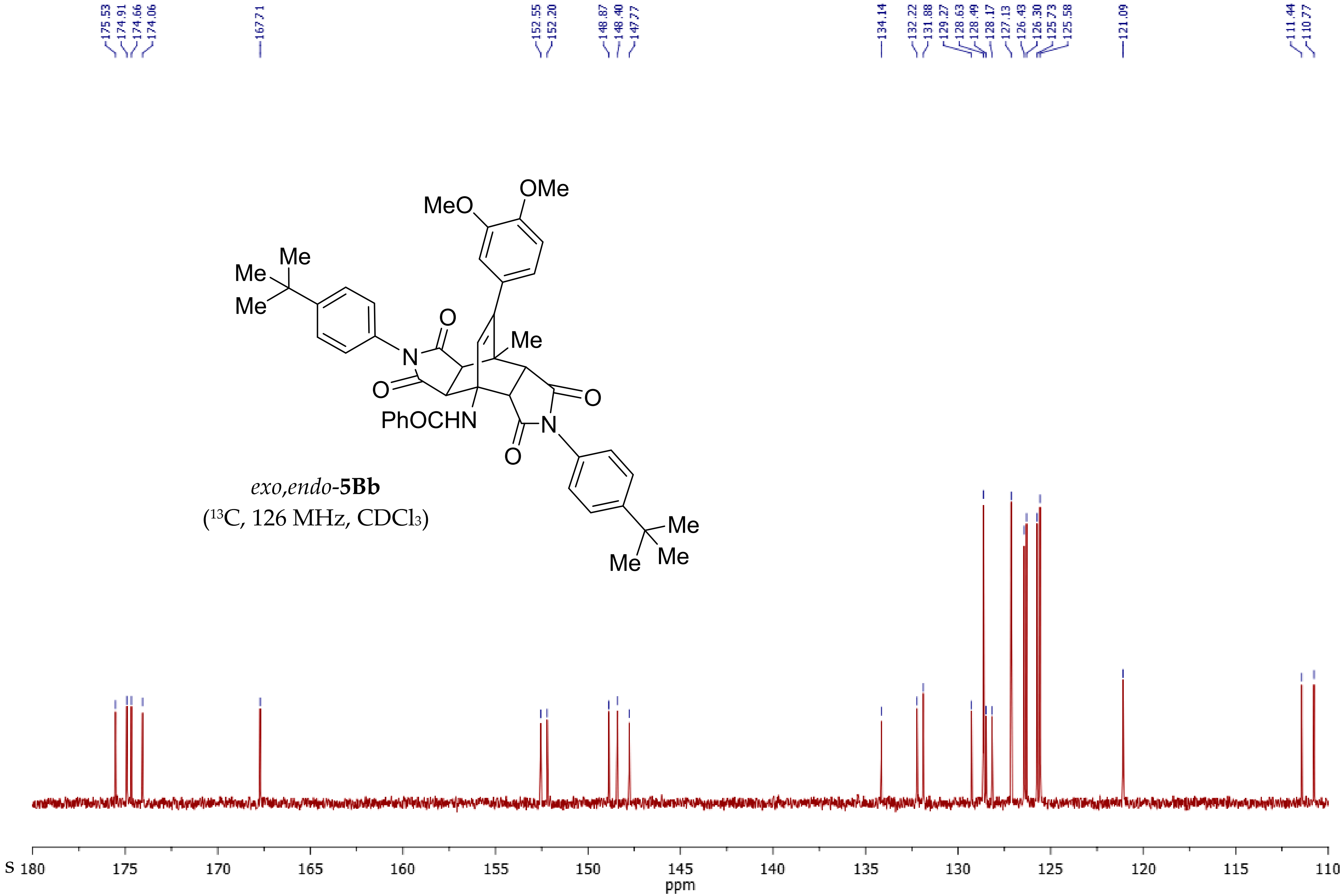

exo,endo-**5Bb**
(^{13}C, 126 MHz, $CDCl_3$)

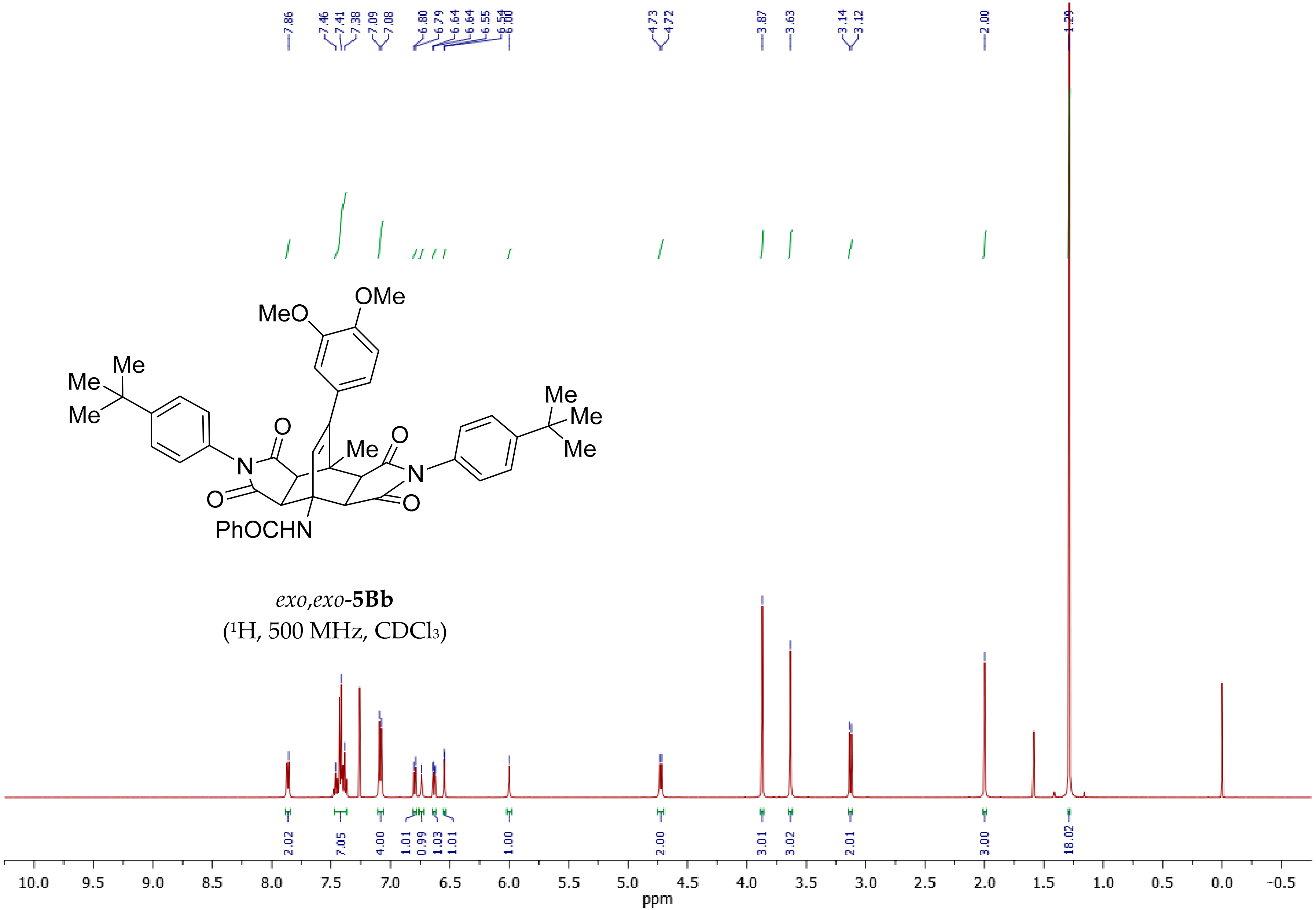
MeO
OMe
Me
Me
Me
Me
Me
Me
O
O
Me
N
N
O
O
PhOCHN
exo,exo-5Bb
(1H, 500 MHz, CDCl3)
ppm

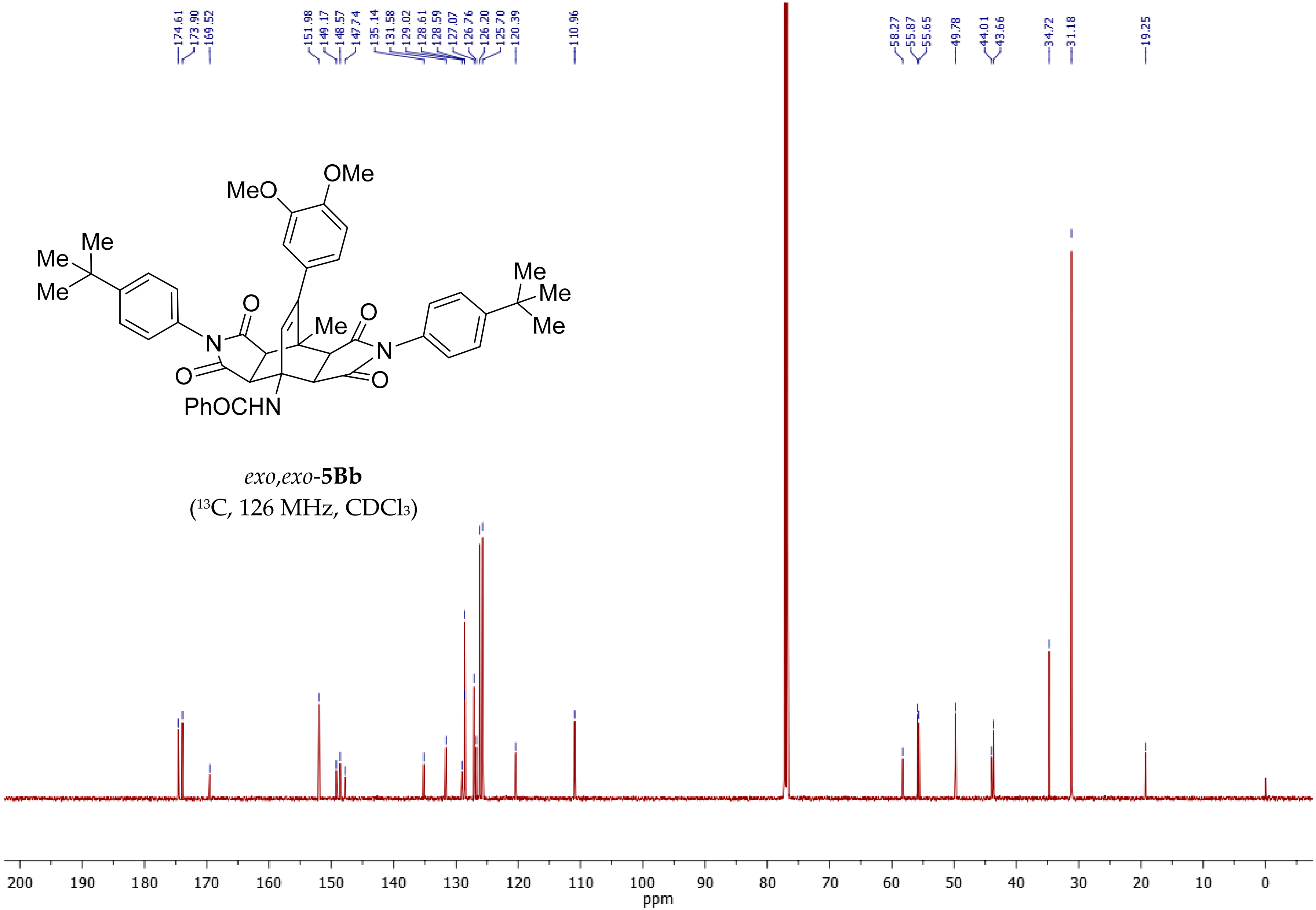

exo,exo-**5Bb**
(^{13}C, 126 MHz, $CDCl_3$)

exo,endo-**5Bc**
(^{1}H, 500 MHz, $CDCl_3$)

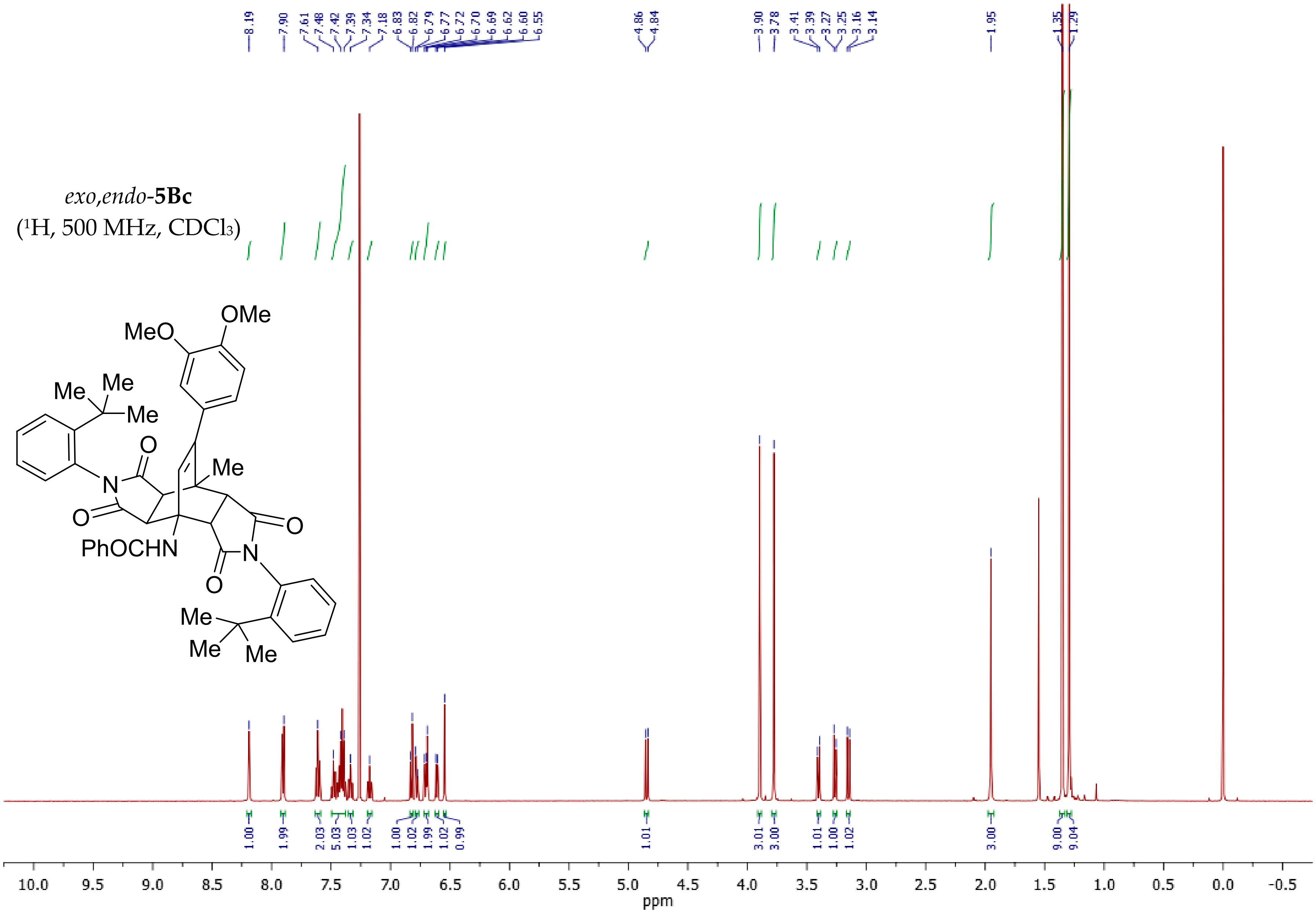

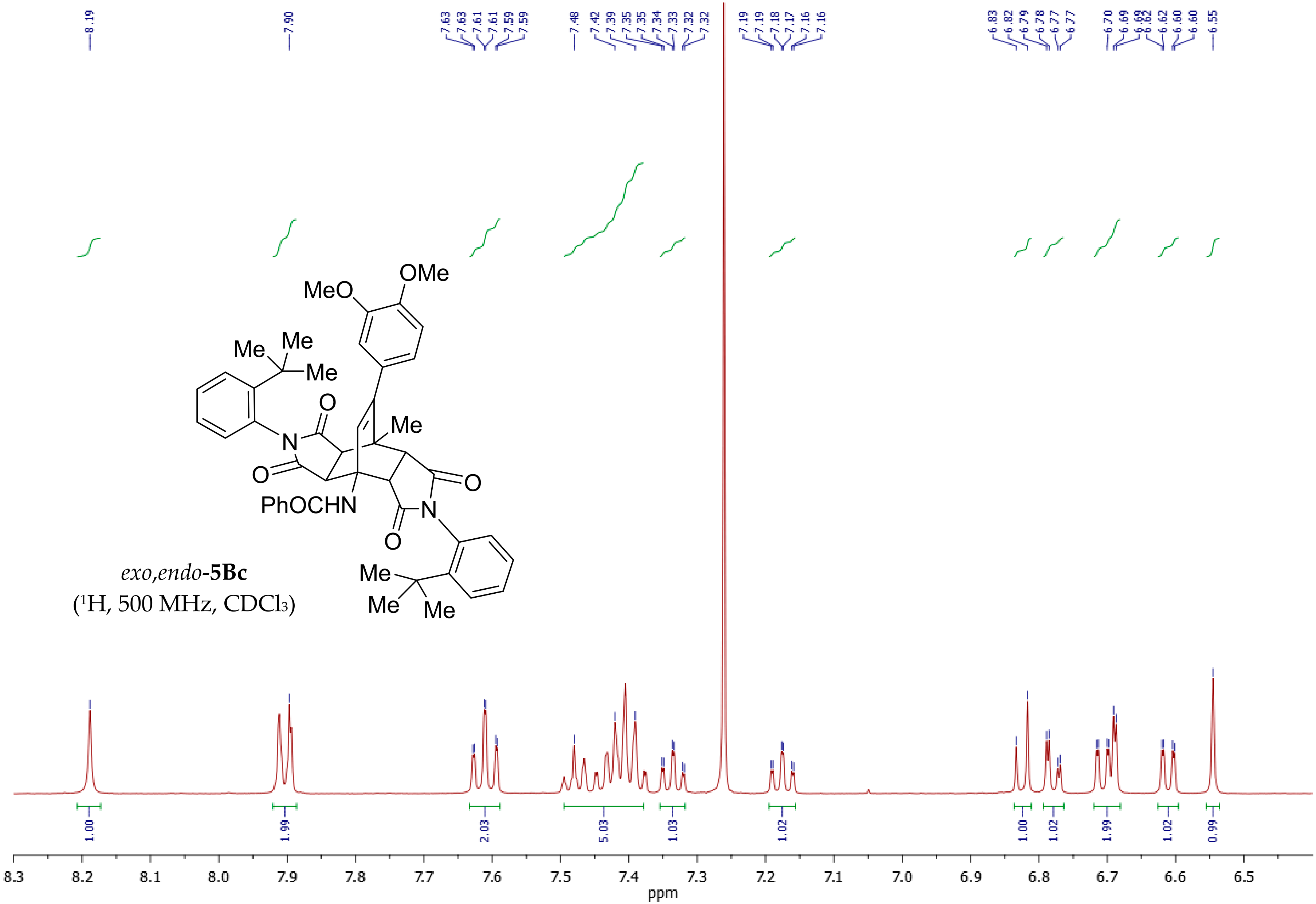

exo,endo-**5Bc**

(^{1}H, 500 MHz, $CDCl_3$)

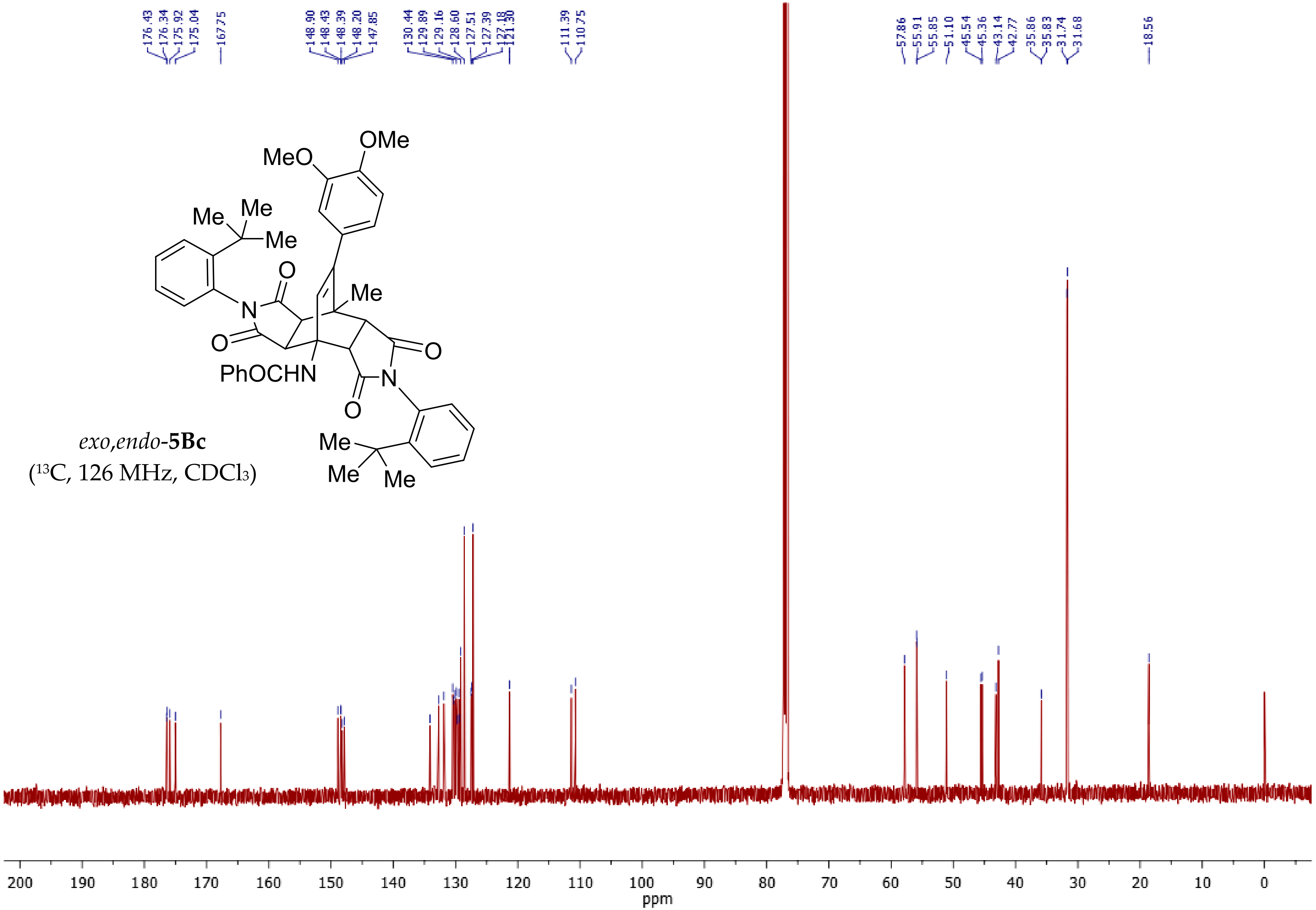
176.43
176.34
175.92
175.04
167.75
148.90
148.43
148.39
148.20
147.85
130.44
129.89
129.16
128.60
127.51
127.39
127.18
121.30
111.39
110.75
57.86
55.91
55.85
51.10
45.54
45.36
43.14
42.77
35.86
35.83
31.74
31.68
18.56
MeO
OMe
Me
Me
Me
O
N
O
Me
O
PhOCHN
N
O
Me
Me
Me
exo,endo-**5Bc**
(^{13}C, 126 MHz, CDCl$_3$)
200
190
180
170
160
150
140
130
120
110
100
90
80
70
60
50
40
30
20
10
0
ppm

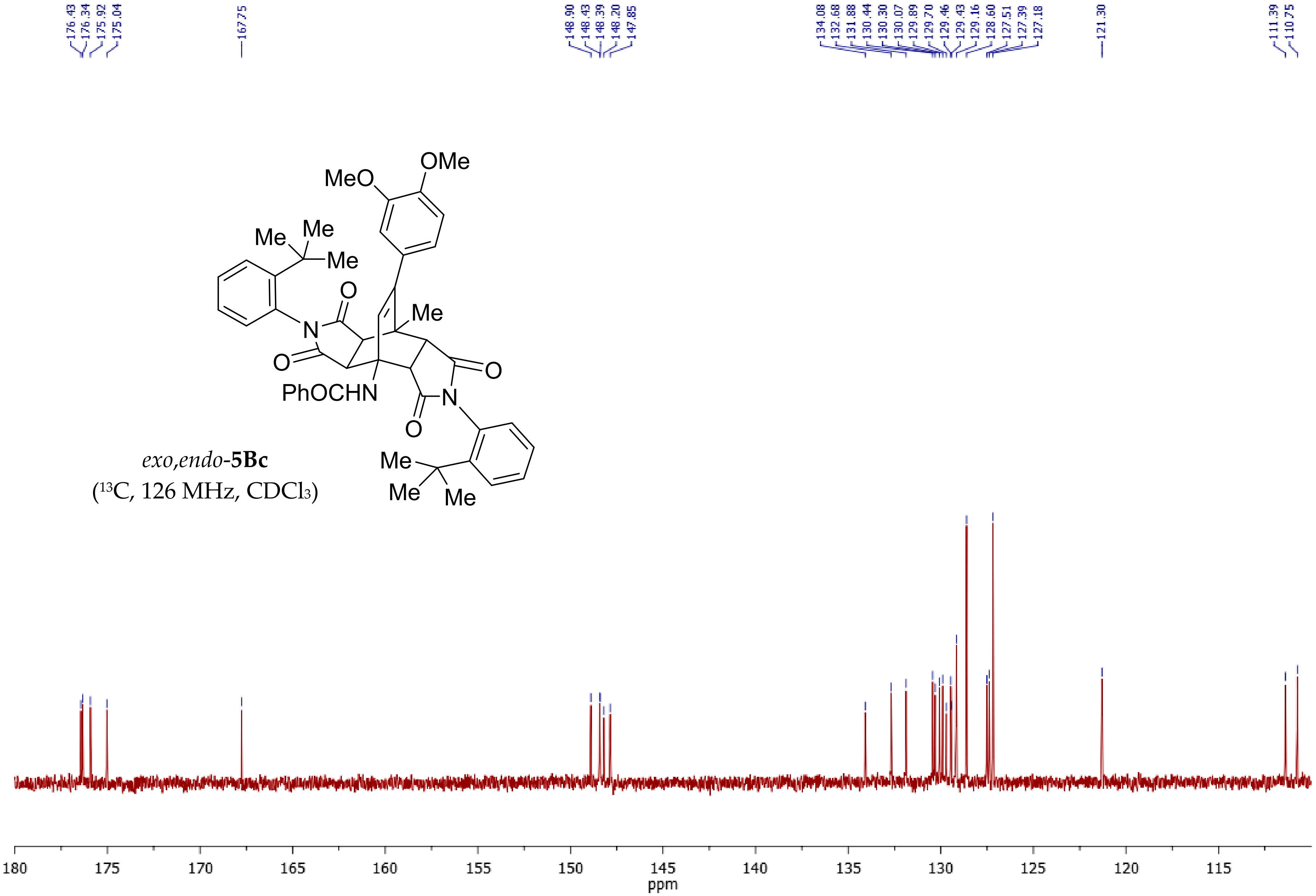

exo,endo-**5Bc**
(^{13}C, 126 MHz, $CDCl_3$)

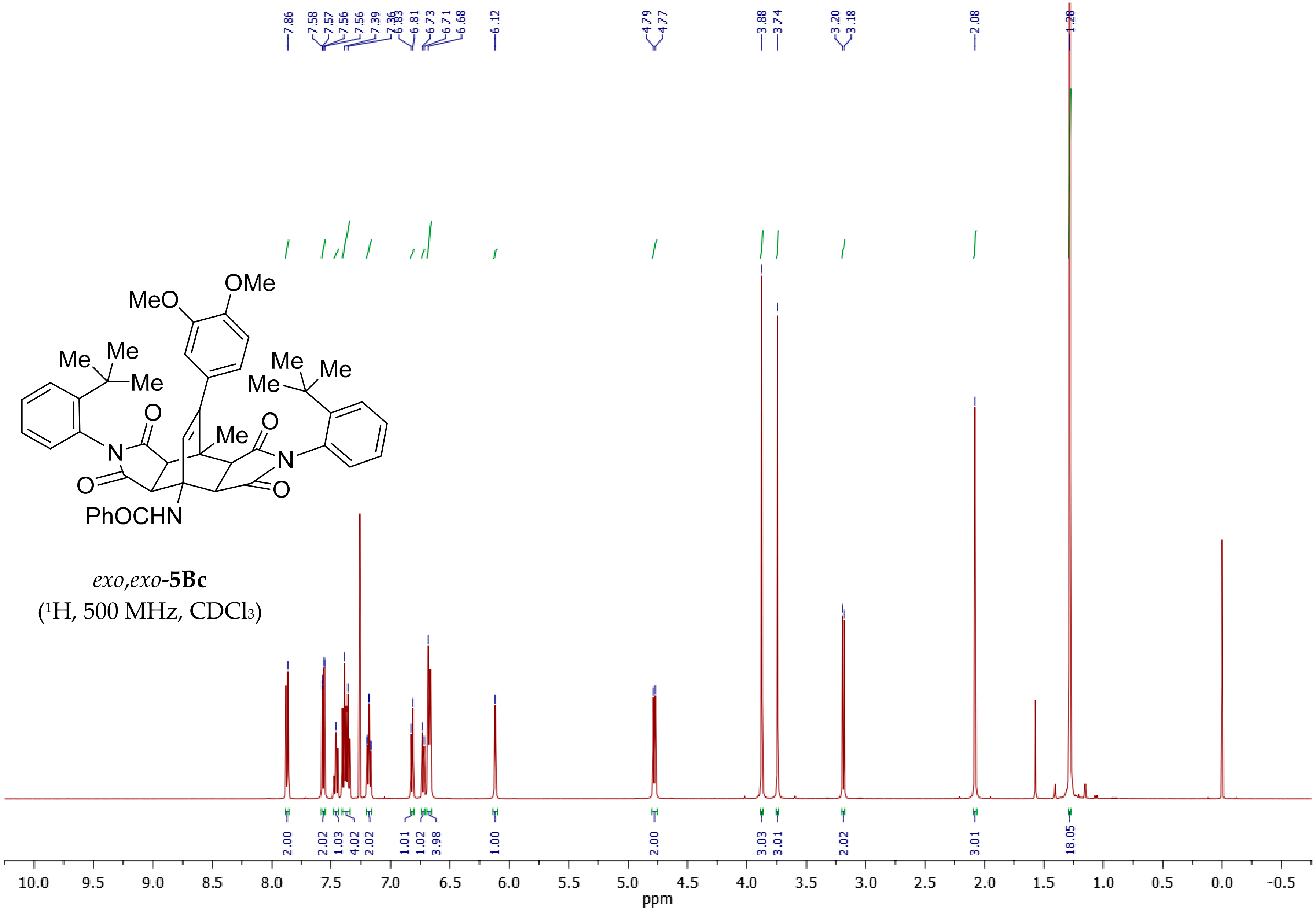

exo,exo-**5Bc**

(^{1}H, 500 MHz, $CDCl_3$)

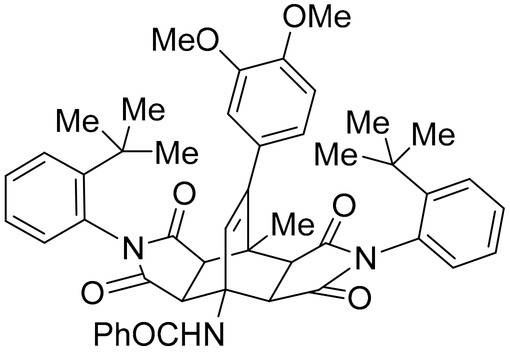

exo,exo-**5Bc**
(^{1}H, 500 MHz, $CDCl_3$)

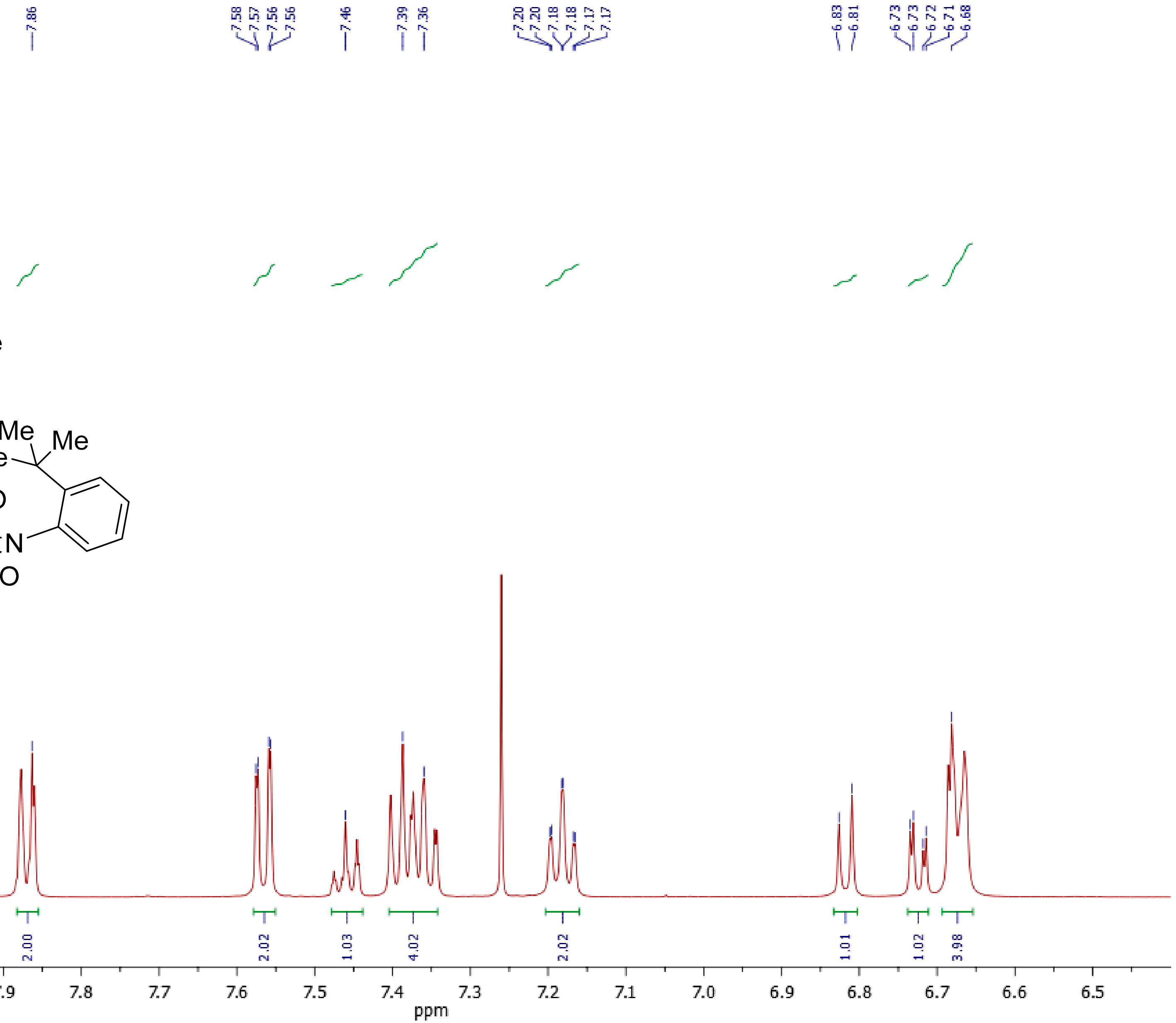

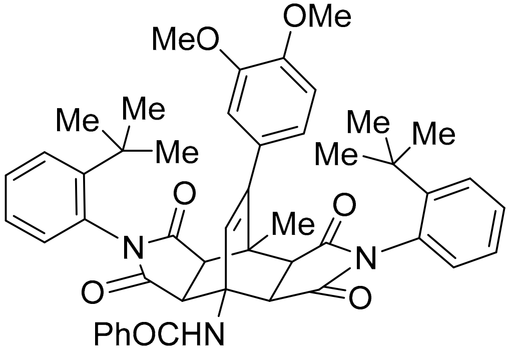

exo,exo-**5Bc**
(^{13}C, 126 MHz, $CDCl_3$)

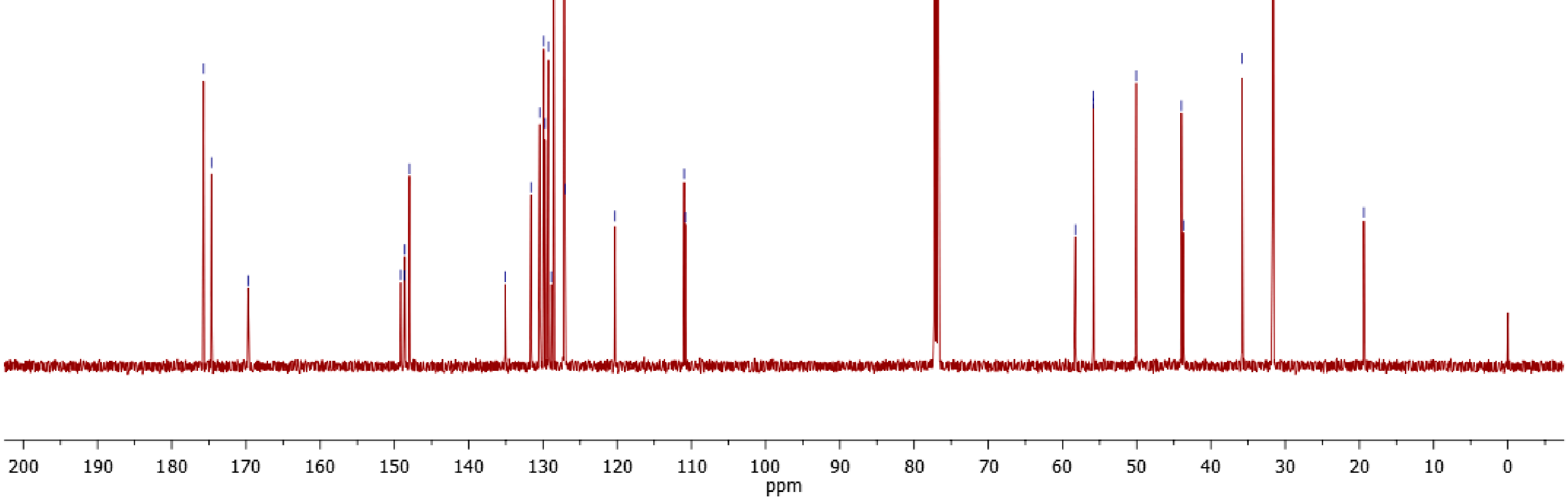

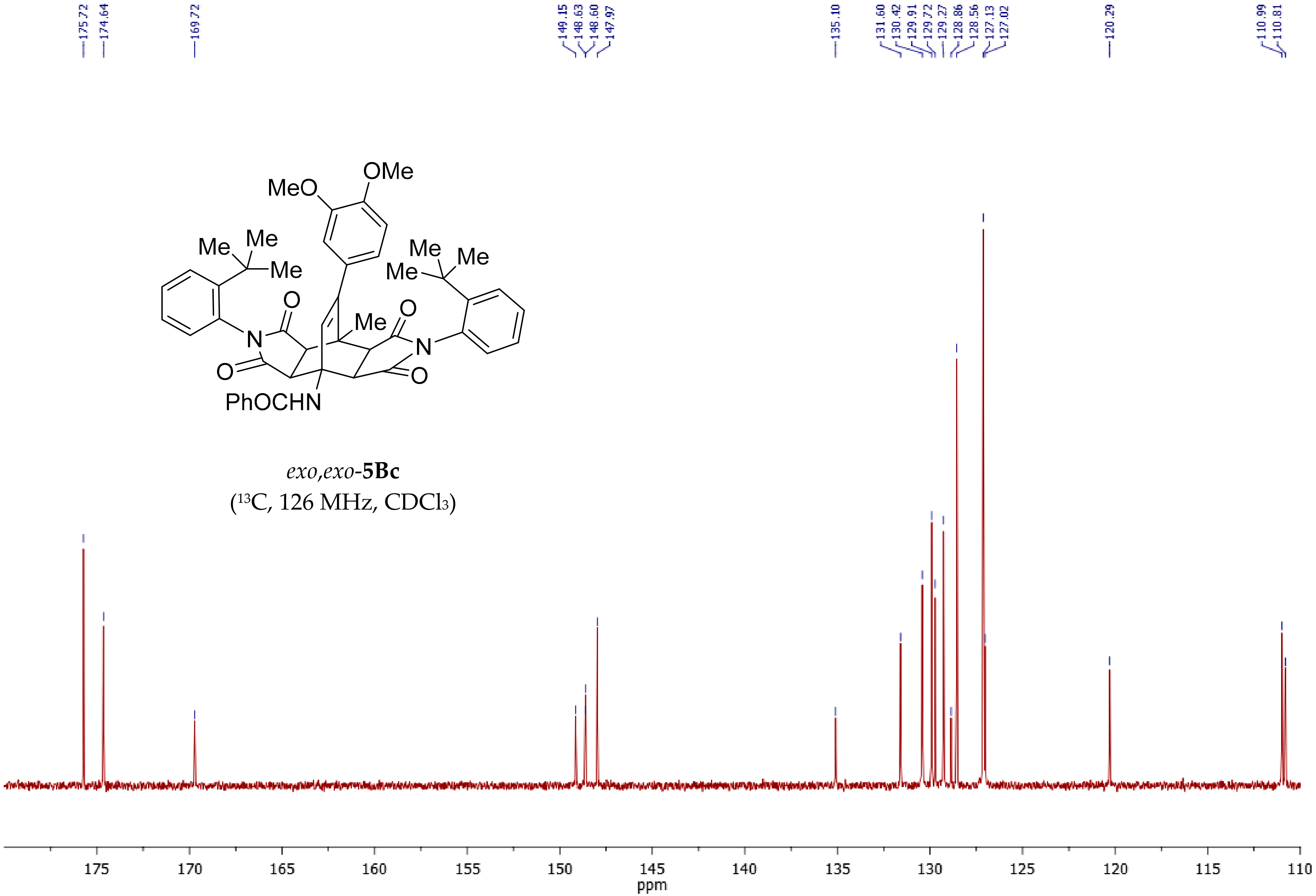

exo,exo-**5Bc**

(^{13}C, 126 MHz, $CDCl_3$)

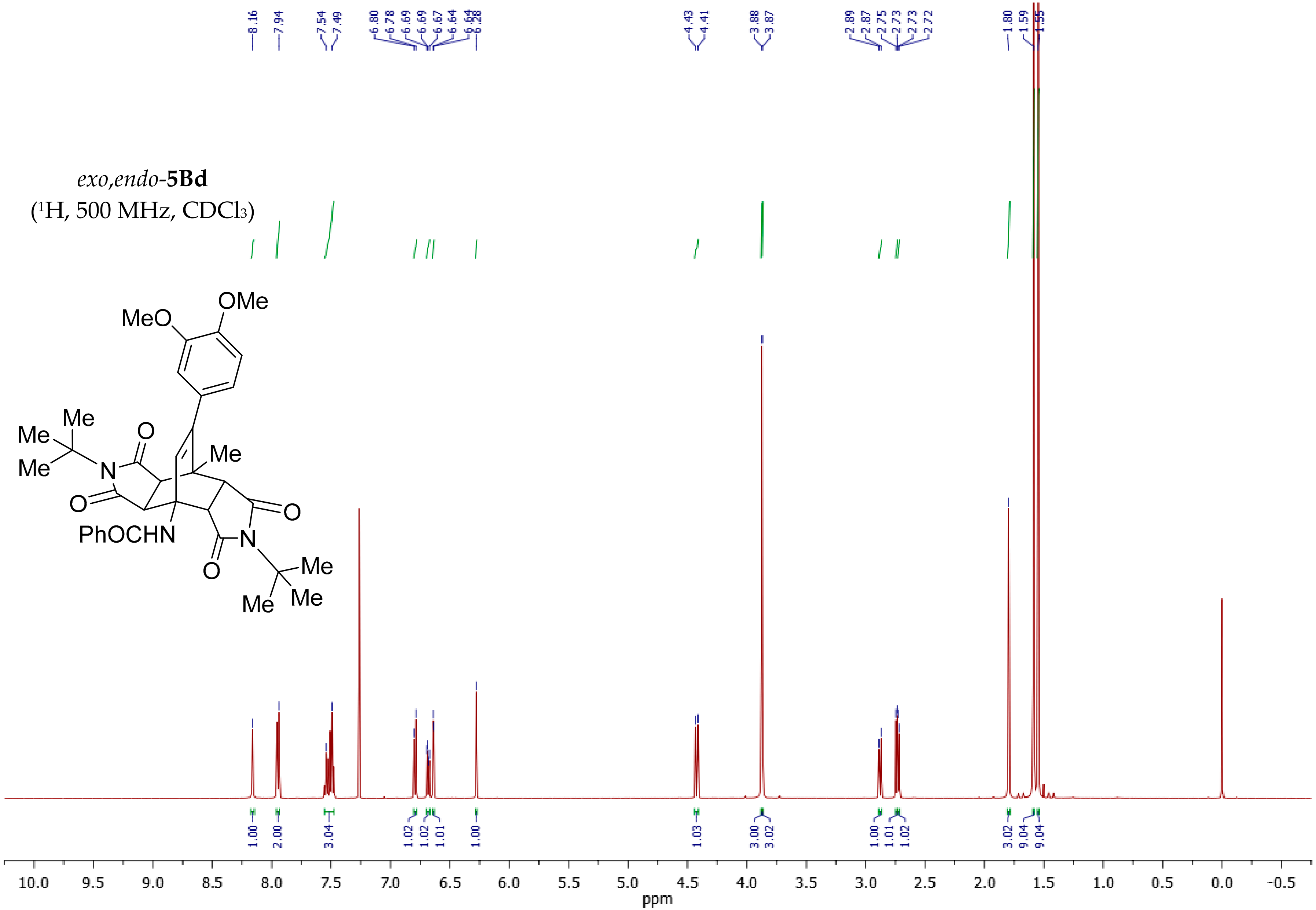

8.16
7.94
7.54
7.49
6.80
6.78
6.69
6.69
6.67
6.64
6.64
6.28
4.43
4.41
3.88
3.87
2.89
2.87
2.75
2.73
2.73
2.72
1.80
1.59
1.55
exo,*endo*-**5Bd**
(¹H, 500 MHz, CDCl₃)
OMe
MeO
Me
Me
Me
O
N
O
Me
O
PhOCHN
O
N
Me
Me
Me
1.00
2.00
3.04
1.02
1.02
1.01
1.00
1.03
3.00
3.02
1.00
1.01
1.02
3.02
9.04
9.04
10.0
9.5
9.0
8.5
8.0
7.5
7.0
6.5
6.0
5.5
5.0
4.5
4.0
3.5
3.0
2.5
2.0
1.5
1.0
0.5
0.0
-0.5
ppm

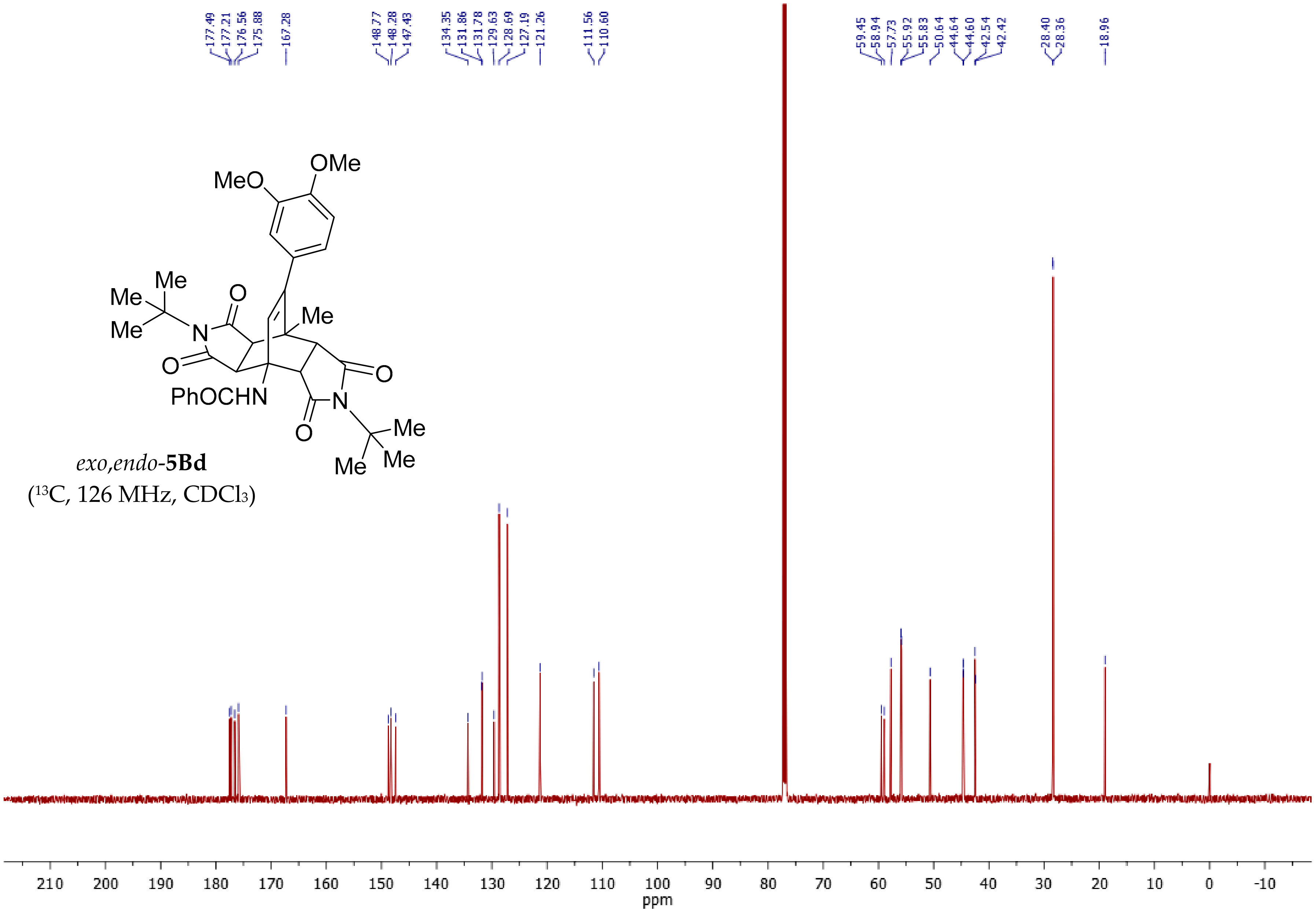

exo,endo-**5Bd**
(^{13}C, 126 MHz, $CDCl_3$)

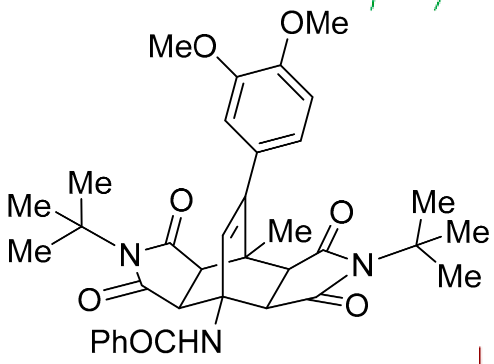

exo,*exo*-**5Bd**
(^{1}H, 500 MHz, $CDCl_3$)

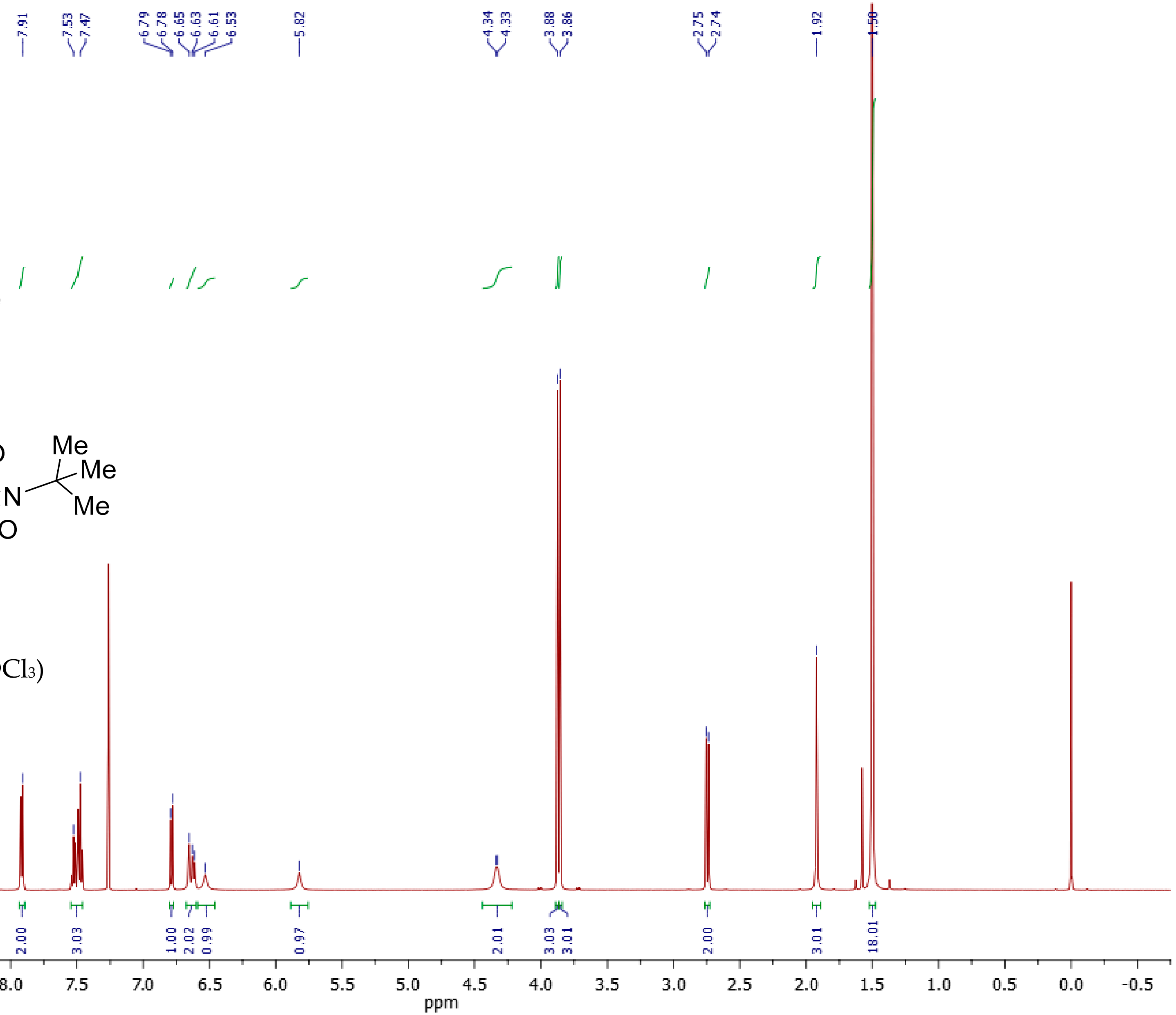

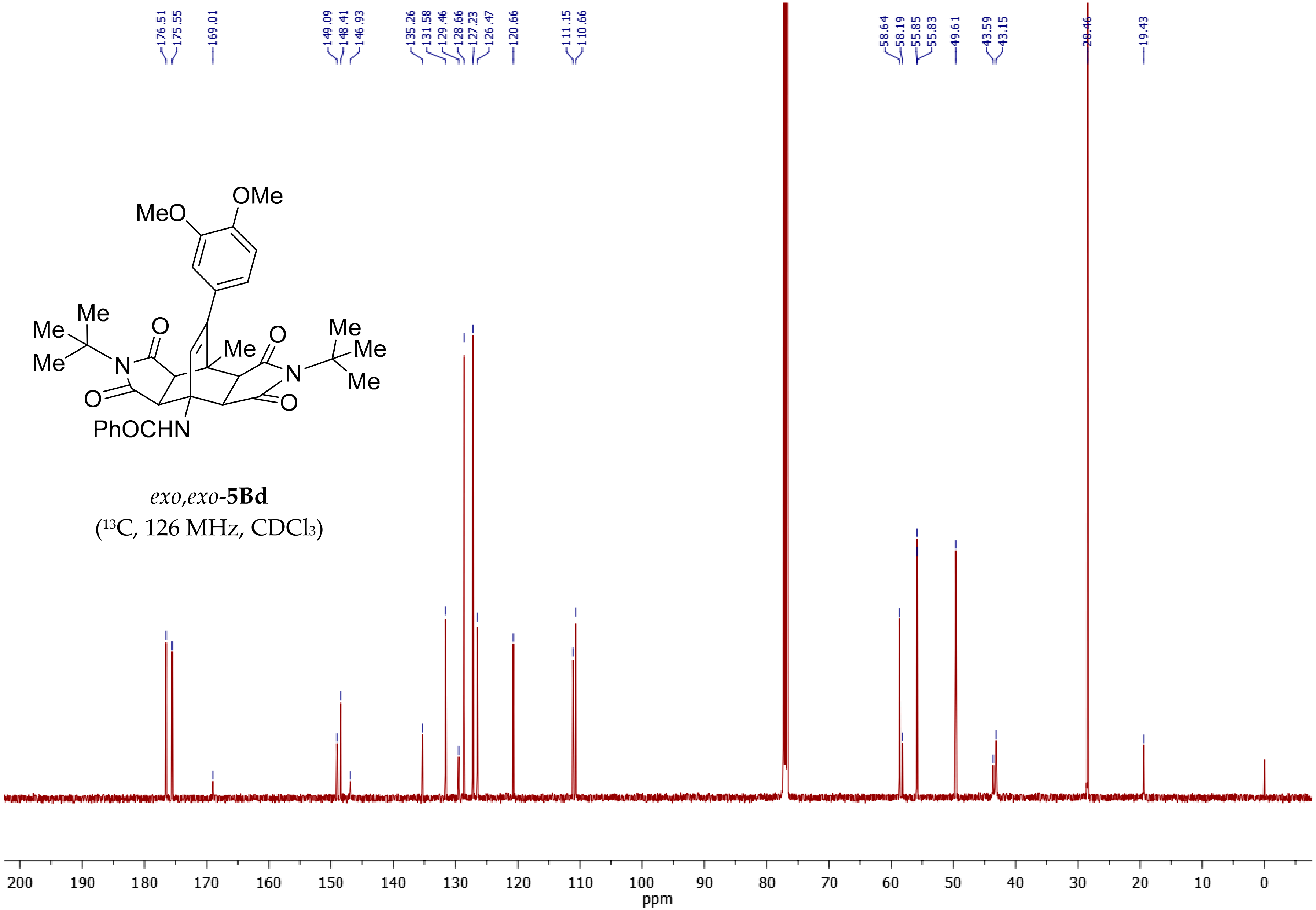

exo,exo-**5Bd**
(^{13}C, 126 MHz, $CDCl_3$)

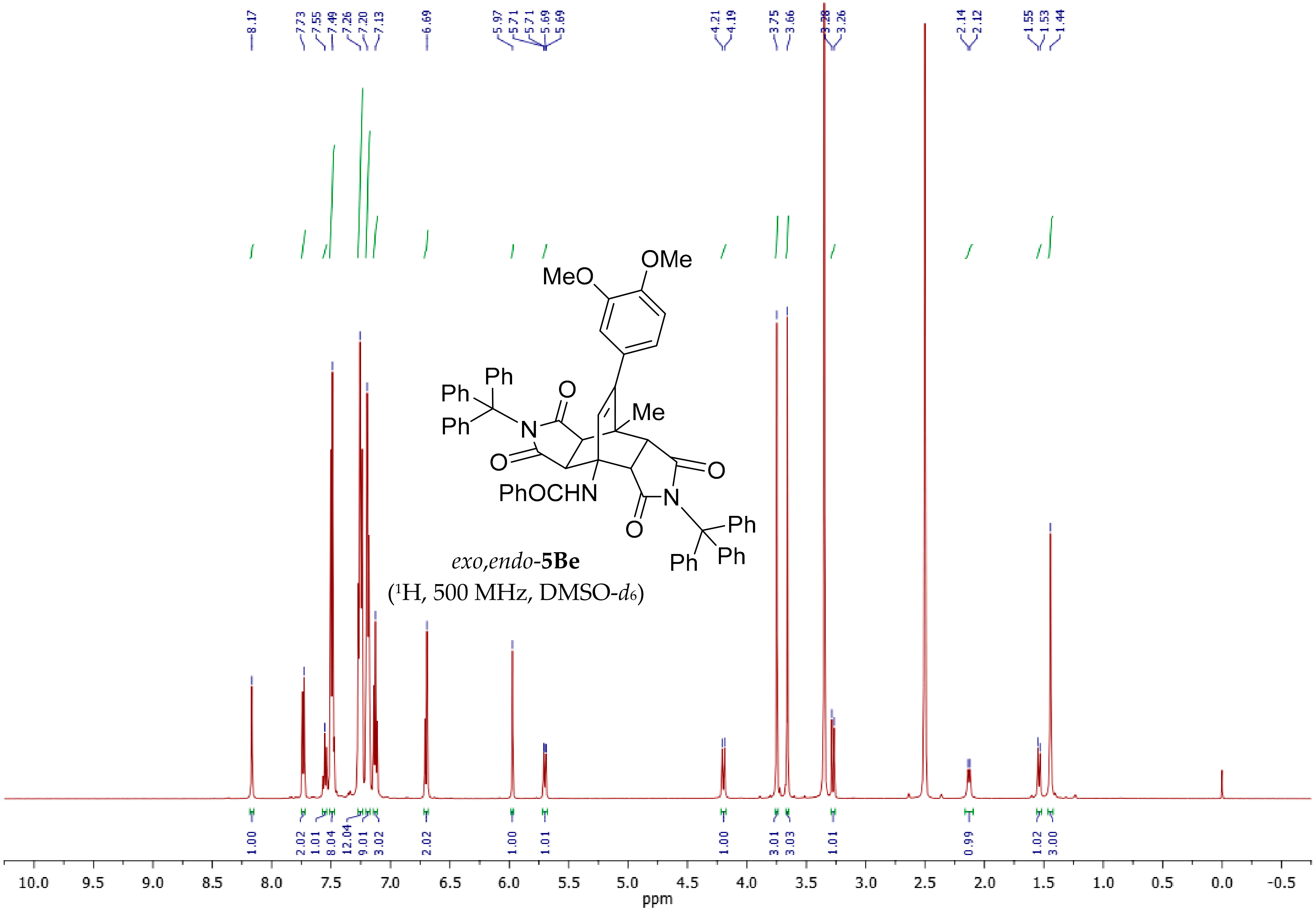

exo,endo-**5Be**

(^{1}H, 500 MHz, DMSO-d_6)

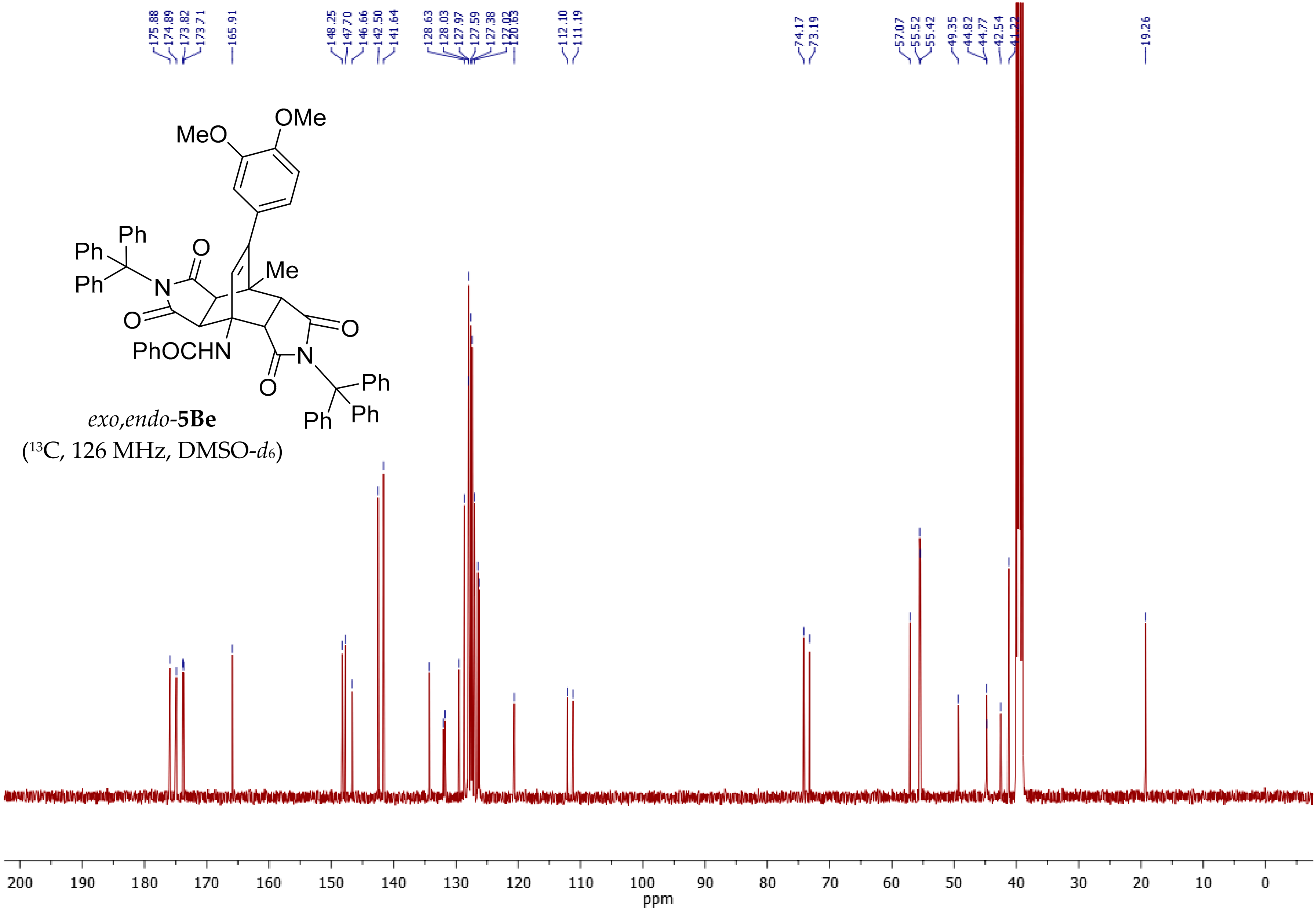

exo,endo-**5Be**

(^{13}C, 126 MHz, DMSO-d_6)

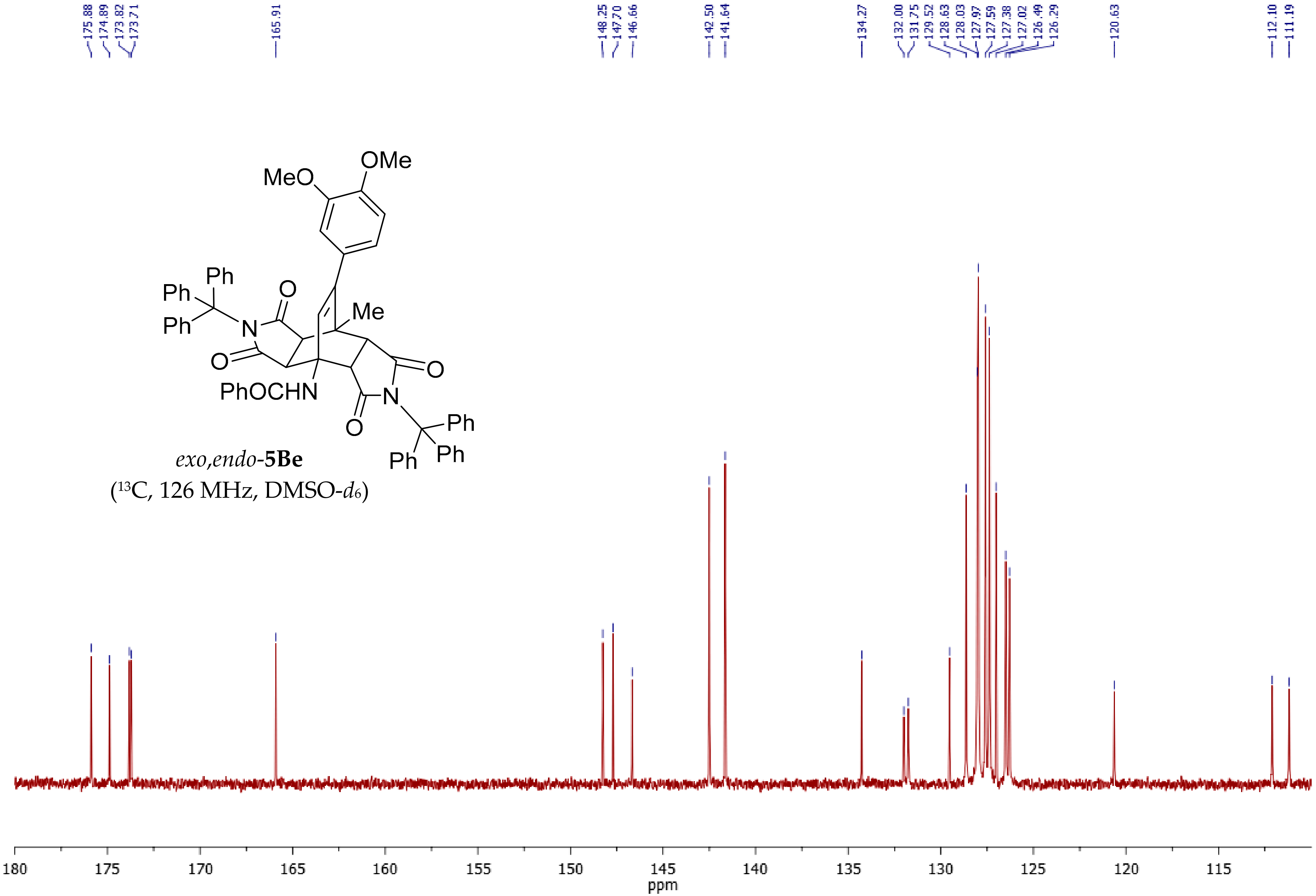

exo,endo-**5Be**
(^{13}C, 126 MHz, DMSO-d_6)

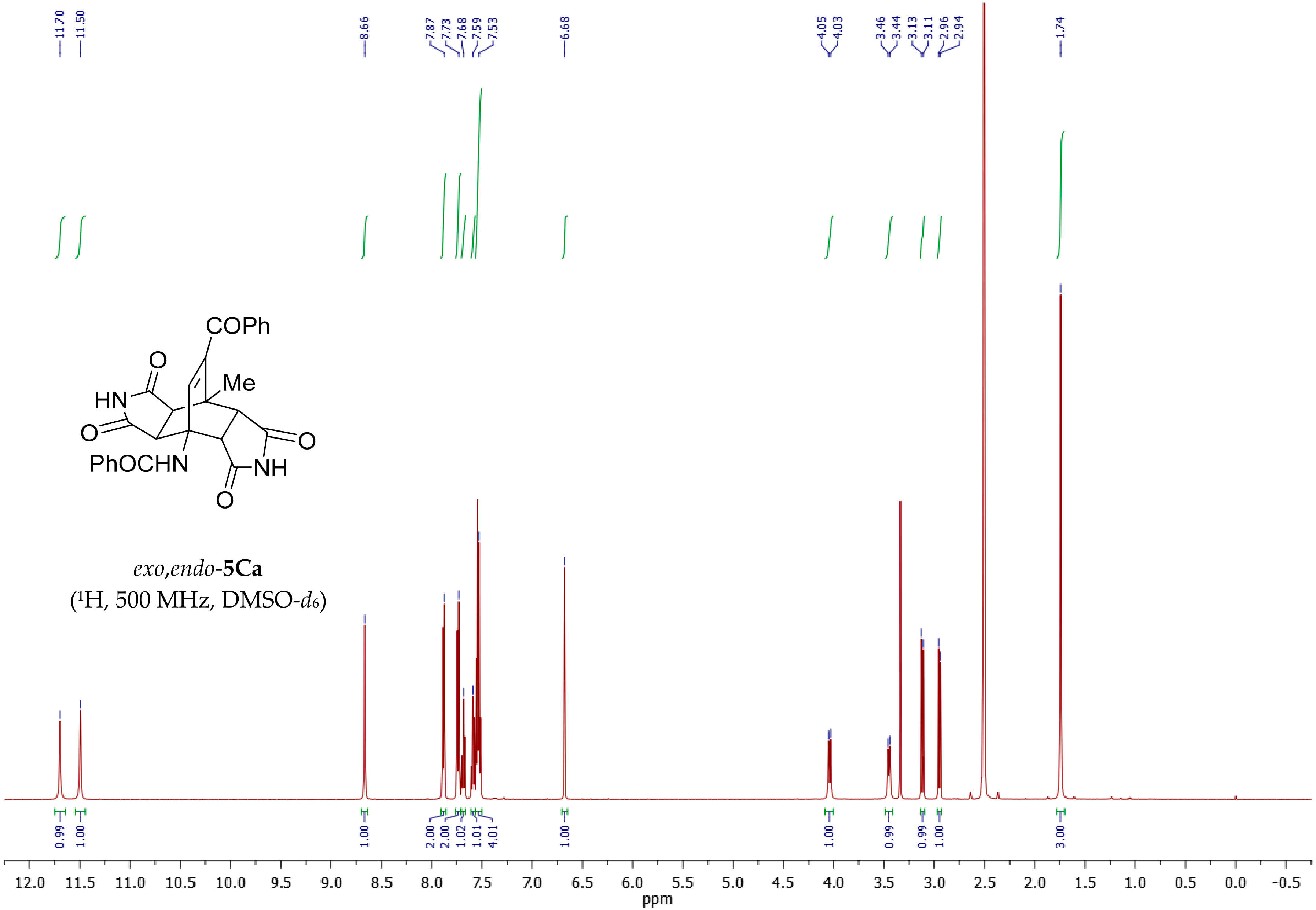

exo,endo-**5Ca**
(^{1}H, 500 MHz, DMSO-d_6)

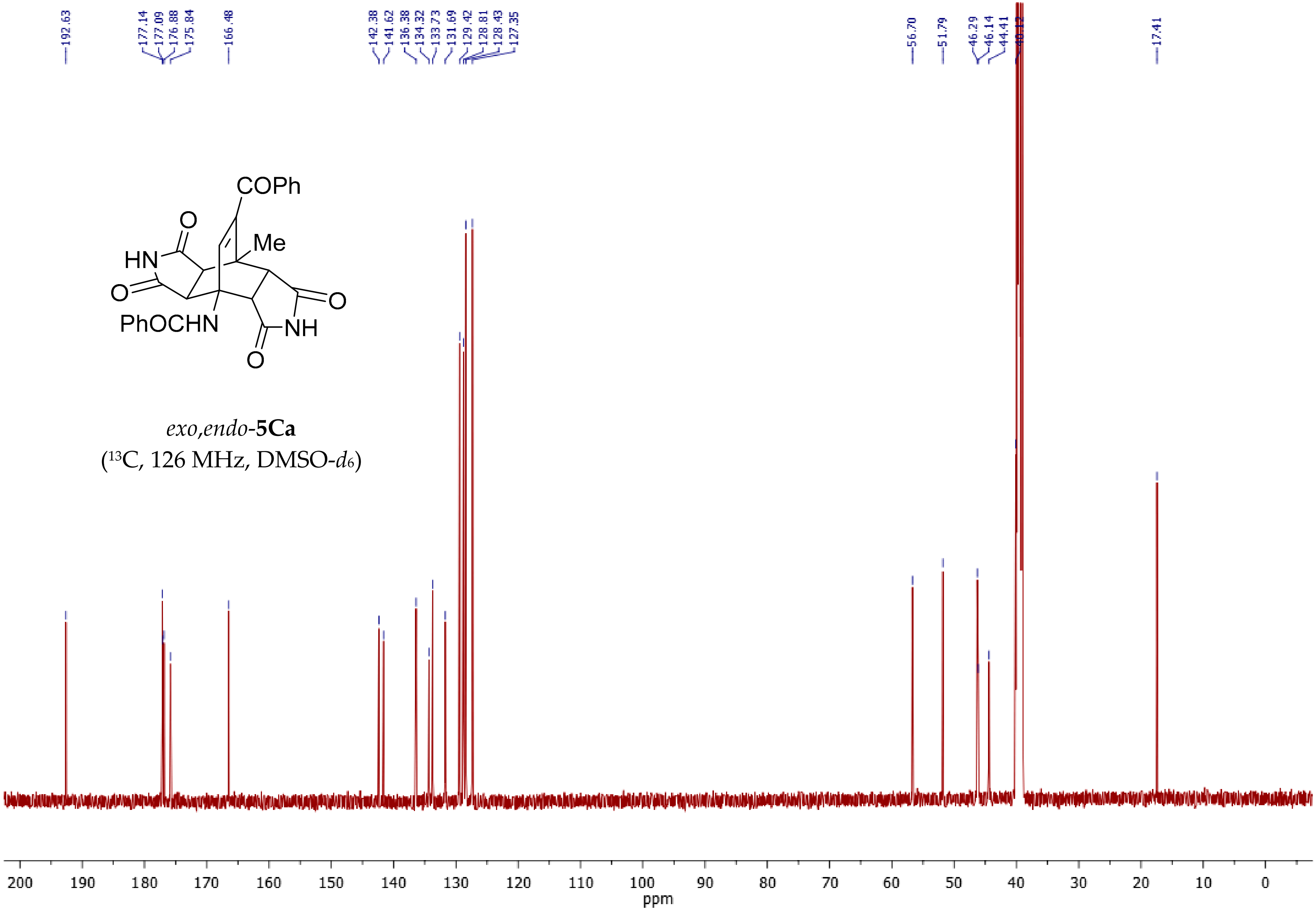

exo,endo-**5Ca**
(^{13}C, 126 MHz, DMSO-d_6)

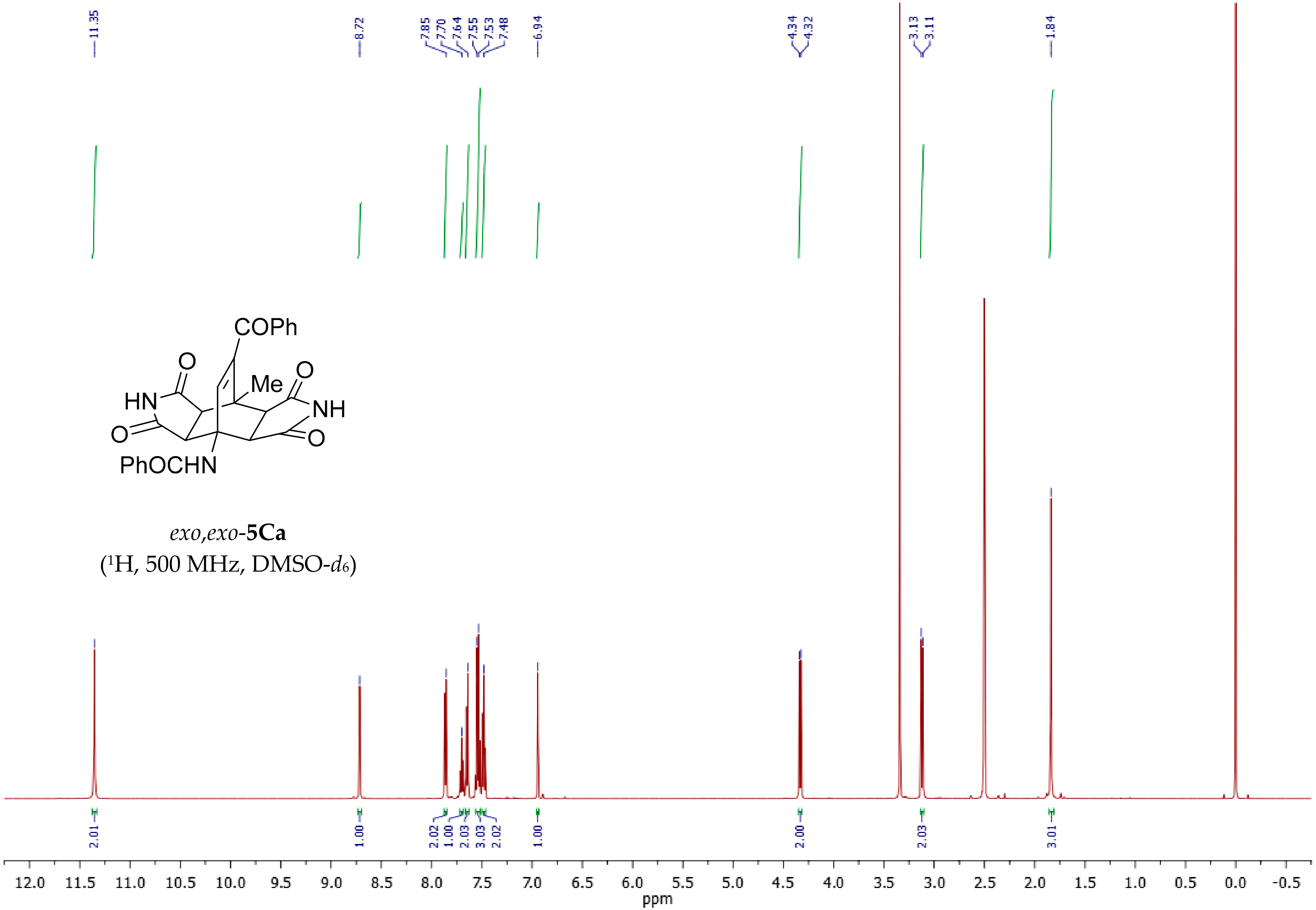

exo,exo-**5Ca**
(^{1}H, 500 MHz, DMSO-*d*$_6$)

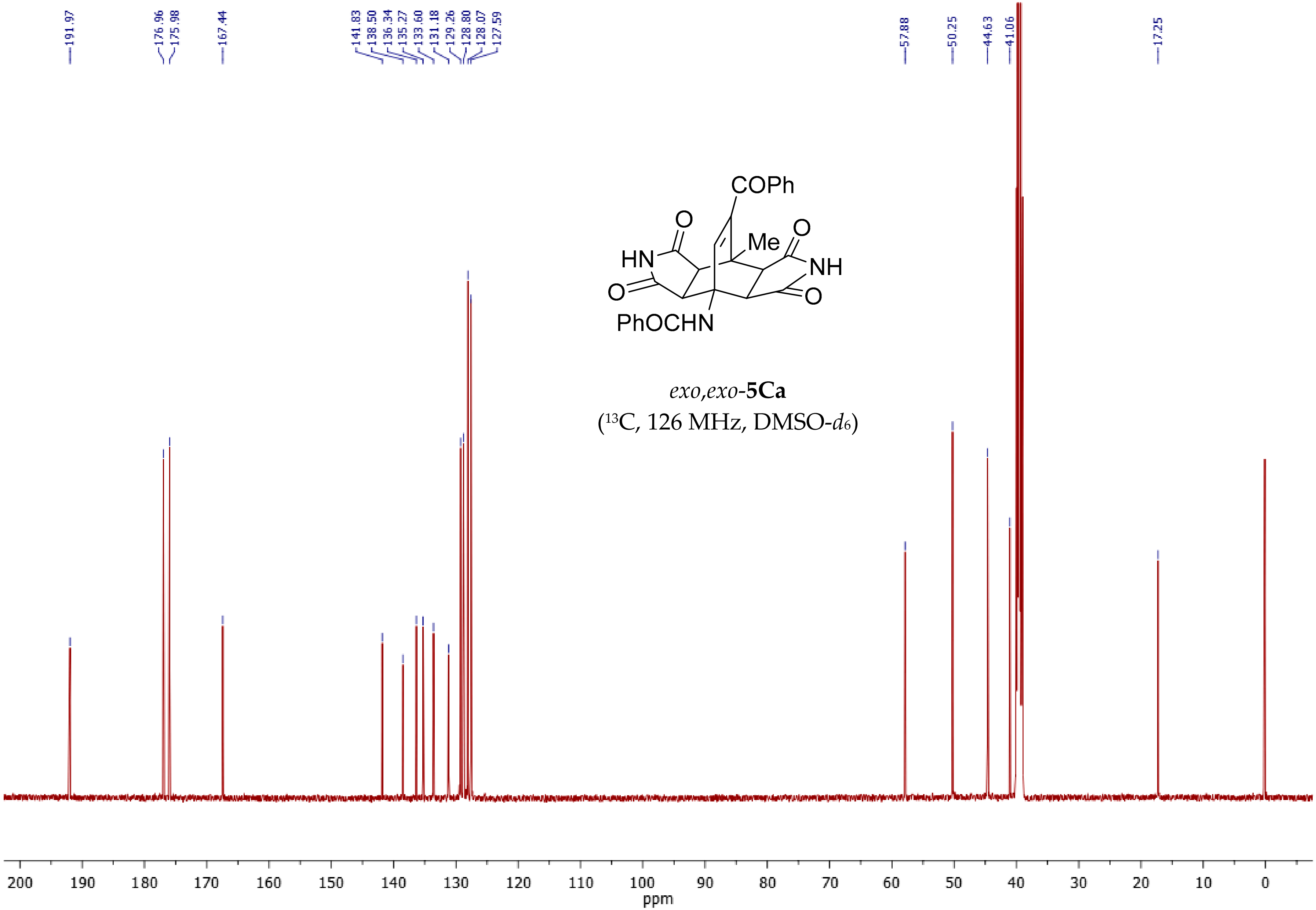
191.97
176.96
175.98
167.44
141.83
138.50
136.34
135.27
133.60
131.18
129.26
128.80
128.07
127.59
57.88
50.25
44.63
41.06
17.25
COPh
O
Me
O
HN
NH
O
O
PhOCHN
exo,exo-5Ca
(13C, 126 MHz, DMSO-d6)
200
190
180
170
160
150
140
130
120
110
100
90
80
70
60
50
40
30
20
10
0
ppm

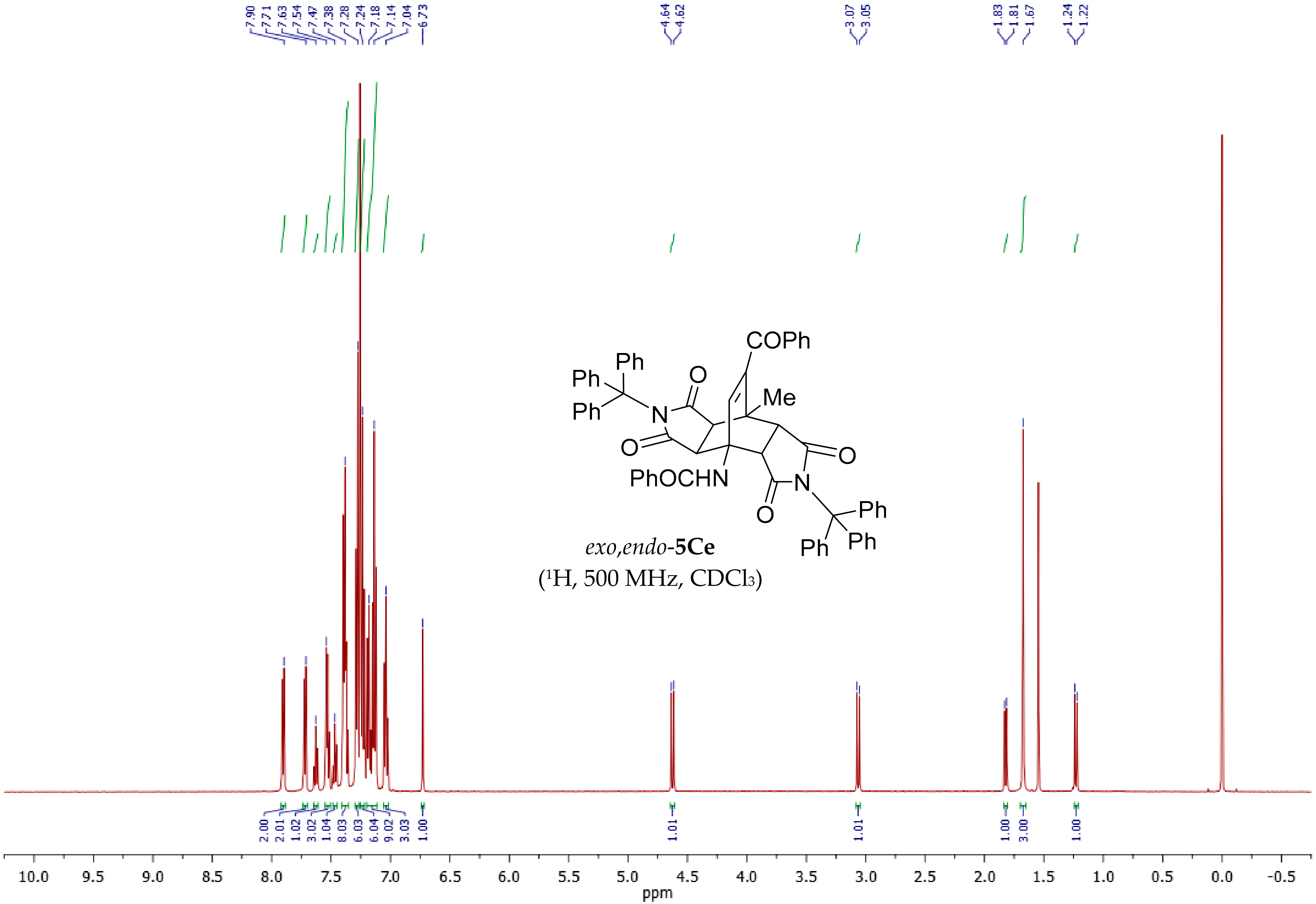

exo,endo-**5Ce**

(^{1}H, 500 MHz, $CDCl_3$)

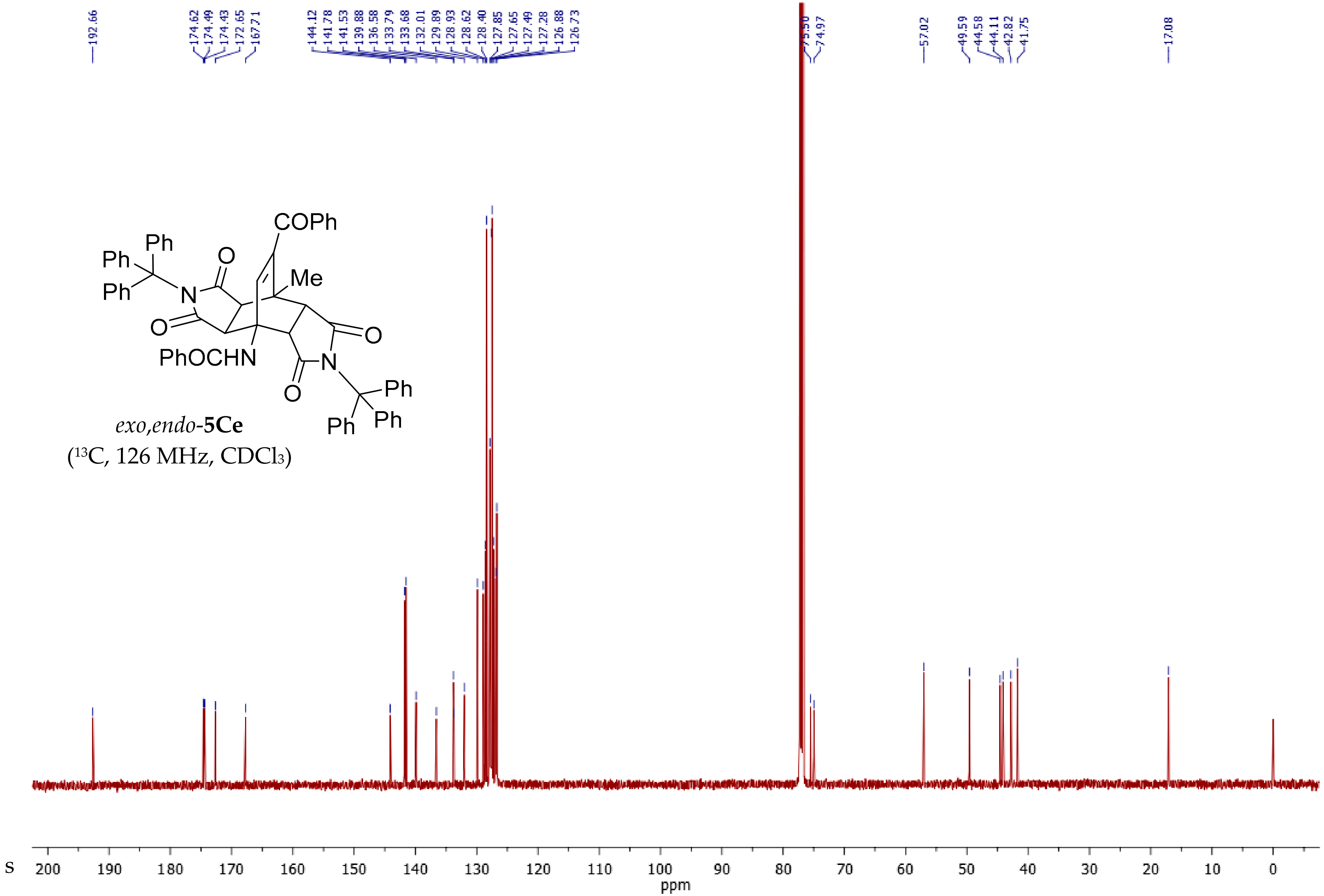

exo,endo-**5Ce**
(^{13}C, 126 MHz, $CDCl_3$)

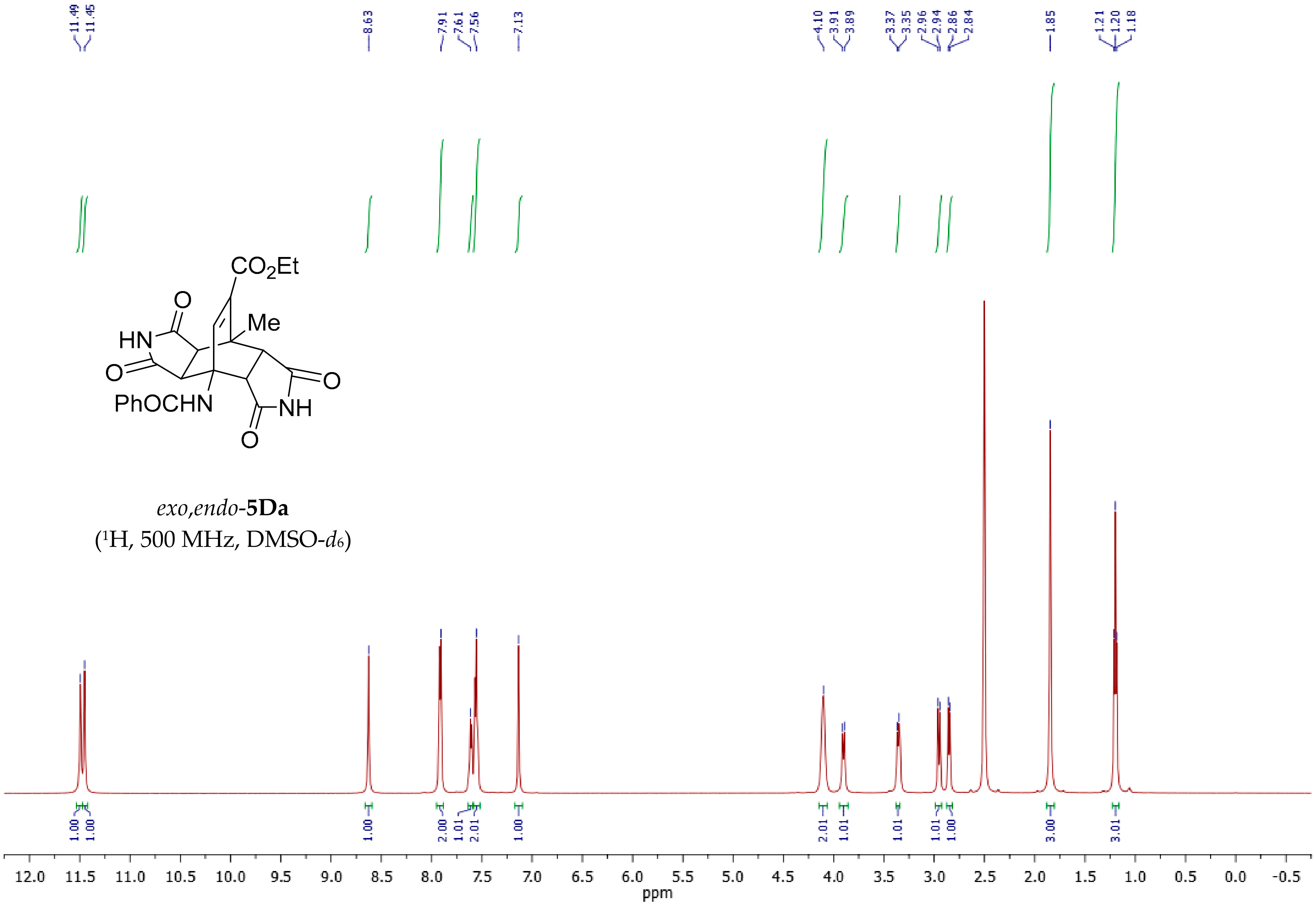

exo,endo-**5Da**

(^{1}H, 500 MHz, DMSO-d_6)

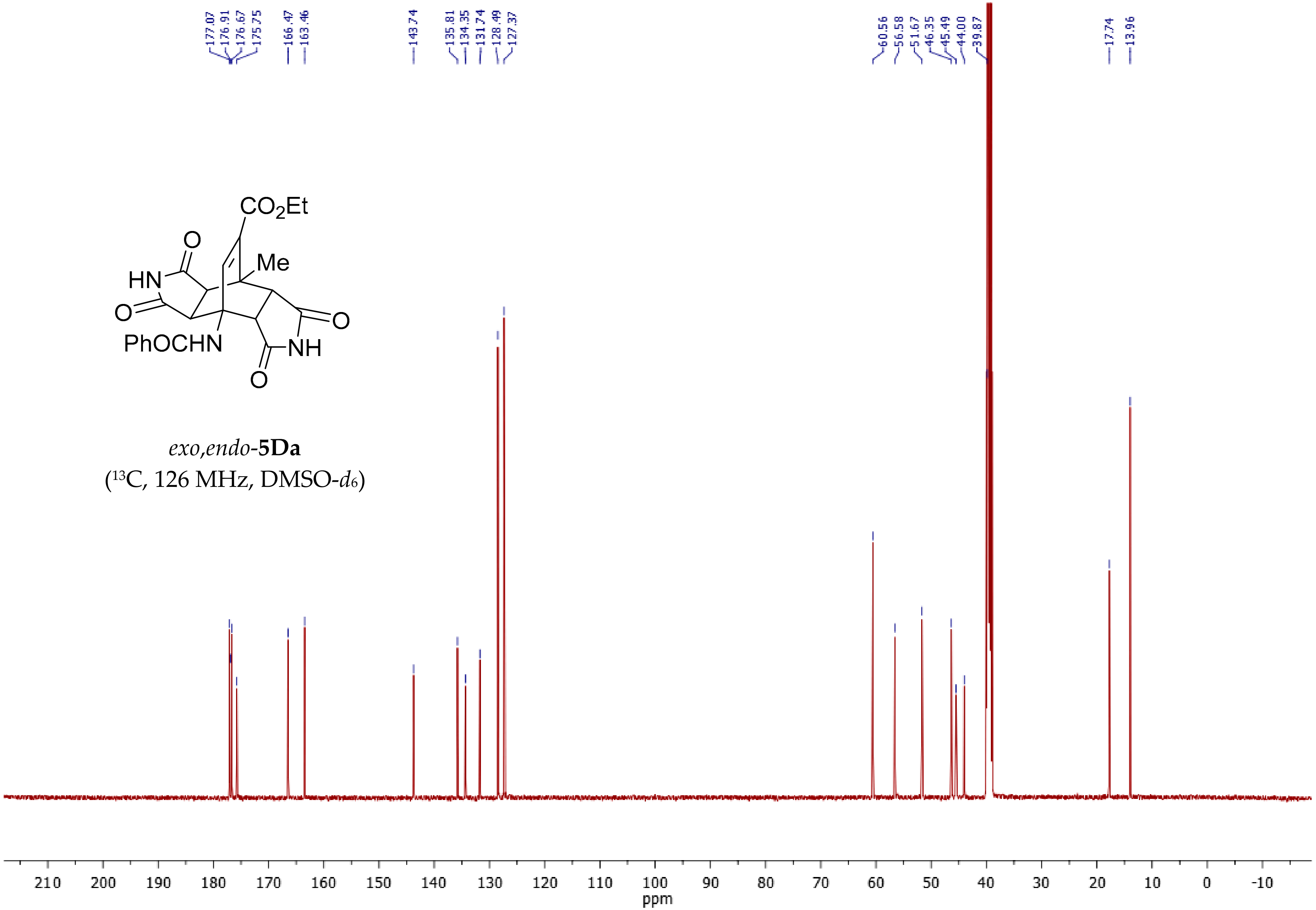

exo,endo-**5Da**
(^{13}C, 126 MHz, DMSO-d_6)

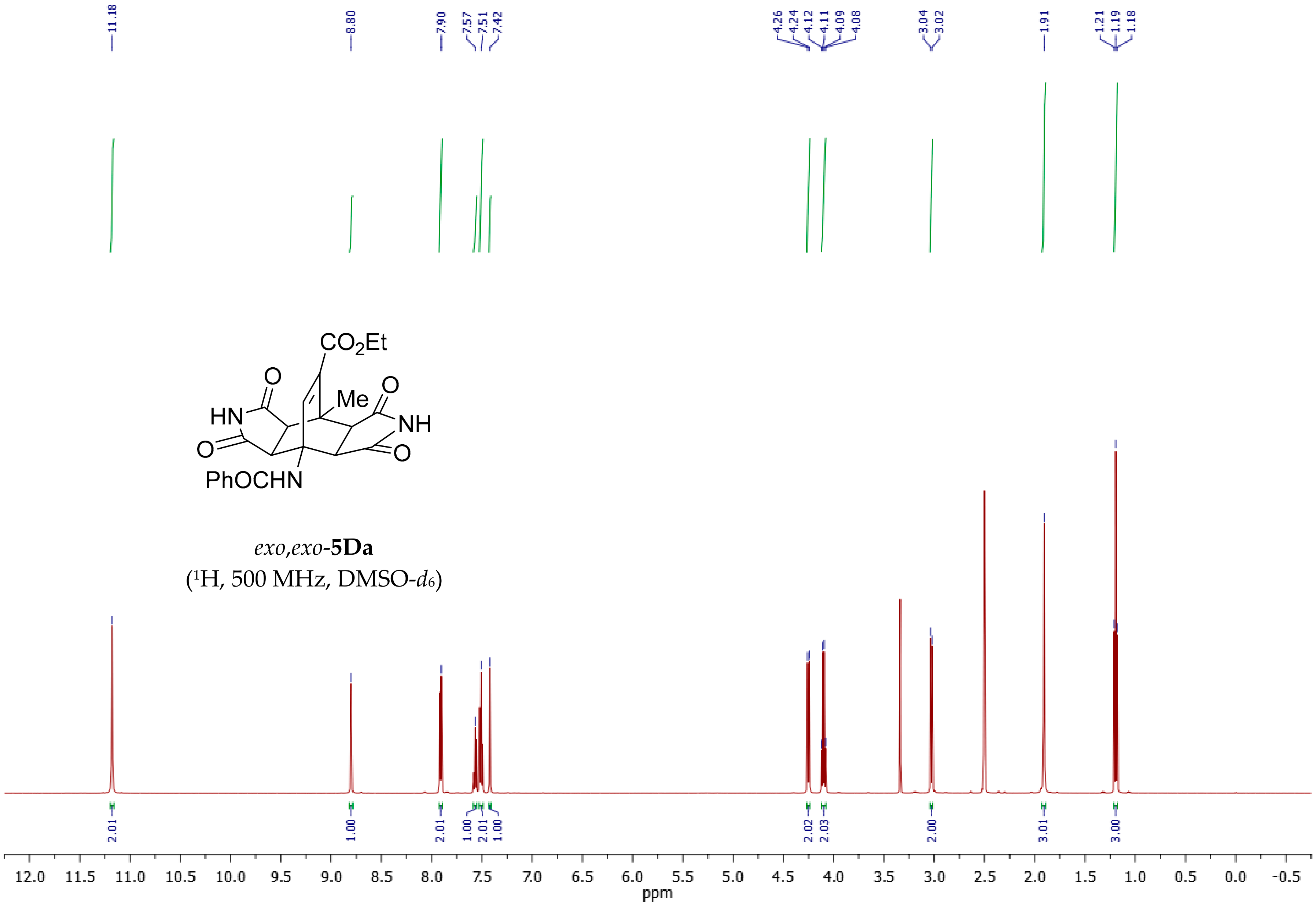

exo,exo-**5Da**
(^{1}H, 500 MHz, DMSO-d_6)

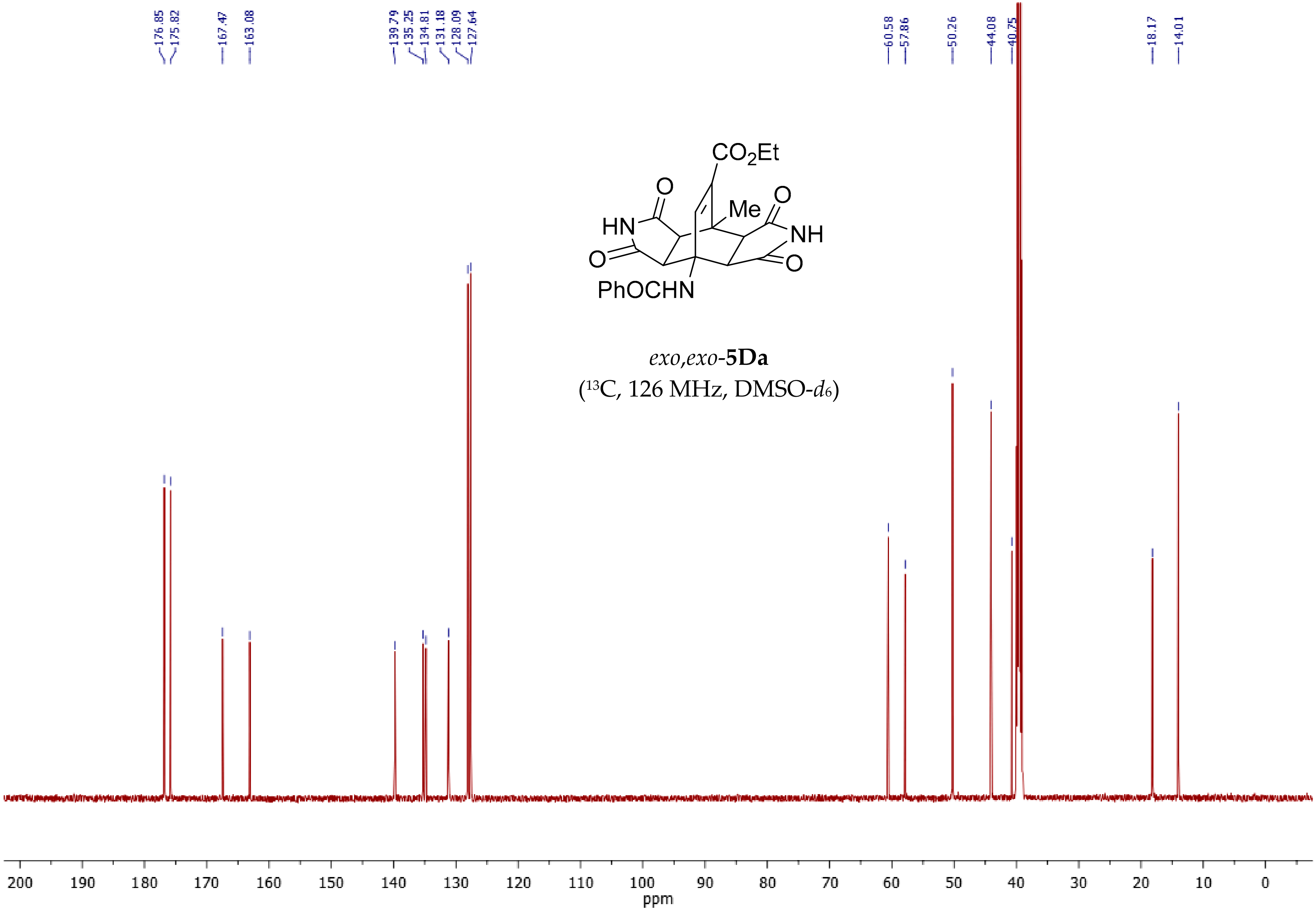

exo,exo-**5Da**
(^{13}C, 126 MHz, DMSO-d_6)

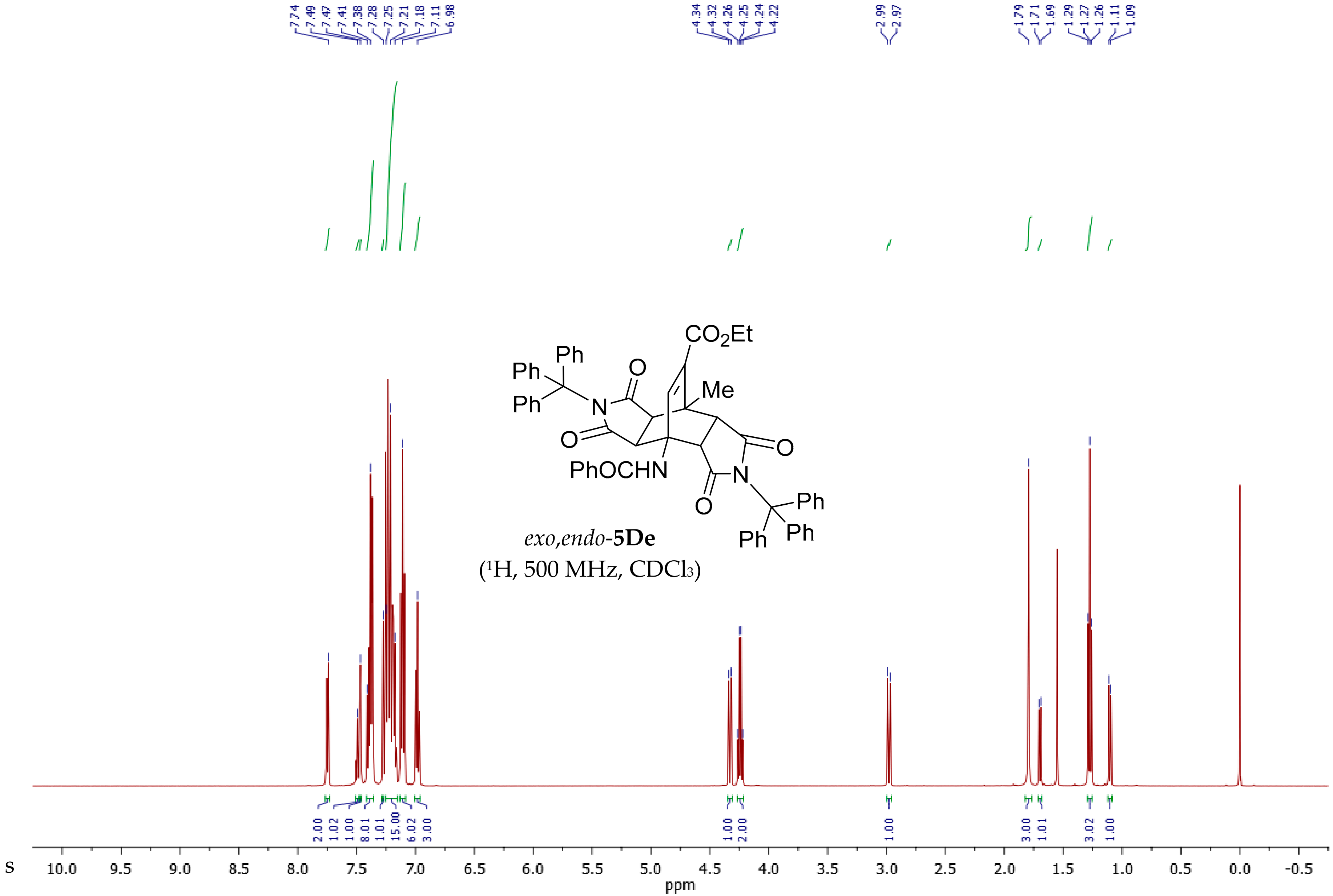

7.74
7.49
7.47
7.41
7.38
7.28
7.25
7.21
7.18
7.11
6.98
4.34
4.32
4.26
4.25
4.24
4.22
2.99
2.97
1.79
1.71
1.69
1.29
1.27
1.26
1.11
1.09
CO2Et
Ph
Ph
Ph
O
N
Me
O
O
PhOCHN
N
O
Ph
Ph
Ph
exo,endo-5De
(1H, 500 MHz, CDCl3)
2.00
1.02
1.00
8.01
1.01
15.00
6.02
3.00
1.00
2.00
1.00
3.00
1.01
3.02
1.00
S
10.0
9.5
9.0
8.5
8.0
7.5
7.0
6.5
6.0
5.5
5.0
4.5
4.0
3.5
3.0
2.5
2.0
1.5
1.0
0.5
0.0
-0.5
ppm

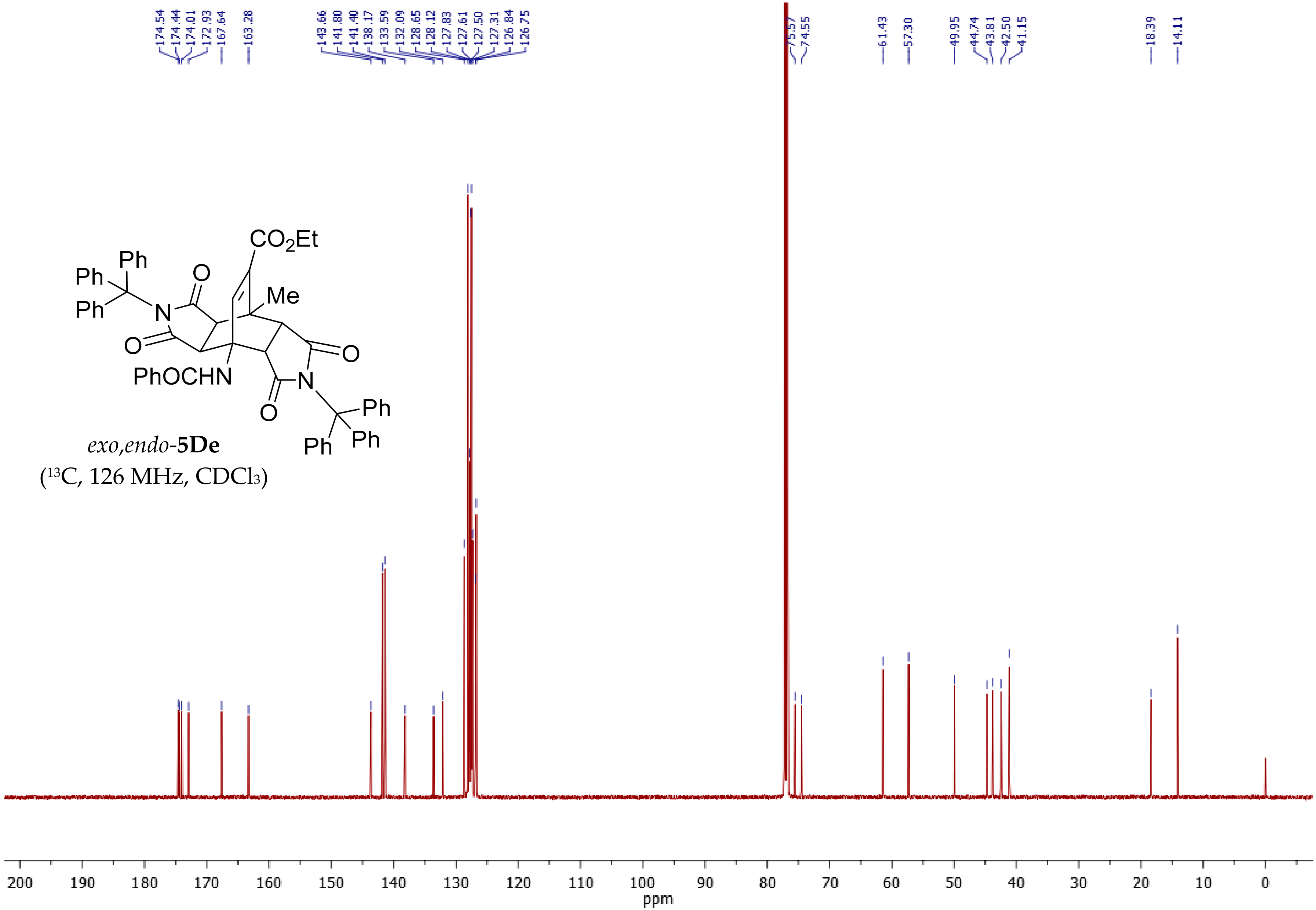

exo,endo-**5De**
(^{13}C, 126 MHz, $CDCl_3$)

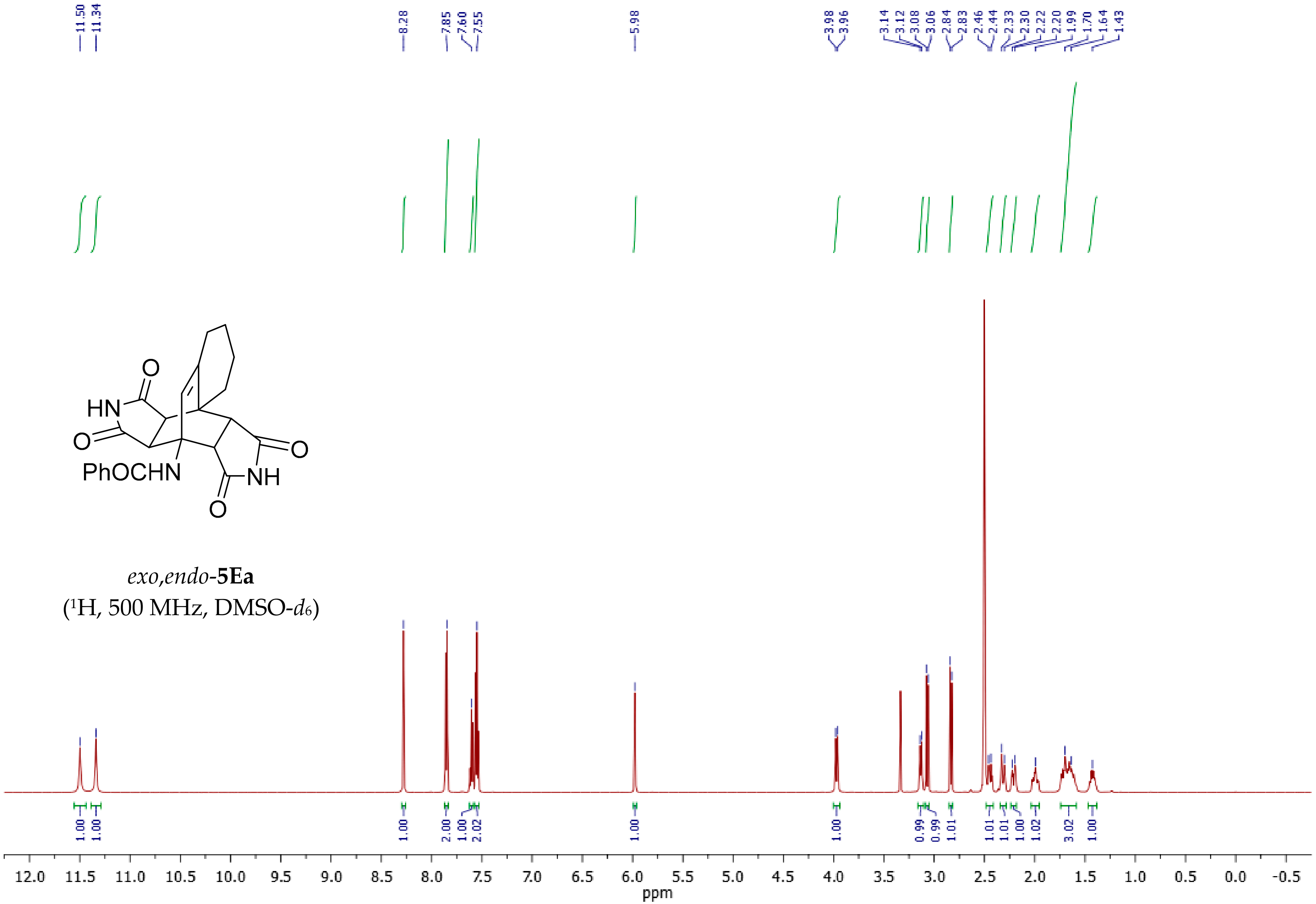

exo,endo-**5Ea**

(^{1}H, 500 MHz, DMSO-d_6)

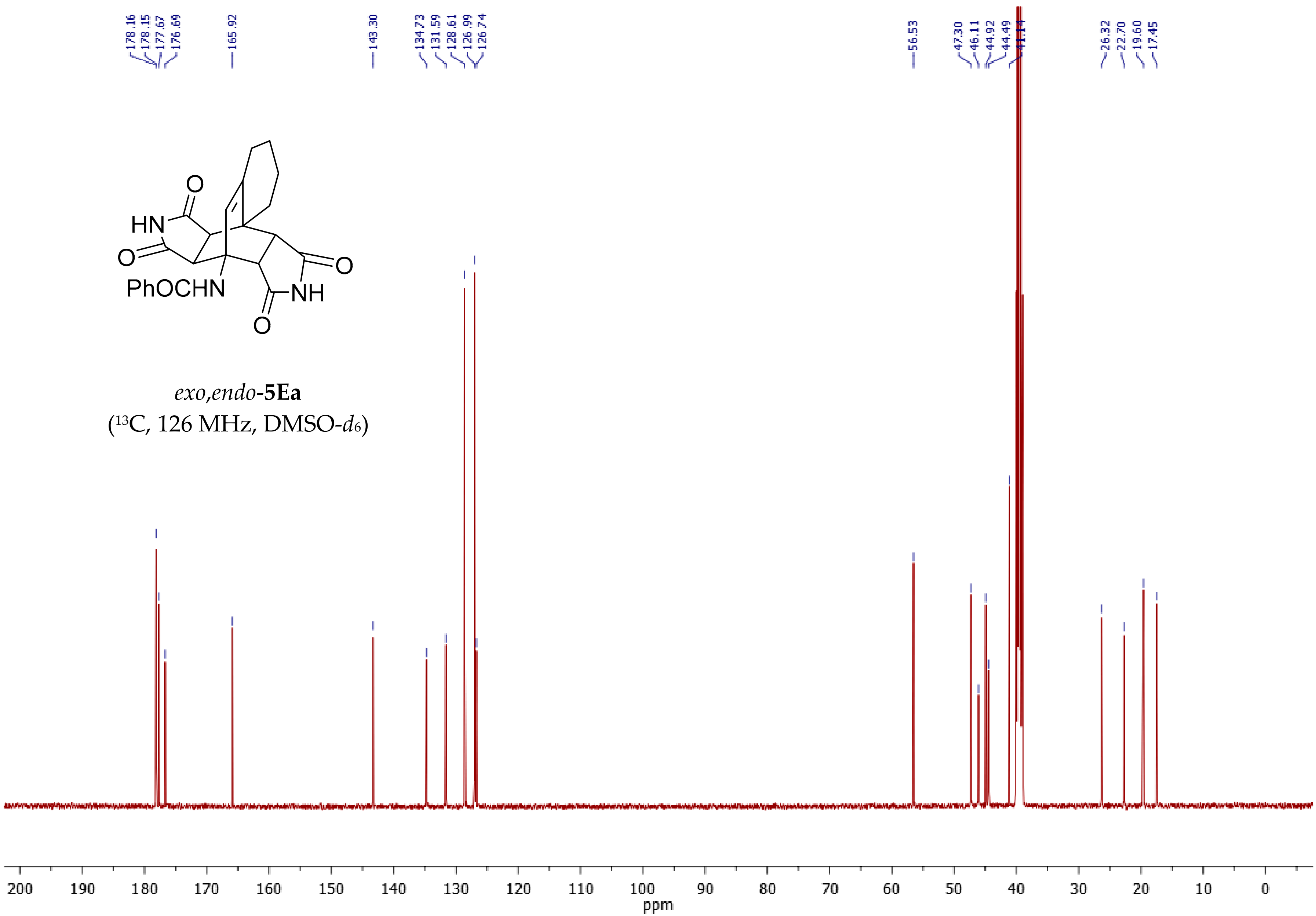

exo,endo-**5Ea**

(^{13}C, 126 MHz, DMSO-d_6)

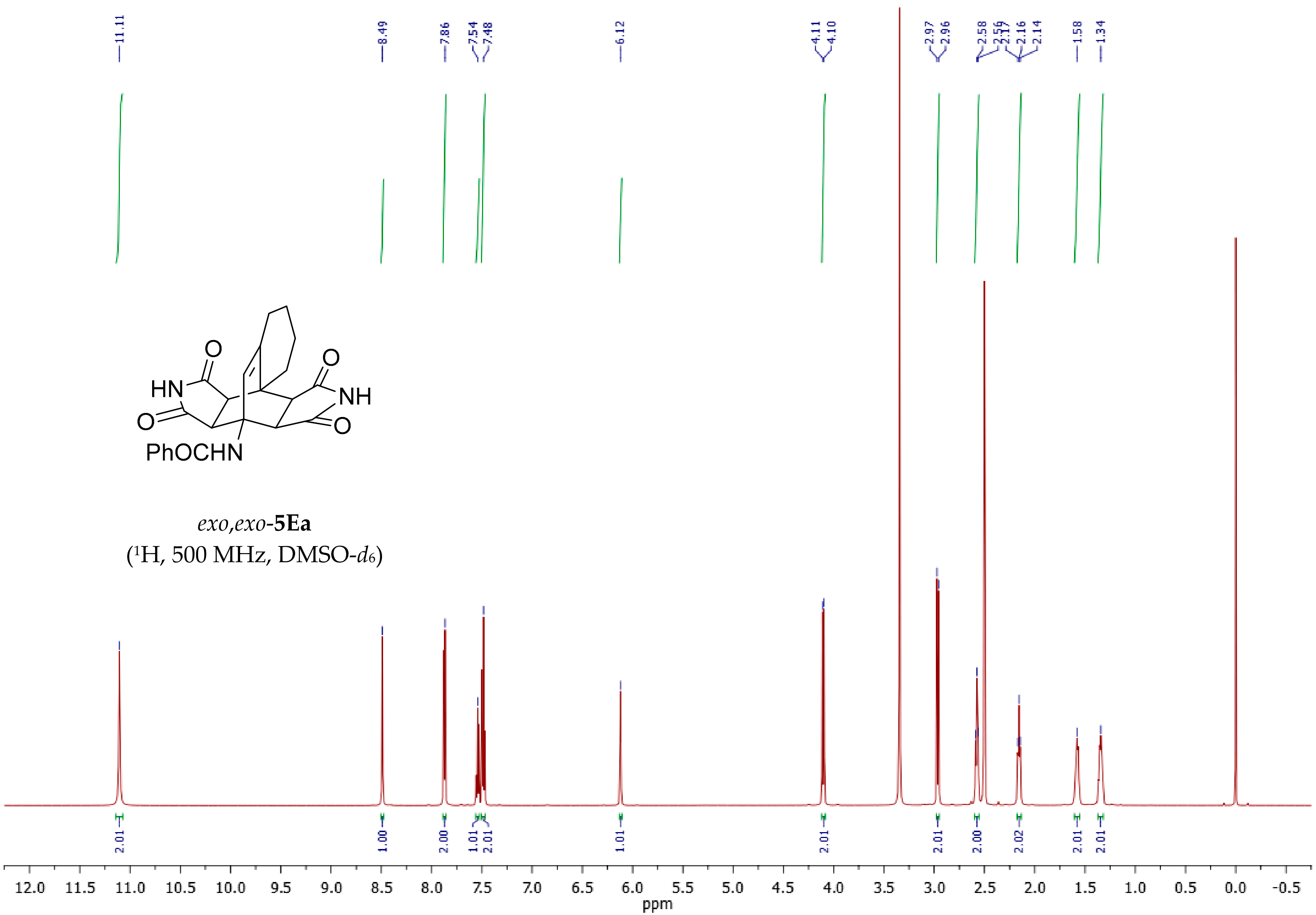

exo,exo-**5Ea**

(^{1}H, 500 MHz, DMSO-d_6)

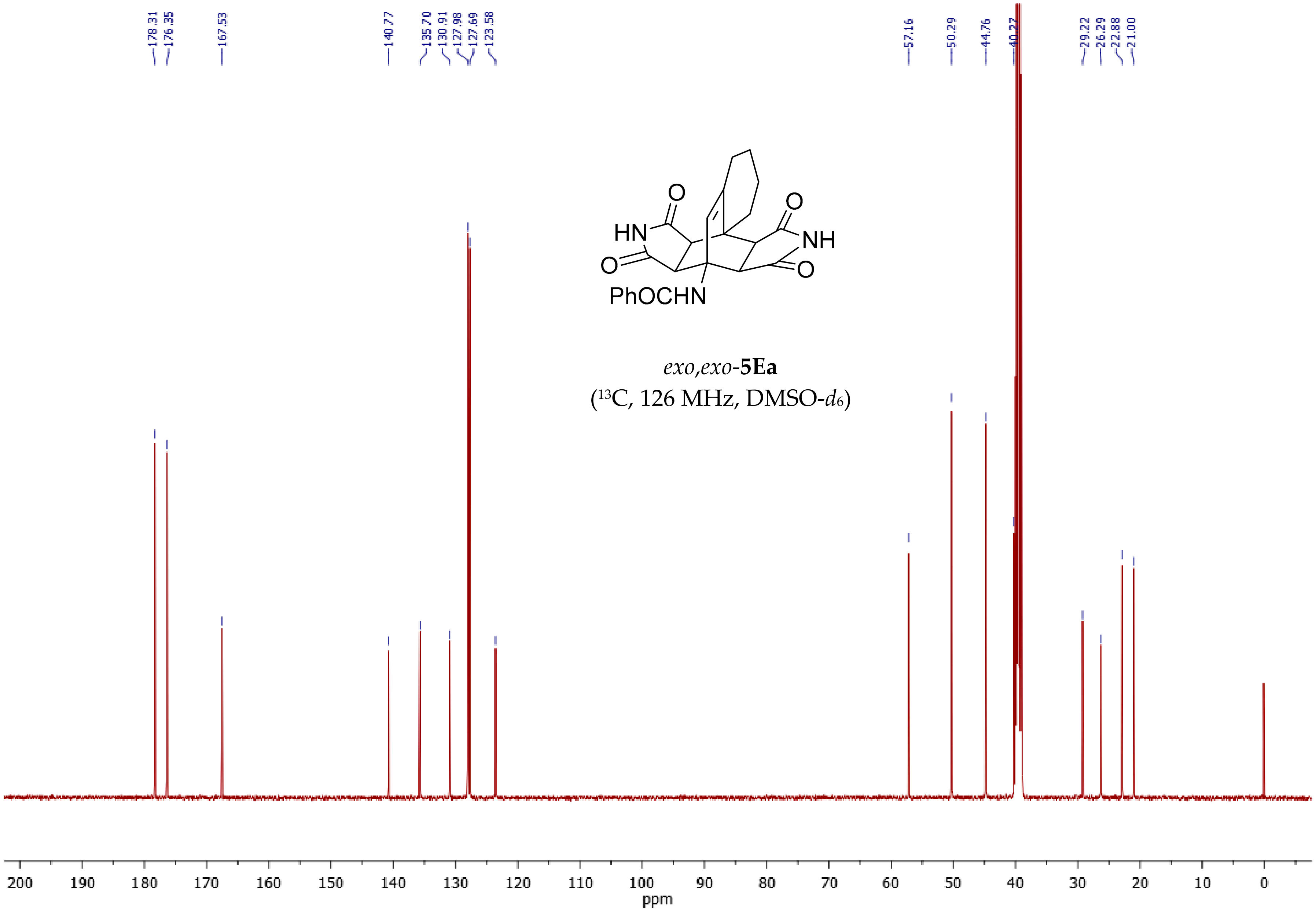
178.31
176.35
167.53
140.77
135.70
130.91
127.98
127.69
123.58
57.16
50.29
44.76
40.27
29.22
26.29
22.88
21.00
O
HN
O
PhOCHN
O
NH
O
exo,exo-5Ea
(13C, 126 MHz, DMSO-d6)
200
190
180
170
160
150
140
130
120
110
100
90
80
70
60
50
40
30
20
10
0
ppm

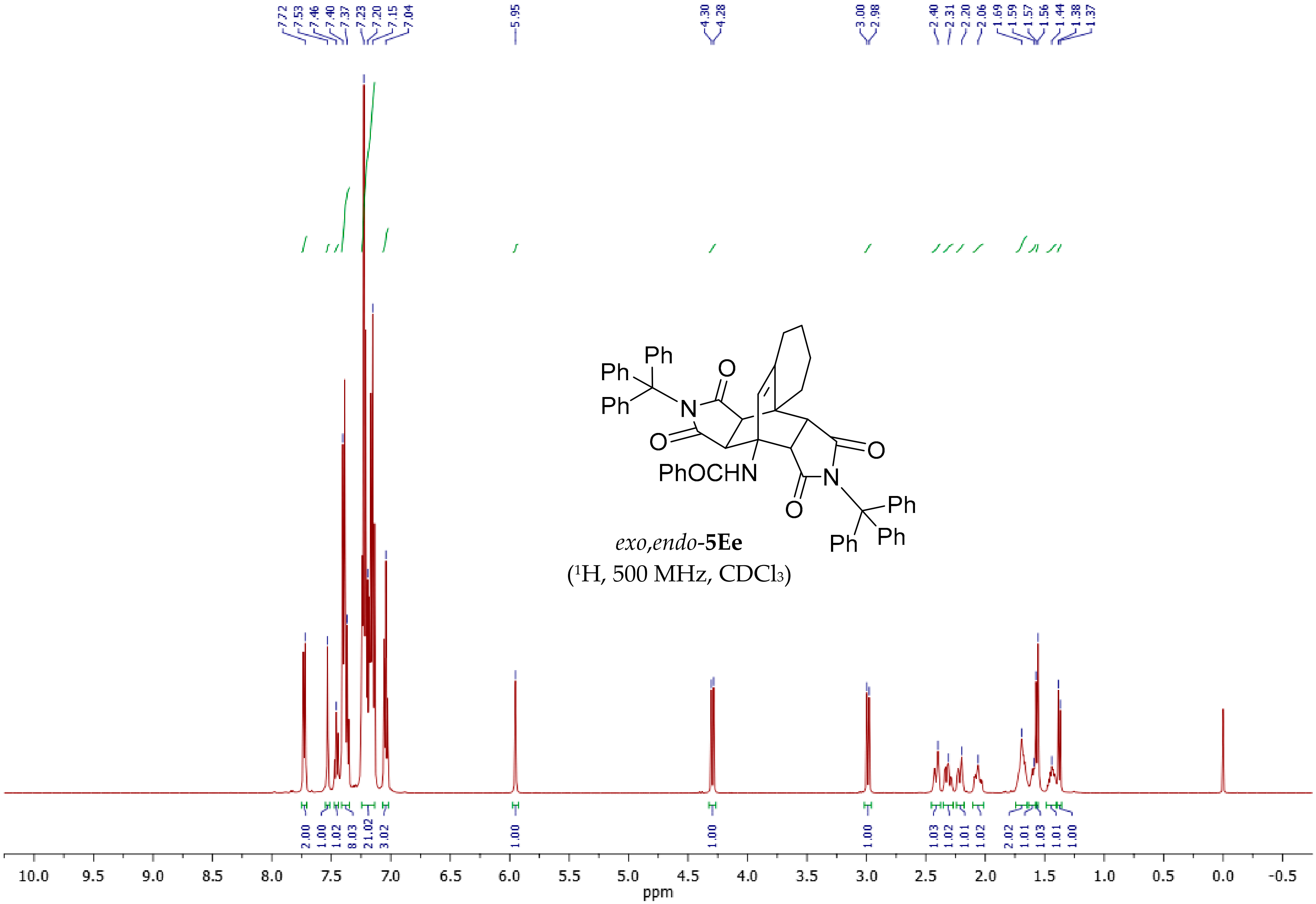

exo,endo-**5Ee**
(^{1}H, 500 MHz, $CDCl_3$)

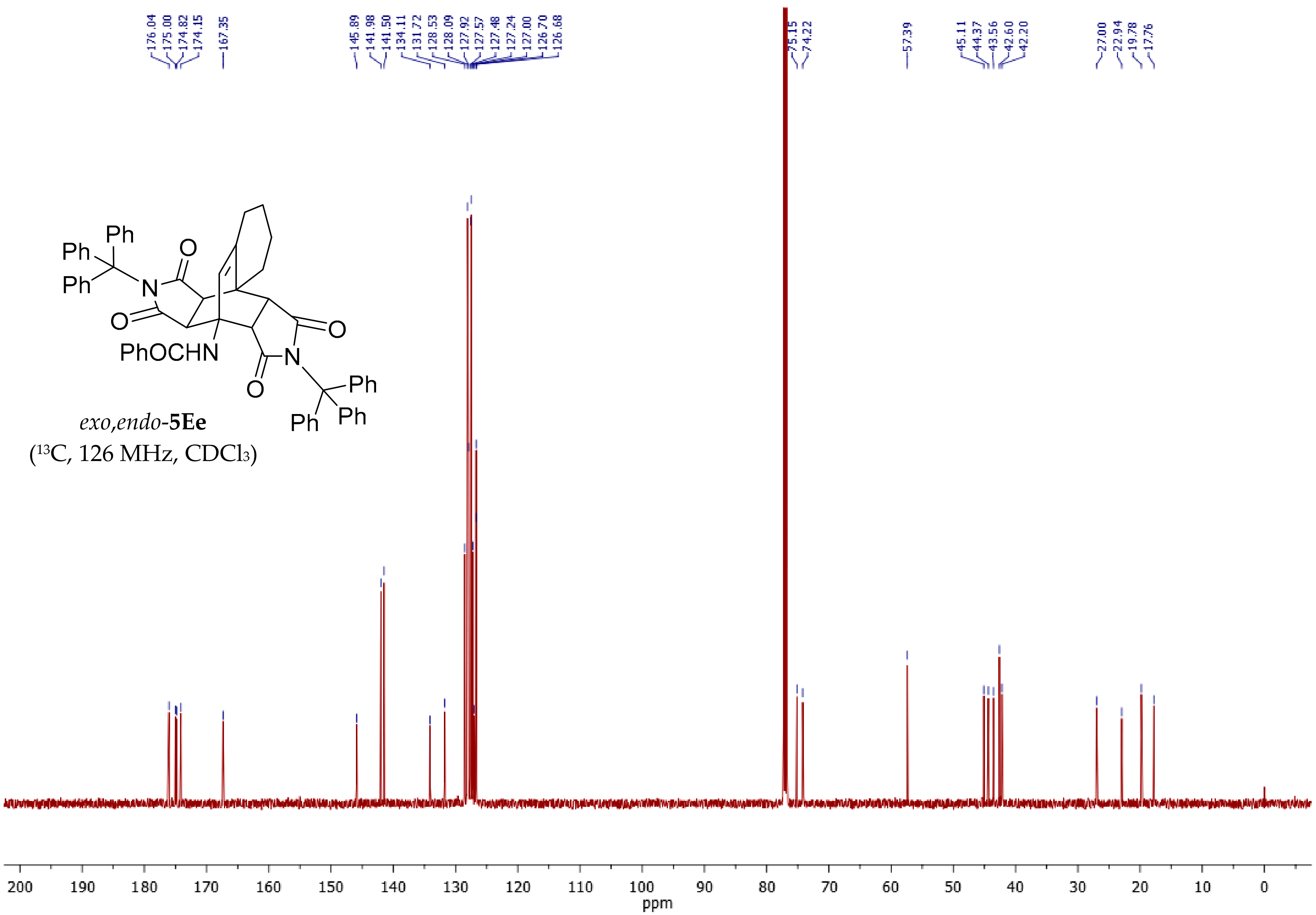

exo,endo-**5Ee**

(^{13}C, 126 MHz, $CDCl_3$)

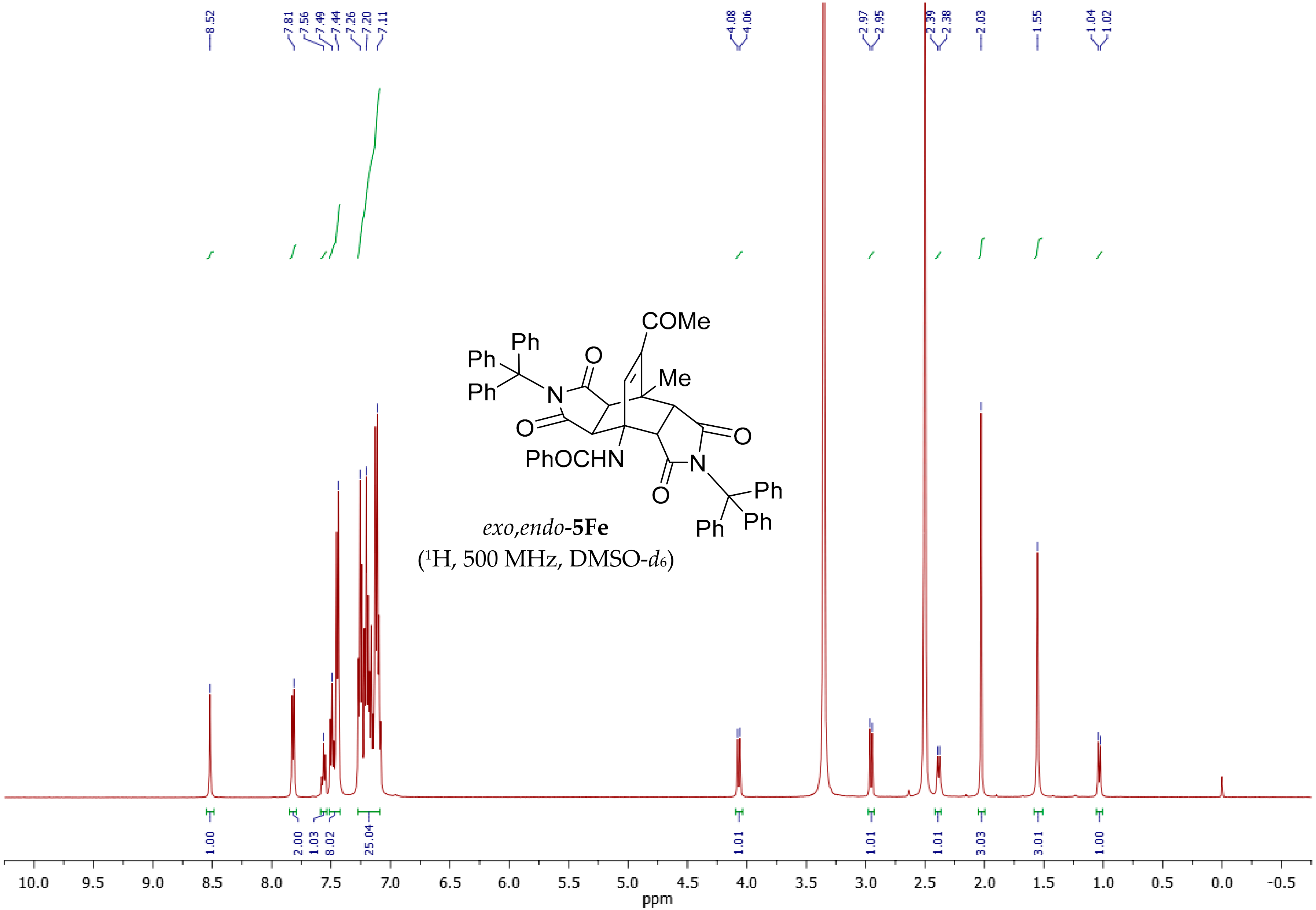

exo,endo-**5Fe**
(^{1}H, 500 MHz, DMSO-d_6)

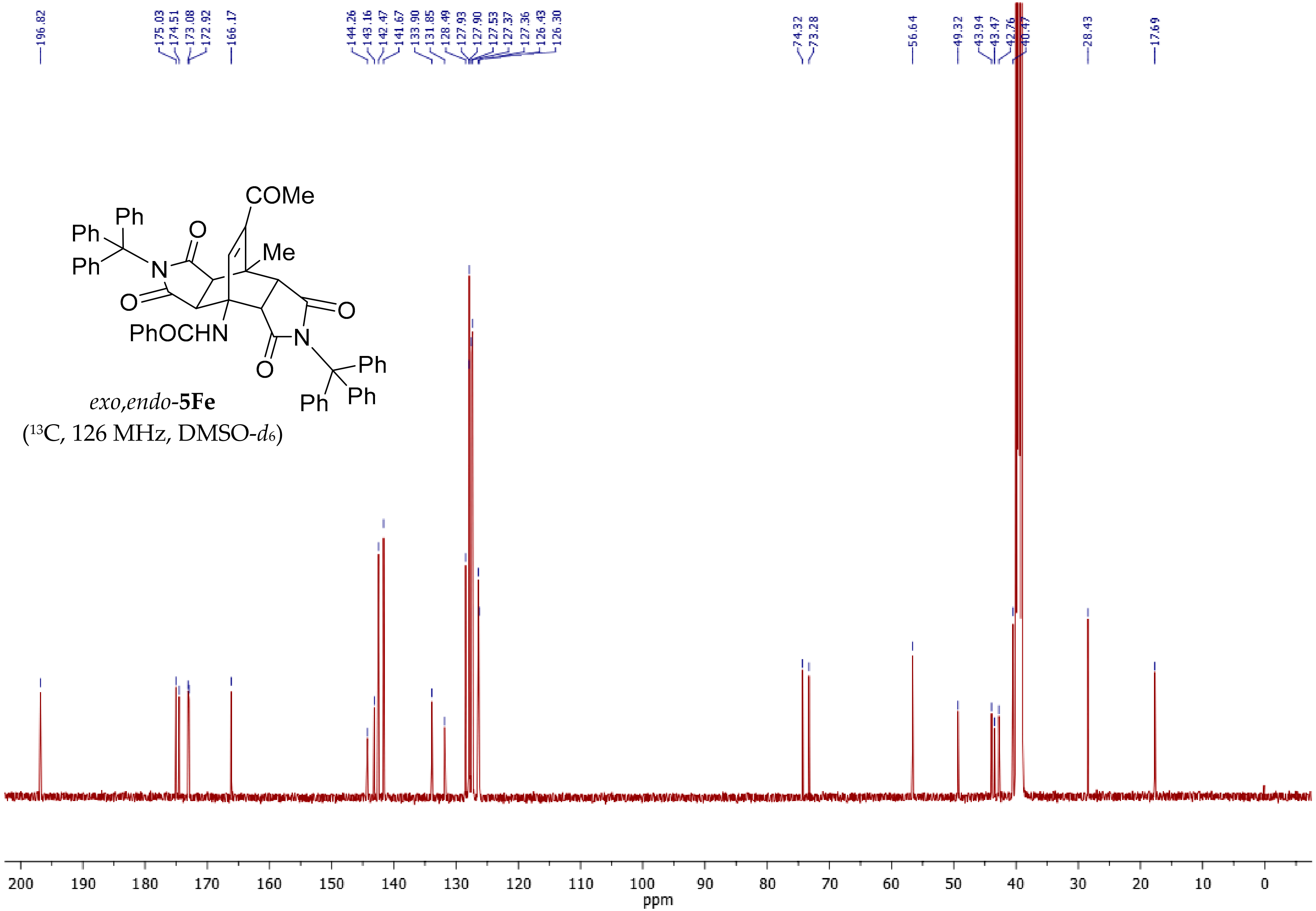

exo,endo-**5Fe**

(^{13}C, 126 MHz, DMSO-d_6)

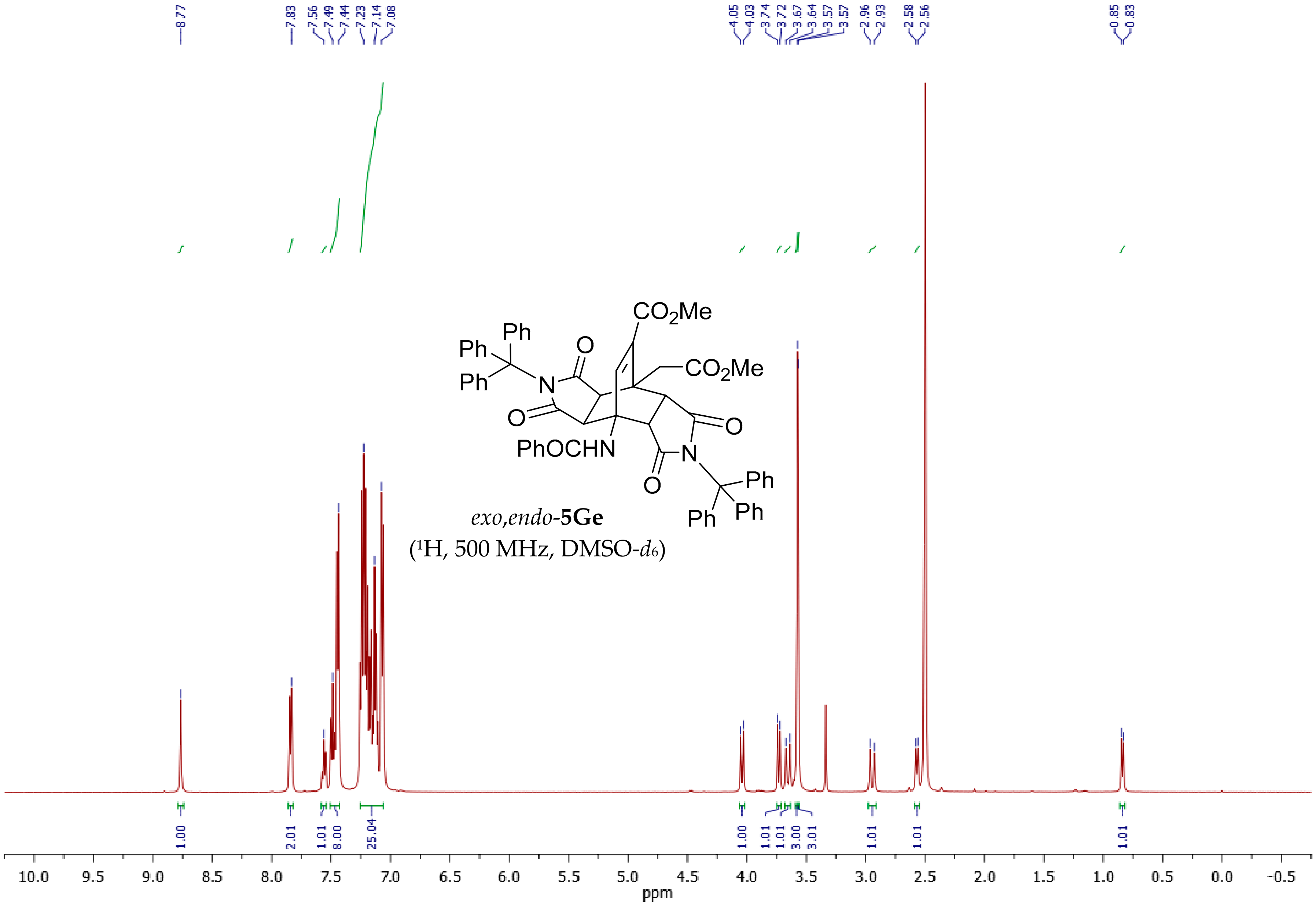

exo,endo-**5Ge**

(^{1}H, 500 MHz, DMSO-d_6)

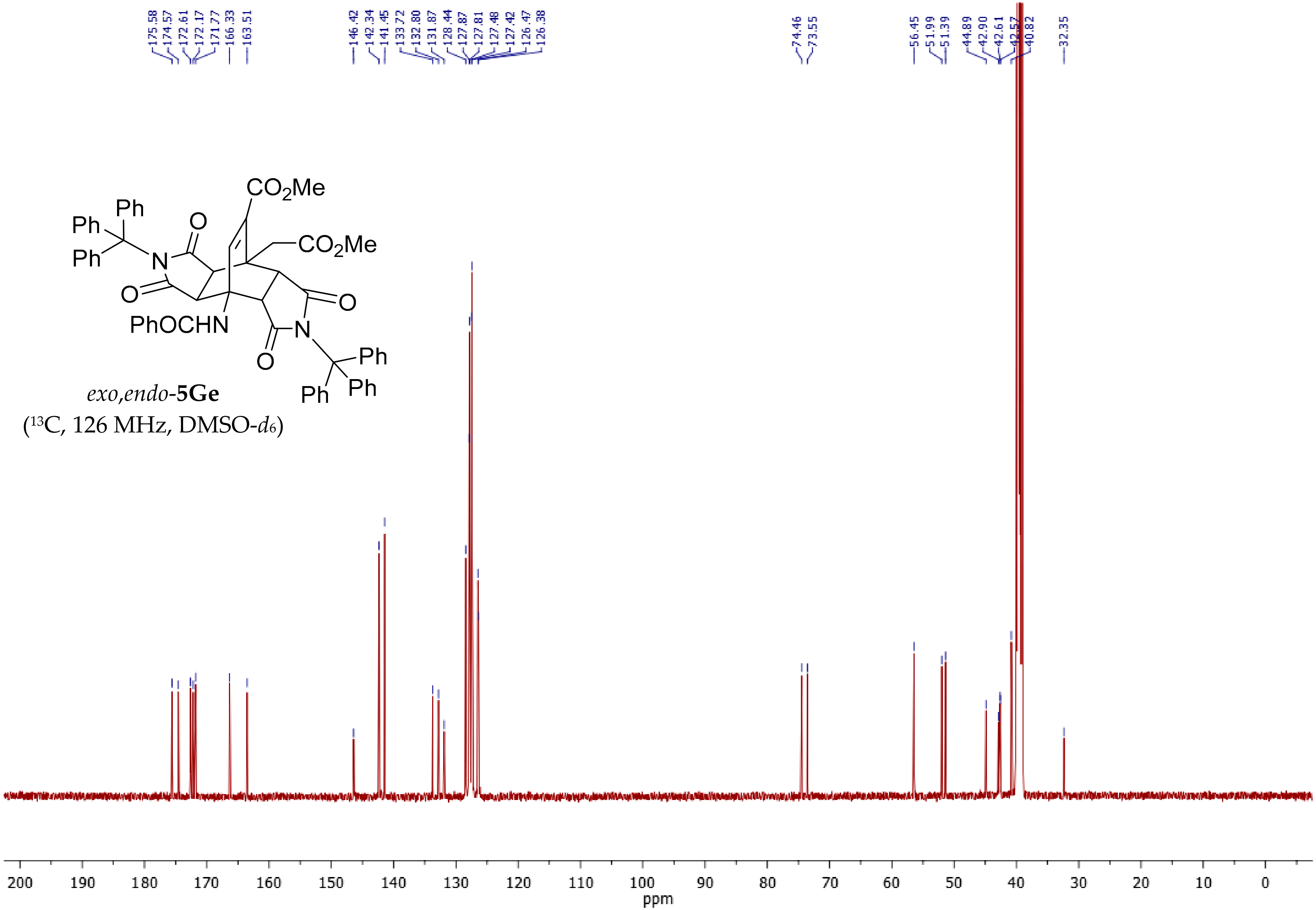
175.58
174.57
172.61
172.17
171.77
166.33
163.51
146.42
142.34
141.45
133.72
132.80
131.87
128.44
127.87
127.81
127.48
127.42
126.47
126.38
74.46
73.55
56.45
51.99
51.39
44.89
42.90
42.61
42.57
40.82
32.35
CO2Me
CO2Me
Ph
Ph
Ph
O
N
O
PhOCHN
O
N
O
Ph
Ph
Ph
exo,endo-**5Ge**
(^{13}C, 126 MHz, DMSO-d_6)
200
190
180
170
160
150
140
130
120
110
100
90
80
70
60
50
40
30
20
10
0
ppm

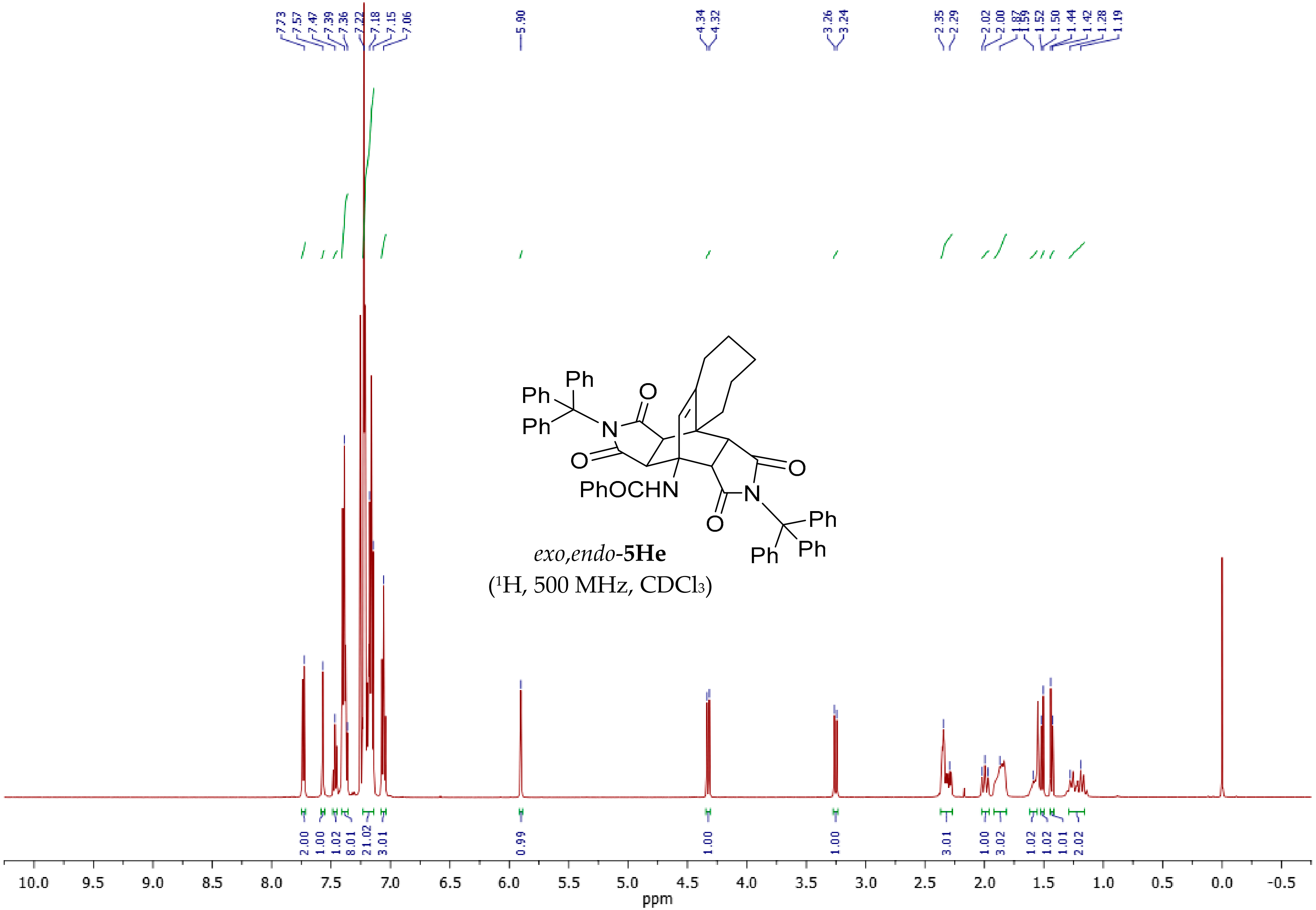

7.73
7.57
7.47
7.39
7.36
7.22
7.18
7.15
7.06
5.90
4.34
4.32
3.26
3.24
2.35
2.29
2.02
2.00
1.87
1.56
1.52
1.50
1.44
1.42
1.28
1.19
Ph
Ph
Ph
O
N
O
PhOCHN
O
N
O
Ph
Ph
Ph
exo,endo-5He
(1H, 500 MHz, CDCl3)
2.00
1.00
1.02
8.01
21.02
3.01
0.99
1.00
1.00
3.01
1.00
3.02
1.02
1.02
1.01
2.02
10.0 9.5 9.0 8.5 8.0 7.5 7.0 6.5 6.0 5.5 5.0 4.5 4.0 3.5 3.0 2.5 2.0 1.5 1.0 0.5 0.0 -0.5
ppm

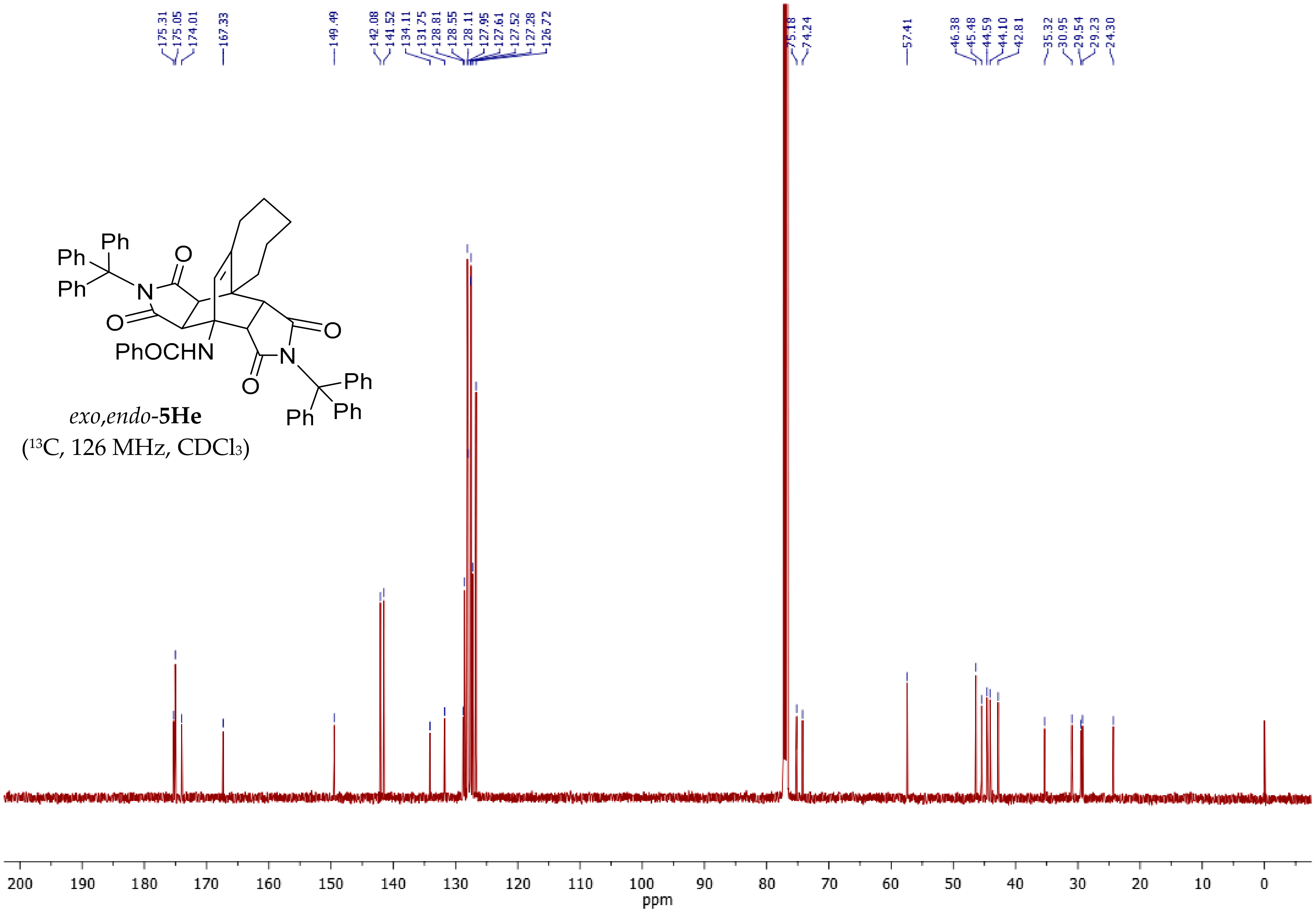

exo,endo-**5He**
(^{13}C, 126 MHz, $CDCl_3$)

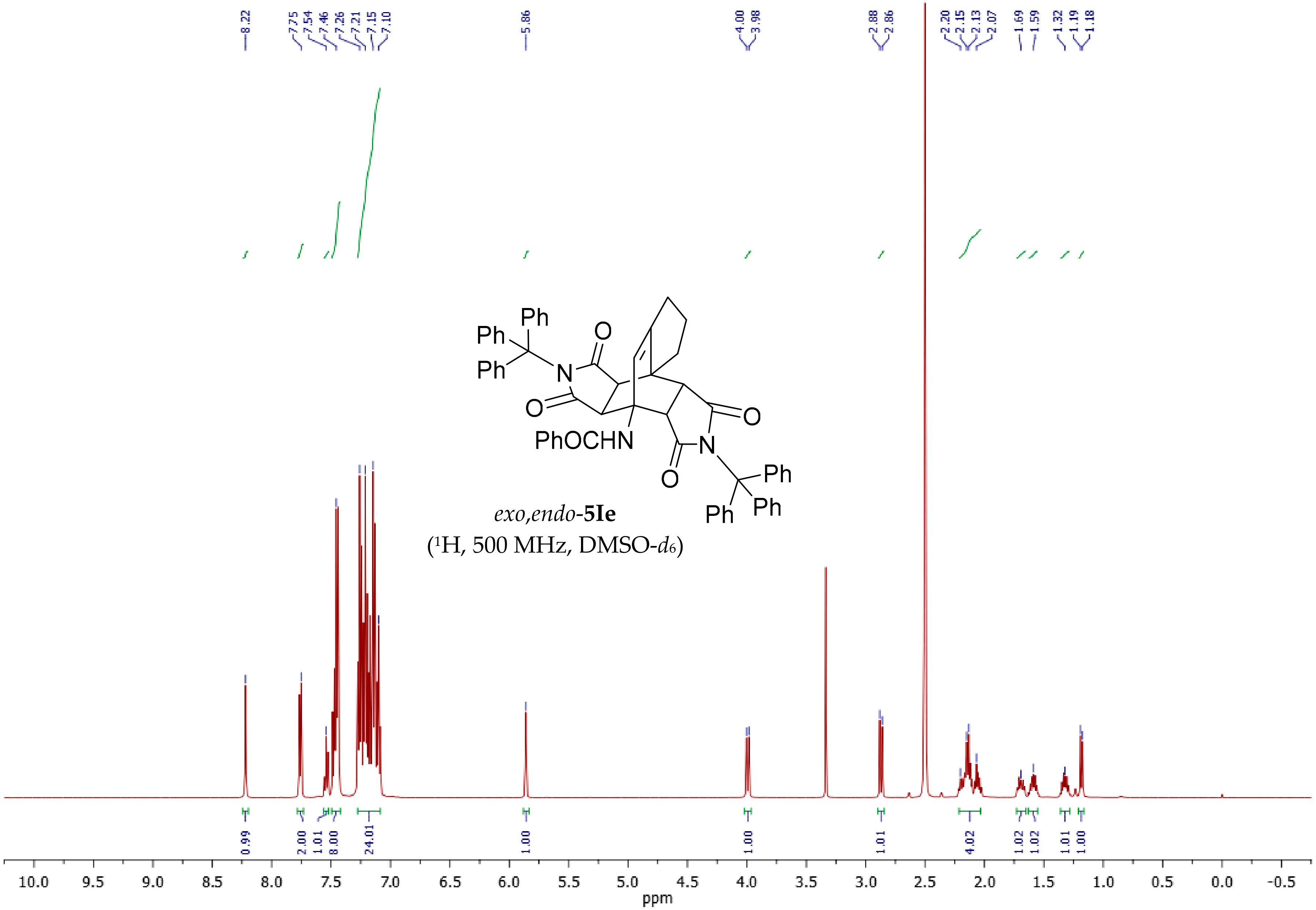

exo,endo-**5Ie**
(^{1}H, 500 MHz, DMSO-d_6)

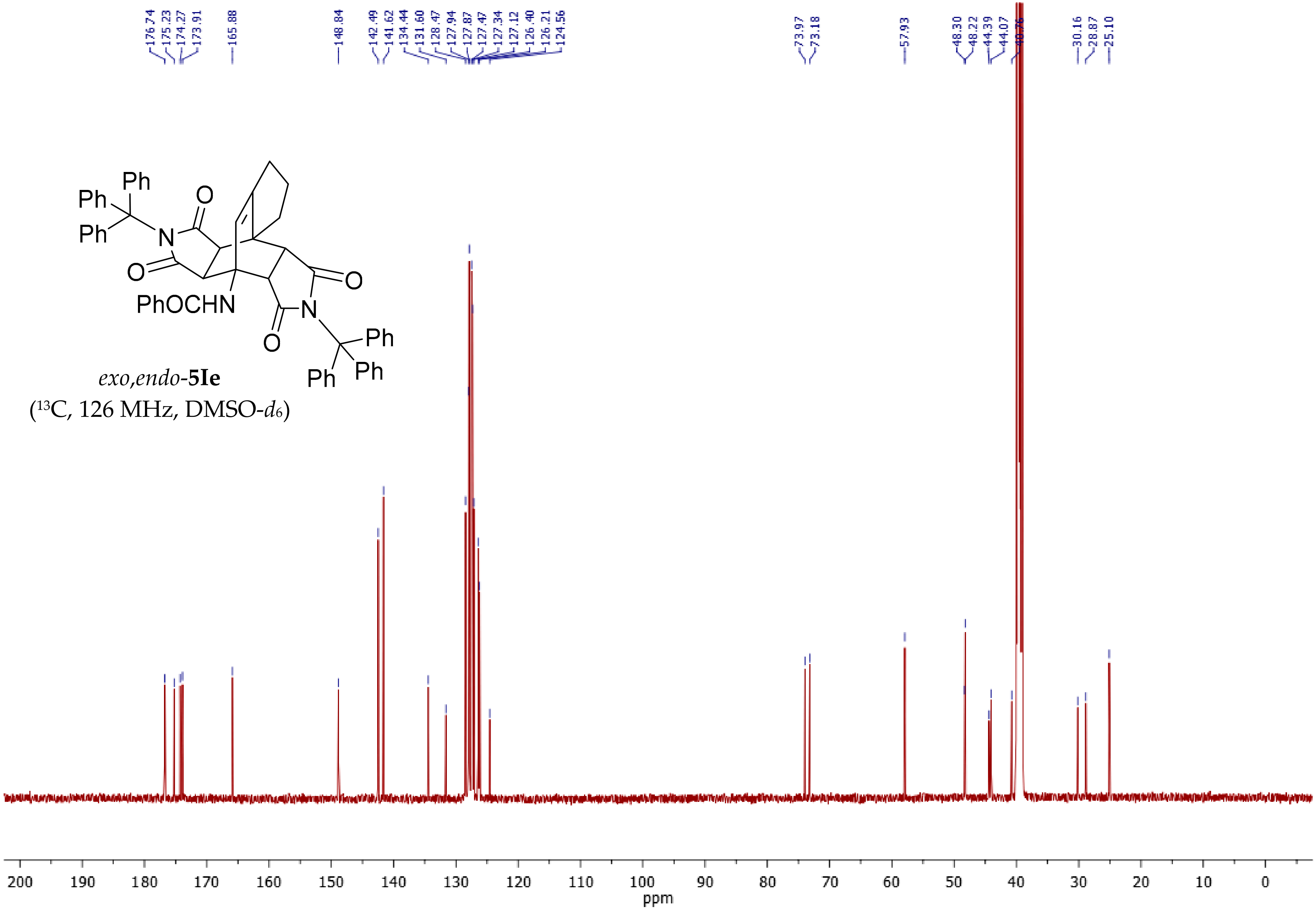
176.74
175.23
174.27
173.91
165.88
148.84
142.49
141.62
134.44
131.60
128.47
127.94
127.87
127.47
127.34
127.12
126.40
126.21
124.56
73.97
73.18
57.93
48.30
48.22
44.39
44.07
30.16
28.87
25.10
Ph
Ph
Ph
O
N
O
PhOCHN
O
N
O
Ph
Ph
Ph
exo,endo-**5Ie**
(^{13}C, 126 MHz, DMSO-d_6)
200 190 180 170 160 150 140 130 120 110 100 90 80 70 60 50 40 30 20 10 0
ppm

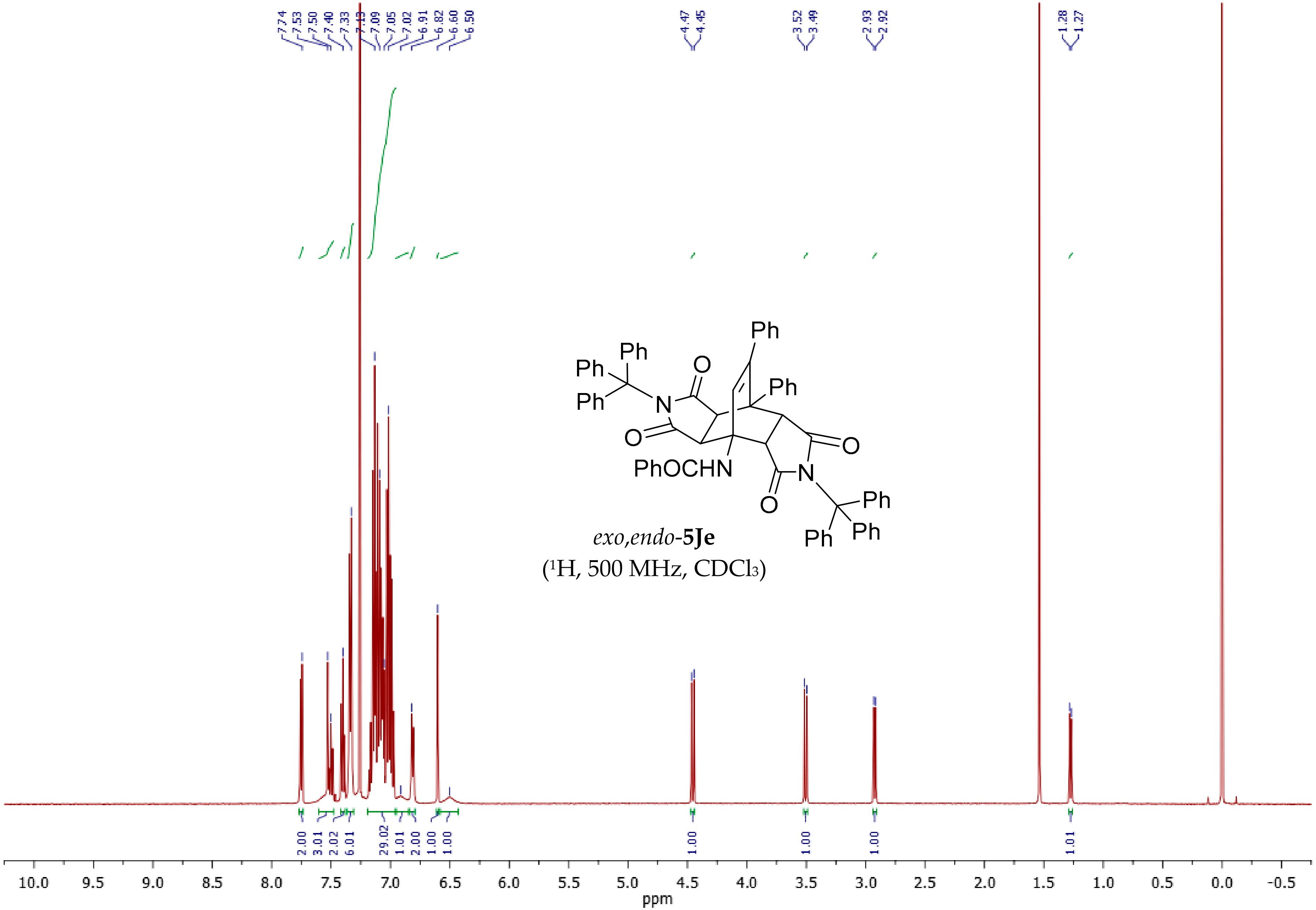

7.74
7.53
7.50
7.40
7.33
7.13
7.09
7.05
7.02
6.91
6.82
6.60
6.50
4.47
4.45
3.52
3.49
2.93
2.92
1.28
1.27
Ph
Ph
Ph
Ph
O
N
Ph
O
PhOCHN
O
N
O
Ph
Ph
Ph
exo,endo-5Je
(1H, 500 MHz, CDCl3)
2.00
3.01
2.02
6.01
29.02
1.01
2.00
1.00
1.00
1.00
1.00
1.00
1.01
10.0
9.5
9.0
8.5
8.0
7.5
7.0
6.5
6.0
5.5
5.0
4.5
4.0
3.5
3.0
2.5
2.0
1.5
1.0
0.5
0.0
-0.5
ppm

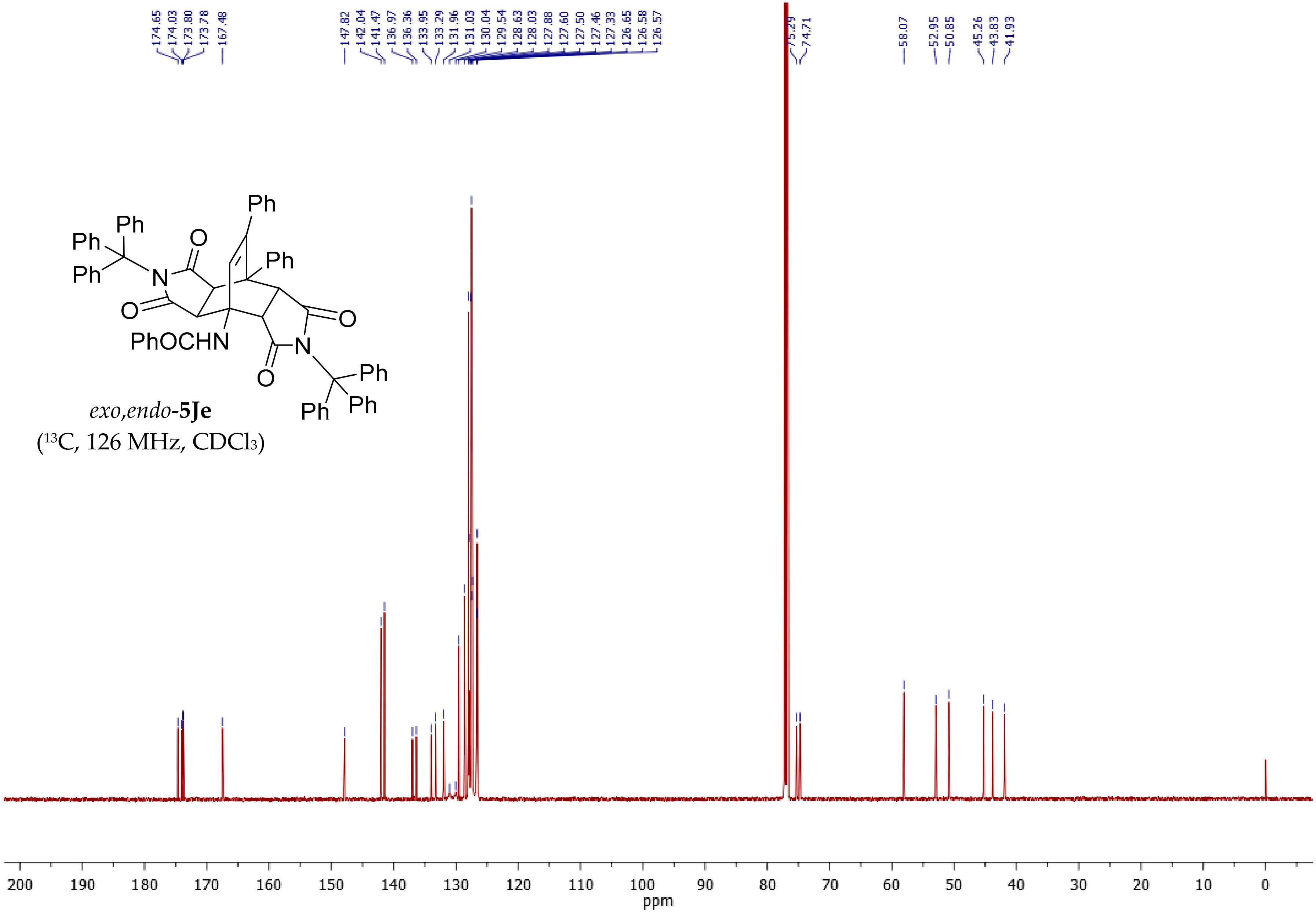

exo,endo-**5Je**
(^{13}C, 126 MHz, $CDCl_3$)

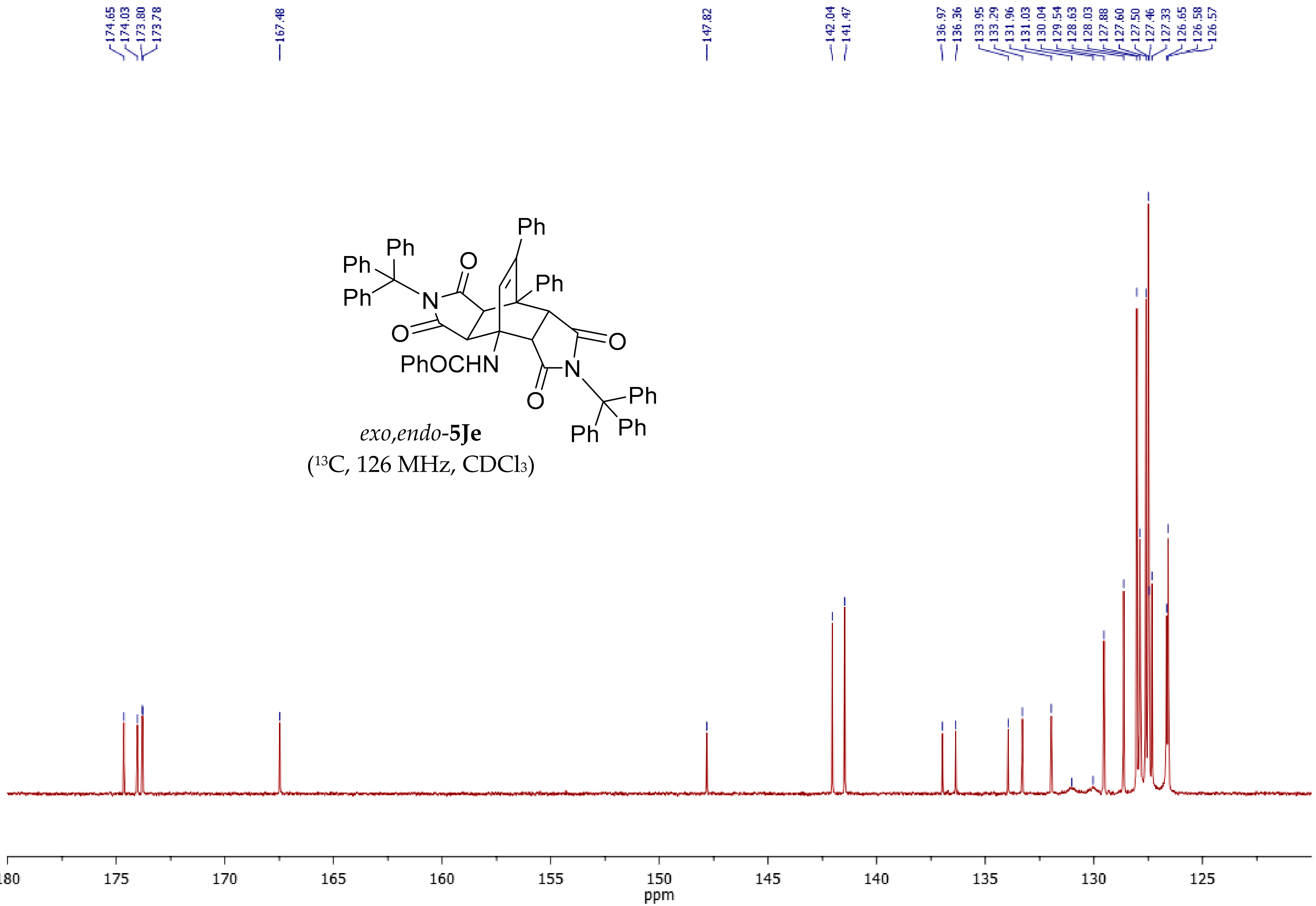

exo,endo-**5Je**

(^{13}C, 126 MHz, $CDCl_3$)

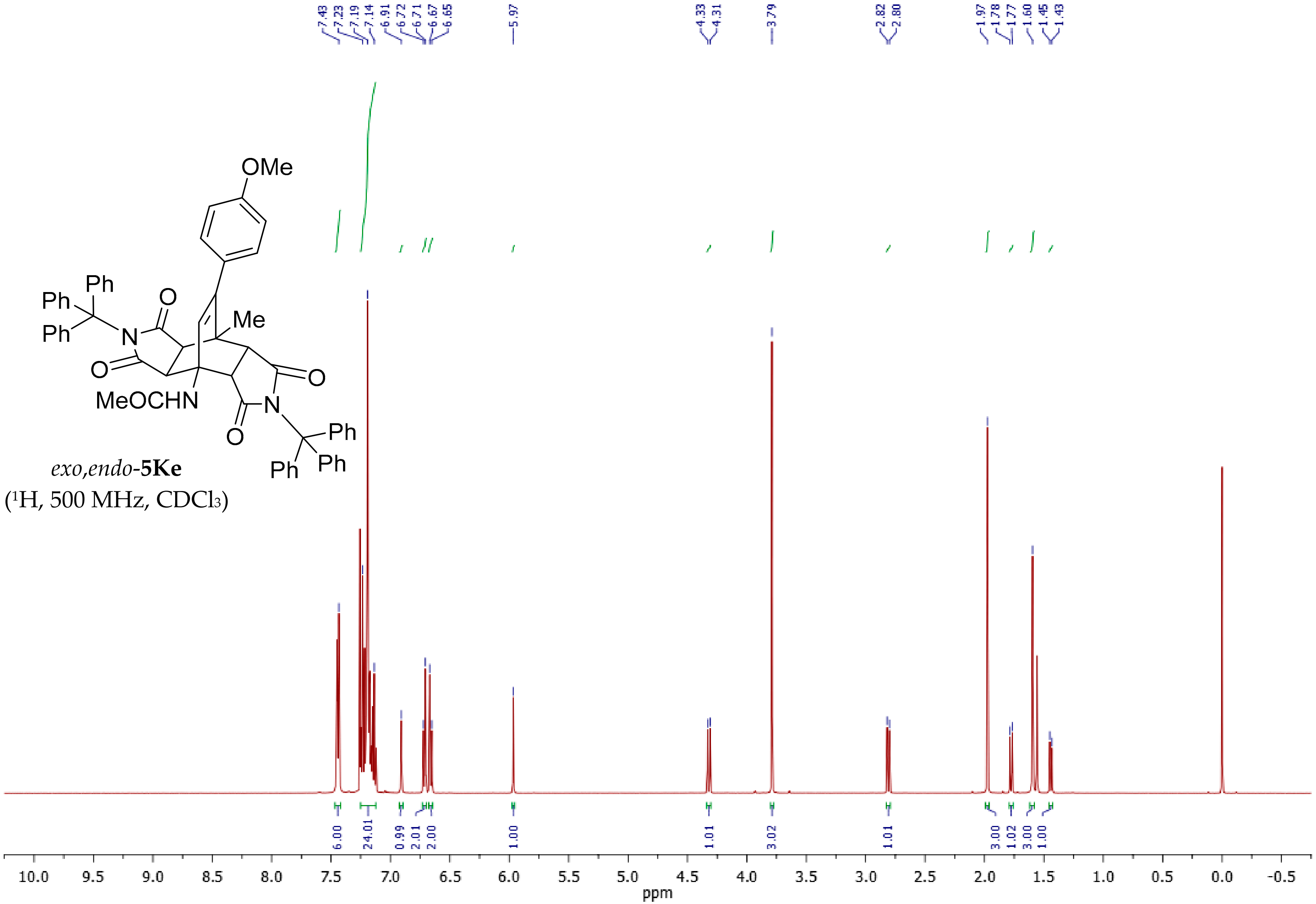

exo,endo-**5Ke**
(^{1}H, 500 MHz, $CDCl_3$)

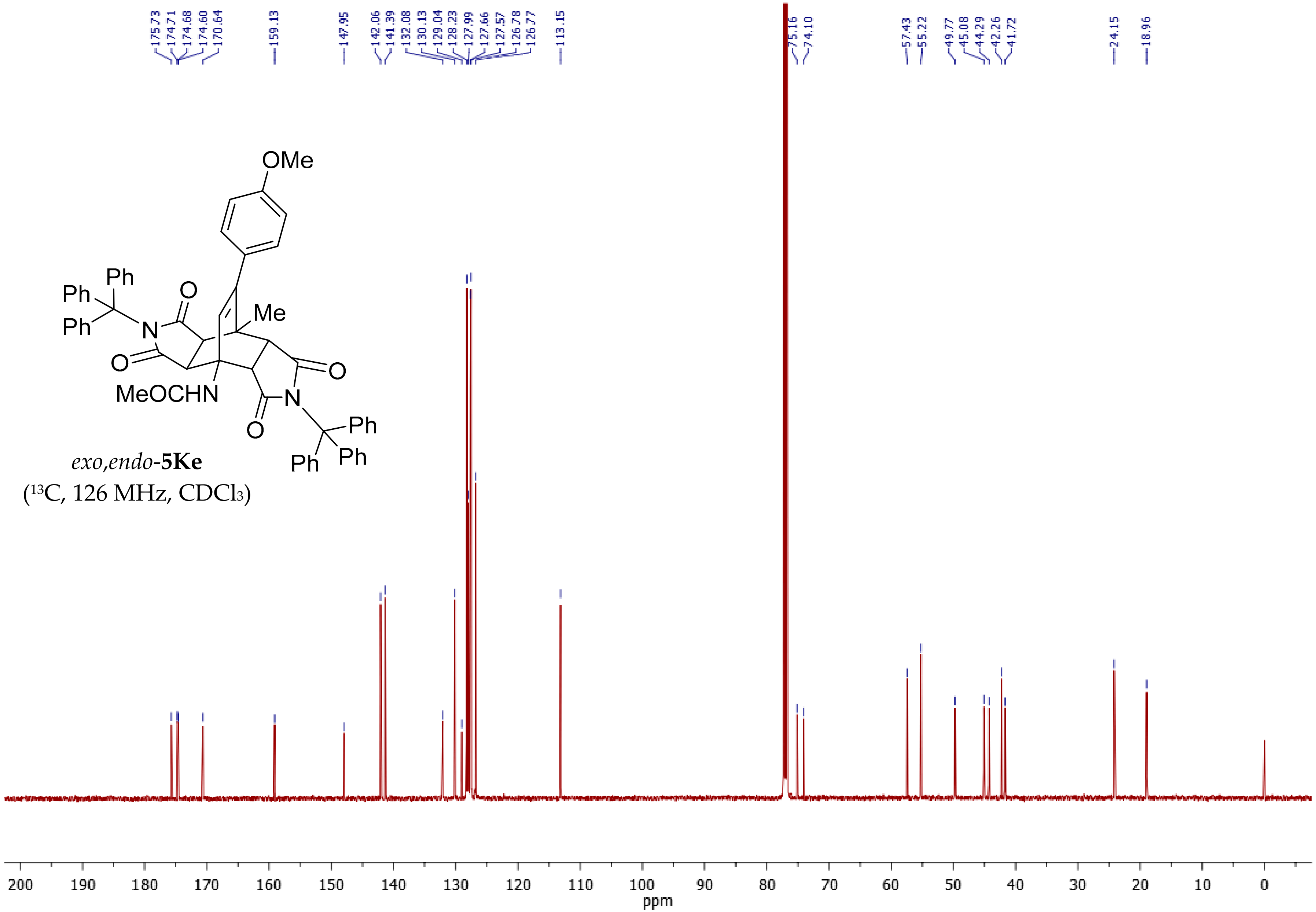

exo,endo-**5Ke**
(^{13}C, 126 MHz, $CDCl_3$)

exo,endo-**5Aa**
(HSQC, DMSO-d_6)

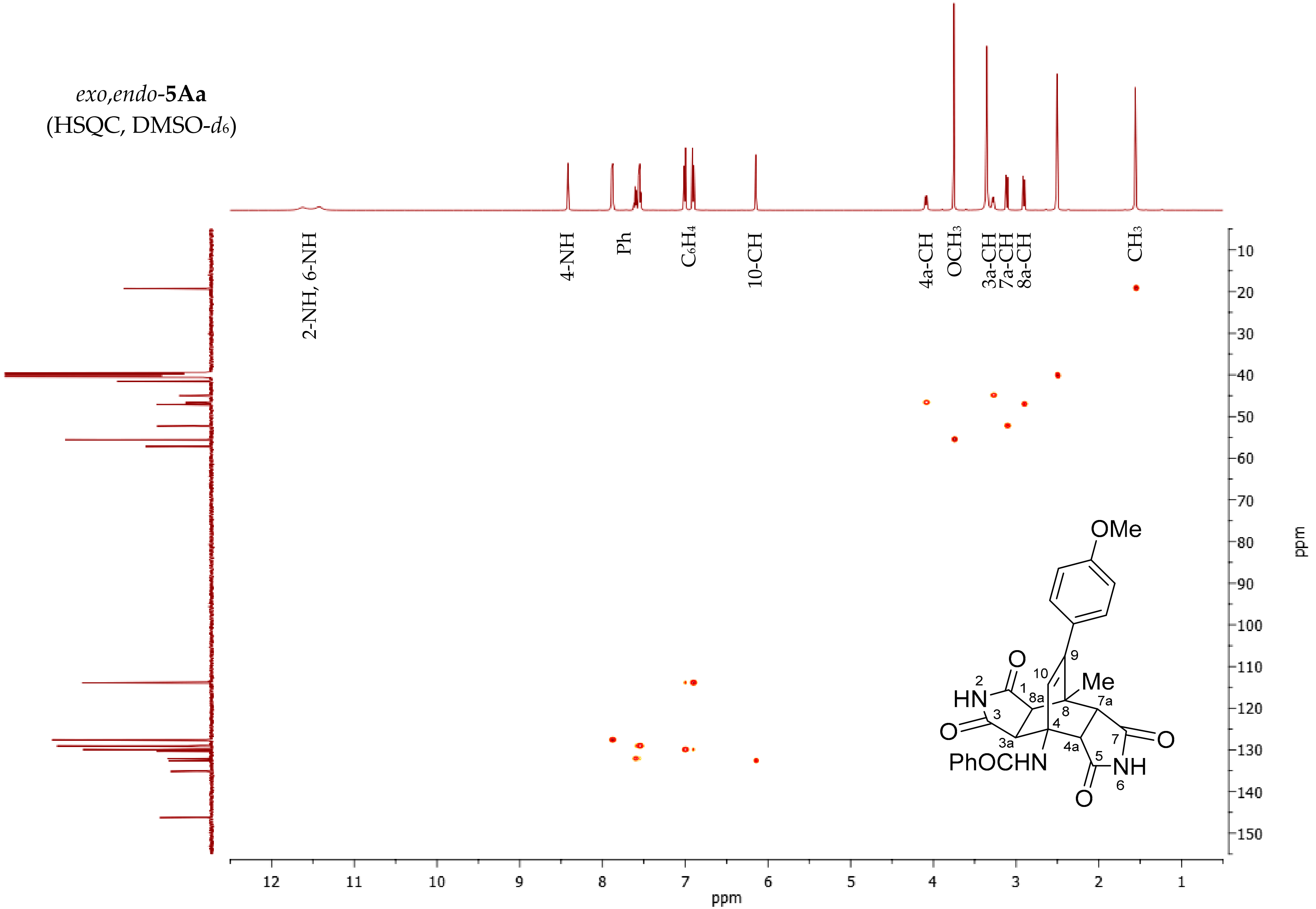

exo,endo-**5Aa**
(HMBC, DMSO-d_6)

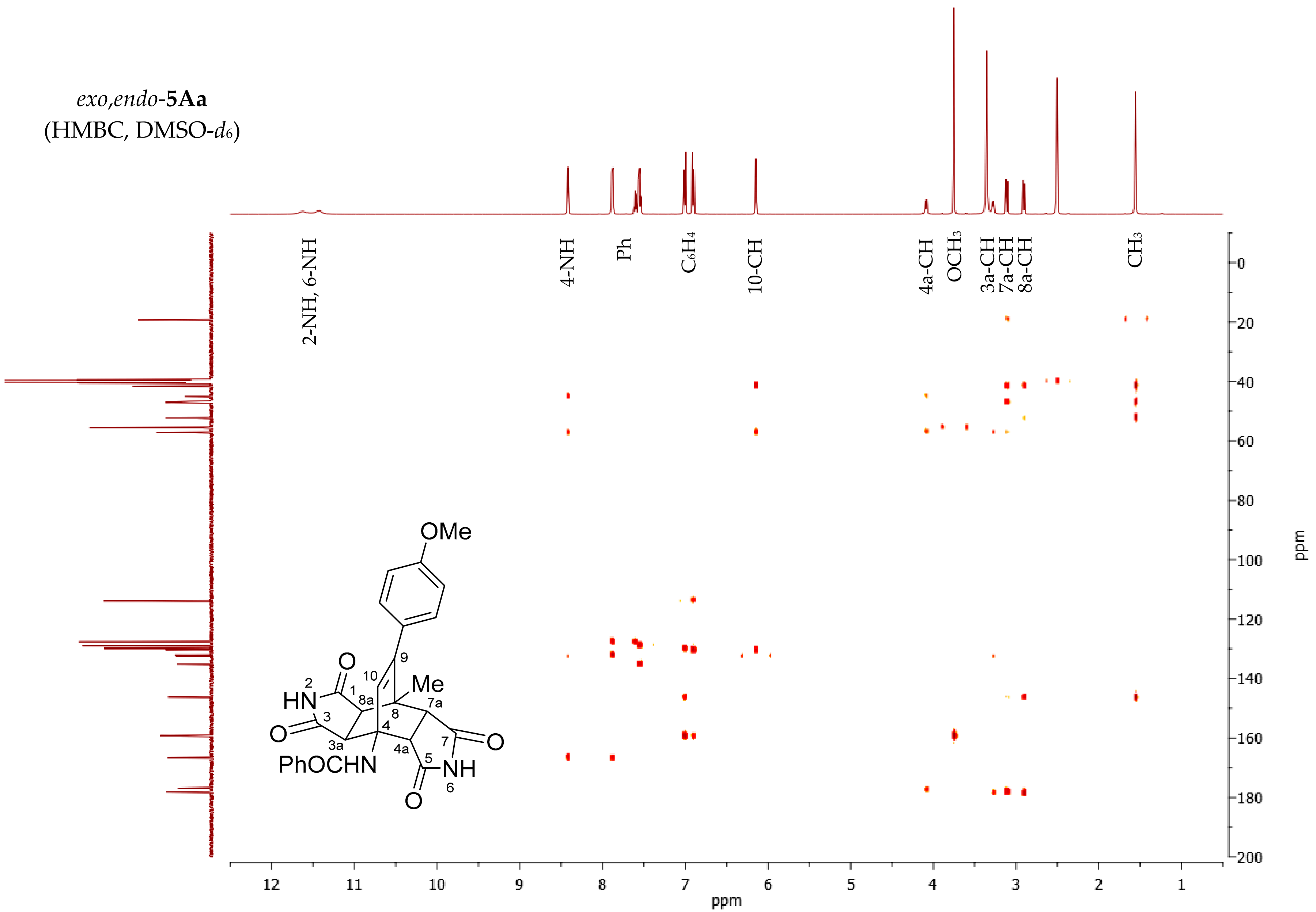

exo,exo-**5Aa**
(HSQC, DMSO-d_6)

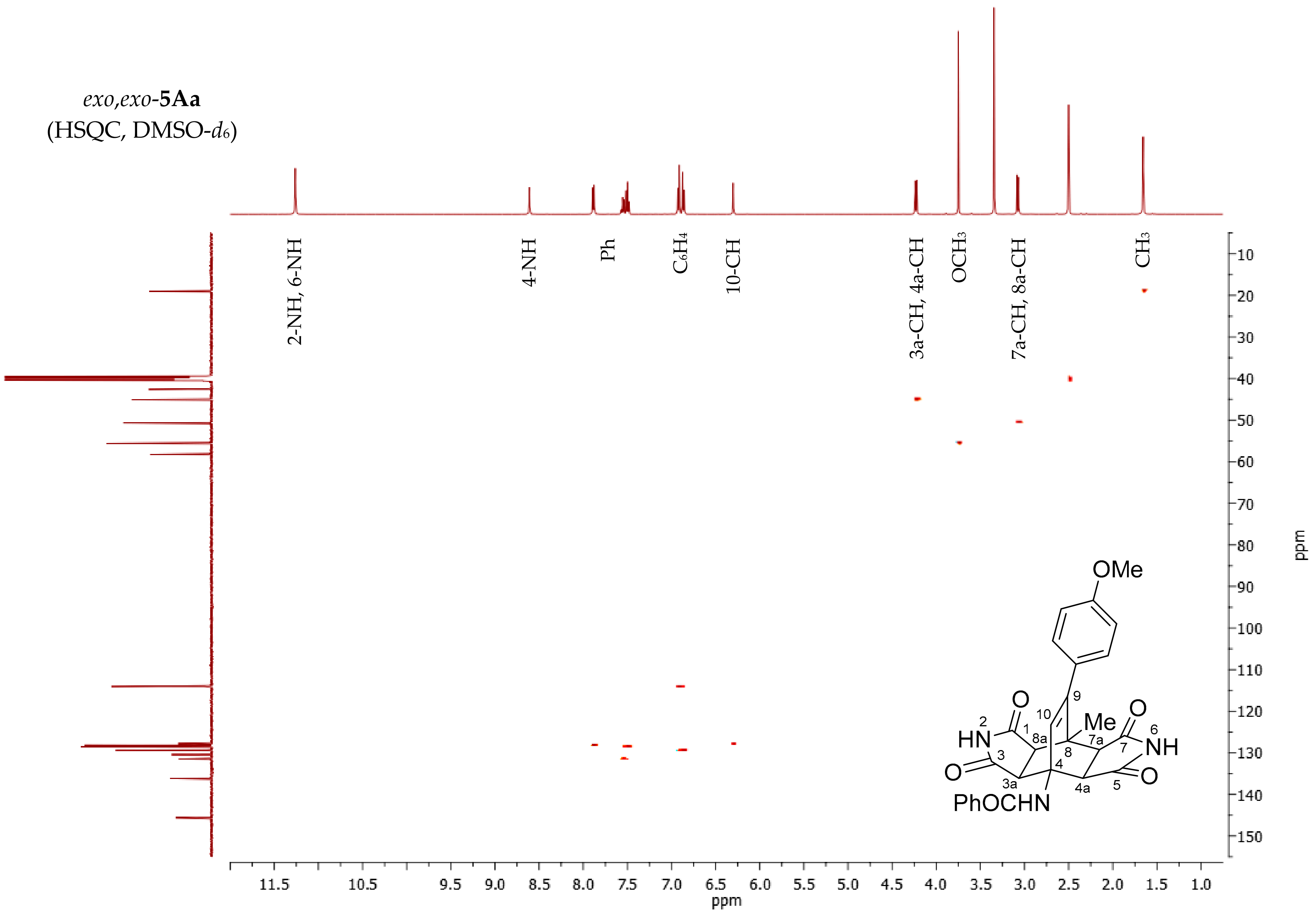

exo,exo-**5Aa**
(HMBC, DMSO-d_6)

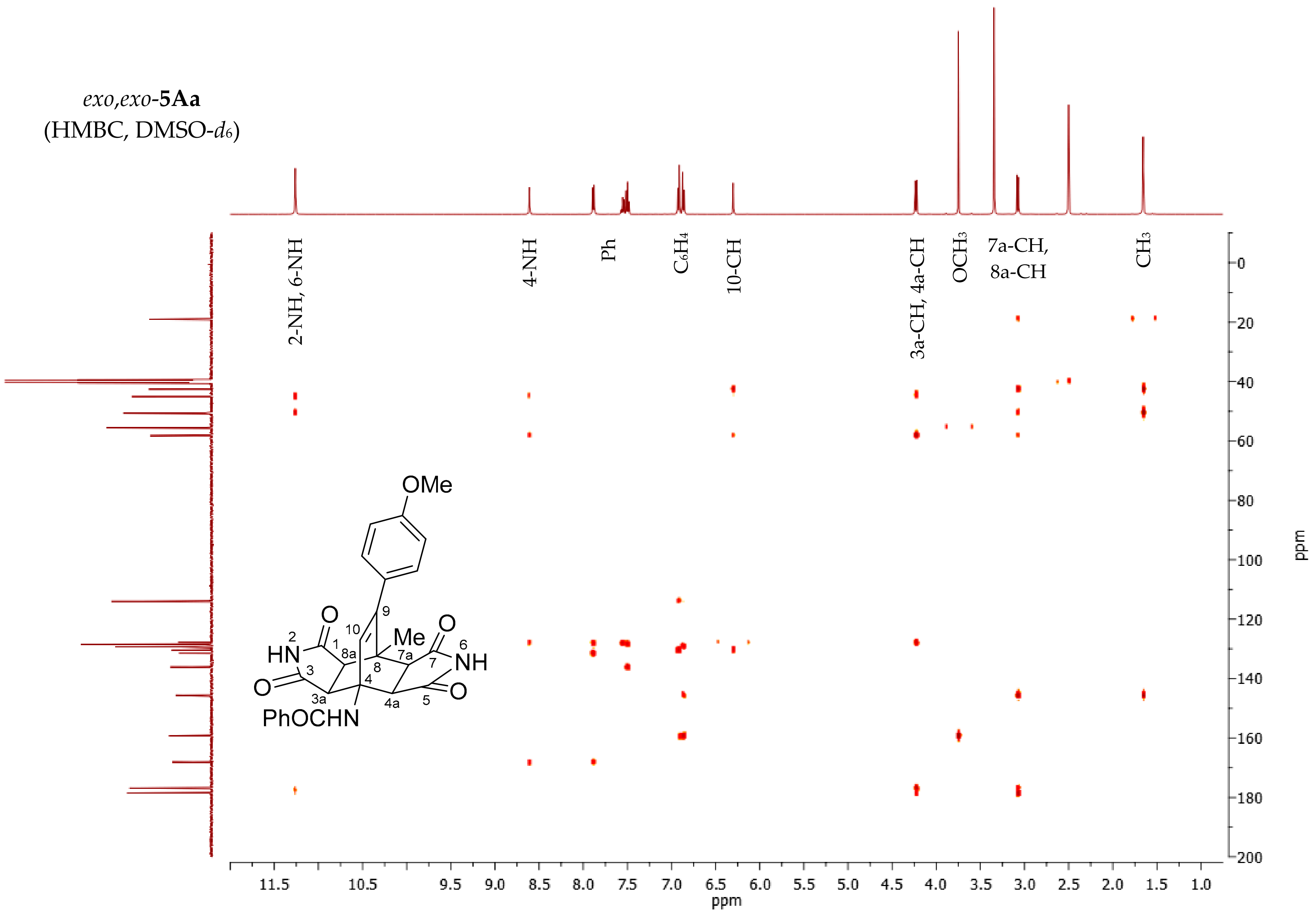

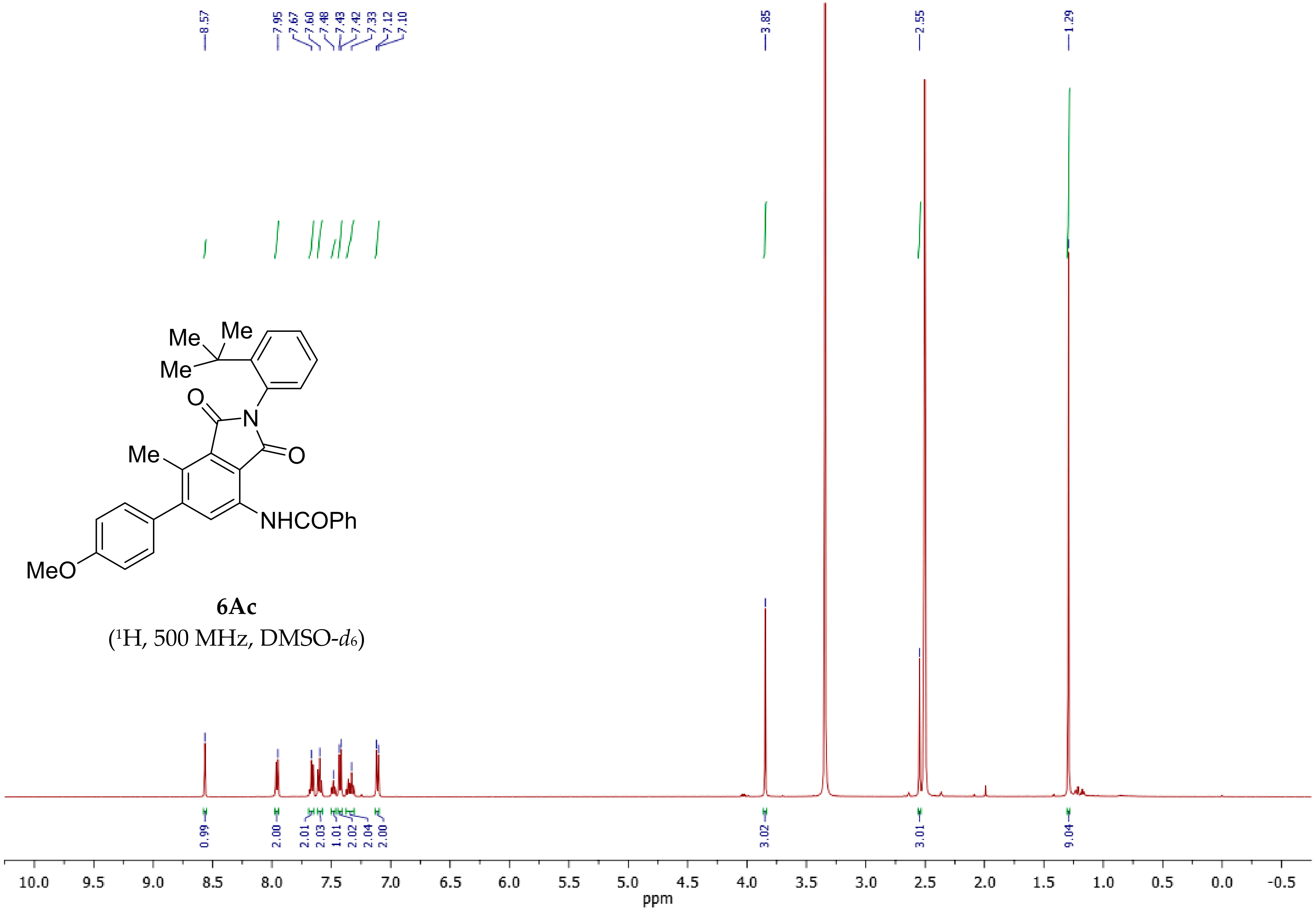

6Ac

(^{1}H, 500 MHz, DMSO-d_6)
